# A tendril perversion in a helical oligomer: trapping and characterizing a mobile screw-sense reversal†
†Electronic supplementary information (ESI) available. CCDC X-ray crystal data for **1a** and **1b** have been deposited with the CCDC, deposition numbers 1518806 and 1518807. For ESI and crystallographic data in CIF or other electronic format see DOI: 10.1039/c6sc05474a



**DOI:** 10.1039/c6sc05474a

**Published:** 2017-01-25

**Authors:** Michael Tomsett, Irene Maffucci, Bryden A. F. Le Bailly, Liam Byrne, Stefan M. Bijvoets, M. Giovanna Lizio, James Raftery, Craig P. Butts, Simon J. Webb, Alessandro Contini, Jonathan Clayden

**Affiliations:** a School of Chemistry , University of Bristol , Cantock's Close , Bristol BS8 1TS , UK . Email: j.clayden@bristol.ac.uk; b Dipartimento di Scienze Farmaceutiche − Sezione di Chimica Generale e Organica “Alessandro Marchesini” , Università degli Studi di Milano , Via Venezian , 21 20133 Milano , Italy; c School of Chemistry , University of Manchester , Oxford Road , Manchester M13 9PL , UK; d Manchester Institute of Biotechnology , University of Manchester , 131 Princess St , Manchester M1 7DN , UK

## Abstract

Helical oligomers of achiral monomers adopt domains of uniform screw sense, which are occasionally interrupted by screw-sense reversals. These rare, elusive, and fast-moving features have eluded detailed characterization. We now describe the structure and habits of a screw-sense reversal trapped within a fragment of a helical oligoamide foldamer of the achiral quaternary amino acid 2-aminoisobutyric acid (Aib). The reversal was enforced by compelling the amide oligomer to adopt a right-handed screw sense at one end and a left-handed screw sense at the other. The trapped reversal was characterized by X-ray crystallography, and its dynamic properties were monitored by NMR and circular dichroism, and modelled computationally. Raman spectroscopy indicated that a predominantly helical architecture was maintained despite the reversal. NMR and computational results indicated a stepwise shift from one screw sense to another on moving along the helical chain, indicating that in solution the reversal is not localised at a specific location, but is free to migrate across a number of residues. Analogous unconstrained screw-sense reversals that are free to move within a helical structure are likely to provide the mechanism by which comparable helical polymers and foldamers undergo screw-sense inversion.

## Abstract 2

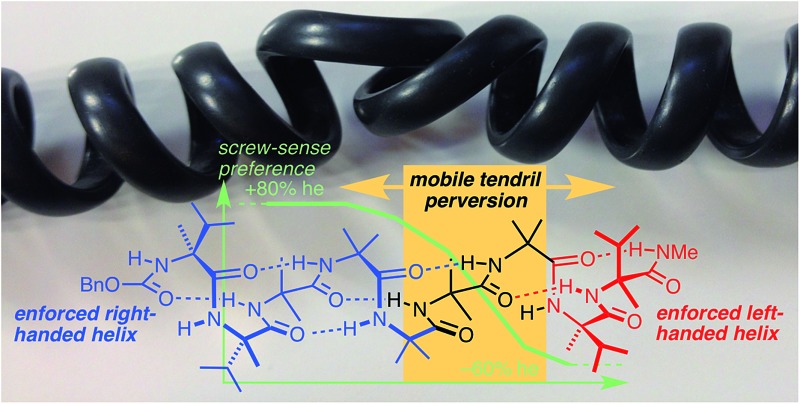
Inducing opposite screw senses at the two ends of a helix traps a helix reversal, allowing it to be characterised spectroscopically.

## Introduction

**Fig. 1:**
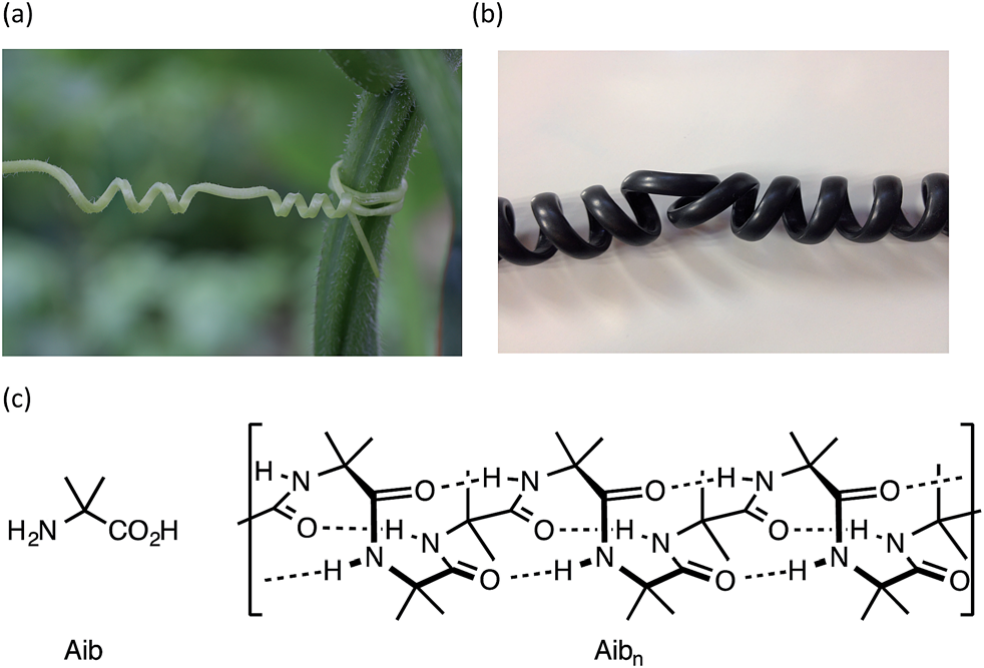
Helical perversions in (a) a tendril of the garden cucumber, *Cucumis sativa*, and (b) the cord of a telephone handset; (c) Aib and a portion of its 3_10_ helical homo-oligomer, shown in its right-handed screw-sense conformation.

Rigid macroscopic helical structures are either entirely left handed (most ‘spiral’ staircases), or entirely right handed (most bolts and screws).^1^ However, macroscopic helices with some flexibility of structure,^2^ such as the tendrils of climbing plants or the cord of a telephone handset, may contain stretches of uniform helical screw sense interrupted by ‘tendril perversions’,^3,4^ in which the screw sense reverses from left to right or right to left (Fig. 1a and b). These perversions are structures of local mirror symmetry that impart a kink to the helical structure.^5–7^

On a molecular scale, helices are common features of polymeric structures.^8–14^ While polymers of chiral monomers typically adopt a single screw sense, polymers of achiral monomers may adopt helical structures in which the screw sense is left-handed or right-handed, or even both, with domains of opposite screw sense present within a single helical structure.^15–20^ The lengths of these domains of uniform screw-sense have been evaluated in certain classes of polymer and oligomer,^16,21,22^ but the structure and dynamics of the screw-sense reversals that separate them have not been explored. A screw-sense reversal provides a molecular analogy of the macroscopic tendril perversion, and in this paper we describe synthetic, spectroscopic, and computational work allowing us to trap and characterize these dynamic structural features.

Oligomers of the achiral quaternary amino acid Aib form hydrogen-bonded 3_10_ helices^23–30^ (Fig. 1c) that invert rapidly (>1000 per second at room temperature^31,32^). In an achiral oligomer the two screw-sense conformers are necessarily equally populated, but covalent^32–43^ or non-covalent^44–48^ bonding to a chiral species may induce a local preference for either right-handed (*P*) or left-handed (*M*) screw sense. This conformational preference propagates through the oligomer, but decreases detectably with distance from the chiral inducer.^49^ By monitoring the decay of a terminally-induced screw-sense preference with increasing distance from the helix terminus, the intrusion of screw-sense reversals into the otherwise uniform helical polyamide structure can be accurately quantified.^22^ Screw-sense reversals are rare, but are more common in polar, hydrogen bonding solvents, and at higher temperatures. In THF, for example, any single Aib residue has 0.5% chance of hosting a screw-sense reversal; in MeOH this figure rises to 6%. Their scarcity, along with their rapid migration along the oligomer chain (which provides a plausible mechanism for the remarkably rapid kinetic screw-sense inversion of Aib oligomers^50^) has made structural characterization of this elusive motif^51^ particularly challenging.

## Trapping a helical reversal within a screw-sense mismatched domain

Our method for quantifying the probability of screw-sense reversals was based on the fact that a helical structure with the same screw sense at each terminus must contain an even number of screw-sense reversals, while a helical structure with a left-handed screw sense at one terminus and a right-handed screw sense at the other must contain an odd number of screw-sense reversals.^52^ Thus, a helical structure in which the termini are forced to adopt opposing screw senses must contain at least one reversal, and such a molecule provides a scaffold for trapping the dynamic reversal motif within an oligo-Aib domain.

**Fig. 2:**
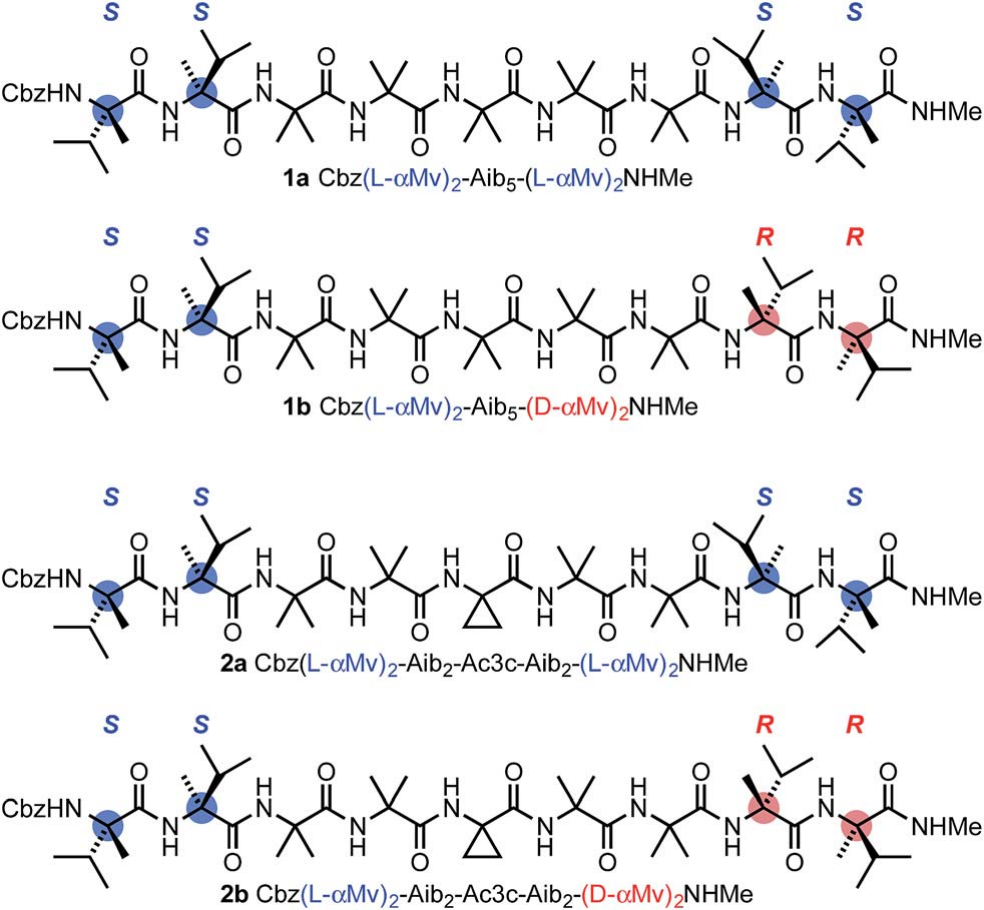
The diastereoisomeric pairs of compounds **1a** and **1b** and **2a** and **2b**. Cbz = BnOCO.

Using solution-phase methods^49,53^ for synthesizing hindered peptides (see ESI†), we made the pairs of diastereoisomeric molecules **1a** and **1b** and **2a** and **2b** (Fig. 2). In each of **1a** and **1b**, a domain formed from five Aib residues is capped at each terminus by a homochiral pair of α-methylvaline (α-MeVal) residues. Like Aib, the hindered quaternary amino acid α-methylvaline favours 3_10_ helical conformations,^54,55^ but its *S* enantiomer is accommodated preferentially by a right-handed helix, and its *R* enantiomer by a left-handed helix. Thus l-(α-MeVal)_2_ is a powerful inducer of local *P* screw-sense, and d-(α-MeVal)_2_ a powerful inducer of local *M* screw-sense, to the extent that l-(α-MeVal)_2_ induces quantitative adoption of a right-handed screw sense in an Aib oligomer.^35,49^

As a consequence, we expected both termini of **1a** to adopt the same screw sense, while in **1b** we expected a right-handed screw sense to be enforced at the N-terminus and a left-handed screw sense to be enforced at the C-terminus. The screw-sense mismatch in **1b** should ensure that the central Aib_5_ domain always contains a helical reversal, while the matched screw senses at the termini of **1a** make the formation of a helical reversal unlikely. Diastereoisomers **2a** and **2b** present a stereochemically analogous pair, but with the central Aib residue replaced by aminocyclopropylcarboxylic acid Ac3c. Achiral quaternary residues other than Aib may also favour 3_10_ helical conformations,^23,56,57^ but residues related to Ac3c have also been associated with the γ-turn screw-sense reversal motif.^58^ The contrast in structure between **1a** and **1b** and the analogous diastereoisomeric pairs was explored by a variety of analytical and computational techniques, with the aim of identifying the distinguishing features of **1b** and **2b** that could allow characterization of the screw-sense reversal.

**Fig. 3:**
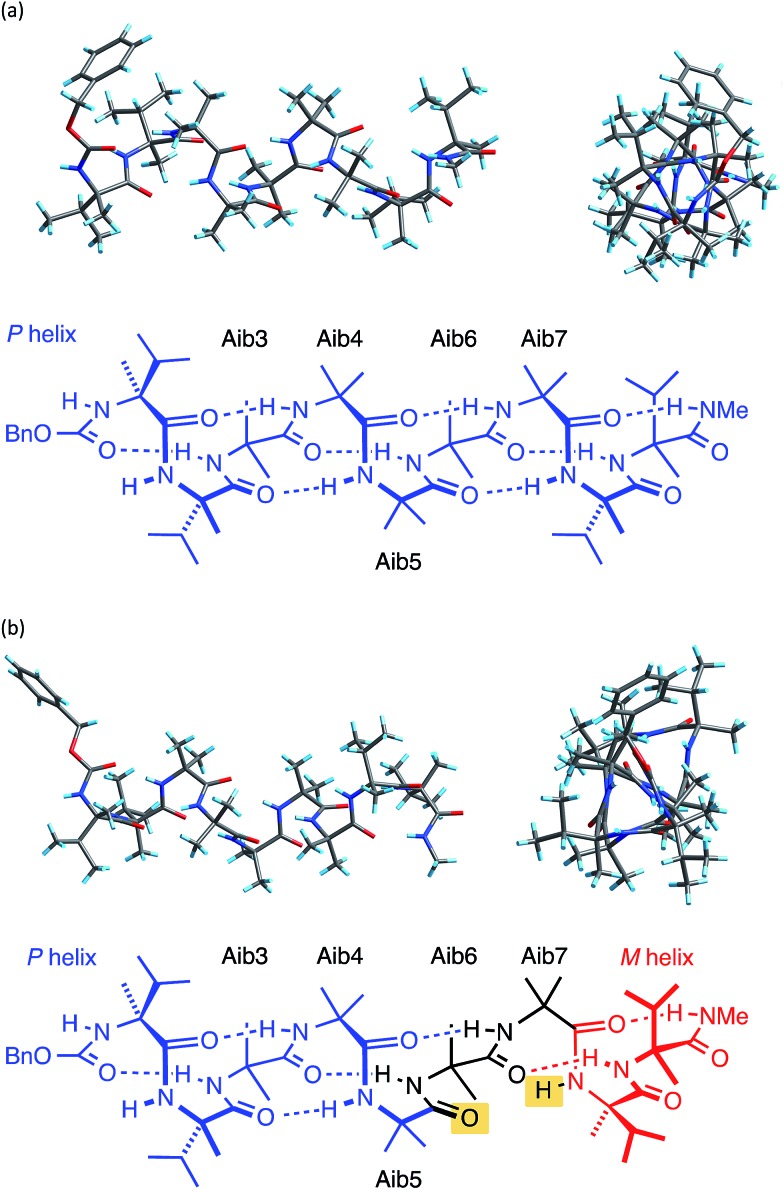
X-ray crystal structure viewed along, and perpendicular to, the helical axis, along with a schematic diagram of the hydrogen bonding in (a) **1a** and (b) **1b**. Non-hydrogen bonded C=O and N–H are highlighted in yellow.

The X-ray crystal structure† of the ‘matched’ oligomer **1a** (Fig. 3a) revealed a well-formed 3_10_ helix with a right-handed screw sense, fully in accordance with structures reported in the literature.^27–30,59,60^ Dihedral angles *φ* and *φ* (see Table 1) closely approximate those of an idealized 3_10_ helix (*φ* = –49°; *φ* = –26°). By contrast, the X-ray crystal structure† of the screw-sense mismatched **1b** (Fig. 3b) showed, as expected, a right-handed helix at the N-terminus and a left-handed helix at the C-terminus, with a switch from right- to left-handed screw sense happening at residue 6. The change in the sign of the dihedral angles at this point in the structure (Table 1, rows indicated ‘X-ray’) confirms this localized screw-sense reversal in the solid state. The conformational consequence of the reversal becomes clear by viewing the helix end-on: the reversal takes a form that has the general appearance of an antisymmetric tendril perversion;^7^ the amide carbonyl groups present their *Re* face to the outside of the helix in the N-terminal *P* domain and their *Si* face to the outside of the helix in the C-terminal *M* domain. At the point of the helical reversal in **1b**, there is one unsatisfied hydrogen bond donor (the NH of α-MeVal8) and one unsatisfied hydrogen bond acceptor (the C=O of Aib5), as illustrated in Fig. 3b.

**Table 1:** Cluster analyses of the final 50 ns of the 297.31 K REMD trajectories of foldamers **1a** and **1b**. Blue cells show right-handed screw sense; red cells show left-handed screw sense; green cells highlight γ-turn motifs; yellow cells highlight C=O groups with unsatisfied hydrogen bonds. Dihedral angles are illustrated by the X-ray crystal structure of **1a**

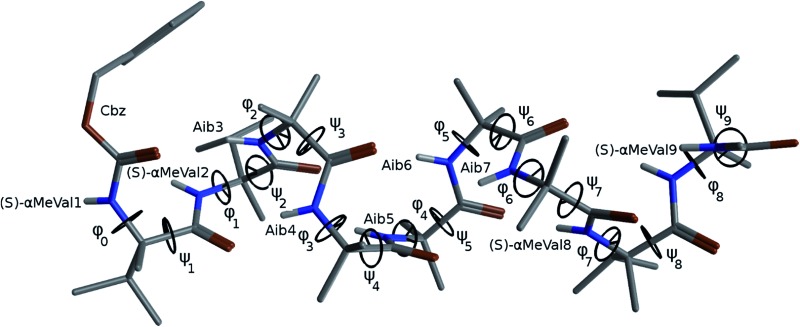
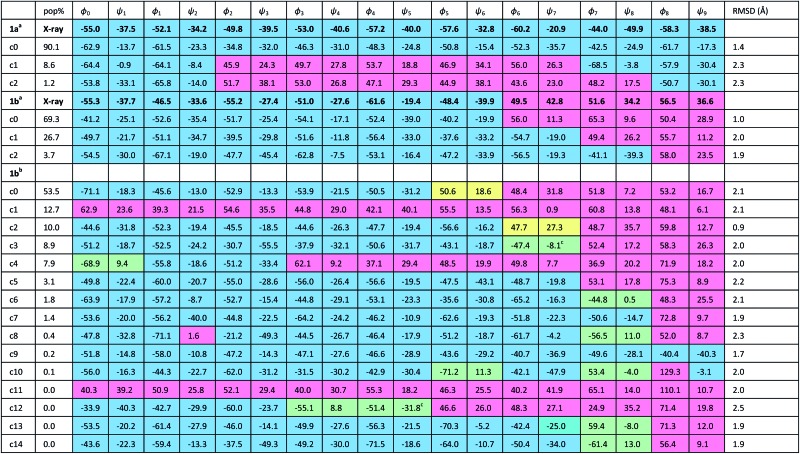

^*a*^REMD analysis in an implicit solvent (water) [only the three most populated clusters (c0–c2) are shown].

^*b*^REMD analysis in explicit methanol.

^*c*^Distorted γ-turn.

**Fig. 4:**
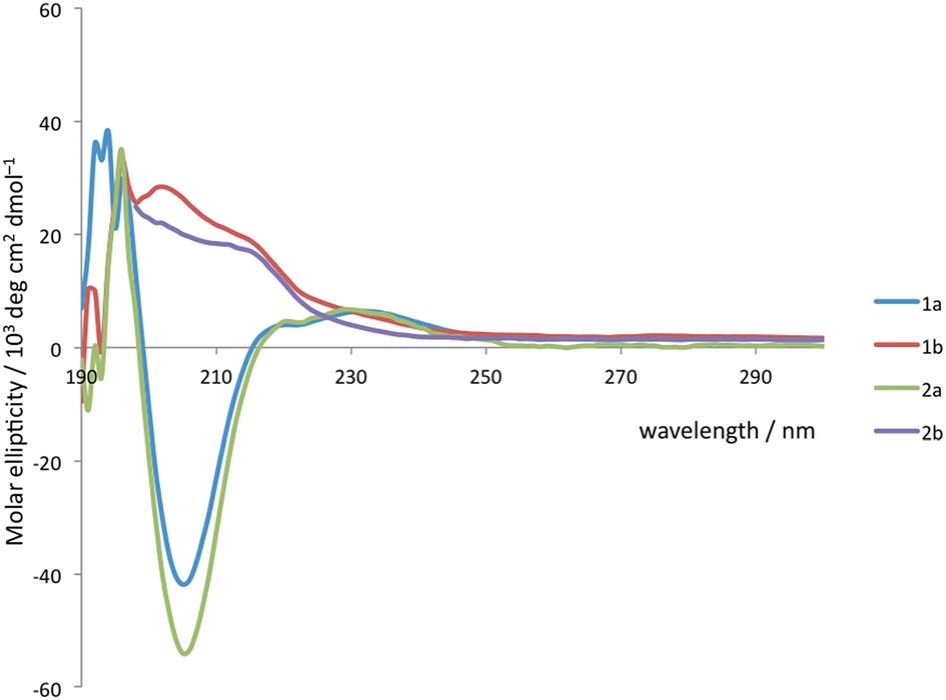
CD spectra of **1a** and **b** and **2a** and **b** recorded at 2.4 × 10^–4^ M in MeOH at 20 °C.

Solution state studies were carried out using circular dichroism (CD) and NMR. CD in methanol suggested that the solution state conformational preferences of **1a** and **2a** were consistent with the crystal structure of **1a**: the CD spectra are characteristic of an Aib-containing 3_10_ helix, showing a clear negative maximum at 208 nm (Fig. 4), consistent with the formation of a right-handed helix in solution.^38,49^ By contrast, the form of the CD spectra of **1b** and **2b** does not correspond closely to that expected for a 3_10_ helix, suggesting that neither an *M* nor a *P* 3_10_ helix predominates. The CD spectra are less intense than those of **1a** and **2a**, suggesting a less well-defined conformation in solution, and consistent with several possible situations: the adoption of stable *M* and *P* screw senses in separate domains within each molecule, the population of a dynamic, interconverting mixture of screw senses across an ensemble of molecules, or a population of conformations rich in non-3_10_-helical motifs.

To distinguish between these alternatives, Raman spectra of **1a** and **1b** were acquired in chloroform (see ESI, Fig. S1†). In the amide I region both spectra show a band at 1664 cm^–1^, and deconvolution of this band showed it to be composed of a single contributor for both **1a** and **1b**. Amide I bands at ∼1660 cm^–1^ have been ascribed to Aib oligomers in 3_10_ helical conformations,^61^ so these Raman spectra are consistent with foldamer populations that have significant 3_10_ helix content in either single or multiple domains.

**Fig. 5:**
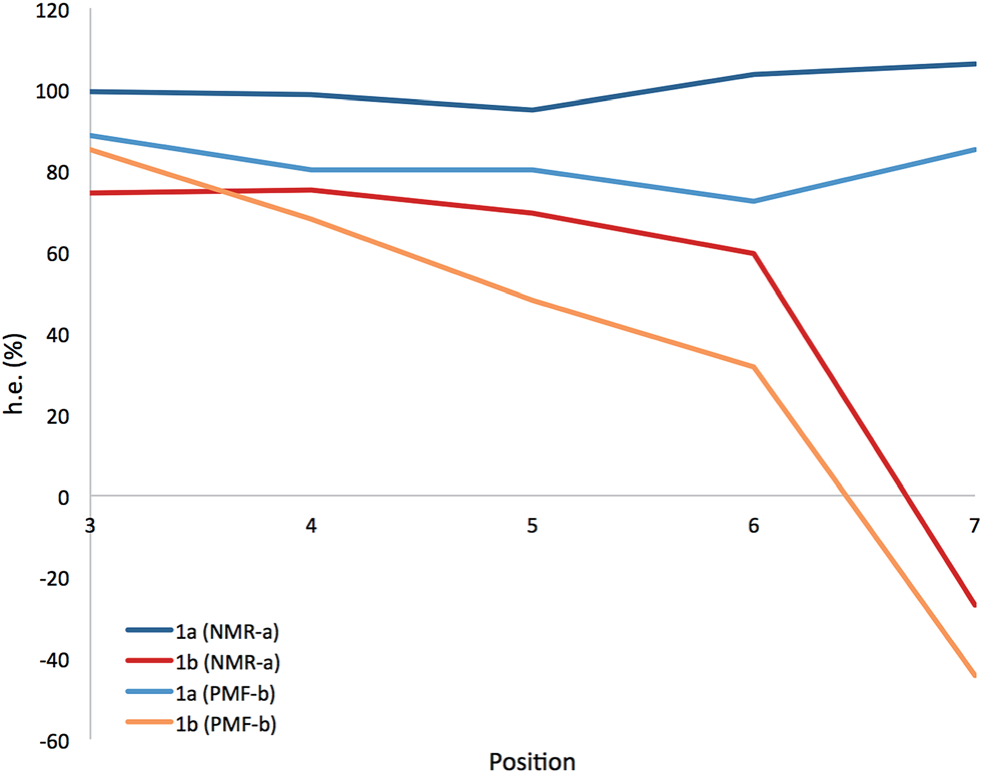
Variation of helical excess with position in the chain for the achiral Aib5 domain of **1a** and **1b** (a) calculated from the chemical shift difference Δ*δ* in the ^13^C NMR spectrum; (b) calculated from the Boltzmann distributions resulting from PMF profiles in implicit solvent (see below).

A more detailed picture of the solution-state conformational populations of **1a** and **1b** was gained by NMR spectroscopy. The screw-sense preference (‘helical excess’, or h.e., defined as the excess population of one screw-sense conformer over the other^35^) at specific residues in an Aib helix may be quantified by measuring the chemical shift difference (anisochronicity, Δ*δ*) between the ^13^C signals of the Aib residue's methyl groups.^22,32,49^ Using ^1^H–^13^C HMBC experiments to assign the paired signals of the diastereotopic methyl groups within each Aib residue of **1a** and **1b**, and a combination of ^1^H–^15^N and ^1^H–^13^C HSQC to confirm their location in the chain, we quantified the chemical shift separations Δ*δ* in MeOH at 23 °C for the pairs of methyl groups corresponding to each of the residues Aib3–Aib7. Fig. 5 shows the variation of Δ*δ* with position in the chain,^62^ along with a value for the helical excess calculated using the reported slow-exchange value for Δ*δ* in related compounds.^22,32,49^

Helical excess in the ‘matched’ oligomer **1a** was more or less consistent along the entire length of the chain, as a consequence of the uniform screw sense, with, intriguingly, a slight drop in the central portion of the chain that may indicate a small population of conformers in which a central left-handed domain is flanked by two screw-sense inversions. Values of h.e. that apparently exceed 100% are likely to be a result of the proximity of Aib7 to the terminal α-MeVal residues, which may perturb the simple dependence of chemical shift on helical excess.

The chemical shift differences of the Aib residues of the ‘mismatched’ oligomer **1b** vary according to their position in the chain, being greatest at the N-terminus and falling towards the C-terminus. In the absence of enantioselective ^13^C labelling,^38,49,53,63^ the scalar value Δ*δ* cannot distinguish positive and negative values of h.e., so (with support from computational work described below) we make the assumption that the gradual reduction in h.e. from Aib3 to Aib6 represents a decreasing preference for right-handed screw sense, crossing over to a left-handed screw sense at Aib7.

The progressive, rather than instantaneous, change in Δ*δ* indicates that the screw-sense reversal located between Aib6 and Aib7 in the crystal structure of the mismatched oligomer **1b** is a mobile, dynamic feature in solution, with the shift from a right-handed screw-sense preference at the N-terminus to a left-handed screw sense at the C-terminus distributed over several residues of the chain. The change in h.e. from one residue (A) to another (B) in a dynamic helical structure can be described by a value *p*(reversal)_A–B_ for the likelihood of a screw-sense reversal being located between those two residues:
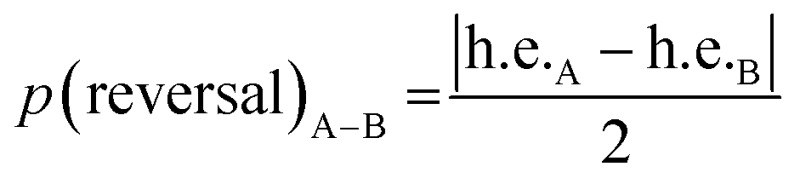
thus*p*(reversal)_A–B_ ∝ |Δ*δ*_A_ – Δ*δ*_B_|

By this reasoning, we deduce that the most likely location for a screw-sense reversal in **1b** is between residues 6 and 7.

Given that the crystallographic screw-sense reversal entails the loss of an intramolecular hydrogen bond, we carried out ^1^H NMR studies of **1a** and **1b** with the aim of identifying NH protons not participating in intramolecular hydrogen bonding. Addition of increasing quantities of DMSO (0–10%) to a solution of either **1a** or **1b** in CDCl_3_ led to significant changes in chemical shift of two NH signals, suggesting that only two NH groups have the potential for intermolecular hydrogen bonding. Natural abundance ^15^N HSQC (see ESI†) experiments on **1b** showed most of the ^15^N signals falling in a narrow band between –250 and –260 ppm, with the exception of the N-terminal carbamate ^15^N and the C-terminal amide ^15^N, which fell significantly upfield of the others, at around –280 ppm. These two upfield ^15^N signals also correspond to the two NH signals that experienced a large shift in the DMSO titrations, suggesting that in this case these NH shifts in DMSO merely show some fraying of the helix termini in polar solvent. The ^15^N spectrum of **1a** shows a tighter grouping of most ^15^N signals, mostly between –240 and –246 ppm, with three signals at –260 ppm, none of which correspond to the NH signals that shift during the DMSO titrations. Earlier studies have shown that correlations between ^15^N chemical shift and hydrogen-bond strength are subject to complex and subtle effects,^64–66^ and this data does not allow us to assign with confidence the population of hydrogen bonds experimentally.

## Computational analysis

In order to gain insight into the conformations populated by oligomer **1b** in the solution phase, we explored the structures of **1a** and **1b** computationally, using Replica Exchange Molecular Dynamics (REMD). REMD is a generalized-ensemble algorithm performing random walks in energy space, allowing exploration of the whole conformational space and statistical evaluation of the most energetically favoured conformations at a chosen temperature. It has been successfully applied to the study of conformational changes in biomolecules, including peptide folding.^67^

**Fig. 6:**
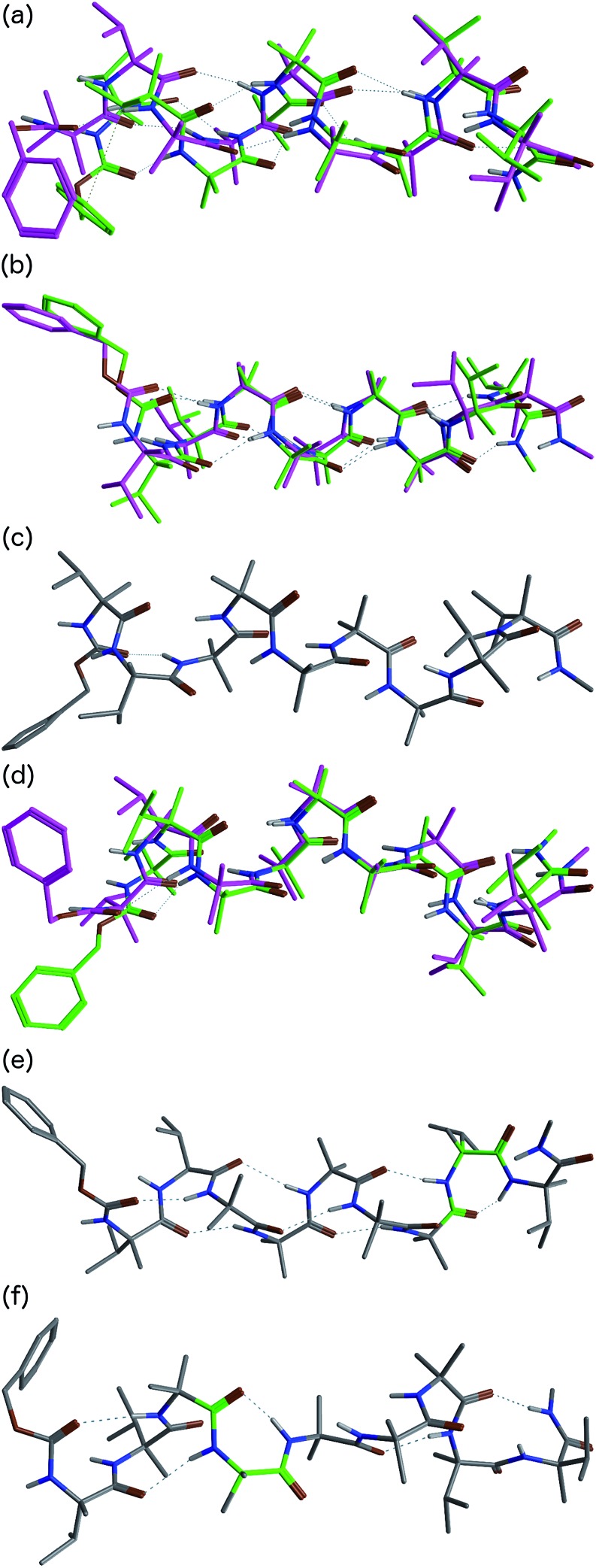
Superposition of the X-ray structures (green) and a representative structure of the most populated cluster (magenta) of (a) oligomer **1a** and (b) oligomer **1b**. (c) Representative structure of the most populated cluster of the REMD trajectory of **1b** in explicit solvent at 303.60 K. (d) Superposition of the crystallographic structure of oligomer **1b** (green) and the representative structure of cluster c2 (magenta) of the REMD trajectory in explicit solvent at 303.60 K. Representative structures of clusters (e) c6 and (f) c12 from the REMD trajectory of **1b** in explicit methanol at 303.60 K. The γ-turns associated with *i* + 2 → *i* hydrogen bonds are highlighted in green.

Cluster analyses performed on the implicit solvent trajectories of oligomers **1a** and **1b**, reported in Table 1, show that in both cases the REMD simulations reproduce the crystallographic data (Fig. 6). The most populated clusters calculated for oligomers **1a** (90.1%) and **1b** (69.3%) have a root-mean-squared (RMS) deviation from the backbone of the corresponding X-ray structures of 1.4 Å and 1.0 Å respectively, and the computation and crystallographic structures are essentially superimposable (Fig. 6a and b). The most populated cluster of oligomer **1a** is a continuous right-handed 3_10_ helix, while that of oligomer **1b** is a right-handed 3_10_ helix from the N-terminus to Aib6 and a left-handed 3_10_ helix from Aib7 to the C-terminus.

The most stable hydrogen bonds (occupancies >50%: see Table S1, ESI†) involve *i* + 3 and *i* residues and form β-turns that build up the 3_10_ helix identifiable in the crystal structures (Fig. 6a and b and Table S1, ESI†). However, the occupancies of the hydrogen bonds between d-αMeVal8 and Aib5 and between d-αMeVal9 and Aib6 of peptide **1b** are about 20% lower than the equivalent hydrogen-bonded interactions between l-αMeVal8 and Aib5 and between l-αMeVal9 and Aib6 of oligomer **1a**, as a result of the screw-sense reversal. The reversal also manifests itself in the population distribution among the clusters. The most populated conformational cluster of oligomer **1a**, corresponding to the right-handed 3_10_ helix, comprises >90% of the total. Simulation of oligomer **1b** suggests a less uniform conformational preference, with two major clusters, each having a screw-sense reversal at a different point along the chain, between residues 6 and 7, or 7 and 8 (Table 1).

Given the importance of hydrogen bonding in the detailed solution phase structures of **1a** and **1b**, and the possible association of the screw-sense reversal with the loss of an intramolecular hydrogen bond,^15,26^ a second REMD simulation of oligomer **1b** was performed with methanol as an explicit solvent (Table 1). A few differences from the earlier simulation in implicit solvent were evident. The representative structure of the most populated cluster (53.5%) now showed a helical screw-sense reversal between Aib5 and Aib6, with an RMS deviation from the crystallographic structure of 2.06 Å (Fig. 6c). Nonetheless, the representative structure of cluster c2 (10.0%) has a conformation that is superimposable on the X-ray structure with a RMSD of 0.90 Å (Fig. 6d).

The screw-sense reversal in clusters c0 and c2 is accompanied by an unsatisfied hydrogen bond, as seen in the X-ray crystal structure (Fig. 3b). Additionally, some minor clusters (*e.g.* c3, c4, c6, c8, c10, c12–14: Table 1 and Fig. 6e and f) clearly display γ-turns, which involve *i* + 2 → *i* hydrogen bonds at various points along the chain. The γ-turns are all associated with screw-sense reversals. Hydrogen-bond analysis of the REMD trajectories in explicit methanol (Table S2, ESI†) indicate that while these γ-turns can involve any residue, the highest occupancies of *i* + 2 → *i* hydrogen bonds are those between Aib7 and Aib5, d-αMeVal8 and Aib6 and d-αMeVal9 and Aib7.

From these data, we deduce that explicit methanol lowers the barriers for the reversal of screw sense at any point along the chain, possibly through the stabilization of γ-turn intermediates. The near-planar γ-turns occur more frequently at the boundary between domains of opposite helical screw sense. A γ-turn has been observed centred on a quaternary derivative of Ac3c,^58^ and we are currently exploring the hypothesis that γ-turns may play a role in the kinetic mechanism of screw-sense inversion.^50^

**Fig. 7:**
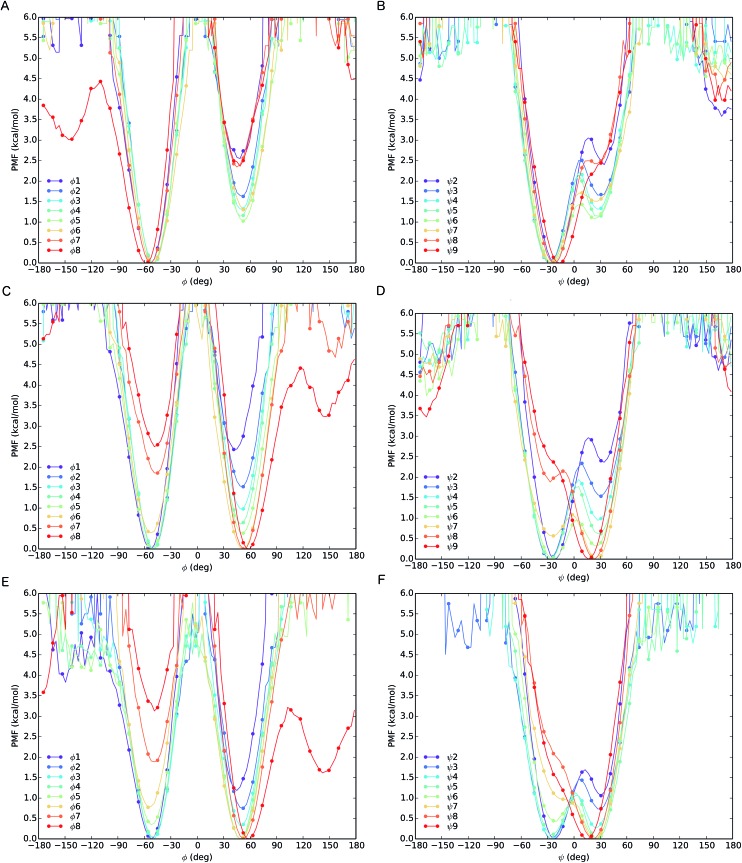
PMF as a function of *φ* (A) and *ψ* (B) dihedrals for oligomer **1a** and of *φ* (C) and *ψ* (D) dihedrals for oligomer **1b** from REMD simulations in implicit solvent. PMF as a function of *φ* (E) and *ψ* (F) dihedrals for oligomer **1b** from REMD simulation in explicit MeOH.

The different behaviour of the two oligomers **1a** and **1b** is confirmed by potential of mean force (PMF) profiles as a function of *φ* and *ψ* dihedral angles (Fig. 7), initially obtained from the implicit solvent trajectories. Two minima are evident for each dihedral angle, with the values of *φ* = ±50° and *ψ* = ±30° corresponding to the right- and left-handed 3_10_ helical conformations. The barriers to interconversion between these enantiomeric conformations of *φ* are significantly higher than the barriers to interconversion between the enantiomeric conformations of *ψ*. PMF profiles of oligomer **1a** show a consistent preference for a right-handed helical conformation (*φ* = –50°; *ψ* = –30°) at every residue in the chain, with the preference being strongest (*ca.* 2.5 kcal mol^–1^) near the termini and weakening (to about 1 kcal mol^–1^) in the middle of the chain (residues 4–6). For oligomer **1b**, the PMF profiles show a right-handed conformational preference at the N-terminus (2.5 kcal mol^–1^ at residues 1, 2) which steadily decreases through residues 3–6. Dihedral angles *φ*_3–5_ and *ψ*_4–6_ display a progressive reduction in the energy difference between the two minima, culminating in a reversal of the screw-sense preference on passing *φ*_6_ and *ψ*_7_ at Aib7. Through the remaining dihedral angles *φ*_6–8_ and *ψ*_7–9_, the global minimum corresponding to the left-handed helix becomes progressively more stable. The change in distribution as a function of position in the chain suggests that the inversion from right-handed to left-handed screw sense in **1b** occurs most commonly between Aib6 and Aib7, but that conformers with the reversal between Aib5 and Aib6 or between Aib7 and αMeVal8 are not greatly disfavoured.

In order to model more closely the conformation in methanol solution, PMF profiles were also calculated for **1b** in explicit methanol (Fig. 7). Both the energetic differences between the two screw-sense conformers and the barriers to screw-sense inversion were lower than in implicit solvent. Importantly, in explicit methanol the right-handed screw sense is favoured from the N-terminus only as far as Aib4. Aib5 has almost equal stability in either screw sense, and from Aib6 to the C-terminus the left-handed screw sense is favoured. Thus, overall, REMD simulations and PMF analysis suggest that in explicit methanol the screw-sense reversal is more mobile and more likely to be found closer to the centre of the oligo-Aib domain, close to Aib5, than in implicit solvent, where the reversal is found to be most likely between Aib6 and Aib7.

The populations at 300 K of left- and right-handed screw-sense conformers at each residue were calculated from the Boltzmann distributions that arise from these PMF energy differences (see ESI, Tables S3–S5†). For comparison, the values derived from NMR experiments are shown in Fig. 5 for **1a** and **1b** in implicit solvent. Values obtained from the implicit solvent simulation match better with the NMR data than those computed from the explicit methanol REMD simulation. However, the differences observed are within the internal error of the method and result from the statistical nature of PMF analysis.

## Locating the screw sense reversal spectroscopically

In agreement with the NMR data (Fig. 5), the computational predictions suggested that in the solution phase the screw-sense reversal in **1b** is mobile, but is most likely to be found in the vicinity of residues Aib5, Aib6 or Aib7 (in other words, between the middle of the oligo-Aib domain and its C-terminal end), depending on the method of simulation. Guided by this information we made a series of compounds with the aim of locating the reversal spectroscopically by methods that allow the differentiation of left- and right-handed screw-sense preference.

**Fig. 8:**
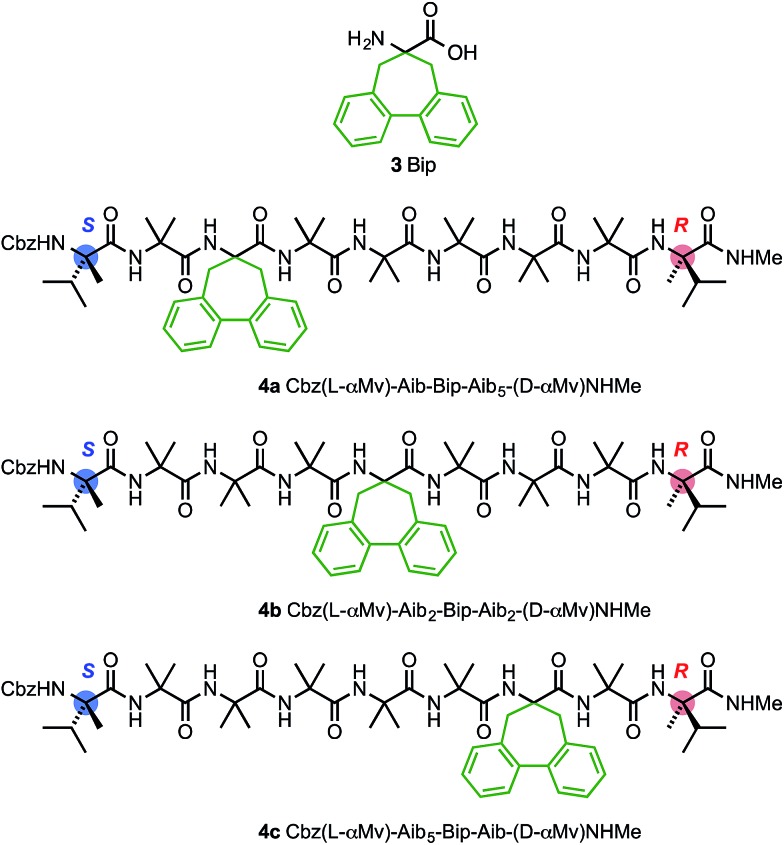
Bip **3** and Bip-containing oligomers **4a–c**, with opposing terminal screw-sense preferences and the CD-responsive Bip residue at positions 3, 5 and 7.

Circular dichroism generally provides an overview of the global conformation of a peptide oligomer through bands located around 200–220 nm. Mazaleyrat *et al.* have used the conformationally responsive aromatic amino acid Bip **3** to provide a more detailed local probe of screw-sense preference in the form of the sign of its Cotton effect at 247 nm.^68–70^ We reasoned that a series of oligomers **4** in which a Bip probe was stationed at successive positions along the chain would allow us to extract local information about screw sense and thus gather evidence for the location of a trapped reversal. A series of three structures **4a–c** were made in which a Bip residue forms the second, fourth or sixth of a series of seven achiral residues, the remainder being Aib (Fig. 8). Owing to the synthetic challenges associated with the repeated hindered couplings required to couple an αMeVal dimer to each end of the oligomer, structures **4a–c** were made with a single l-αMeVal residue at the N-terminus and a single d-αMeVal at the C-terminus. Cbz-l-αMeVal is reported^35^ to induce a *P* screw-sense preference of ∼50% h.e. at a site four residues from the N-terminus of an Aib oligomer and d-αMeValNHR an *M* screw-sense preference of ∼70% h.e. at a site four residues from the C-terminus.^36^ The mismatch between these approximately equal and opposite screw-sense preferences should, as in **1b** and **2b**, trap a screw-sense reversal within the achiral domains of **4a–c**.

**Fig. 9:**
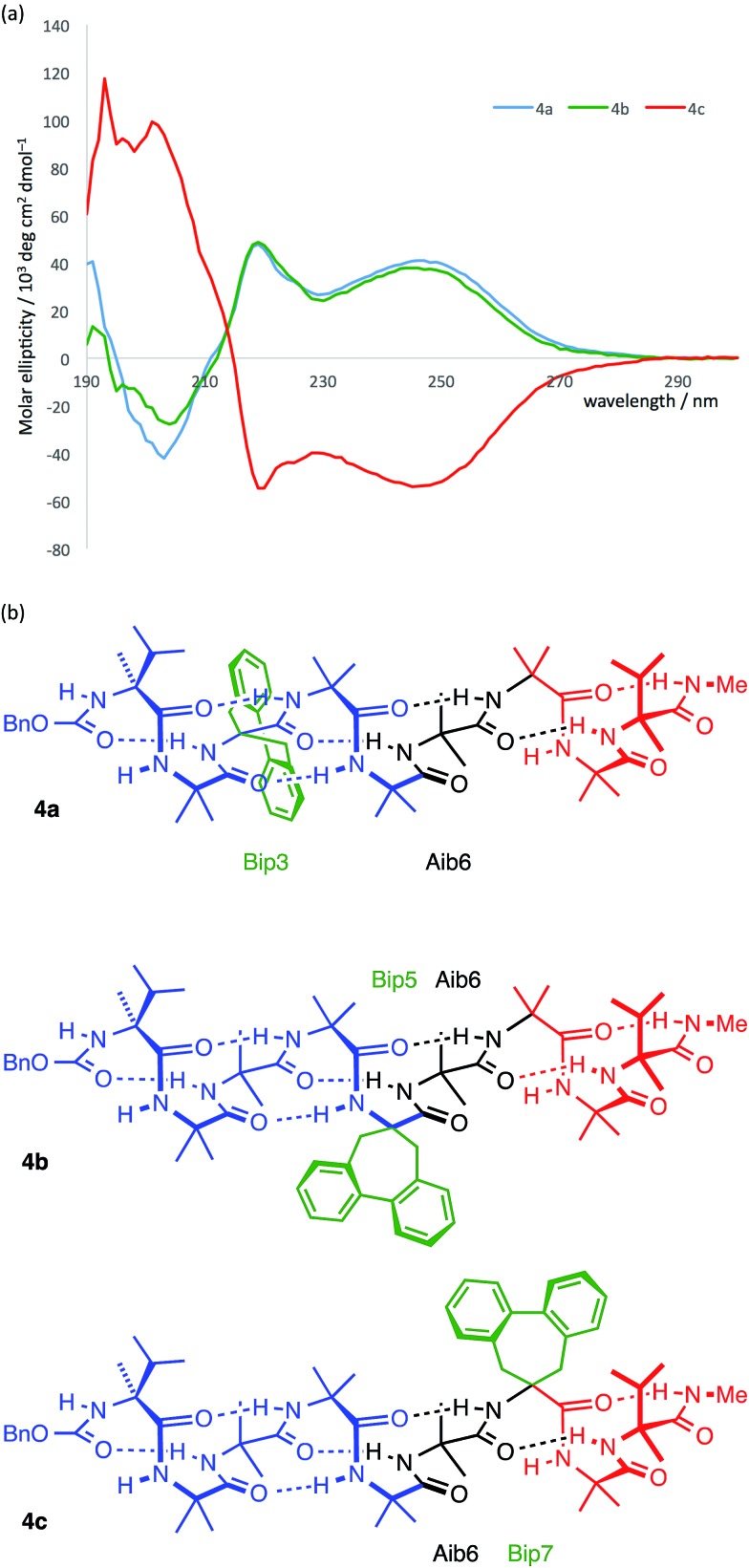
(a) CD spectra of compounds **4a–c** indicating that the Bip residue is in a *P* environment in **4a** and **4b** and in an *M* environment in **4c**; (b) presumed principal conformation of the three isomers, with the screw-sense reversal located at Aib6. Blue indicates right-handed screw sense; red indicates left-handed screw sense.

The CD spectra of **4a–c** are shown in Fig. 9a. In each case a Cotton effect is evident, due to the Bip residue, with a positive or negative maximum at 247 nm.^68–70^ For the two compounds **4a** and **4c** in which the Bip residue is located close to the terminal controllers, the sign of the Cotton effect is consistent with the screw sense induced by the nearby controller, as expected, and both Cotton effects have more or less equal and opposite molar ellipticity. The CD spectrum of the oligomer **4b** indicates that the screw sense at the location of the Bip residue, which lies in the middle of the chain, is right-handed. This suggests, that in these compounds, the screw-sense reversal lies between residue 5 and 7 of the oligomer, a result consistent with evidence from X-ray crystallography, NMR and computation that the screw-sense reversal of **1b** lies closer to the C-terminus than to the N-terminus of the achiral central domain.

Curiously, the degree of screw-sense induction does not decrease when the Bip residue is moved from position 3 to position 5, which suggests that in **4b** the screw-sense reversal never lies to the N-terminal side of residue 5. This seemed surprising, but the bulky Bip residue is manifestly not a ‘silent’ local reporter of screw-sense preference, and could itself have a role to play in amplifying the screw-sense preference. We therefore turned to less intrusive NMR methods and to the analysis of chemical shift differences between ^13^C signals of enantioselectively isotopically enriched Aib residues in order to quantify changes in screw-sense preference along the chain.

**Fig. 10:**
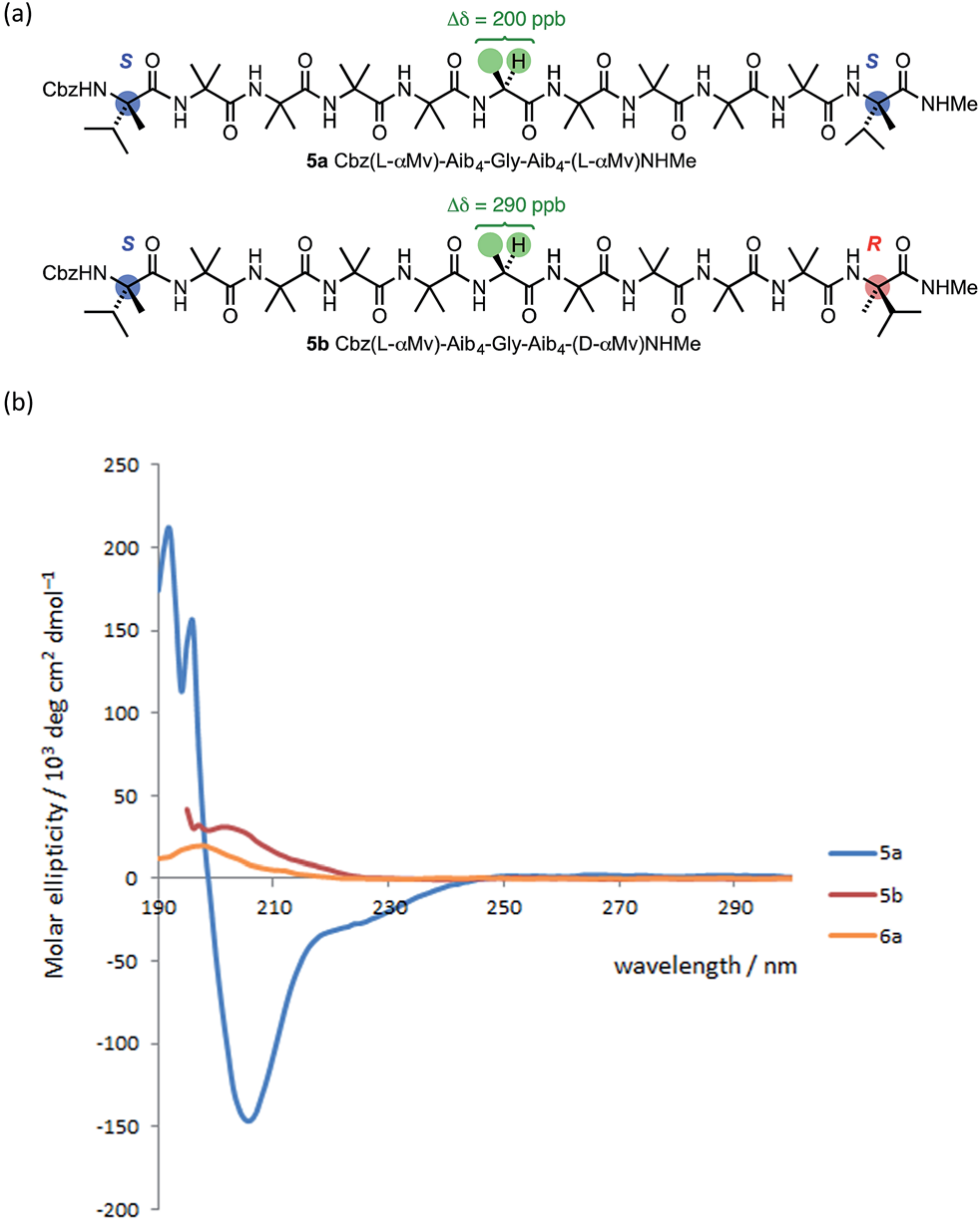
(a) Gly-containing compounds **5**; (b) CD spectra of **5a**, **5b** and **6a** recorded in MeOH at 20 °C.

The oligomer **5a**, terminated at both ends by matched l-(α-MeVal) residues and carrying a Gly residue at the mid-point of the chain,^71^ showed a lower degree of anisochronicity in the ^1^H NMR signals of this Gly residue (Δ*δ* = 200 ppb) than its ‘mismatched’ diastereoisomer **5b** (Δ*δ* = 290 ppb). The conformational flexibility associated with a Gly residue,^53,56^ along with its tendency to loosen a 3_10_ helix towards an α-helix structure,^60^ makes it difficult to draw quantitative conclusions from these figures. The CD spectra of **5a** and **5b** (Fig. 10) suggest that **5a** does indeed display more right-handed α-helical character than the all-Aib chain (the band at 220 nm is negative rather than positive as in **1a**) and that **5b** contains essentially equal amounts of left- and right-handed helical structures (its CD spectrum is much weaker than **5a**).

Raman spectroscopy of **5a** and **5b** showed in both cases a strong band centred at 1662 cm^–1^ (ESI, Fig. S2†). Band deconvolution on the amide 1 region showed two chief contributors to this band, at 1661 cm^–1^ and 1682 cm^–1^, in a 19 : 1 ratio for **5a** and a 9 : 1 ratio for **5b**. As found for **1a** and **1b**, these foldamers principally adopt 3_10_ helical conformations, either in single or multiple domains. However the small contribution at 1682 cm^–1^ would be consistent with small regions adopting non-helical conformations. (The dimer N_3_Aib_2_O^*t*^Bu, for example, which is too short to fold into a helix, shows only a single amide 1 band at 1681 cm^–1^ after deconvolution [ESI, Fig. S3†].) This contributor at 1682 cm^–1^ is more pronounced for the screw-sense mismatched foldamer **5b**, consistent with a non-helical region possibly localized around the Gly residue.

**Fig. 11:**
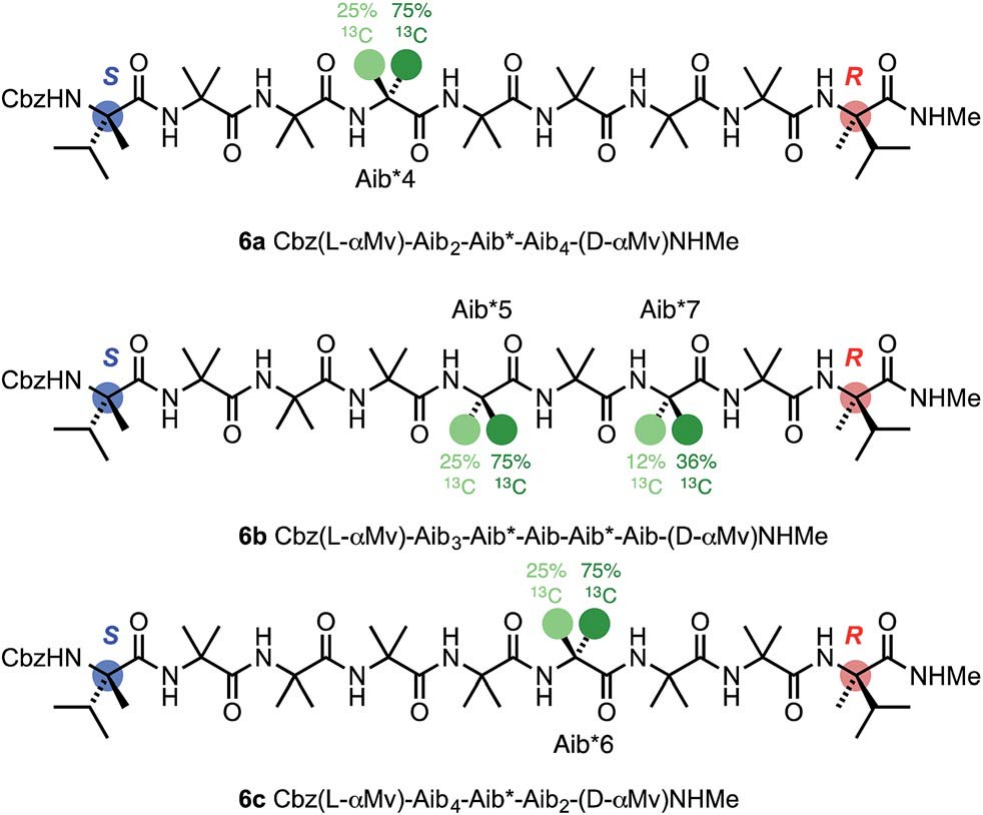
Oligomers containing ^13^C-labelled Aib residues enantioselectively isotopically enriched in their pro-*R* : pro-*S* methyl groups in a 3 : 1 ratio. The isotopic enrichment of residue 7 of **6b** is half that of residue 5, to aid identification.

To allow confidence in assigning absolute screw-sense preference at each point along the chain, compounds **6a–c** were made, in which individual Aib residues were labelled with enantioselective ^13^C enrichment in one of their two Me groups (Fig. 11). To minimize the number of compounds needed, previous indications that the reversal was likely to reside between the Aib4 and Aib7 residues of the Aib heptamer fragment were used to narrow the search window to this area of the molecule. Oligomers **6a**, **6b** and **6c** were made with (*R*)-mono-^13^CH_3_-Aib (Aib*, *ca.* 75 : 25 e.r.^63^) residues incorporated at positions 4, 5 and 6 respectively, with **6b** additionally containing a second ^13^C-labelled Aib residue at position 7, and diluted to 50% total ^13^C abundance to allow the two labelled residues to be distinguished.^22^ The Aib* residues were introduced by means of labelled 2-azidoisobutyric acid derivatives, made by treatment of monolabelled Aib* with trifluoromethylsulfonyl azide.^72,73^ The CD spectrum of **6a** (Fig. 10), like that of **5b**, showed no strong preference for either screw sense, as expected for a structure adopting an *M* screw-sense at the N-terminus and a *P* screw-sense at the C-terminus.

**Fig. 12:**
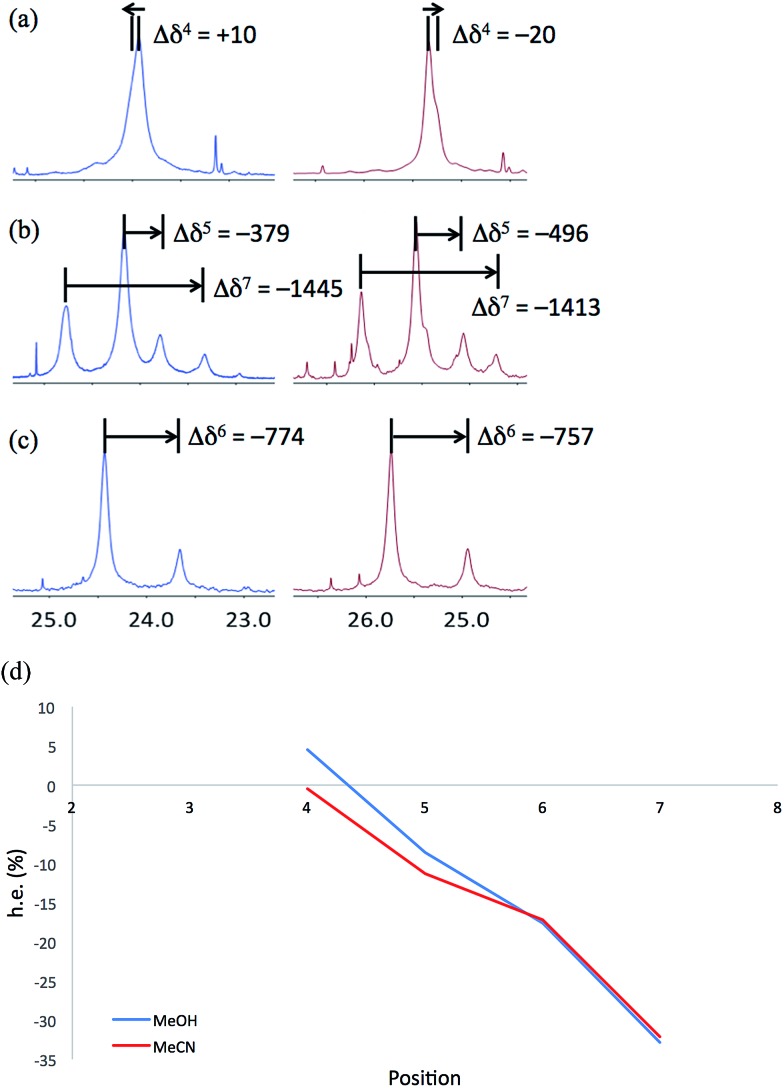
Portions of the ^13^C NMR spectra recorded at 23 °C in MeOH (blue, left) and MeCN (red, right), with Δ*δ* values in ppb, of (a) **6a**, (b) **6b** and (c) **6c**. (d) Plot of h.e., calculated from chemical shift difference Δ*δ* in the ^13^C NMR spectrum recorded in methanol (blue) and acetonitrile (red), against the position in the chain, showing a change from an *M* to a *P* screw-sense at around Aib4. (Negative h.e. indicates an *M* screw-sense.)

NMR spectra of **6a–6c** were run in MeOH and in MeCN and the results (shown in Fig. 12) are broadly comparable. The absolute screw sense is low at all four positions, with values of Δ*δ* all less than 1500 ppb, corresponding to *ca.* 30% h.e. *M* screw sense (indicated by a downfield major signal, and represented here as a negative value for both Δ*δ* and h.e.) dominating from the C-terminus to residue 5, with the decrease in chemical shift separation from Aib7 to Aib5 indicating a reduction in average screw-sense preference further from the C-terminus. By Aib4, the screw sense is essentially zero, with the spectrum in MeOH suggesting a *P* screw-sense is just beginning to dominate, while those in MeCN suggest that an *M* screw-sense persists even as far as Aib4.

These data are consistent with our general interpretation of the experimental and computational data shown in Fig. 5: these show a gradual, rather than sudden, change in screw sense along the achiral domain of **1** and **6**, with a reversal in the average screw-sense preference – and thus the average location of the screw-sense reversal – occurring somewhere close to the centre of the achiral domain, around Aib4 (in **6**) or Aib6 (in **1**).

## Summary and conclusions

We studied a group of substrates of the general structure Cbz-Paa1-Xaa2-Aib3-Aib4-Aib6-Aib7-Yaa8-Maa9-NHMe, in which Paa and Maa are residues that induce preferred *P* and *M* screw-sense preferences respectively, and Xaa and Yaa may be either Aib or additional residues that reinforce those preferences. Using a variety of techniques, we located and characterized, in the solid state, in solution, and computationally, the reversal of screw sense that must occur in these terminally mismatched helical oligomers. The crystal structure of **1b** suggests that the screw-sense reversal may take the form of an antisymmetric tendril perversion, accompanied by the loss of a single hydrogen bond. Computational studies in implicit solvent are consistent with this view of the screw-sense inversion in the absence of solvent. The minimum energy structures calculated for **1b** are close to that observed crystallographically, with a reversal of screw sense located at Aib7.

Studies using NMR spectroscopy allowed us not only to locate the average position of the screw-sense reversal, but also to assess its mobility and preferred habits. In compound **1b**, the reversal happens on average between Aib6 and Aib7, but the less than quantitative preference for a *P* screw-sense at residues 3–6 suggests that the screw-sense inversion spends some of its time close to the N-terminus. The unchanging value of h.e. between residues 3 and 6 indicates that the reversal is not commonly found in this part of the structure.

Computational studies of **1b** in explicit methanol support this interpretation. The most populated clusters of structures show screw-sense reversals at Aib6 or Aib7, consistent with the NMR data. A less populated, but still accessible, cluster shows a left-handed helical preference stretching as far as the N-terminus, also consistent with the NMR data. Calculation of the relative energies of the *M* and *P* screw-senses at each point in the chain show a steady decrease in energetic preference for a *P* screw-sense from Aib3 to Aib4, switching to a preference for an *M* screw-sense at Aib5. These screw-sense preferences show a good match to the solution state values. Computed structures in explicit methanol also suggest that screw-sense reversals are associated with γ-turns, and a γ-turn offers a relatively stable solution-state alternative to the non-hydrogen bonded structure adopted by the screw-sense reversal in the crystalline state.

The less powerfully induced terminal screw-sense preference of **6** results in an even more gradual variation of screw-sense preference between Aib3 and Aib7. NMR spectroscopy shows that the screw-sense reversal in **6** lies on average at Aib4, but that there is a smooth and gradual increase in preference for an *M* screw-sense between this point and the C-terminus, indicating that the screw-sense reversal is significantly more mobile in **6** than in **1**, spending some of its time at most positions in the chain. The mobility of the reversal must be rapid on the NMR time scale, with migration of a screw-sense reversal from one end of a helical oligomer to the other providing a plausible mechanism by which left- and right-handed screw senses interconvert with one another on this rapid time scale.

Our strategy for exploring the helical reversal using mismatched induction of screw sense has potential for applicability across a wide range of helical polymers. Foldamer chemistry has classically sought to build conformationally well defined structures, but the enforced induction of this conformationally mobile feature makes possible the planned incorporation of well-defined, localised conformational flexibility into artificial structures. Evaluation of the relative population of rapidly interconverting conformers is made possible by reducing structural complexity to the point where quantified NMR analysis is facilitated. The simplification of these structures in order to reveal the fine details of conformation and to open up possibilities for using fine-grained control over the dynamics of biomimetic molecules is a concept ripe for further application.
