# Supplementary material for: A tendril perversion in a helical oligomer: trapping and characterizing a mobile screw-sense reversal
Source: Chem Sci. 2017 Jan 25;8(4):3007–18. doi: 10.1039/c6sc05474a (PMC5380885; doi:10.1039/c6sc05474a)
Supplement: Supplementary file 1 [file SC-008-C6SC05474A-s001.pdf]

Electronic Supplementary Material (ESI) for Chemical Science.
This journal is © The Royal Society of Chemistry 2017

A tendril perversion in a helical oligomer: trapping and characterizing a mobile screw-sense reversal

Michael Tomsett[a], Irene Maffucci[b], Bryden A. F. Le Bailly[a], Liam Byrne[c], Stefan M. Bijvoets[c], M. Giovanna Lizio[c], James Raftery[c], Craig P. Butts[a], Simon J. Webb[c], Alessandro Contini[b], Jonathan Clayden[a]*

[a]School of Chemistry, University of Bristol, Cantock's Close, Bristol BS8 1TS, UK. j.clayden@bristol.ac.uk; [b]Dipartimento di Scienze Farmaceutiche Sezione di Chimica Generale e Organica "Alessandro Marchesini", Università degli Studi di Milano, Via Venezian, 21 20133 Milano, Italy; [c]School of Chemistry, University of Manchester, Oxford Road, Manchester M13 9PL, UK.

SUPPORTING INFORMATION

Contents:

1. Experimental procedures 2-37
2. NMR spectra 38-77
3. Computational methods 78-81
4. Raman spectroscopy 82-83
5. X-ray crystallography 83
6. References 84

1. Synthetic procedures

General Procedure A: Oxalyl chloride mediated acid chloride formation and peptide formation

To a stirred solution of acid (1 eq.) and DMF (0.1 mL) in DCM (2 mL/mmol) at 0 °C was added oxalyl chloride (1.1 eq) dropwise. The reaction mixture was allowed to return to rt and stirred for 2 h. The excess solvent and oxalyl chloride were removed *in vacuo.* A solution of amine (0.8 eq.) and Et_3N (1.5 eq.) in DCM (2 mL/mmol) was added dropwise to the 2-azido-2-methylpropanoyl chloride residue at 0 °C and the reaction stirred for a further 16 h at rt. The solvent was removed *in vacuo* and the residue taken up in EtOAc (2 mL/mmol) and washed with 5% aq. $KHSO_4$ (2 ml/mmol) and sat. aq. $NaHCO_3$ (2 mL/mmol). The organic layer was dried ($MgSO_4$), and the solvent evaporated to give the crude product.

General Procedure B: Pd/C catalysed hydrogenation

To a stirred solution of compound (1 eq.) in MeOH (1 mL/mmol) was added Pd/C (~0.1 mg/mg) as a slurry in MeOH/DCM. The reaction was stirred under an atmosphere of H2 until judged complete by TLC. The reaction mixture was then filtered through Celite® and the solvent evaporated to give the crude amine.

General Procedure C: TFFH mediated acid fluoride formation and peptide formation

To a stirred solution of Cbz-αMv-OH (2 eq.) and pyridine (2 eq.) in DCM (4 mL/mmol) was added TFFH (3 eq.) and the reaction left to stir for 4 h. The reaction mixture was washed with ice water (4 × 2 mL/mmol), dried ($MgSO_4$) and the solvent evaporated to give Cbz-αMv-F. To a stirred solution of amine (1 eq.) and DIPEA (1 eq.) in DCM (8 mL/mmol) at 0 °C was added Cbz-αMv-F in DCM (1 mL/mmol) dropwise. The reaction was stirred for 5 d at rt. Once complete the solvent was removed *in vacuo* and the residue taken up in EtOAc (2 mL/mmol), washed with 5% aq. $KHSO_4$ (2 mL/mmol), sat. aq. $NaHCO_3$ (2 mL/mmol) and brine (1 mL/mmol), dried ($MgSO_4$) and the solvent evaporated to give the crude product.

General Procedure D: TFA deprotection of *tert*-butyl esters

To a stirred solution of ester (1 eq.) in DCM (3 mL/mmol) at 0 °C was added TFA (30 eq.) dropwise. Reaction was stirred until judged complete by TLC whereupon all solvent was removed *in vacuo* to give the crude product.

General Procedure E: ICBF mediated peptide formation

To a stirred solution of acid (1 eq.) and NMM (1.05 eq.) in THF (3 mL/mmol) at −15 °C was added iBuCOCl (1 eq.) dropwise and the reaction stirred for 15 min. A suspension of amine (2 eq.) and NMM (2 eq.) in THF (5 mL/mmol) was added, and the reaction brought to rt and stirred for 16 h. The solvent

was removed *in vacuo* and the residue taken up in EtOAc (15 mL/mmol) and washed with 5% aq. $KHSO_4$ (15 mL/mmol), sat. aq. $NaHCO_3$ (15 mL/mmol) and brine (15 mL/mmol). The organic phase was dried ($MgSO_4$) and the solvent evaporated to give the crude product.

General Procedure F: Peptide coupling through azlactone formation

To a stirred solution of acid (1 eq.) and EDC.HCl (1.5 eq.) in DCM (5 mL/mmol) was added DIPEA (1.5 eq.) and the reaction stirred for 4 h. Reaction solvent was removed *in vacuo* and the residue taken up in EtOAc (5 mL/mmol. The organic layer was washed with 5% aq. $KHSO_4$ (2 × 3 mL/mmol) and brine (3 mL/mmol), dried ($MgSO_4$) and the solvent removed in vacuo to give the azlactone. A solution of azlactone in MeCN (5 mL/mmol) was added to amine (1 eq.) and the reaction stirred at reflux for at least 5 d. The reaction mixture was then allowed to cool, the solvent removed *in vacuo* and the residue taken up in EtOAc (10 mL/mmol). The organic layer was washed with 5% aq. $KHSO_4$ (10 mL/mmol), sat. aq. $NaHCO_3$ (10 mL/mmol) and brine (5 mL/mmol), dried ($MgSO_4$) and the solvent evaporated to give the crude product.

Cbz-L/D-αMv-OH – S1

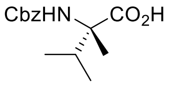

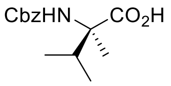

To a stirred solution of H-L/D-αMv-OH (1.00 g, 7.63 mmol) in acetone (17.5 mL) and 2 M aq. NaOH (17.5 mL) at 0 °C was added benzyl chloroformate (0.65 mL, 4.58 mmol) in acetone (2 mL) dropwise over 30 min. The reaction was stirred for 6 h before benzyl chloroformate was added again in the same manner. The pH was adjusted to 13 with 2 M aq. NaOH and left to stir overnight. The acetone was removed *in vacuo* and the aqueous diluted with 2 M aq. NaOH (10 mL). This was washed with Et_2O (3 × 20 mL), and then acidified to pH 2 with conc. HCl. The aqueous was then extracted with EtOAc (3 × 30 mL), and the organic washed with brine (30 mL), dried ($MgSO_4$) and evaporated to give **S1** as a thick oil which crystallises over time (1.24 g, 4.67 mmol, 61%). δ_H (500 MHz, $CDCl_3$) 7.31-7.21 (5H, m, Ar*H*), 5.29 (1H, s, br, N*H*), 4.14 (1H, d, *J* = 7.2, PhCH_2), 4.11 (1H, d, *J* = 7.2, PhCH_2), 2.30-2.14 (1H, m, C*H*(CH_3)$_2$), 1.50 (3H, s, NHC(CH_3)), 0.92 (3H, d, *J* = 6.9, CH(CH_3)$_2$), 0.89 (3H, d, *J* = 6.9, CH(CH_3)$_2$); δ_C (125 MHz, $CDCl_3$) 178.4 (*C*O_2H), 155.6 (*C*ONH), 136.2, 128.6, 128.2, 128.1 (Ar), 66.9 (NH*C*(CH_3)), 63.0 (Ph*C*H_2), 34.7 (*C*H(CH_3)$_2$), 18.8 (NHC(*C*H_3)), 17.3 (CH(*C*H_3)$_2$), 17.1 (CH(*C*H_3)$_2$); (**L**) **$[\alpha]_D^{20}$** = +16.0 (*c* 0.33, CH_3OH), (**D**) **$[\alpha]_D^{20}$** = −16.2 (*c* 1.00; CH_3OH) (Lit = ± 16.3, *c* 0.80). Data are in accordance with literature values.[1]

Cbz-L/D-αMv-NHMe – S2

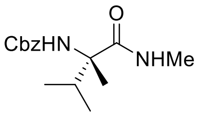

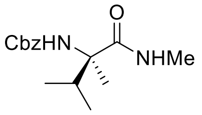

According to the procedure of Toniolo:[2] To a stirred solution of **S1** (1.23 g, 4.64 mmol), HOBt (1.03 g, 6.96 mmol) and DIPEA (1.20 mL, 6.95 mmol) in DCM (15 mL) was added EDC.HCl (133 g, 6.96 mmol) and left to stir for 1 h. $MeNH_2$.HCl (938 mg, 13.9 mmol) was added with DIPEA (3.25 mL, 13.9 mmol) and the reaction was stirred for 16 h. When complete the reaction mixture was taken up in EtOAc (60 mL), and washed with 5% aq. $KHSO_4$ (40 mL), sat. aq. $NaHCO_3$ (40 mL) and brine (20 mL). The organic layer was dried ($MgSO_4$) and the solvent evaporated to give the crude product. This was purified by flash column chromatography (1:1 PE:EtOAc increasing to 1:4) to give pure **S2** as a clear oil (993 mg, 3.57 mmol, 77%). **δ_H** (500 MHz, $CDCl_3$) 7.23-7.14 (5H, m, Ar*H*), 6.82 (1H, s, br, N*H*), 5.69 (1H, s, N*H*), 4.97 (1H, d, *J* = 12.3, PhCH_2), 4.91 (1H, d, *J* = 12.3, PhCH_2), 2.62 (3H, s, NHCH_3), 2.26-2.08 (1H, m, C*H*(CH_3)$_2$), 1.37 (3H, s, NHC(CH_3)), 0.79 (3H, d, *J* = 6.9, CH(CH_3)$_2$), 0.77 (3H, d, *J* = 6.9, CH(CH_3)$_2$); **δ_C** (125 MHz, $CDCl_3$) 174.3 (*C*ONHCH_3), 155.5 (*C*ONHCH_2), 136.2, 128.5, 128.1, 128.0 (Ar), 66.6 (NH*C*(CH_3)), 63.2 (Ph*C*H_2), 34.5 (*C*H(CH_3)$_2$), 26.5 (NH*C*H_3), 18.0 (NHC(*C*H_3)), 17.1 (CH(*C*H_3)$_2$), 17.0 (CH(*C*H_3)$_2$); (**L**) **$[\alpha]_D^{20}$** = +6.4 (*c* 0.50; CH_3OH), (**D**) **$[\alpha]_D^{20}$** = −6.4 (*c* 1.00; CH_3OH) (Lit = ± 0.6, *c* 0.50). Data are in accordance with literature values.

H-L/D-αMv-NHMe – S3

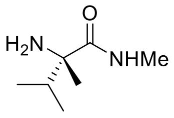

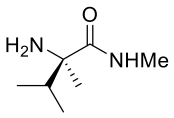

From **S2** (1.18 g, 3.96 mmol) according to **General Procedure B**. **S3** obtained as white powder (553 mg, 3.84 mmol, 97%). **R_f** 0.24 (EtOAc); **m.p.** >235 °C (decomp); **δ_H** (500 MHz, CD_3OD), 4.85 (2H, s, br, NH_2), 2.74 (3H, s, NHCH_3), 2.03 (1H, sep, *J* = 6.9, C*H*(CH_3)$_2$), 1.29 (3H, s, NHC(CH_3)), 0.90 (3H, d, *J* = 6.9, CH(CH_3)$_2$), 0.81 (3H, d, *J* = 6.9, CH(CH_3)$_2$); **δ_C** (125 MHz, CD_3OD) 180.6 (*C*ONHCH_3), 61.4 (NH*C*(CH_3)), 36.4 (*C*H(CH_3)$_2$), 26.3 (NH*C*H_3), 24.8 (NHC(*C*H_3)), 17.7 (CH(*C*H_3)$_2$), 16.4 (CH(*C*H_3)$_2$); **v_{max}/cm^{-1}** 3264m.br (N-H), 2971m (C-H), 1661s (C=O), 1554m (C-N), 1515m (C-N); (**L**) **$[\alpha]_D^{20}$** = −24.0 (*c* 1.04, CH_3OH), (**D**) **$[\alpha]_D^{20}$** = +24.0 (*c* 1.07, CH_3OH).

Cbz-L/D-αMv$_2$-NHMe – S4

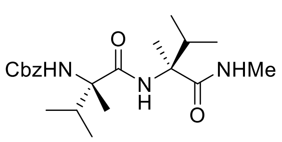

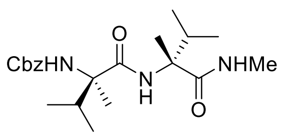

From **S1** (1.86 g, 7.68 mmol), TFFH (3.04 g, 11.5 mmol), pyridine (0.56 mL, 7.68 mmol), **S3** (553 mg, 3.84 mmol) and DIPEA (0.67 mL, 3.84 mmol) according to **General Procedure C**. Crude **S4** was purified by flash column chromatography (99:1 DCM:MeOH increasing to 19:1) to give the pure product (412 mg, 1.05 mmol, 27%). **R_f** 0.20 (1:1 PE:EtOAc); **m.p.** 105-108 °C; **δ_H** (500 MHz, $CDCl_3$) 7.36-7.30 (5H, m, Ar*H*), 7.24 (1H, s, br, CON*H*), 6.22 (1H, s, br, CON*H*), 5.21 (1H, s, PhCH_2), 5.19 (1H, s, PhCH_2), 3.85 (1H, s, br, CON*H*), 2.77 (3H, d, *J* = 4.5,

NHCH_3), 1.97-1.87 (2H, m, CH(CH_3)$_2$), 1.53 (3H, s, NHC(CH_3)), 1.43 (3H, s, NHC(CH_3)), 0.96 (3H, d, *J* = 6.9, CH(CH_3)$_2$), 0.93 (3H, d, *J* = 6.9, CH(CH_3)$_2$), 0.82 (3H, d, *J* = 6.9, CH(CH_3)$_2$), 0.66 (3H, d, *J* = 6.9, CH(CH_3)$_2$); **δ_C** (125 MHz, $CDCl_3$) 173.9 (*C*ONH), 171.6 (*C*ONH), 156.2 (*C*O_2Bn), 135.7, 128.7, 128.7, 128.6 (Ar), 67.5 (PhCH_2), 63.8 (NH*C*CO), 63.0 (NH*C*CO), 35.9 (*C*H(CH_3)$_2$), 35.8 (*C*H(CH_3)$_2$), 26.4 (NHCH_3), 19.6 (NHC(CH_3)), 17.8 (NHC(CH_3)), 17.3 (CH(CH_3)$_2$), 17.2 (CH(CH_3)$_2$), 17.1 (CH(CH_3)$_2$), 16.6 (CH(CH_3)$_2$); **ν_{max}/cm^{-1}** 3280m.br (N-H), 2965m (C-H), 1711s (C=O), 1668s (C=O), 1531m (C-N), 1497m (C-N); ***m/z*** (ESI$^+$) 392.60 ([M+H]$^+$, 100%), 414.58 ([M+Na]$^+$, 70%), HRMS (ESI$^+$) found [M+Na]$^+$ 414.2361 [$C_{21}H_{33}N_3O_4Na$]$^+$ requires 414.2369; (**L**) **[α]$_D^{20}$** = +35.6 (*c* 0.91, CH_3OH), (**D**) **[α]$_D^{20}$** = −35.5 (*c* 1.00; CH_3OH).

H-L/D-αMv$_2$-NHMe – S5

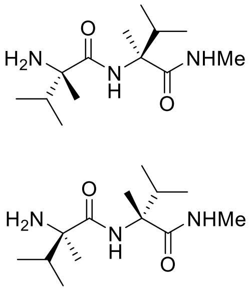

From **S4** (348 mg, 0.93 mmol) according to **General Procedure B**. **S5** was obtained as a clear oil (319 mg, *quant.*). **R$_f$** 0.43 (19:1 DCM:MeOH); **δ_H** (500 MHz, CD_3OD) 2.76 (3H, s, *J* = 4.5, NHCH_3), 2.30 (1H, sep, *J* = 6.9, CH(CH_3)$_2$), 2.22 (1H, sep, *J* = 6.9, CH(CH_3)$_2$), 1.57 (3H, s, NHC(CH_3)), 1.55 (3H, s, NHC(CH_3)), 1.08 (3H, d, *J* = 6.9, CH(CH_3)$_2$), 1.04 (3H, d, *J* = 6.9, CH(CH_3)$_2$), 1.00 (3H, d, *J* = 6.9, CH(CH_3)$_2$), 0.92 (3H, d, *J* = 6.9, CH(CH_3)$_2$); **δ_C** (125 MHz, $CDCl_3$) 173.7 (*C*ONH), 169.1 (*C*ONH), 64.6 (NH*C*CO), 64.1 (NH*C*CO), 35.1 (*C*H(CH_3)$_2$), 34.7 (*C*H(CH_3)$_2$), 30.1 (NHCH_3), 26.7 (NHC(CH_3)), 18.6 (NHC(CH_3)), 17.6 (CH(CH_3)$_2$), 17.5 (CH(CH_3)$_2$), 17.0 (CH(CH_3)$_2$), 16.9 (CH(CH_3)$_2$); **ν_{max}/cm^{-1}** 3362m.br (N-H), 1658s (C=O), 1494m (C-N); (**L**) **[α]$_D^{20}$** = +18.4 (*c* 1.00; CH_3OH), (**D**) **[α]$_D^{20}$** = −18.4 (*c* 1.01, CH_3OH).

2-Azido-2-methylpropanoic acid – S6

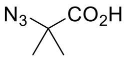

To a stirred solution of 2-bromo-2-methylpropanoic acid (15.0 g, 89.9 mmol) in DMF (80 mL) was added NaN_3 (8.80 g, 135 mmol). The reaction was stirred for 72 h, and then diluted with water (30 mL) and acidified to pH 2 with 1 M aq. HCl (40 mL). This was extracted with TBME (2 × 75 mL) and the organic layer washed with 1 M aq. HCl (4 × 20 mL). The organic phase was dried ($MgSO_4$) and concentrated *in vacuo* to give **S6** as a yellow oil (9.90 g, 76.6 mmol, 85%). **δ_H** (500 MHz, $CDCl_3$) 11.54 (1H, s, br, CO_2H), 1.52 (6H, s, C(CH_3)$_2$); **δ_C** (125 MHz, $CDCl_3$) 179.6 (*C*O_2H), 63.0 (*C*(CH_3)$_2$), 24.4 (C(CH_3)$_2$). Data are in accordance with literature values.[3]

N$_3$-Aib-L/D-αMv$_2$-NHMe – S7

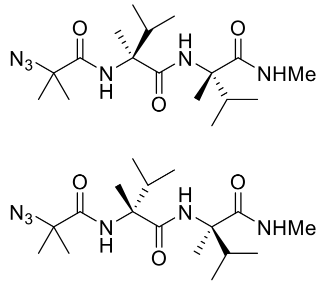

From **S6** (150 mg, 1.17 mmol), oxalyl chloride (0.10 mL, 1.28 mmol), **S5** (150 mg, 0.58 mmol) and Et_3N (0.16 mL, 1.17 mmol) according to **General Procedure A**. Crude **S7** was purified by flash column chromatography (5:1 PE:EtOAc, increasing EtOAc) to the pure product as white crystals (123 mg, 0.33 mmol, 58%). **R_f** 0.30 (5:1 PE:EtOAc); **m.p.** 74-76 °C; **δ_H** (500 MHz, $CDCl_3$) 7.12 (1H, s, br, N*H*CH_3), 6.93 (1H, s, CON*H*), 6.16 (1H, s, CON*H*), 2.74 (3H, d, *J* = 4.7, NHC*H*$_3$), 2.07 (1H, sep, *J* = 7.0, C*H*$(CH_3)_2$), 2.04 (1H, sep, *J* = 7.0, C*H*$(CH_3)_2$), 1.51 (3H, s, NHC(C*H*$_3$)), 1.50 (3H, s, N_3C(C*H*$_3$)$_2$), 1.49 (3H, s, N_3C(C*H*$_3$)$_2$), 1.34 (3H, s, NHC(C*H*$_3$)), 0.94 (3H, d, *J* = 7.0, CH(C*H*$_3$)$_2$), 0.90 (3H, d, *J* = 7.0, CH(C*H*$_3$)$_2$), 0.84 (3H, d, *J* = 7.0, CH(C*H*$_3$)$_2$), 0.77 (3H, d, *J* = 7.0, CH(C*H*$_3$)$_2$); **δ_C** (125 MHz, $CDCl_3$) 173.7 (*C*ONH), 173.1 (*C*ONH), 171.1 (*C*ONH), 64.5 (N_3*C*), 63.8 (NH*C*CO), 63.3 (NH*C*CO), 35.6 (*C*H$(CH_3)_2$), 35.3 (*C*H$(CH_3)_2$), 26.3 (NH*C*H$_3$), 24.3 (NHC(*C*H$_3$)) 17.3 (CH(*C*H$_3$)$_2$), 17.1 (CH(*C*H$_3$)$_2$); **ν_{max}/cm^{-1}** 3337m.br (N-H), 2968m (C-H), 2113s (N_3), 1683m (C=O), 1648s (C=O), 1494s (C-N) 1457m (C-N); ***m/z*** (ESI$^+$) 269.4 ($[M+H]^+$, 90%), 391.4 ($[M+Na]^+$, 100%), HRMS (ESI$^+$) found $[M+Na]^+$ 391.2431 $[C_{17}H_{32}N_6O_3Na]^+$ requires 391.2428; (**L**) **$[\alpha]_D^{20}$** = +69.7 (*c* 0.33, CH_3OH), (**D**) **$[\alpha]_D^{20}$** = −69.5 (*c* 0.50; CH_3OH).

H-L/D-αMv$_2$-NHMe – S8

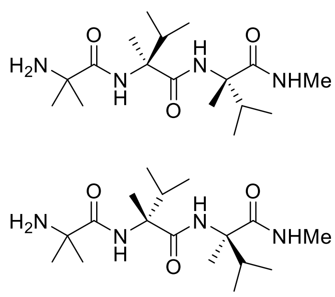

From **S7** (348 mg, 0.93 mmol) according to **General Procedure B**. **S8** was obtained as a clear oil (319 mg, *quant.*). **R_f** 0.69 (19:1 DCM:MeOH); **δ_H** (500 MHz, CD_3OD) 2.76 (3H, s, *J* = 4.5, NHC*H*$_3$), 2.30 (1H, sep, *J* = 6.9, C*H*$(CH_3)_2$), 2.22 (1H, sep, *J* = 6.9, C*H*$(CH_3)_2$), 1.57 (3H, s, NHC(C*H*$_3$)), 1.55 (3H, s, NHC(C*H*$_3$)), 1.08 (3H, d, *J* = 6.9, CH(C*H*$_3$)$_2$), 1.04 (3H, d, *J* = 6.9, CH(C*H*$_3$)$_2$), 1.00 (3H, d, *J* = 6.9, CH(C*H*$_3$)$_2$), 0.92 (3H, d, *J* = 6.9, CH(C*H*$_3$)$_2$); **δ_C** (125 MHz, $CDCl_3$) 173.7 (*C*ONH), 169.1 (*C*ONH), 64.6 (NH*C*CO), 64.1 (NH*C*CO), 35.1 (*C*H$(CH_3)_2$), 34.7 (*C*H$(CH_3)_2$), 30.1 (NH*C*H$_3$), 26.7 (NHC(*C*H$_3$)), 18.6 (NHC(*C*H$_3$)), 17.6 (CH(*C*H$_3$)$_2$), 17.5 (CH(*C*H$_3$)$_2$), 17.0 (CH(*C*H$_3$)$_2$), 16.9 (CH(*C*H$_3$)$_2$); **ν_{max}/cm^{-1}** 3392m.br (N-H), 2968m (C-H), 1617s (C=O), 1463m (C-N), 1403s (C-N); ***m/z*** (ESI$^+$) 243.4 ($[M+H]^+$, 100%), 365.4 ($[M+Na]^+$, 20%), HRMS (ESI$^+$) found $[M+Na]^+$ 365.2528 $[C_{17}H_{34}N_4O_3Na]^+$ requires 365.2529; (**L**) **$[\alpha]_D^{20}$** = +18.4 (*c* 1.00; CH_3OH), (**D**) **$[\alpha]_D^{20}$** = −18.4 (*c* 1.00; CH_3OH).

Cbz-L-αMv$_2$-Aib$_4$-OH – S9

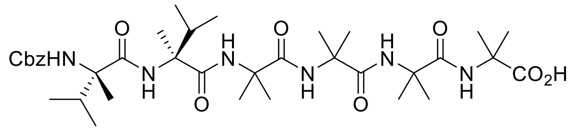

Prepared as previously reported.[4]

Cbz-L-αMv$_2$-Aib$_5$-L-αMv$_2$-NHMe – 1a

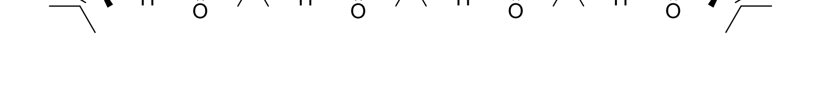

From **S9** (72 mg, 0.10 mmol), EDC.HCl (25 mg, 0.13 mmol), DIPEA (23 µL, 0.13 mmol) and **L-S8** (52 mg, 0.15 mmol) according to **General Procedure F**. Crude **1a** was purified by flash column chromatography (99:1 DCM:MeOH increasing to 90:10) to give the pure product as a white powder (64 mg, 0.06 mmol, 61%). **R*f*** 0.22 (19:1 DCM:MeOH); **m.p.** >250 °C; **δ_H** (500 MHz, $CDCl_3$) 7.79 (1H, s, CON*H*), 7.77 (1H, s, CON*H*), 7.72 (1H, s, CON*H*), 7.63 (1H, s, CON*H*), 7.59 (1H, s, CON*H*), 7.38 (5H, m, Ph*H*), 7.33 (2H, s, CON*H*), 7.00 (1H, s, CON*H*), 6.37 (1H, s, CON*H*), 5.21 (1H, s, CON*H*), 5.19 (1H, d, *J* = 12.2, PhCH_2), 5.03 (1H, d, *J* = 12.2, PhCH_2), 2.80 (3H, d, *J* = 4.6, NHCH_3), 2.15 (1H, sep, *J* = 6.7, C*H*(CH_3)$_2$), 2.07 (1H, sep, *J* = 6.8, C*H*(CH_3)$_2$), 1.86 (1H, sep, *J* = 6.7, C*H*(CH_3)$_2$), 1.53 (1H, m, C*H*(CH_3)$_2$), 1.51 (3H, s, NHC(CH_3)$_2$), 1.49 (6H, s, NHC(CH_3)$_2$), 1.49 (3H, s, NHC(CH_3)$_2$), 1.49 (6H, s, NHC(CH_3)$_2$), 1.47 (3H, s, NHC(CH_3)$_2$), 1.47 (3H, s, NHC(CH_3)$_2$), 1.47 (3H, s, NHC(CH_3)$_2$), 1.46 (3H, s, NHC(CH_3)), 1.45 (6H, s, NHC(CH_3)), 1.41 (3H, s, NHC(CH_3)), 1.37 (3H, s, NHC(CH_3)), 1.07 (3H, d, *J* = 6.7, CH(CH_3)$_2$), 1.02 (3H, d, *J* = 6.7, CH(CH_3)$_2$), 1.00 (3H, d, *J* = 6.8, CH(CH_3)$_2$), 0.97 (3H, d, *J* = 6.8, CH(CH_3)$_2$), 0.96 (3H, d, *J* = 7.1, CH(CH_3)$_2$), 0.95 (3H, d, *J* = 7.1, CH(CH_3)$_2$), 0.80 (3H, d, *J* = 6.5, NHC(CH_3)$_2$), 0.79 (3H, d, *J* = 6.4, CH(CH_3)$_2$); **δ_C** (100 MHz, $CDCl_3$) 1794, 176.3, 175.9, 175.6, 175.6, 175.4, 173.4, 173.2, 172.6 (*C*ONH), 156.4 (*C*O_2Bn), 135.8, 128.9, 128.9, 128.7 (Ar), 67.9 (Ph*C*H_2), 63.6, 63.5, 63.3, 62.5 (NH*C*(CH_3)), 57.0, 56.9, 56.8, 56.7 (NH*C*(CH_3)$_2$), 36.2, 35.9, 35.8, 35.8 (*C*H(CH_3)$_2$), 27.7, 27.6, 27.4, 27.4, 27.3 (NHC(*C*H_3)$_2$), 26.5 (NH*C*H_3), 23.0, 23.0, 22.9, 22.8 (NHC(*C*H_3)$_2$), 18.2, 18.1, 18.0, 17.5, 17.5, 17.4, 17.3, 17.2, 17.1, 16.7, 16.2, 16.2 (CH(*C*H_3)$_2$); **ν_{max}/cm^{-1}** 3296m (N-H), 2982m (C-H), 1656s (C=O), 1531m (C-N); ***m/z*** (ESI$^+$) 1065.9 ([M+Na]$^+$, 100%), HRMS (ESI$^+$) found [M+H]$^+$ 1043.6816 [$C_{53}H_{91}N_{10}O_{11}$]$^+$ requires 1043.6869; **[α]$_D^{20}$** = +24.3 (*c* 1.00; (CH_3Cl).

Cbz-L-αMv$_2$-Aib$_5$-D-αMv$_2$-NHMe – 1b

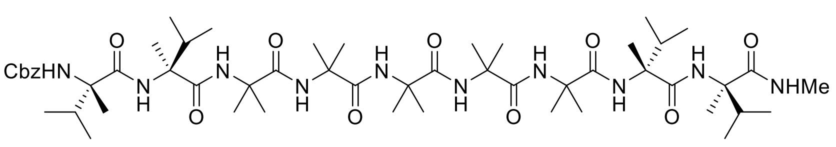

From **S9** (65 mg, 0.09 mmol), EDC.HCl (19 mg, 0.10 mmol), DIPEA (17 µL, 0.10 mmol) and **D-S8** (37 mg, 0.11 mmol) according to **General Procedure F**. Crude **1b** was purified by flash column chromatography (96:4 DCM:MeOH increasing to 94:6) to give the pure product as a white powder (49 mg, 0.05 mmol, 56%). **R*f*** 0.23 (1:1 EtOAc:DCM); **m.p.** 254-256 °C; **δ_H** (400 MHz, $CDCl_3$) 7.77 (1H, s, CON*H*), 7.69 (1H, s, CON*H*), 7.60 (2H, s, CON*H*), 7.68 (1H, s, CON*H*), 7.57 (1H, s, CON*H*), 7.47 (1H, s, CON*H*), 7.36 (5H, s, Ph*H*), 7.20 (1H, s, CON*H*), 6.48 (1H, s, CON*H*), 5.60 (1H, s, CON*H*), 5.17 (1H, d, *J* = 12.1, PhCH_2), 5.03 (1H, d, *J* = 12.1, PhCH_2), 2.81 (3H, d, *J* = 4.5, NHCH_3), 2.51 (1H, sep, *J* = 6.7, C*H*(CH_3)$_2$), 2.09 (1H, sep, *J* = 6.7, C*H*(CH_3)$_2$), 1.91 (1H, m, C*H*(CH_3)$_2$), 1.60 (1H, sep, *J* = 6.8, C*H*(CH_3)$_2$), 1.52 (3H, s, NHC(CH_3)$_2$), 1.50 (6H, s, NHC(CH_3)$_2$), 1.49 (3H, s, NHC(CH_3)$_2$), 1.47 (6H, s, NHC(CH_3)$_2$), 1.47 (3H, s, NHC(CH_3)$_2$), 1.46 (6H, s, NHC(CH_3)$_2$), 1.45 (3H, s, NHC(CH_3)$_2$), 1.44 (3H, s, NHC(CH_3)), 1.43 (3H, s, NHC(CH_3)), 1.39 (3H, s, NHC(CH_3)), 1.38 (3H, s, NHC(CH_3)), 0.98 (3H, d, *J* = 6.7, CH(CH_3)$_2$), 0.97 (3H,

d, *J* = 6.8, CH(C*H*$_3$)$_2$), 0.95 (6H, m, CH(C*H*$_3$)$_2$), 0.95 (3H, d, *J* = 6.7, CH(C*H*$_3$)$_2$), 0.92 (6H, m, CHC(C*H*$_3$)$_2$), 0.80 (6H, d, *J* = 6.7, CH(C*H*$_3$)$_2$); **δ_C** (125 MHz, $CDCl_3$) 176.7, 175.7, 175.4, 175.3, 175.3, 175.1, 173.1, 172.7, 172.5 (*C*ONH), 156.3 (*C*O$_2$Bn), 135.8, 128.7, 128.7, 128.5 (Ar), 67.6 (Ph*C*H$_2$), 64.2, 63.5, 63.2, 62.5 (NH*C*(CH$_3$)), 57.1, 57.0, 56.8, 56.8 (NH*C*(CH$_3$)$_2$), 36.1, 35.8, 35.7, 33.7 (*C*H(CH$_3$)$_2$), 27.0, 26.7, 26.7, 26.6, 25.9 (NHC(*C*H$_3$)$_2$), 24.9 (NH*C*H$_3$), 24.1, 23.7, 23.4, 23.2 (NHC(*C*H$_3$)$_2$), 18.4, 18.1, 18.0, 17.7, 17.7, 17.5, 17.5, 17.4, 17.3, 17.3, 17.2 (CH(*CH$_3$*)$_2$); **v_{max}/cm^{-1}** 3295m (N-H), 2982m (C-H), 1656s (C=O), 1531m (C-N); ***m/z*** (ESI$^+$) 1044.2 ([M+H]$^+$, 100%), 1066.2 ([M+Na]$^+$, 90%), HRMS (ESI$^+$) found [M+H]$^+$ 1043.6869 $[C_{53}H_{91}N_{10}O_{11}]^+$ requires 1043.6869; **$[\alpha]_D^{20}$** = +23.6 (*c* 1.00; (CH_3Cl).

H-Aib$_2$-O^{t}Bu – S10

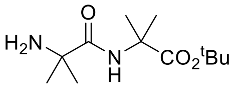

Prepared as previously reported.[5]

Cbz-L-αMv-Aib$_2$-O^{t}Bu – S11

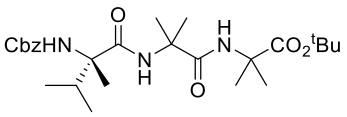

From **L-S1** (941 mg, 3.55 mmol), TFFH (1410 mg, 5.36 mmol), pyridine (0.28 mL, 3.52 mmol), **S10** (461 mg, 1.89 mmol) and DIPEA (0.33 mL. 1.89 mmol) according to **General Procedure C**. Crude **S11** was purified by flash column chromatography (DCM) and trituration (Et_2O) to give the pure product (387 mg, 0.79 mmol, 42%). **R_f** 0.43 (DCM); **δ_H** (500 MHz, $CDCl_3$) 7.38-7.29 (5H, m, Ph*H*), 5.16 (1H, d, *J* = 12.3, PhC*H*$_2$), 5.03 (1H, d, *J* = 12.3, PhC*H*$_2$), 2.04 (1H, sep, *J* = 6.9, C*H*(CH$_3$)$_2$), 1.56-1.40 (12H, m, NHC(C*H*$_3$)$_2$), 1.44 (9H, s, C(C*H*$_3$)$_3$), 1.01 – 0.88 (9H, m, CH(C*H*$_3$)$_2$, NHC(C*H*$_3$)); **δ_C** (125 MHz, $CDCl_3$) 174.2 (*C*ONH), 173.7 (*C*ONH), 172.9 (*C*O$_2$C(CH$_3$)$_3$), 155.7 (*C*O$_2$Bn), 136.1, 128.7, 128.5, 128.2 (Ar), 80.5 (*C*(CH$_3$)$_3$), 72.2 (NH*C*(CH$_3$)), 67.0 (Ph*C*H$_2$), 66.7 (NH*C*(CH$_3$)$_2$), 63.2 (NH*C*(CH$_3$)$_2$), 38.7 (*C*H(CH$_3$)$_2$), 27.9 (C(*C*H$_3$)$_3$), 25.0 (NHC(*C*H$_3$)$_2$), 24.3 (NHC(*C*H$_3$)$_2$), 24.1 (NHC(*C*H$_3$)$_2$), 22.0 (NHC(*C*H$_3$)), 17.2 (CH(*C*H$_3$)$_2$), 17.1 (CH(*C*H$_3$)$_2$); **v_{max}/cm^{-1}** 3350m.br (N-H), 2975m (C-H), 2485s (C-H), 1783s (C=O), 1707s (C=O), 1518s (C-N), 1454m (C-O); ***m/z*** (ESI$^+$) 492.4 ([M+H]$^+$, 100%), 514.4 ([M+Na]$^+$, 99%), HRMS (ESI$^+$) found [M+H]$^+$ 492.3066 $[C_{26}H_{42}N_3O_6]^+$ requires 492.3068; **$[\alpha]_D^{20}$** = +3.2 (*c* 1.25; CH_3OH).

H-L-αMv-Aib$_2$-O^{t}Bu – S12

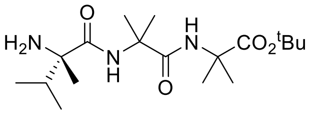

From **S11** (387 mg, 0.94 mmol) according to **General Procedure B**. **S12** obtained as a clear oil (283 mg, 0.80 mmol, 85%). **R_f** 0.00 (EtOAc); **δ_H** (500 MHz, $CDCl_3$) 8.02 (1H, s, br, CON*H*), 7.36 (1H, s, br, CON*H*), 4.21 (1H, s, br, N*H*$_2$), 2.23 (1H, sep, *J* = 6.9, C*H*(CH$_3$)$_2$), 1.51 (6H, s, NHC(C*H*$_3$)$_2$), 1.45 (6H, s, NHC(C*H*$_3$), 1.40

(9H, s, C(C*H*$_3$)$_3$), 1.34 (3H, s, NHC(C*H*$_3$)), 0.91 (3H, d, *J* = 6.9, CH(C*H*$_3$)$_2$), 0.87 (3H, d, *J* = 6.9, CH(C*H*$_3$)$_2$); **δ$_C$** (125 MHz, CDCl$_3$) 176.7 (*C*O$_2$C(CH$_3$)$_3$), 174.3 (*C*ONH), 173.8 (*C*ONH), 80.7 (*C*(CH$_3$)$_3$), 60.4 (NH*C*(CH$_3$)), 56.1 (NH*C*(CH$_3$)$_2$), 34.4 (*C*H(CH$_3$)$_2$), 26.7 (C(*C*H$_3$)$_3$), 23.8 (NHC(*C*H$_3$)$_2$), 23.7 (NHC(*C*H$_3$)$_2$), 23.4 (NHC(*C*H$_3$)$_2$), 23.1 (NHC(*C*H$_3$)), 16.3 (CH(*C*H$_3$)$_2$), 16.2 (CH(*C*H$_3$)$_2$); **ν$_{max}$/cm^{-1}** 3307w.br (N-H), 2973m (C-H), 1731m (C=O), 1650s (C=O), 1507m (C-N); ***m/z*** (ESI$^+$) 358.4 ([M+H]$^+$, 100%), 380.4 ([M+Na]$^+$, 80%), HRMS (ESI$^+$) found [M+H]$^+$ 358.2694 [$C_{18}H_{36}N_3O_4$]$^+$ requires 358.2700; **[α]$_D^{20}$** = −19.6 (*c* 1.00; CH$_3$OH).

Cbz-L-αMv$_2$-Aib$_2$-O^{t}Bu – S13

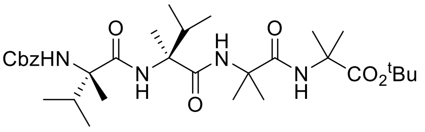

From **L-S1** (387 mg, 1.46 mmol), TFFH (578 mg, 2.19 mmol), pyridine (118 μL, 1.46 mmol), **S12** (178 mg, 0.73 mmol) and DIPEA (130 μL, 0.73 mmol) according to **General Procedure C**. Crude **S13** was purified by flash column chromatography (99:1 DCM:MeOH increasing to 19:1) to give the pure product (190 mg, 0.31 mmol, 43%). **R*$_f$*** 0.30 (98:2 DCM:MeOH); **δ$_H$** (500 MHz, CDCl$_3$) 7.39-7.33 (5H, m, Ph*H*), 7.19 (1H, s, br, CON*H*), 6.32 (1H, s, br, CON*H*), 5.18 (1H, d, *J* = 12.0, PhC*H*$_2$), 5.15 (1H, s, br, CON*H*), 5.00 (1H, d, *J* = 12.0, PhC*H*$_2$), 2.81 (1H, s, CON*H*) 1.88 (2H, m, C*H*(CH$_3$)$_2$), 1.53 (3H, s, NHC(C*H*$_3$)$_2$), 1.49 (3H, s, NHC(C*H*$_3$)$_2$), 1.45 (6H, s, NHC(C*H*$_3$)), 1.43 (12H, s, C(C*H*$_3$)$_3$, NHC(C*H*$_3$)$_2$), 1.40 (3H, s, NHC(C*H*$_3$)$_2$), 0.97 (3H, d, *J* = 6.8, C*H*(CH$_3$)$_2$), 0.96 (3H, d, *J* = 6.9, C*H*(CH$_3$)$_2$), 0.79 (3H, d, *J* = 6.8, C*H*(CH$_3$)$_2$), 0.76 (3H, d, *J* = 6.9, C*H*(CH$_3$)$_2$); **δ$_C$** (125 MHz, CDCl$_3$) 174.0 (*C*ONH), 173.5 (*C*ONH), 171.8 (*C*ONH), 170.9 (*C*O$_2$C(CH$_3$)$_3$), 155.9 (*C*O$_2$Bn), 135.7, 128.7 (Ar), 79.8 (*C*(CH$_3$)$_3$), 67.6 (Ph*C*H$_2$), 63.4 (NH*C*(CH$_3$)$_2$), 62.5 (NH*C*(CH$_3$)$_2$), 56.8 (NH*C*(CH$_3$)$_2$), 56.0 (NH*C*(CH$_3$)$_2$), 36.0 (*C*H(CH$_3$)$_2$), 35.9 (*C*H(CH$_3$)$_2$), 27.9 (C(*C*H$_3$)$_3$), 27.8 (NHC(*C*H$_3$)$_2$), 25.8 (NHC(*C*H$_3$)$_2$), 23.6 (NHC(*C*H$_3$)), 23.1 (NHC(*C*H$_3$)), 18.5 (CH(*C*H$_3$)$_2$), 18.1 (CH(*C*-H$_3$)$_2$), 17.1 (CH(*C*H$_3$)$_2$), 17.0 (CH(*C*H$_3$)$_2$); **ν$_{max}$/cm^{-1}** 3330m.br (N-H), 2975m (C-H), 1783s (C=O), 1706s (C=O), 1668m (C=O), 1517m (C-N); ***m/z*** (ESI$^+$) 606.0 ([M+H]$^+$, 100%), HRMS (ESI$^+$) found [M+H]$^+$ 605.3906 [$C_{32}H_{53}N_4O_7$]$^+$ requires 605.3909; **[α]$_D^{20}$** = +8.7 (*c* 0.97, CH$_3$OH).

Cbz-L-αMv$_2$-Aib$_2$-OH – S14

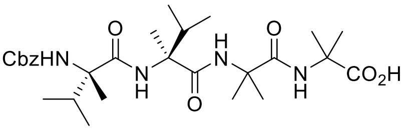

To a stirred solution of **S13** (50 mg, 0.08 mmol) and thioanisole (170 μL, 1.45 mmol) in DCM (0.20 mL) at 0 °C was added TFA (190 μL, 2.50 mmol) dropwise. The reaction was stirred at rt for 4 h before the solvent was removed *in vacuo*. The crude product was purified by column chromatography (99:1

DCM:MeOH increasing to 19:1) to give pure **S14** as a white solid (40 mg, 0.08 mmol, *quant.*). $\mathbf{R_f}$ 0.16 (19:1 DCM:MeOH); **m.p.** 249-251 °C; δ_H (500 MHz, $CDCl_3$) 7.80 (1H, s, CON*H*), 7.61 (1H, s, CON*H*), 7.43-7.30 (5H, m, Ph*H*), 6.98 (1H, s, CON*H*), 5.20 (1H, d, *J* = 12.4, PhCH_2), 5.04 (1H, d, *J* = 12.4, PhCH_2), 1.93 (1H, sep, *J* = 6.8, C*H*$(CH_3)_2$), 1.56 (1H, sep, *J* = 6.8, C*H*$(CH_3)_2$), 1.51 (6H, s, $C(CH_3)_2CO_2H$), 1.46 (3H, s, NHC$(CH_3)_2$), 1.44 (3H, s, NHC$(CH_3)_2$), 1.42 NHC(CH_3)), 1.37 (3H, s, NHC$(CH_3)_2$), 1.00 (3H, d, *J* = 6.8, C*H*$(CH_3)_2$), 0.95 (3H, d, *J* = 6.8, C*H*$(CH_3)_2$), 0.83 (3H, d, *J* = 6.8, C*H*$(CH_3)_2$), 0.80 (3H, d, *J* = 6.8, C*H*$(CH_3)_2$); δ_C (125 MHz, $CDCl_3$) 181.5 (CO_2H), 176.7 (*C*ONH), 175.0 (*C*ONH), 173.7 (*C*ONH), 158.3 (CO_2Bn), 138.3, 129.6, 129.3 (Ar), 68.0 (PhCH_2), 64.5 (NH*C*(CH_3)), 63.9 (NH*C*(CH_3)), 58.1 (NH*C*$(CH_3)_2$), 55.1 (NH*C*$(CH_3)_2$), 37.0 (*C*H$(CH_3)_2$), 36.8 (*C*H$(CH_3)_2$), 28.1 (NHC$(CH_3)_2$), 26.4 (NHC$(CH_3)_2$), 24.3 (NHC(CH_3)), 23.5 (NHC(CH_3)), 18.7 (CH$(CH_3)_2$), 18.4 (CH$(CH_3)_2$), 17.9 (CH$(CH_3)_2$), 17.5 (CH$(CH_3)_2$); $\mathbf{\nu_{max}/cm^{-1}}$ 2980w.br (C-H), 1656s (C=O), 1416s (C-N); ***m/z*** (ESI^-) 547.6 ($[M-H]^-$, 100%), HRMS (ESI^+) found $[M+H]^+$ 549.3277 $[C_{28}H_{45}N_4O_7]^+$ requires 549.3283; $[\alpha]_D^{20}$ = +28.2 (*c* 1.00; CH_3OH).

N_3-Aib$_2$-L/D-αMv$_2$-NHMe – S15

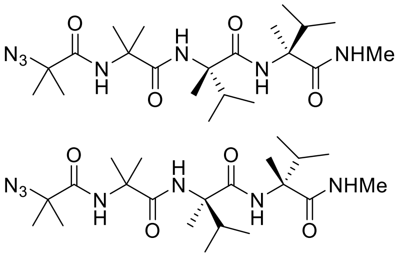

From **S6** (85 mg, 0.66 mmol), oxalyl chloride (62 μL, 0.73 mmol), **S8** (113 mg, 0.33 mmol) and Et_3N (90 μL, 0.66 mmol) according to **General Procedure A**. Crude **S15** was purified by flash column chromatography (99:1 DCM:MeOH increasing to 19:1) to give the pure product as a white solid (97 mg, 0.21 mmol, 65%). $\mathbf{R_f}$ 0.50 (19:1 DCM:MeOH); **m.p.** 156-158 °C; δ_H (400 MHz, $CDCl_3$) 7.20 (1H, d, br, *J* = 4.4, CO_2N*H*CH_3), 6.86 (1H, s, CON*H*), 6.77 (1H, s, CON*H*), 6.43 (1H, s, CON*H*), 2.77 (3H, d, *J* = 4.4, CONHCH_3), 2.02 (1H, sep, *J* = 6.9, C*H*$(CH_3)_2$), 1.93 (1H, sep, *J* = 6.9, C*H*$(CH_3)_2$), 1.53 (3H, s, CONHC$(CH_3)_2$) 1.50 (3H, s, CONHC$(CH_3)_2$), 1.48 (9H, s, $N_3C(CH_3)_2$, CONHC(CH_3), CONHC(CH_3)), 1.38 (3H, s, $N_3C(CH_3)_2$), 0.93 (3H, d, *J* = 6.9, CH$(CH_3)_2$), 0.91 (3H, d, *J* = 6.9, CH$(CH_3)_2$), 0.90 (3H, d, *J* = 6.9, CH$(CH_3)_2$), 0.90 (3H, d, *J* = 6.9, CH$(CH_3)_2$); δ_C (100 MHz, $CDCl_3$) 174.6 (*C*ONH), 173.7 (*C*ONH), 173.0 (*C*ONH), 171.3 (*C*ONH), 64.3 (NH*C*(CH_3), 63.6 (NH*C*(CH_3), 63.1 (CONH*C*$(CH_3)_2$), 57.6 ($N_3C(CH_3)_2$, 36.0 (*C*H$(CH_3)_2$), 35.9 (*C*H$(CH_3)_2$), 26.6 ($N_3C(CH_3)_2$), 26.4 (NHCH_3), 24.5 ($N_3C(CH_3)_2$), 24.5 (NHC$(CH_3)_2$), 23.7 (NHC$(CH_3)_2$), 18.8 (NHC$(CH_3)_2$), 17.7 (NHC(CH_3)), 17.6 (CH$(CH_3)_2$), 17.3 (CH$(CH_3)_2$), 17.2 (CH$(CH_3)_2$), 17.1 (CH$(CH_3)_2$); $\mathbf{\nu_{max}/cm^{-1}}$ 3344m.br (N-H), 2972m (C-H), 2111s (N_3), 1656m (C=O), 1510m (C-N); ***m/z*** (ESI^+) 476.4 ($[M+Na]^+$, 100%), HRMS (ESI^+) found $[M+Na]^+$ 476.2961 $[C_{21}H_{39}N_7O_4Na]^+$ requires 476.2956; (**L**) $[\alpha]_D^{20}$ = +20.4 (*c* 1.00; CH_3OH), (**D**) $[\alpha]_D^{20}$ = −20.3 (*c* 1.00; CH_3OH).

H-Aib$_2$-L/D-αMv$_2$-NHMe – S16

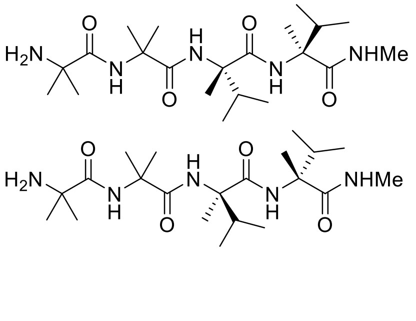

From **S15** (97 mg, 0.21 mmol) according to **General Procedure B**. **S16** was obtained as a clear oil (92 mg, 0.21 mmol, *quant.*). $\mathbf{R_f}$ 0.00

(EtOAc); **δ_H** (400 MHz, CD_3OD) 2.74 (3H, s, CONHCH_3), 2.09-2.00 (2H, m, C*H*$(CH_3)_2$), 1.54 (3H, s, $H_2NC(CH_3)_2$) 1.56 (3H, s, $H_2NC(CH_3)_2$), 1.49 (3H, s, CONHC$(CH_3)_2$), 1.49 (3H, s, CONHC$(CH_3)_2$), 1.45 (CONHC(CH_3), 1.38 (CONHC(CH_3), 1.03 (3H, d, *J* = 6.8, CH$(CH_3)_2$), 0.97 (3H, d, *J* = 6.8, CH$(CH_3)_2$), 0.95 (3H, d, *J* = 7.0, CH$(CH_3)_2$), 0.93 (3H, d, *J* = 7.0, CH$(CH_3)_2$); **δ_C** (125 MHz, CD_3OD) 176.8 (*C*ONHCH_3), 176.5 (*C*ONH), 173.4 (*C*ONH), 173.3 (*C*ONH), 64.9 (NH*C*(CH_3), 64.8 (NH*C*(CH_3), 64.5 (CONH*C*$(CH_3)_2$), 58.6 (H_2N*C*$(CH_3)_2$), 37.1 (*C*H$(CH_3)_2$), 36.7 (*C*H$(CH_3)_2$), 26.5 ($H_2NC(CH_3)_2$), 26.1 (CONH*C*H_3), 25.6 (CONHC$(CH_3)_2$), 25.5 (CONHC$(CH_3)_2$), 23.9 CONHC(CH_3), 18.3 ($H_2NC(CH_3)_2$, 18.3 (CONHC(CH_3), 18.2 (CH$(CH_3)_2$), 17.7 (CH$(CH_3)_2$), 17.6 (CH$(CH_3)_2$), 17.6 (CH$(CH_3)_2$); **ν_{max}/cm^{-1}** 3356m.br (N-H), 2970m (C-H), 2485s (C-H), 1655s (C=O), 1519w (C-N), 1409m (C-N); ***m/z*** (ESI$^+$) 428.4 ($[M+H]^+$, 100%), HRMS (ESI$^+$) found $[M+Na]^+$ 450.3050 $[C_{21}H_{41}N_5O_4Na]^+$ requires 450.3051; (**L**) **$[\alpha]_D^{20}$** = +14.8 (*c* 1.00, CH_3OH), (**D**) **$[\alpha]_D^{20}$** = −14.8 (*c* 1.00, CH_3OH).

Cbz-Ac$_3$C-Aib$_2$-L/D-αMv$_2$-NHMe – S17

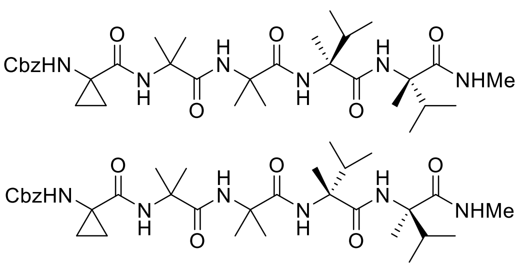

From Cbz-Ac$_3$C-OH (99 mg, 0.42 mmol), TFFH (166 mg, 0.63 mmol), pyridine (34 μL, 0.42 mmol), **S16** (91 mg, 0.21 mmol) and DIPEA (37 μL, 0.21 mmol) according to **General Procedure C**. Crude **S17** was purified by flash column chromatography (99:1 DCM:MeOH increasing to 19:1) to give the pure product (110 mg, 0.17 mmol, 80%). **R_f** 0.30 (94:6 DCM:MeOH); **m.p.** 248-250 °C; **δ_H** (500 MHz, $CDCl_3$) 7.46-7.06 (10H, m, Ph*H*, CON*H*), 6.87 (1H, s, br, N*H*CH_3), 5.21 (1H, d, *J* = 12.0, PhCH_2), 5.06 (1H, d, *J* = 12.0, PHCH_2), 2.73 (3H, s, NHCH_3), 2.01 (1H, sep, *J* = 6.1, C*H*$(CH_3)_2$), 2.00 (1H, sep, *J* = 6.1, C*H*$(CH_3)_2$), 1.88-1.16 (22H, m, C(CH_2-CH_2), NHC$(CH_3)_2$, NHC$(CH_3)_2$, NHC(CH_3), NHC(CH_3)), 0.98 (6H, d, *J* = 6.1, CH$(CH_3)_2$), 0.90 (6H, d, *J* = 6.1, CH$(CH_3)_2$); **δ_C** (125 MHz, $CDCl_3$) 183.8 (*C*ONH), 175.9 (*C*ONH), 175.7 (*C*ONH), 174.4 (*C*ONH), 173.3 (*C*ONH), 157.8 (*C*O_2Bn), 136.8, 128.7, 128.5, 128.0 (Ar), 63.4 (NH*C*(CH_3)), 63.4 (NH*C*(CH_3)), 56.6 (Ph*C*H_2), 55.6 (NH*C*$(CH_3)_2$, NH*C*$(CH_3)_2$), 36.8 (*C*(CH_2-CH_2)), 35.8 (*C*H$(CH_3)_2$), 35.7 (*C*H$(CH_3)_2$), 31.1 (NH*C*H_3), 27.3 (NHC$(CH_3)_2$), 27.1 (NHC$(CH_3)_2$), 27.0 (NHC$(CH_3)_2$), 26.6 (NHC$(CH_3)_2$), 23.3 (NHC(CH_3), 23.0 (NHC(CH_3), 17.9 (CH$(CH_3)_2$), 17.8 (CH$(CH_3)_2$), 17.5 (CH$(CH_3)_2$), 17.4 (CH$(CH_3)_2$), 17.0 (C(CH_2-CH_2), 15.9 (C(CH_2-CH_2); **ν_{max}/cm^{-1}** 3336m (N-H), 3268m (N-H), 2973m (C-H), 1658s (C=O), 1529m (C-N); ***m/z*** (ESI$^+$) 345.8 ($[M+H]^+$, 100%), 667.8 ($[M+Na]^+$, 60%), HRMS (ESI$^+$) found $[M+H]^+$ 645.3968 $[C_{33}H_{53}N_6O_7]^+$ requires 645.3970; (**L**) **$[\alpha]_D^{20}$** = +39.6 (*c* 0.95; CH_3OH, (**D**) **$[\alpha]_D^{20}$** = −39.6 (*c* 1.00; CH_3OH).

H-Ac$_3$C-Aib$_2$-L/D-αMv$_2$-NHMe – S18

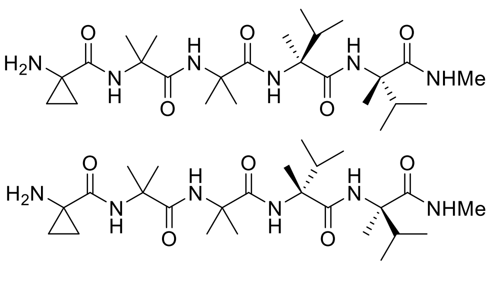

From **S17** (110 mg, 0.17 mmol) according to **General Procedure B**. **S18** was obtained as white powder (86 mg,

0.17 mmol, *quant.*). **R_f** 0.00 (EtOAc); **m.p.** >190 °C (decomp); **δ_H** (400 MHz, CD_3OD) 2.75 (3H, s, NHC*H*3), 2.15 (1H, sep, *J* = 6.8, C*H*(CH3)2), 2.06 (1H, sep, *J* = 6.8, C*H*(CH3)2), 1.46 (3H, s, NHC(C*H*3)2), 1.45 (3H, s, NHC(C*H*3)2), 1.43 (6H, s, NHC(C*H*3)2), 1.36 (3H, s, NHC(C*H*3)), 1.30 (3H, s, NHC(C*H*3)), 1.26-1.23 (2H, m, C(C*H*2-C*H*2)), 1.02 (3H, d, *J* = 6.8, CH(C*H*3)2), 0.98 (3H, d, *J* = 6.8, CH(C*H*3)2), 0.93-0.87 (8H, m, CH(C*H*3)2, C(C*H*2-C*H*2)); **δ_C** (100 MHz, $CDCl_3$) 177.7 (*C*ONH), 177.5 (*C*ONH), 177.5 (*C*ONH), 176.3 (*C*ONH), 175.5 (*C*ONH), 64.7 (NH*C*(CH3)), 64.6 (NH*C*(CH3)), 57.7 (NH*C*(CH3)2), 57.6 (NH*C*(CH-3)2), 37.0 (*C*(CH2-CH2)), 36.9 (*C*H(CH3)2), 36.5 (*C*H(CH3)2), 27.4 (NH*C*H3), 26.6 (NHC(*C*H3)2), 26.5 (NHC(*C*H3)2), 24.2 (NHC(*C*H3)), 23.4 (NH*C*(CH3)), 18.3 (C(*C*H2-*C*H2)), 18.2 (C(*C*H2-*C*H2)), 17.9 (CH(*C*H3)2), 17.5 (CH(*C*H3)2), 17.1 (CH(*C*H3)2), 16.4 (CH(*C*H3)2); **ν_{max}/cm^{-1}** 3338m.br (N-H), 2970m (C-H), 1646s (C=O), 1424s (C-N); ***m/z*** (ESI$^+$) 512 ([M+H]$^+$, 100%), 534 ([M+Na]$^+$, 80%), HRMS (ESI$^+$) found [M+H]$^+$ 511.3598 [$C_{25}H_{47}N_6O_5$]$^+$ requires 321.3602; (**L**) **[α]$_D^{20}$** = +9.2 (*c* 1.00; CH_3OH), (**D**) **[α]$_D^{20}$** = −8.6 (*c* 1.01; CH_3OH).

Cbz-L-αMv2-Aib2-Ac3C-Aib2-L-αMv2-NHMe – 2a

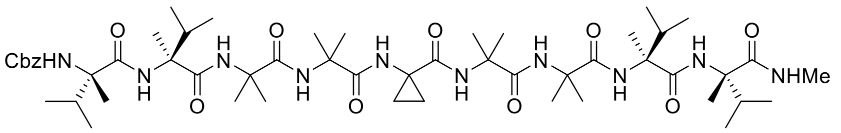

From **S14** (50 mg, 0.09 mmol), EDC.HCl (26 mg, 0.14 mmol), DIPEA (24 µL, 0.14 mmol) and **L-S18** (47 mg, 0.09 mmol) according to **General Procedure F**. Crude **2a** was purified by flash column chromatography (99:1 DCM:MeOH increasing to 19:1) to give the pure product as a white powder (38 mg, 0.04 mmol, 44%). **R_f** 0.21 (19:1 DCM:MeOH); **m.p.** >250 °C; **δ_H** (500 MHz, $CDCl_3$) 8.25 (1H, s, CON*H*), 7.94 (1H, s, CON*H*), 7.78 (1H, s, CON*H*), 7.68 (1H, s, CON*H*), 7.43 (1H, s, CON*H*), 7.37 (6H, s, Ph*H*, CON*H*), 7.22 (1H, s, CON*H*), 7.01 (1H, s, CON*H*), 6.42 (1H, s, CON*H*), 5.48 (1H, s, CON*H*), 5.18 (1H, d, *J* = 12.1, PhC*H*2), 5.03 (1H, d, *J* = 12.1, PhC*H*2), 2.78 (3H, d, *J* = 4.5, NHC*H*3), 2.12 (1H, sep, *J* = 7.2, C*H*(CH3)2), 1.98 (1H, sep, *J* = 6.7, C*H*(CH3)2), 1.87 (1H, sep, *J* = 6.9, C*H*(CH3)2), 1.71-1.63 (3H, m, C(C*H*2-C*H*2), C*H*(CH3)2), 1.55 (3H, s, NHC(C*H*3)2), 1.51 (8H, s, NHC(C*H*3)2, C(C*H*2-C*H*2)), 1.50 (6H, s, NHC(C*H*3)2), 1.48 (3H, s, NHC(C*H*3)2), 1.47 (6H, s, NHC(C*H*3)2), 1.43 (3H, s, NHC(C*H*3)), 1.41 (3H, s, NHC(C*H*3)), 1.37 (3H, s, NHC(C*H*3)), 1.36 (3H, s, NHC(C*H*3)), 1.02 (3H, d, *J* = 6.7, CH(C*H*3)2), 1.01 (3H, d, *J* = 6.9, CH(C*H*3)2), 0.98 (3H, d, *J* = 6.9, CH(C*H*3)2), 0.95 (3H, d, *J* = 6.7, CH(C*H*3)2), 0.94-0.90 (6H, m, CH(C*H*3)2), 0.82 (3H, d, *J* = 6.8, CH(C*H*3)2), 0.78 (3H, d, *J* = 6.8, CH(C*H*3)2); **δ_C** (125 MHz, $CDCl_3$) 178.2, 175.9, 175.8, 175.6, 175.2, 173.4, 173.0, 172.8, 172.5 (*C*ONH), 156.5 (*C*O2Bn), 135.8, 128.9, 128.9, 128.7 (Ar), 67.9 (Ph*C*H2), 63.6, 63.6, 63.4, 62.5 (NH*C*(CH3)), 57.2, 57.1, 56.8, 56.7 (NH*C*(CH3)2), 36.1 (*C*(CH2-CH2)), 35.9, 35.9, 35.9, 35.4 (*C*H(CH3)2), 27.7, 27.7, 27.4, 27.1 (NHC(*C*H3)2), 26.5 (NH*C*H3), 23.2, 22.9, 22.8, 21.2 (NHC(*C*H3)2), 18.3, 18.1, 17.9, 17.9 (NHC(*C*H3)), 17.6, 17.5, 17.4, 17.3, 17.3, 17.2, 17.1 (CH(*C*H3)2), 16.5, 16.0 (C(*C*H2-*C*H2)), 15.2 (CH(*C*H3)2); **ν_{max}/cm^{-1}** 3293m (N-H), 2979w (C-H), 1654s

(C=O), 1528m (C-N); ***m/z*** (ESI$^+$) 1064.8 ([M+Na]$^+$, 100%), HRMS (ESI$^+$) found [M+H]$^+$ 1041.6697 [$C_{53}H_{89}N_{10}O_{11}$]$^+$ requires 1041.6707; **[α]$_D$^{20}** = +48.9 (*c* 1.12; CH_3OH).

Cbz-L-αMv$_2$-Aib$_2$-Ac$_3$C-Aib$_2$-D-αMv$_2$-NHMe – 2b

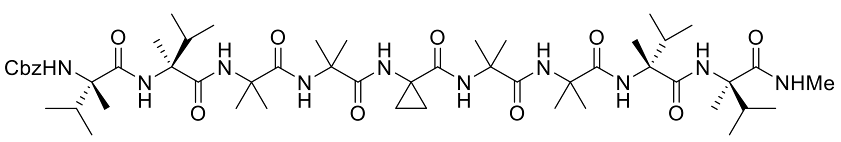

From **S14** (50 mg, 0.09 mmol), EDC.HCl (26 mg, 0.14 mmol), DIPEA (24 µL, 0.14 mmol) and **D-S18** (47 mg, 0.09 mmol) according to **General Procedure F**. Crude **2b** was purified by flash column chromatography (99:1 DCM:MeOH increasing to 19:1) to give the pure product as a white powder (38 mg, 0.04 mmol, 44%). **R*f*** 0.22 (19:1 DCM:MeOH); **m.p.** 243-245 °C; **δ$_H$** (500 MHz, $CDCl_3$) 8.08 (1H, s, CON*H*), 7.90 (1H, s, CON*H*), 7.70 (1H, s, CON*H*), 7.65 (1H, s, CON*H*), 7.40-7.34 (7H, m, Ph*H*, CON*H*), 7.12 (1H, s, CON*H*), 7.11 (1H, s, CON*H*), 6.42 (1H, s, CON*H*), 6.42 (1H, s, CON*H*), 5.39 (1H, s, CON*H*), 5.18 (1H, d, *J* = 12.1, PhC*H*$_2$), 5.03 (1H, d, *J* = 12.1, PhC*H*$_2$), 2.80 (3H, d, *J* = 4.6, NHC*H*$_3$), 2.36 (1H, m, C*H*(CH_3)$_2$), 2.09 (1H, sep, *J* = 6.8, C*H*(CH_3)$_2$), 1.89 (1H, sep, *J* = 6.8, C*H*(CH_3)$_2$), 1.79-1.71 (4H, m, C(C*H*$_2$-C*H*$_2$)), 1.58 (1H, m, C*H*(CH_3)$_2$), 1.54 (3H, s, NHC(C*H*$_3$)$_2$), 1.53 (3H, s, NHC(C*H*$_3$)$_2$), 1.52 (3H, s, NHC(C*H*$_3$)$_2$), 1.51 (3H, s, NHC(C*H*$_3$)$_2$), 1.50 (3H, s, NHC(C*H*$_3$)$_2$), 1.49 (3H, s, NHC(C*H*$_3$)$_2$), 1.47 (3H, s, NHC(C*H*$_3$)$_2$), 1.45 (3H, s, NHC(C*H*$_3$)$_2$), 1.43 (3H, s, NHC(C*H*$_3$)), 1.42 (3H, s, NHC(C*H*$_3$)), 1.37 (3H, s, NHC(C*H*$_3$)), 1.35 (3H, s, NHC(C*H*$_3$)), 1.00-0.90 (18H, m, CH(C*H*$_3$)$_2$), 0.82 (3H, d, *J* = 6.8, CH(C*H*$_3$)$_2$), 0.78 (3H, d, *J* = 6.8, CH(C*H*$_3$)$_2$); **δ$_C$** (100 MHz, $CDCl_3$) 177.6, 176.1, 176.0, 175.8, 175.1, 173.4, 172.9, 172.8, 172.6 (*C*ONH), 156.5 (*C*O$_2$Bn), 135.8, 128.9, 128.9, 128.7 (Ar), 67.8 (Ph*C*H$_2$), 64.1, 63.6, 63.3, 62.5 (NH*C*(CH_3)), 57.3, 57.1, 56.9, 56.8 (NH*C*(CH_3)$_2$), 36.1, 35.9, 35.8, 35.1 (*C*H(CH_3)$_2$), 34.1 (*C*(CH$_2$-CH$_2$)), 27.2, 26.9 (NHC(*C*H$_3$)$_2$), 26.6 (NH*C*H$_3$), 26.0, 25.6, 25.4, 24.8, 23.1, 23.1 (NHC(*C*H$_3$)$_2$), 18.4, 18.0, 17.9, 17.7 (NHC(*C*H$_3$)), 17.6, 17.6, 17.5, 17.4, 17.3, 17.43 17.2, 17.2 (CH(*C*H$_3$)$_2$), 16.4, 15.9 (C(*C*H$_2$-*C*H$_2$)); **ν$_{max}$/cm^{-1}** 3295m (N-H), 2978m (C-H), 1657s (C=O), 1530m (C-N); ***m/z*** (ESI$^+$) 1063.8 ([M+Na]$^+$, 100%), HRMS (ESI$^+$) found [M+NH$_4$]$^+$ 1058.6970 [$C_{53}H_{92}N_{11}O_{11}$]$^+$ requires 1058.6972; **[α]$_D$^{20}** = +23.6 (*c* 1.00; CH_3OH).

N$_3$-Aib-L/D-αMv-NHMe – S19

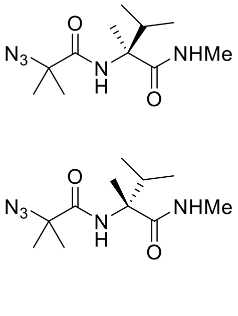

From **S6** (893 mg, 6.92 mmol), oxalyl chloride (0.62 mL, 7.38 mmol), **S3** (664 mg, 4.60 mmol) and Et_3N (0.97 mL, 6.92 mmol) according to **General Procedure A**. Crude **S19** was purified by flash column chromatography (99:1 DCM:MeOH increasing to 19:1) to give the pure compound as a clear oil (740 mg, 2.90 mmol, 63%). **R*f*** 0.43 (19:1 DCM:MeOH); **δ$_H$** (500 MHz, $CDCl_3$) 7.00 (1H, s, br, CON*H*), 6.81 (1H, d, *J* = 4.8, br, N*H*CH$_3$), 2.74 (3H, d, *J* = 4.8, NHC*H*$_3$), 2.44 (1H, sep, *J* = 6.9,

C*H*(CH_3)$_2$), 1.46 (3H, s, NHC(C*H*$_3$)), 1.45 (3H, s, N_3C(C*H*$_3$)$_2$), 1.42 (3H, s, N_3C(C*H*$_3$)$_2$), 0.85 (3H, d, *J* = 6.9, CH(C*H*$_3$)$_2$), 0.81 (3H, d, *J* = 6.9, CH(C*H*$_3$)$_2$); **δ_C** (125 MHz, $CDCl_3$) 173.8 (*C*ONH), 172.3 (*C*ONH), 64.6 (N_3*C*(CH_3)$_2$), 63.9 (NH*C*(CH_3)), 33.4 (*C*H(CH_3)$_2$), 26.5 (NH*C*H$_3$), 24.4 (N_3C(*C*H$_3$)$_2$), 24.3 (N_3*C*(CH_3)$_2$), 17.9 (NHC(*C*H$_3$)), 17.2 (CH(*C*H$_3$)$_2$), 17.1 (CH(*C*H$_3$)$_2$); **ν_{max}/cm^{-1}** 3350m.br (N-H), 2971m (C-H), 2111s (N_3), 1651s (C=O), 1507m (C-N); ***m/z*** (ESI$^+$) 278.2 ([M+Na]$^+$, 95%), 533.4 ([2M+Na]$^+$, 100%), HRMS (ESI$^+$) found [M+Na]$^+$ 278.1579 [$C_{11}H_{21}N_5O_2Na$]$^+$ requires 278.1587; (L) **[α]$_D^{20}$** = +20.6 (*c* 0.99, CH_3OH), (D) **[α]$_D^{20}$** = −20.4 (*c* 1.10; CH_3OH).

H-Aib-L/D-αMv-NHMe – S20

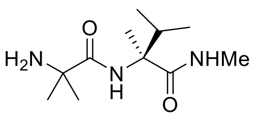

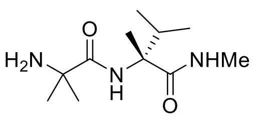

From **S19** (420 mg, 1.64 mmol) according to **General Procedure B**. **S20** obtained as a white powder (376 mg, *quant.*). **R$_f$** 0.00 (EtOAc); **m.p.** 99-101 °C; **δ_H** (500 MHz, CD_3OD) 2.76 (3H, d, *J* = 4.8, NHC*H*$_3$), 2.16 (1H, sep, *J* = 6.8, C*H*(CH_3)$_2$), 1.48 (3H, s, NHC(C*H*$_3$)), 1.40 (3H, s, H_2NC(C*H*$_3$)$_2$), 1.37 (3H, s, H_2NC(C*H*$_3$)$_2$), 1.02 (3H, d, *J* = 6.8, CH(C*H*$_3$)$_2$), 0.95 (3H, d, *J* = 6.8, CH(C*H*$_3$)$_2$); **δ_C** (125 MHz, CD_3OD) 178.4 (*C*ONHCH$_3$), 176.2 (*C*ONH), 63.9 (NH*C*(CH_3)), 56.4 (H_2N*C*(CH_3)$_2$), 36.4 (*C*H(CH_3)$_2$), 27.9 (NHC(*C*H$_3$)), 27.7 (H_2NC(*C*H$_3$)$_2$), 26.6 (NH*C*H$_3$), 17.6 (NHC(*C*H$_3$)), 17.5 (CH(*C*H$_3$)$_2$), 17.4 (CH(*C*H$_3$)$_2$); **ν_{max}/cm^{-1}** 3308m.br (N-H), 2966m (C-H), 2467m (C-H), 1641s (C=O), 1510m (C-N), 1456m (C-N); ***m/z*** (ESI$^+$) 230.2 ([M+H]$^+$, 100%), HRMS (ESI$^+$) found [M+H]$^+$ 230.1858 [$C_{11}H_{24}N_3O_2$]$^+$ requires 230.1863; (L) **[α]$_D^{20}$** = +2.0 (*c* 0.99, CH_3OH), (D) **[α]$_D^{20}$** = −2.0 (*c* 1.00; CH_3OH).

N_3-Aib$_4$-OH – S21

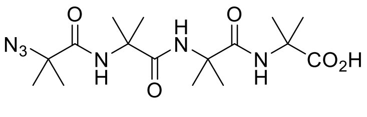

Prepared as previously reported.[5]

N_3-Aib$_5$-D-αMv-NHMe – S22

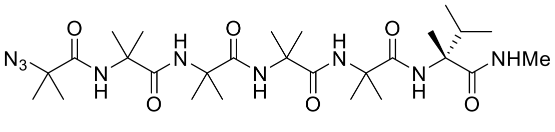

From **S21** (150 mg, 0.39 mmol), EDC.HCl (113 mg, 0.59 mmol), Et_3N (62 μL, 0.59 mmol) and **D-S20** (138 mg, 0.59 mmol) according to **General Procedure F**. Crude **S22** was purified by flash column chromatography (99:1 DCM:MeOH increasing to 19:1) to give the pure compound as a white powder (43 mg, 0.07 mmol, 19%). **R$_f$** 0.12 (19:1 DCM:MeOH); **m.p.** 249-251 °C; **δ_H** (400 MHz, $CDCl_3$) 7.60 (1H, s, CON*H*), 7.48 (1H, s, CON*H*), 7.34 (1H, d, *J* = 4.4, N*H*CH$_3$), 6.98 (1H, s,

CON*H*), 6.90 (1H, s, CON*H*), 6.23 (1H, s, CON*H*), 2.80 (3H, d, *J* = 4.4, NHC*H*$_3$), 2.15 (1H, sep, *J* = 6.8, C*H*(CH$_3$)$_2$), 1.56 (3H, s, NHC(C*H*$_3$)$_2$), 1.54 (3H, s, NHC(C*H*$_3$)$_2$), 1.50 (3H, s, NHC(C*H*$_3$)$_2$), 1.49 (6H, s, NHC(C*H*$_3$)$_2$), 1.48 (3H, s, NHC(C*H*$_3$)$_2$), 1.47 (3H, s, NHC(C*H*$_3$)$_2$), 1.46 (3H, s, NHC(C*H*$_3$)$_2$), 1.45 (6H, s, NHC(C*H*$_3$)$_2$), 1.38 (3H, s, NHC(C*H*$_3$)), 0.99 (3H, d, *J* = 6.8, CH(C*H*$_3$)$_2$), 0.96 (3H, d, *J* = 6.8, CH(C*H*$_3$)$_2$); **δ_C** (100 MHz, CDCl$_3$) 175.4 (*C*ONH), 175.4 (*C*ONH), 174.4 (*C*ONH), 173.5 (*C*ONH), 173.3 (*C*ONH), 173.3 (*C*ONH), 64.1 (N$_3$*C*(CH$_3$)$_2$), 63.4 (NH*C*(CH$_3$)), 57.2 (NH*C*(CH$_3$)$_2$), 57.0 (NH*C*(CH$_3$)$_2$), 57.0 (NH*C*(CH$_3$)$_2$), 56.9 (NH*C*(CH$_3$)$_2$), 35.8 (*C*H(CH$_3$)$_2$), 27.3 (N$_3$C(*C*H$_3$)$_2$), 27.2 (N$_3$C(*C*H$_3$)$_2$), 26.5 (NHC(*C*H$_3$)$_2$, NH*C*H$_3$), 26.1 (NHC(*C*H$_3$)$_2$), 24.5 (NHC(*C*H$_3$)$_2$), 24.4 (NHC(*C*H$_3$)$_2$), 23.8 (NHC(*C*H$_3$)$_2$), 23.6 (NHC(*C*H$_3$)$_2$), 23.5 (NHC(*C*H$_3$)$_2$, NHC(*C*H$_3$)), 17.8 (CH(*C*H$_3$)$_2$), 17.3 (CH(*C*H$_3$)$_2$); **ν_{max}/cm^{-1}** 3322m.br (N-H), 2975w (C-H), 2112m (N$_3$), 1655s (C=O), 1522m (C-N); ***m/z*** (ESI$^+$) 596.5 ([M+H]$^+$, 100%), HRMS (ESI+) found [M+Na]$^+$ 618.3704 [C$_{27}$H$_{49}$N$_9$O$_6$Na]$^+$ requires 618.3704; **[α]$_D^{20}$** = −25.2 (*c* 1.00; CH$_3$OH).

H-Aib$_5$-D-αMv-NHMe – S23

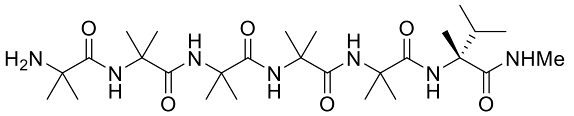

From **S22** (40 mg, 0.07 mmol) according to **General Procedure B**. **S23** was obtained as a white powder (37 mg, 0.07 mmol, *quant.*). **R$_f$** 0.29 (19:1 DCM:MeOH); **m.p.** 210-212 °C; **δ_H** (400 MHz, CDCl$_3$) 8.29 (2H, s, N*H*$_2$), 7.77 (1H, m, CON*H*), 7.69 (1H, m, CON*H*), 7.35 (1H, m, CON*H*), 6.99 (1H, m, CON*H*), 6.90 (1H, s, br, CON*H*), 6.59 (1H, s, br, CON*H*), 2.78 (3H, d, *J* = 4.5, NHC*H*$_3$), 2.14 (1H, m, *J* = 6.8, C*H*(CH$_3$)$_2$), 1.56-1.31 (33H, m, NHC(C*H*$_3$)$_2$, NHC(C*H*$_3$)), 0.98 (3H, d, *J* = 6.8, CH(C*H*$_3$)$_2$), 0.92 (3H, d, *J* = 6.4, CH(C*H*$_3$)$_2$); **δ_C** (100 MHz, CDCl$_3$) 178.8 (*C*ONHCH$_3$), 175.9 (*C*ONH), 175.7 (*C*ONH), 174.7 (*C*ONH), 174.3 (*C*ONH), 173.9 (*C*ONH), 63.2 (NH*C*(CH$_3$)), 57.1 (NH*C*(CH$_3$)$_2$), 56.8 (NH*C*(CH$_3$)$_2$), 56.7 (NH*C*(CH$_3$)$_2$), 56.3 (NH*C*(CH$_3$)$_2$), 54.8 (H$_2$N*C*(CH$_3$)$_2$), 35.8 (*C*H(CH$_3$)$_2$), 29.0 (H$_2$NC(*C*H$_3$)$_2$), 28.9 (H$_2$NC(*C*H$_3$)$_2$), 27.4 (NHC(*C*H$_3$)$_2$), 26.9 (NHC(*C*H$_3$)$_2$), 26.6 (NHC(*C*H$_3$)$_2$, NH*C*H$_3$), 26.4 (NHC(*C*H$_3$)$_2$), 23.5 (NHC(*C*H$_3$)$_2$), 23.4 (NHC(*C*H$_3$)$_2$), 23.3 (NHC(*C*H$_3$)$_2$, NHC(*C*H$_3$)), 17.9 (CH(*C*H$_3$)$_2$), 17.4 (CH(*C*H$_3$)$_2$); **ν_{max}/cm^{-1}** 3308m (N-H), 2980m (C-H), 1757s (C=O), 1527m (C-N); ***m/z*** (ESI$^+$) 570.5 ([M+H]$^+$, 100%), HRMS (ESI+) found [M+Na]$^+$ 592.3798 [C$_{27}$H$_{51}$N$_7$O$_6$Na]$^+$ requires 592.3799; **[α]$_D^{20}$** = −12.8 (c = 1.00; CH$_3$OH).

H-Bip-O^{t}Bu – S24

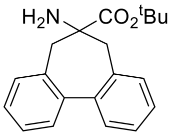

From Cbz-Bip-O^{t}Bu (428 mg, 0.96 mmol) according to **General Procedure B**. **S24** was obtained as a pale yellow powder (292 mg, 0.94 mmol, 98%). **δ_H** (400 MHz, CDCl$_3$) 7.45-7.27 (8H, m, Ar*H*), 3.02 (2H, d, *J* = 13.5, ArC*H*$_2$), 2.56-2.41 (2H, m, ArC*H*$_2$), 1.46 (9H, s, C(C*H*$_3$)$_3$); **δ_C** (100 MHz, CDCl$_3$) 174.2 (*C*O$_2$), 140.7, 130.3, 128.5, 127.6 (Ar), 82.0 (*C*(CH$_3$)$_3$), 68.6 (ArCH$_2$*C*), 42.6 (Ar*C*H$_2$), 28.1 (C(*C*H$_3$)$_3$). Data are in accordance with literature values.[6]

N_3-Aib-Bip-O^{t}Bu – S25

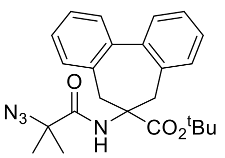

From **S6** (77 mg, 0.60 mmol), oxalyl chloride (54 µL, 0.64 mmol), **S24** (100 mg, 0.40 mmol) and Et_3N (170 µL, 1.20 mmol) according to **General Procedure A**. Crude **S25** was purified by flash column chromatography (9:1 PE:EtOAc) obtained as a glassy solid (115 mg, 0.27 mmol, 68%). **R$_f$** 0.57 (4:1 PE:EtOAc); **m.p.** 131-133 °C; **δ_H** (400 MHz, $CDCl_3$) 7.46-7.37 (4H, m, Ar*H*), 7.32 (2H, q, *J* = 7.1, Ar*H*), 7.20 (2H, d, *J* = 7.1, Ar*H*), 6.72 (1H, s, CON*H*), 3.33-2.29 (2H, m, ArC*H$_2$*), 3.15 (2H, d, *J* = 11.3, ArC*H$_2$*), 1.56 (6H, s, NHC(C*H$_3$*)$_2$), 1.47 (9H, s, C(C*H$_3$*)$_3$); **δ_C** (100 MHz, $CDCl_3$) 171.5 (*C*O$_2$) 170.5 (*C*ONH), 140.8, 135.2, 130.0, 128.4, 127.9, 127.8 (Ar), 81.6 (*C*(CH$_3$)$_3$), 69.1 (ArCH$_2$*C*), 64.5 (N$_3$*C*(CH$_3$)$_2$), 40.1 (Ar*C*H$_2$), 28.0 (C(*C*H$_3$)$_3$), 24.5 (N$_3$C(*C*H$_3$)$_2$); **ν_{max}/cm^{-1}** 3411w (N-H), 2978m (C-H), 2111s (N_3) 1736s (C=O), 1505s (C-N); ***m/z*** (ESI$^+$) 421.3 ([M+H]$^+$, 100%), 443.3 ([M+Na]$^+$, 50%), HRMS (ESI$^+$) found [M+Na]$^+$ 443.2070 [$C_{24}H_{28}N_4O_3Na$]$^+$ requires 443.2059.

N_3-Aib-Bip-OH – S26

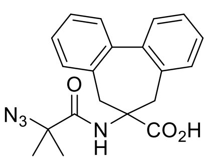

From **S25** (115 mg, 0.27 mmol) according to **General Procedure D**. Crude **S26** was purified by flash column chromatography (97:3 DCM:MeOH increasing to 19:1) to give the pure product as a clear oil (87 mg, 0.24 mmol, 89%). **R$_f$** 0.27 (19:1 DCM:MeOH); **δ_H** (500 MHz, $CDCl_3$) 9.34 (1H, s, br, CO$_2$*H*), 7.47-7.41 (4H, m, Ar*H*), 7.34 (2H, dd, *J* = 6.0, Ar*H*), 7.30-7.23 (2H, m, Ar*H*), 6.89 (1H, s, CON*H*), 3.46-2.29 (2H, m, ArC*H$_2$*), 3.34-3.12 (2H, m, ArC*H$_2$*), 1.57 (6H, s, NHC(C*H$_3$*)$_2$); **δ_C** (125 MHz, $CDCl_3$) 176.5 (*C*O$_2$H) 172.6 (*C*ONH), 140.7, 134.6, 130.0, 128.5, 128.1, 127.9 (Ar), 68.8 (ArCH$_2$*C*), 64.3 (N$_3$*C*(CH$_3$)$_2$), 38.9 (Ar*C*H$_2$), 24.4 (N$_3$C(*C*H$_3$)$_2$); **ν_{max}/cm^{-1}** 2978s.br (O-H, N-H, C-H), 2112s (N_3) 1715m (C=O), 1680m (C=O), 1509m (C-N); ***m/z*** (ESI$^-$) 365.2 ([M+H]$^+$, 100%), HRMS (ESI$^+$) found [M+Na]$^+$ 387.1442 [$C_{20}H_{20}N_4O_3Na$]$^+$ requires 387.1433.

N_3-Aib-Bip-Aib$_5$-D-αMv-NHMe – S27

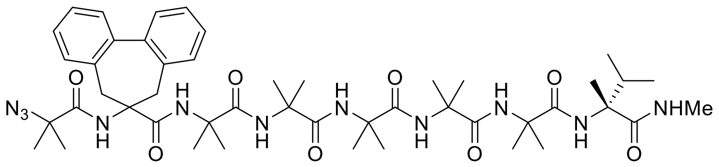

From **S26** (46 mg, 0.13 mmol), EDC.HCl (36 mg, 0.19 mmol), Et_3N (34 µL, 0.19 mmol) and **S23** (38 mg, 0.07 mmol) according to **General Procedure F**. Crude **S27** was purified by flash column chromatography (99:1 DCM:MeOH increasing to 19:1) to give the pure compound

as a clear film (36 mg, 0.04 mmol, 57%). **R$_f$** 0.27 (19:1 DCM:MeOH); **m.p.** 230-231 °C; **δ$_H$** (400 MHz, $CDCl_3$) 7.77 (1H, s, CON*H*), 7.73 (1H, s, CON*H*), 7.59 (1H, s, CON*H*), 7.51-7.30 (8H, m, Ar*H*), 7.23-7.12 (3H, m, CON*H*), 7.02 (1H, s, CON*H*), 6.82 (1H, s, CON*H*), 3.33 (1H, m, ArC*H*$_2$), 3.08 (1H, d, *J* = 12.0, ArC*H*$_2$), 2.96-2.25 (2H, m, ArC*H*$_2$), 2.77 (3H, d, *J* = 4.5, NHC*H*$_3$), 2.13 (1H, m, C*H*(CH$_3$)$_2$), 1.60 (6H, s, NHC(C*H*$_3$)$_2$), 1.51 (3H, s, NHC(C*H*$_3$)$_2$), 1.48 (12H, s, NHC(C*H*$_3$)$_2$, 1.47 (3H, s, NHC(C*H*$_3$)$_2$), 1.46 (3H, s, NHC(C*H*$_3$)$_2$), 1.45-1.39 (9H, m, NHC(C*H*$_3$)$_2$), NHC(C*H*$_3$), N$_3$C(C*H*$_3$)$_2$), 1.39-1.28 (N$_3$C(C*H*$_3$)$_2$), 0.99 (3H, d, *J* = 6.6, CH(C*H*$_3$)$_2$), 0.93 (3H, d, *J* = 6.6, CH(C*H*$_3$)$_2$); **δ$_C$** (100 MHz, $CDCl_3$) 176.1 (*C*ONH), 175.7 (*C*ONH), 175.5 (*C*ONH), 175.2 (*C*ONH), 174.8 (*C*ONH), 174.4 (*C*ONH), 173.5 (*C*ONH), 171.9 (*C*ONH), 140.7, 140.5, 135.0, 133.4, 130.2, 130.1, 129.4, 128.7, 128.6, 128.5, 128.1, 128.0 (Ar), 69.5 (ArCH$_2$*C*), 64.1 (N$_3$*C*(CH$_3$)$_2$), 63.2 (NH*C*(CH$_3$)), 57.1 (NH*C*(CH$_3$)$_2$), 57.1 (NH*C*(CH$_3$)$_2$), 56.9 (NH*C*(CH$_3$)$_2$), 56.8 (NH*C*(CH$_3$)$_2$), 56.7 (NH*C*(CH$_3$)$_2$), 35.8 (Ar*C*H$_2$), 29.8 (*C*H(CH$_3$)$_2$), 27.5-26.4 (N$_3$C(*C*H$_3$)$_2$, NHC(*C*H$_3$)$_2$, NH*C*H$_3$), 24.7-24.3 (NHC(*C*H$_3$)$_2$), 23.7-22.9 (NHC(*C*H$_3$)$_2$, NHC(*C*H$_3$)), 17.9 (CH(*C*H$_3$)$_2$), 17.4 (CH(*C*H$_3$)$_2$); **ν$_{max}$/cm^{-1}** 3305m (N-H), 2982w (C-H), 2114m (N$_3$), 1656m (C=O), 1526m (C-N); ***m/z*** (ESI$^+$) 1159.8 ([M+H]$^+$, 100%), HRMS (ESI+) found [M+Na]$^+$ 938.5182 [$C_{47}H_{69}N_{11}O_8Na$]$^+$ requires 938.5228; **[α]$_D^{20}$** = −49.6 (*c* 1.00; CH$_3$OH).

H-Aib-Bip-Aib$_5$-D-αMv-NHMe – S28

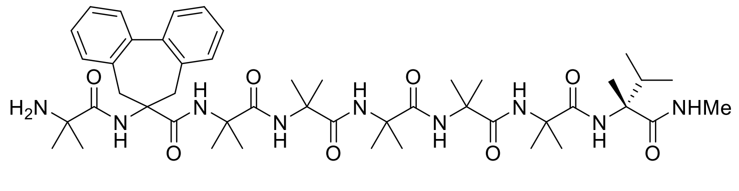

From **S27** (36 mg, 0.04 mmol) according to **General Procedure B**. **S28** was obtained as a clear film (21 mg, 0.02 mmol, 58%). **R$_f$** 0.17 (19:1 DCM:MeOH); **δ$_H$** (500 MHz, $CDCl_3$) 8.54 (2H, s, br, N*H*$_2$), 7.86-.6.93 (14H, m, Ar*H*, CON*H*), 6.83 (1H, s, br, CON*H*), 6.34 (1H, s, br, CON*H*), 3.74-2.95 (2H, m, ArC*H*$_2$), 2.79 (3H, d, *J* = 4.5, NHC*H*$_3$), 2.21-2.09 (3H, m, ArC*H*$_2$, C*H*CH$_3$)$_2$), 1.64-1.20 (36H, s, NHC(C*H*$_3$)$_2$, NHC(C*H*$_3$)), 0.99 (3H, d, *J* = 6.5, CH(C*H*$_3$)$_2$), 0.93 (3H, d, *J* = 6.2, CH(C*H*$_3$)$_2$); **δ$_C$** (125 MHz, $CDCl_3$) 179.2 (*C*ONH), 176.2 (*C*ONH), 175.8 (*C*ONH), 175.7 (*C*ONH), 175.4 (*C*ONH), 174.9 (*C*ONH), 174.7 (*C*ONH), 174.7 (*C*ONH), 140.5, 135.7, 133.8, 130.2, 129.5, 128.6, 128.5, 128.4, 128.3, 127.9 (Ar), 69.1 (ArCH$_2$*C*), 63.2 (NH*C*(CH$_3$)), 57.4 (NH*C*(CH$_3$)$_2$), 57.1 (NH*C*(CH$_3$)$_2$), 56.9 (NH*C*(CH$_3$)$_2$), 56.8 (NH*C*(CH$_3$)$_2$), 56.6 (NH*C*(CH$_3$)$_2$), 55.0 (H$_2$N*C*(CH$_3$)$_2$), 35.9 (Ar*C*H$_2$), 29.8 (*C*H(CH$_3$)$_2$), 29.1 (H$_2$NC(*C*H$_3$)$_2$), 27.7-26.3 (H$_2$NC(*C*H$_3$)$_2$, NHC(*C*H$_3$)$_2$, NH*C*H$_3$), 23.7-22.7 (NHC(*C*H$_3$)$_2$, NHC(*C*H$_3$)), 17.9 (CH(*C*H$_3$)$_2$), 17.4 (CH(*C*H$_3$)$_2$); **ν$_{max}$/cm^{-1}** 3328m.br (N-H), 2971m (C-H), 1667s (C=O), 1512m (C-N); ***m/z*** (ESI$^+$) 891.5 ([M+H]$^+$, 100%), HRMS (ESI+) found [M+Na]$^+$ 912.5279 [$C_{47}H_{71}N_9O_8$]$^+$ requires 912.5323; **[α]$_D^{20}$** = −74.0 (*c* 1.00; CH$_3$OH).

Cbz-L-αMv-Aib-Bip-Aib$_5$-D-αMv-NHMe – 4a

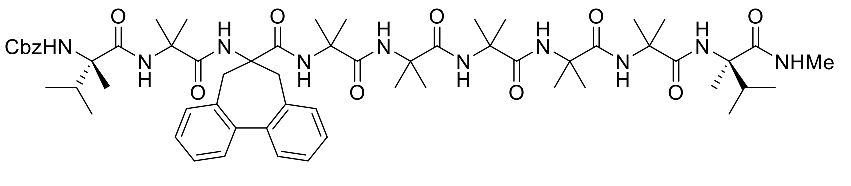

From **L-S1** (12 mg, 0.05 mmol), TFFH (18 mg, 0.07 mmol), pyridine (4 µL, 3.04 mmol), **S28** (21 mg, 0.02 mmol) and DIPEA (4 L. µL, 0.02 mmol) according to **General Procedure C**. Crude **4a** was purified by flash column chromatography (99:1 DCM:MeOH increasing to 19:1) to give the pure product (16 mg, 0.01 mmol, 62%). $\mathbf{R_f}$ 0.30 (19:1 DCM:MeOH); **m.p.** >250 °C; δ_H (400 MHz, $CDCl_3$) 7.80-6.96 (21H, m, Ar*H*, CON*H*), 6.52 (1H, s, CON*H*), 5.43 (1H, s, CON*H*) 5.22-4.58 (2H, m, PhC*H*$_2$), 3.34-1.89 (9H, m, ArC*H*$_2$, NHC*H*$_3$, C*H*(CH_3)$_2$), 1.63-1.08 (42H, m, NHC(C*H*$_3$)$_2$, NHC(C*H*$_3$)), 1.05-0.76 (12H, m, CH(C*H*$_3$)$_2$); δ_C (125 MHz, CD_2Cl_2/CD_3OD) 177.3 (*C*ONH), 177.3 (*C*ONH), 176.8 (*C*ONH), 176.7 (*C*ONH), 176.7 (*C*ONH), 176.4 (*C*ONH), 176.1 (*C*ONH), 176.0 (*C*ONH), 176.0 (*C*ONH), 157.0 (*C*O$_2$Bn), 141.4, 136.8, 130.5, 129.1, 128.8, 128.6, 128.6, 128.4, 128.2, 128.0 (Ar), 70.4 (ArCH_2*C*), 67.4 (Ph*C*H$_2$), 63.7 (NH*C*(CH_3)), 57.7-57.1 (NH*C*(CH_3)$_2$), 36.5-35.1 (Ar*C*H$_2$), 32.5 (NHC(*C*H$_3$)), 30.4-22.8 (NH*C*H$_3$, NHC(*C*H$_3$)$_2$, NHC(*C*H$_3$)), 18.1-17.2 (CH(*C*H$_3$)$_2$); ν_{max}**/cm^{-1}** 3303w (N-H), 2927w (C-H), 1653s (C=O), 1521m (C-N); ***m/z*** (ESI$^+$) 1159.7 ([M+Na]$^+$, 100%), HRMS (ESI+) found [M+Na]$^+$ 1159.6530 [$C_{61}H_{88}N_{10}O_{11}Na$]$^+$ requires 1159.6526; $[\alpha]_D^{20}$ = −5.00 (*c* 0.10; CH_3OH).

Cbz-L-αMv-Aib$_3$-O^{t}Bu – S29

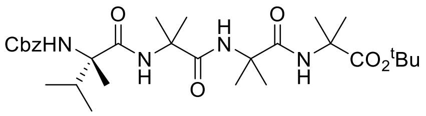

Prepared as previously reported.[7]

Cbz-L-αMv-Aib$_3$-OH – S30

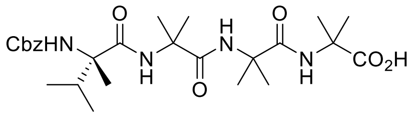

From **S29** (745 mg, 1.29 mmol) according to **General Procedure D**. Crude **S30** was purified by flash column chromatography (99:1 DCM:MeOH increasing to 19:1) to give the pure product as white powder (593 mg, 1.14 mmol, 88%). $\mathbf{R_f}$ 0.32 (19:1 DCM:MeOH); **m.p.** 176-178 °C; δ_H (500 MHz, $CDCl_3$) 7.57 (2H, s, CON*H*), 7.41-7.30 (5H, m, Ph*H*), 6.41 (1H, s, br, CON*H*), 5.39 (1H, s, br, CON*H*), 5.18 (1H, d, *J* = 12.2, PhC*H*$_2$), 5.03 (1H, d, *J* = 12.2, PhC*H*$_2$), 1.94 (1H, m, C*H*(CH_3)$_2$), 1.60 (3H, s, NHC(C*H*$_3$)$_2$), 1.55 (3H, s, NHC(C*H*$_3$)$_2$), 1.46 (3H, s, NHC(C*H*$_3$)$_2$), 1.43 (6H, s, NHC(C*H*$_3$)$_2$), 1.39 (3H, s, NHC(C*H*$_3$)$_2$), 1.39 (3H, s, NHC(C*H*$_3$)), 0.97 (3H, d, *J* = 5.8, CH(C*H*$_3$)$_2$), 0.93 (3H, d, *J* = 6.0, CH(C*H*$_3$)$_2$); δ_C (100 MHz, $CDCl_3$) 176.0 (*C*O$_2$H), 175.9 (*C*ONH), 174.5 (*C*ONH), 173.0 (*C*ONH), 156.2 (*C*O$_2$Bn), 136.2, 128.9, 128.8, 128.3 (Ar), 67.6 (NH*C*(CH_3)), 63.1 (Ph*C*H$_2$), 57.8 (NH*C*(CH_3)$_2$), 57.0 (NH*C*(CH_3)$_2$), 56.8 (NH*C*(CH_3)$_2$), 35.6 (*C*H(CH_3)$_2$), 26.8 (NHC(*C*H$_3$)$_2$), 26.8 (NHC(*C*H$_3$)$_2$), 25.8 (NHC(*C*H$_3$)$_2$), 24.7 (NHC(*C*H$_3$)$_2$), 23.6 (NHC(*C*H$_3$)$_2$), 23.4 (NHC(*C*H$_3$)$_2$, NHC(*C*H$_3$)), 17.4 (CH(*C*H$_3$)$_2$), 17.3 (CH(*C*H$_3$)$_2$); ν_{max}**/cm^{-1}** 3306m.br

(O-H, N-H), 2981m (C-H), 1660s (C=O), 1524m (C-N); ***m/z*** (ESI$^+$) 521.3 ([M+H]$^+$, *100%*), HRMS (ESI$^+$) found [M+Na]$^+$ 543.2795 [$C_{26}H_{40}N_4O_7Na$]$^+$ requires 543.2795; **[α]$_D^{20}$** = +21.2 (*c* 1.00; CH_3OH).

Cbz-L-αMv-Aib$_3$-Bip-O^{t}Bu – S31

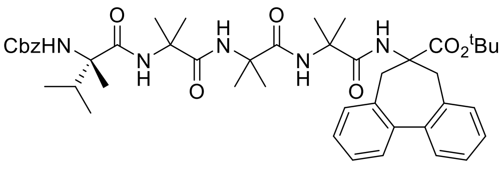

From **S30** (505 mg, 0.97 mmol), EDC.HCl (278 mg, 1.46 mmol), Et_3N (0.26 mL, 1.46 mmol) and **S24** (300 mg, 0.97 mmol) according to **General Procedure F**. Crude **S31** was purified by flash column chromatography (99:1 DCM:MeOH increasing to 19:1) to give the pure product as an off white powder. **R$_f$** 0.43 (19:1 DCM:MeOH); **m.p.** 150-151 °C; **δ$_H$** (400 MHz, $CDCl_3$) 7.66-7.08 (17H, m, N*H*, Ar*H*), 6.13 (1H, d, *J* = 9.6, CON*H*), 5.22 (1H, d, *J* = 9.7, CON*H*), 5.15 (1H, d, *J* = 12.1, PhC*H*$_2$), 4.99 (1H, d, *J* = 12.1, PhC*H*$_2$), 3.20-2.79 (4H, m, ArC*H*$_2$), 1.89 (1H, sep, *J* = 6.7, C*H*(CH_3)$_2$), 1.75-1.18 (30H, m, NHC(C*H*$_3$)$_2$, NHC(C*H*$_3$), C(C*H*$_3$)$_3$), 0.97 (3H, d, *J* = 6.4, CH(C*H*$_3$)$_2$), 0.95-0.89 (3H, m, CH(C*H*$_3$)$_2$); **δ$_C$** (100 MHz, $CDCl_3$) 174.4 (*C*O-NH), 173.6 (*C*ONH), 173.5 (*C*ONH), 172.4 (*C*ONH), 171.6 (*C*O$_2$C(*C*H$_3$)$_3$), 156.0 (*C*O$_2$Bn), 141.3, 140.9, 136.1, 135.5, 135.4, 130.8, 129.6, 129.5, 128.8, 128.7, 128.3, 127.9, 127.7, 127.5, 127.4, 127.2, 127.1, 126.4 (Ar), 80.6 (*C*(CH_3)$_3$)$_a$, 80.3 (*C*(CH_3)$_3$)$_b$, 69.7 (ArCH$_2$*C*)$_a$, 69.4 (ArCH$_2$*C*)$_b$, 67.4 (NH*C*(CH_3)), 63.0 (Ph*C*H$_2$), 57.0 (NH*C*(CH_3)$_2$), 56.9 (NH*C*(CH_3)$_2$), 56.7 (NH*C*(CH_3)$_2$), 40.6 (Ar*C*H$_2$), 36.6 (Ar*C*H$_2$), 35.5 (*C*H(CH_3)$_2$), 29.0 (C(*C*H$_3$)$_3$), 27.6 (NHC(*C*H$_3$)$_2$), 26.9 (NHC(*C*H$_3$)$_2$), 26.6 (NHC(*C*H$_3$)$_2$), 23.7 (NHC(*C*H$_3$)$_2$), 23.5 (NHC(*C*H$_3$)$_2$), 23.2 (NHC(*C*H$_3$)$_2$), 22.7 (NHC(*C*H$_3$)), 17.6 (CH(*C*H$_3$)$_2$), 17.4 (CH(*C*H$_3$)$_2$); **ν$_{max}$/cm^{-1}** 3322m (N-H), 2977m (C-H), 1665s (C=O), 1525m (C-N); ***m/z*** (ESI$^+$) 834.4 ([M+Na]$^+$, 100%), HRMS (ESI$^+$) found [M+Na]$^+$ 834.4425 [$C_{46}H_{61}N_5O_8Na$]$^+$ requires 834.4412; **[α]$_D^{20}$** = +10.0 (*c* 0.50; CH_3OH).

Cbz-L-αMv-Aib$_3$-Bip-OH – S32

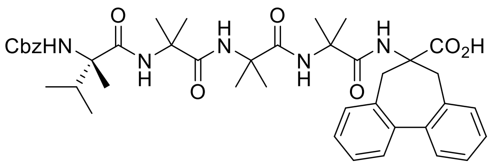

From **S31** (536 mg, 0.66 mmol) according to **General Procedure D**. Crude **S32** was purified by flash column chromatography (99:1 DCM:MeOH increasing to 19:1) to give the pure product as white powder (480 mg, 0.63 mmol, 95%). **R$_f$** 0.17 (19:1 DCM:MeOH); **m.p.** 131-133 °C; **δ$_H$** (400 MHz, CD_3OD) 7.45-7.10 (13H, m, Ar*H*), 5.17 (1H, d, *J* = 12.7, PhC*H*$_2$), 5.03 (1H, d, *J* = 12.7, PhC*H*$_2$), 3.13-2.80 (4H, m, ArC*H*$_2$), 1.98 (1H, sep, *J* = 6.5, C*H*(CH_3)$_2$), 1.62-1.48 (6H, m, NHC(C*H*$_3$)$_2$), 1.41-1.22 (9H, m, C(C*H*$_3$)$_2$), 1.18-1.01 (6H, m, NHC(C*H*$_3$)$_2$, NHC(C*H*$_3$)), 0.99 (3H, d, *J* = 6.5, CH(C*H*$_3$)$_2$), 0.92 (3H, d, *J* = 6.5, CH(C*H*$_3$)$_2$); **δ$_C$** (125 MHz, CD_3OD) 189.1 (CO_2H), 177.1 (*C*ONH), 177.0 (*C*ONH), 175.7 (*C*ONH), 175.5 (*C*ONH), 158.1 (*C*O$_2$Bn), 142.2, 138.6, 137.6, 130.6, 129.6, 129.0, 128.8, 128.8, 128.7, 128.5 (Ar), 70.8 (ArCH$_2$*C*), 67.7 (NH*C*(CH_3)), 63.9 (Ph*C*H$_2$), 58.0 (NH*C*(CH_3)$_2$), 57.9 (NH*C*(CH_3)$_2$), 57.6 (NH*C*(CH_3)$_2$), 41.6 (Ar*C*H$_2$), 36.0 (*C*H(CH_3)$_2$), 27.5 (NHC(*C*H$_3$)$_2$), 26.9 (NHC(*C*H$_3$)$_2$), 26.4 (NHC(*C*H$_3$)$_2$), 24.4 (NHC(*C*H$_3$)$_2$), 24.2 (NHC(*C*H$_3$)$_2$), 23.8

(NHC(CH_3)$_2$), 18.0 (CH(CH_3)$_2$), 17.9 (CH(CH_3)$_2$) 17.6 (NHC(CH_3)); **ν_{max}/cm^{-1}** 3299m.br (O-H, N-H), 2982m (C-H), 1703s (C=O), 1658m (C=O), 1528m (C-N); ***m/z*** (ESI$^-$) 754.6 ([M-H]$^-$, 100%), HRMS (ESI$^+$) found [M+Na]$^+$ 778.3784 [$C_{42}H_{53}N_5O_8Na$]$^+$ requires 778.3792; **$[\alpha]_D^{20}$** = +20.0 (*c* 0.20; CH_3OH).

Cbz-L-αMv-Aib$_3$-Bip-Aib$_2$-O^{t}Bu – S33

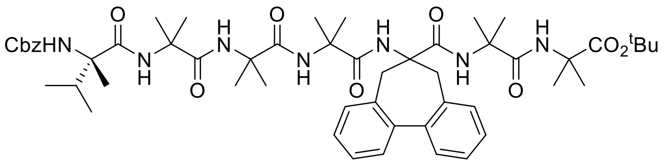

From **S32** (461 mg, 0.61 mmol), EDC.HCl (175 mg, 0.92 mmol), Et_3N (0.13 mL, 0.92 mmol) and **S10** (164 mg, 0.67 mmol) according to **General Procedure F**. Crude **S33** was purified by flash column chromatography twice (99:1 DCM:MeOH increasing to 19:1) to give the pure product as white powder (248 mg, 0.25 mmol, 41%). **R_f** 0.44 (19:1 DCM:MeOH); **m.p.** 224-226 °C; **δ_H** (400 MHz, $CDCl_3$) 7.75 (1H, s, CON*H*), 7.67 (1H, s, CON*H*), 7.55 (1H, s, CON*H*), 7.45 (1H, s, CON*H*), 7.39 (1H, s, CON*H*), 7.37-7.27 (8H, m, Ar*H*), 7.25-7.02 (5H, m, Ar*H*), 6.18 (1H, d, CON*H*), 5.70 (1H, s, CON*H*), 5.16 (1H, d, *J* = 12.2, PhCH_2), 4.99 (1H, d, *J* = 12.2 PhCH_2), 3.28 (1H, d, *J* = 13.9, ArCH_2), 3.06 (1H, d, *J* = 13.9, ArCH_2), 2.99 (1H, d, *J* = 13.6, ArCH_2), 2.74 (1H, d, *J* = 13.6, ArCH_2), 1.83 (1H, m, C*H*(CH_3)$_2$), 1.67 (3H, s, NHC(CH_3)$_2$), 1.58 (3H, s, NHC(CH_3)$_2$), 1.56 (6H, s, NHC(CH_3)$_2$), 1.48 (3H, s, NHC(CH_3)$_2$), 1.46 (3H, s, NHC(CH_3)$_2$), 1.42 (12H, s, NHC(CH_3)$_2$, C(CH_3)$_3$), 1.39 (3H, s, NHC(CH_3)$_2$), 1.35 (3H, s, NHC(CH_3)), 0.96-0.91 (6H, CH(CH_3)$_2$, NHC(CH_3)$_2$), 0.88 (3H, d, *J* = 6.4, CH(CH_3)$_2$), 0.77 (3H, s, NHC(CH_3)$_2$); **δ_C** (100 MHz, $CDCl_3$) 174.6 (*C*ONH), 174.3 (*C*ONH), 173.7 (*C*ONH), 173.5 (*C*ONH), 173.3 (*C*ONH), 172.2 (*C*ONH), 171.6 (*C*O_2C(CH_3)$_3$), 156.0 (*C*O_2Bn), 141.3, 141.0, 141.0, 140.6, 137.5, 137.1, 136.0, 135.6, 135.5, 132.4, 132.3, 130.9, 129.6, 129.5, 128.9, 128.8, 128.4, 128.0, 127.7, 127.5, 127.5, 127.4, 127.2, 127.1, 127.1, 126.6, 126.4 (Ar)$_{a,b}$, 80.6 (*C*(CH_3)$_3$)$_a$, 80.4 (*C*(CH_3)$_3$)$_b$, 69.7 (ArCH_2*C*)$_a$, 69.5 (ArCH_2*C*)$_b$, 67.6 (Ph*C*H_2), 63.0 (NH*C*(CH_3)), 57.0 (NH*C*(CH_3)$_2$), 56.9 (NH*C*(CH_3)$_2$), 56.7 (NH*C*(CH_3)$_2$), 56.6 (NH*C*(CH_3)$_2$), 56.5 (NH*C*(CH_3)$_2$), 40.6 (Ar*C*H_2), 36.7 (Ar*C*H_2)$_a$, 36.2 (Ar*C*H_2)$_b$, 35.7 (*C*H(CH_3)$_2$), 28.1 (C(*C*H_3)$_3$, NHC(*C*H_3)$_2$), 27.6 (NHC(*C*H_3)$_2$), 27.0 (NHC(*C*H_3)$_2$), 26.7 (NHC(*C*H_3)$_2$), 23.7 (NHC(*C*H_3)$_2$), 23.5 (NHC(*C*H_3)$_2$), 23.2 (NHC(*C*H_3)$_2$), 22.8 (NHC(*C*H_3)$_2$), 17.7 (NHC(*C*H_3)), 17.4 (CH(*C*H_3)$_2$), 17.2 (CH(*C*H_3)$_2$); **ν_{max}/cm^{-1}** 3304s.br (N-H), 2981s (C-H), 1657s (C=O), 1528s (C-N), 1531m (C-N); ***m/z*** (ESI$^+$) 491.0 ([M+2H]$^{2+}$, 100%), HRMS (ESI$^+$) found [M+Na]$^+$ 1004.5445 [$C_{54}H_{75}N_7O_{10}Na$]$^+$ requires 1004.5473; **$[\alpha]_D^{20}$** = +65.2 (*c* 1.00; CH_3OH).

Cbz-L-αMv-Aib$_3$-Bip-Aib$_2$-OH – S34

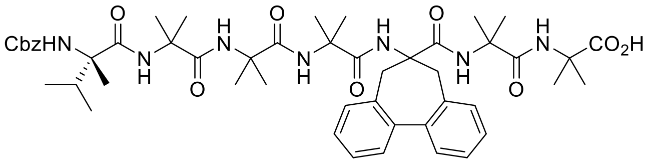

From **S33** (371 mg, 0.38 mmol) according to **General Procedure D**. Crude **S34** was purified by flash column chromatography (99:1

DCM:MeOH increasing to 19:1) to give the pure product as white powder (212 mg, 0.23 mmol, 61%). **R*f*** 0.16 (19:1 DCM:MeOH); **m.p.** 242-243 °C; **δ_H** (400 MHz, $CDCl_3$) 7.95-7.73 (3H, m, CONH), 7.47-6.99 (16H, m, ArH, CONH), 6.85 (1H, d, CONH), 6.34 (1H, s, CONH), 5.14 (1H, d, *J* = 12.4, $PhCH_2$), 4.93 (1H, d, *J* = 12.4, $PhCH_2$), 3.20 (1H, d, *J* = 13.2, $ArCH_2$), 3.02 (1H, d, *J* = 13.7, $ArCH_2$), 2.96 (1H, d, *J* = 13.2, $ArCH_2$), 2.73 (1H, d, *J* = 13.7, $ArCH_2$), 1.92 (1H, m, $CH(CH_3)_2$), 1.65 (3H, s, $NHC(CH_3)_2$), 1.63 (3H, s, $NHC(CH_3)_2$), 1.59 (6H, s, $NHC(CH_3)_2$), 1.54 (6H, s, $NHC(CH_3)_2$), 1.36 (3H, s, $NHC(CH_3)_2$), 1.33 (3H, s, $NHC(CH_3)_2$), 1.32 (3H, s, $NHC(CH_3)$), 0.96 (3H, d, *J* = 6.0, $CH(CH_3)_2$), 0.93 (3H, s, $NHC(CH_3)_2$), 0.82 (3H, d, *J* = 6.0, $CH(CH_3)_2$), 0.71 (3H, s, $NHC(CH_3)_2$); **δ_C** (100 MHz, $CDCl_3$) 180.4 (CO_2H), 176.6 (CONH), 176.3 (CONH), 176.0 (CONH), 175.0 (CONH), 174.1 (CONH), 173.9 (CONH), 156.5 (CO_2Bn), 140.8, 140.7, 137.0, 136.7, 134.8, 130.1, 129.5, 128.6, 128.2, 128.1, 127.9, 127.7, 127.6, 127.4, 127.2, 127.1 (Ar), 69.6 ($ArCH_2C$), 66.9 ($PhCH_2$), 63.0 ($NHC(CH_3)$), 57.3 ($NHC(CH_3)_2$), 56.9 ($NHC(CH_3)_2$), 56.8 ($NHC(CH_3)_2$), 56.7 ($NHC(CH_3)_2$), 56.3 ($NHC(CH_3)_2$), 42.1 ($ArCH_2$), 34.8 ($ArCH_2$), 34.7 ($CH(CH_3)_2$), 27.5 ($NHC(CH_3)_2$), 27.4 ($NHC(CH_3)_2$), 27.0 ($NHC(CH_3)_2$), 26.7 ($NHC(CH_3)_2$), 26.4 ($NHC(CH_3)_2$), 24.1 ($NHC(CH_3)_2$), 23.3 ($NHC(CH_3)_2$), 22.8 ($NHC(CH_3)_2$), 22.6 ($NHC(CH_3)_2$), 21.7 ($NHC(CH_3)_2$), 17.3 ($CH(CH_3)_2$), 17.2 ($CH(CH_3)_2$) 16.00 ($NHC(CH_3)$); **ν_{max}/cm^{-1}** 3289w.br (O-H), 2982w (C-H), 1652s (C=O), 1527m (C-N); ***m/z*** (ESI^+) 949.6 ($[M+Na]^+$, 100%), 927.6 ($[M+H]^+$, 50%), HRMS (ESI^+) found $[M+Na]^+$ 948.4855 $[C_{50}H_{67}N_7O_{10}Na]^+$ requires 948.4847; **$[\alpha]_D^{20}$** = +72.0 (*c* 0.50; CH_3OH).

Cbz-L-αMv-Aib$_3$-Bip-Aib$_3$-D-αMv-NHMe – 4b

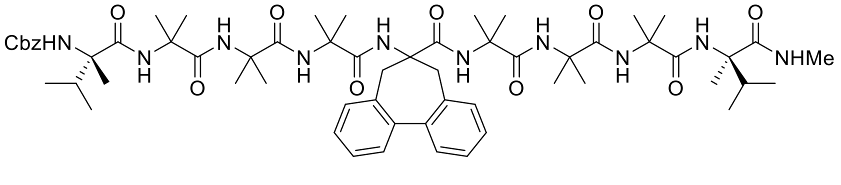

From **S34** (100 mg, 0.11 mmol), EDC.HCl (32 mg, 0.17 mmol), Et_3N (30 µL, 0.17 mmol) and **D-S20** (26 mg, 0.11 mmol) according to **General Procedure F**. Crude **4b** was purified by flash column chromatography twice (99:1 DCM:MeOH increasing to 19:1) to give the pure product as white powder (76 mg, 0.07 mmol, 61%). **R*f*** 0.39 (19:1 DCM:MeOH); **m.p.** 240-242 °C; **δ_H** (500 MHz, CD_2Cl_2/CD_3OD) 7.90-6.89 (18H, m, ArH, CONH), 5.16-4.90 (2H, m, $PhCH_2$), 3.08-2.49 (7H, m, $ArCH_2$, $NHCH_3$), 2.18-1.73 (2H, m, $CH(CH_3)_2$), 1.56-0.95 (42H, m, $NHC(CH_3)_2$, $NHC(CH_3)$), 0.93-0.76 (12H, m, $NHC(CH_3)_2$); **δ_C** (125 MHz, CD_2Cl_2/CD_3OD) 177.0 (CONH), 176.9 (CONH), 176.7 (CONH), 176.6 (CONH), 176.4 (CONH), 176.2 (CONH), 175.5 (CONH), 175.4 (CONH), 173.9 (CONH), 156.9 (CO_2Bn), 141.4, 141.3, 137.2, 135.5, 130.6, 130.3, 129.2, 128.9, 128.4, 128.3, 128.1, 127.9, 127.8 (Ar), 70.2 ($ArCH_2C$), 67.6 ($PhCH_2$), 63.5 ($NHC(CH_3)$), 57.7-56.8 ($NHC(CH_3)_2$), 36.8-34.7 ($ArCH_2$), 30.3 ($NHC(CH_3)$), 28.1-22.1 ($NHCH_3$, $NHC(CH_3)_2$, $NHC(CH_3)$), 19.3-16.3 ($CH(CH_3)_2$); **ν_{max}/cm^{-1}** 3296s.br (N-H), 2982w (C-H), 1651s (C=O), 1527m (C-N); ***m/z*** (ESI^+) 1159.7 ($[M+Na]^+$, 100%), HRMS (ESI^+) found $[M+Na]^+$ 1159.6530 $[C_{61}H_{88}N_{10}O_{11}Na]^+$ requires 1159.6526; **$[\alpha]_D^{20}$** = +4.8 (c = 0.50; CH_3OH).

N_3-Aib-Bip-Aib-D-αMv-NHMe – S35

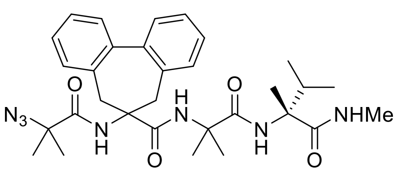

From **S26** (84 mg, 0.23 mmol), EDC.HCl (67 mg, 0.35 mmol), Et_3N (60 μL, 0.35 mmol) and **D-S20** (80 mg, 0.23 mmol) according to **General Procedure F**. Crude **S35** was purified by flash column chromatography (99:1 DCM:MeOH increasing to 19:1) to give the pure compound as a clear film (90 mg, 0.04 mmol, 57%). $\mathbf{R_f}$ 0.13 (97:3 DCM:MeOH); $\mathbf{\delta_H}$ (400 MHz, $CDCl_3$) 7.49-7.37 (5H, m, Ar*H*, CON*H*), 7.33 (2H, dd, *J* = 6.3, 6.3, Ar*H*), 7.24-7.14 (3H, m, Ar*H*, CON*H*), 6.90 (1H, s, CON*H*), 6.67 (1H, s, CON*H*), 3.32-3.09 (2H, m, ArCH_2), 3.10 (2H, d, *J* = 11.0, ArCH_2), 2.76 (3H, d, *J* = 3.2, NHCH_3), 2.59 (1H, m, ArCH_2), 2.14 (1H, m, C*H*(CH_3)$_2$), 1.57 (6H, s, N_3C(CH_3)$_2$), 1.48 (6H, s, NHC(CH_3)$_2$), 1.43 (3H, s, NHC(CH_3)), 0.90 (3H, d, *J* = 6.8, CH(CH_3)$_2$), 0.87 (3H, d, *J* = 6.8, CH(CH_3)$_2$); $\mathbf{\delta_C}$ (100 MHz, $CDCl_3$) 174.6 (*C*ONH), 173.2 (*C*ONH), 172.7 (*C*ONH), 171.7 (*C*ONH), 140.7, 140.5, 134.9, 133.6, 130.1, 129.7, 128.6, 128.5, 128.1, 128.1, 128.0, 127.9 (Ar), 69.8 (ArCH$_2$*C*), 64.3 (N_3*C*(CH_3)$_2$), 63.4 (NH*C*(CH_3)), 57.5 (NH*C*(CH_3)$_2$), 35.4 (*C*H(CH_3)$_2$), 31.1 (Ar*C*H$_2$), 27.3 (NHC(*C*H$_3$)$_2$), 26.6 (NH*C*H$_3$), 24.6 (NHC(*C*H$_3$)$_2$), 24.5 (N_3C(*C*H$_3$)$_2$), 24.0 (N_3C(*C*H$_3$)$_2$), 17.9 (NHC(*C*H$_3$)), 17.6 (CH(*C*H$_3$)$_2$), 17.2 (CH(*C*H$_3$)$_2$); $\mathbf{\nu_{max}/cm^{-1}}$ 3346m.br (N-H), 2972w (C-H), 2113m (N_3), 1664s (C=O), 1512m (C-N); ***m/z*** (ESI^+) 576.3 ($[M+H]^+$, 100%), HRMS (ESI^+) found $[M+Na]^+$ 598.3099 $[C_{31}H_{41}N_7O_4]^+$ requires 598.3118; $\mathbf{[\alpha]_D^{20}}$ = −72.8 (*c* 1.00; CH_3OH).

H-Aib-Bip-Aib-D-αMv-NHMe – S36

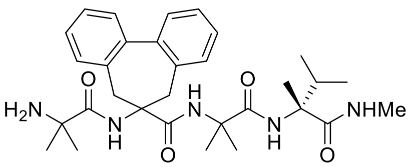

From **S35** (90 mg, 0.16 mmol) according to **General Procedure B**. **S36** was obtained as a clear oil (88 mg, 0.16 mmol, *quant.*). $\mathbf{R_f}$ 0.06 (19:1 DCM:MeOH); $\mathbf{\delta_H}$ (400 MHz, CD_3OD) 7.47-7.38 (4H, m, Ar*H*), 7.36-7.26 (2H, m, Ar*H*), 7.23-7.08 (2H, m, Ar*H*), 3.01-2.56 (7H, m, ArCH_2, NHCH_3), 2.20 (1H, m, C*H*(CH_3)$_2$), 1.56-1.42 (6H, m, H_2NC(CH_3)$_2$, NHC(CH_3)$_2$), 1.41 (3H, s, H_2NC(CH_3)$_2$), 1.40 (3H, s, NHC(CH_3)), 1.39 (3H, s, NHC(CH_3)), 1.02 (3H, d, *J* = 6.6, CH(CH_3)$_2$), 0.91 (3H, d, *J* = 6.6, CH(CH_3)$_2$); $\mathbf{\delta_C}$ (100 MHz, $CDCl_3$) 174.9 (*C*ONH), 173.2 (*C*ONH), 173.0 (*C*ONH), 172.9 (*C*ONH), 140.3, 130.7, 128.5, 128.4, 128.3, 128.2, 128.0, 128.0, 127.9, 127.9, 127.8, 127.8 (Ar), 69.6 (ArCH$_2$*C*), 63.3 (NH*C*(CH_3)), 57.3 (NH*C*(CH_3)$_2$), 55.1 (H_2N*C*(CH_3)$_2$), 35.5 (*C*H(CH_3)$_2$), 29.8 (H_2NC(*C*H$_3$)$_2$), 29.2 (NHC(*C*H$_3$)$_2$), 26.7 (NH*C*H$_3$), 18.6 (NHC(*C*H$_3$)), 17.7 (CH(*C*H$_3$)$_2$), 17.6 (CH(*C*H$_3$)$_2$); $\mathbf{\nu_{max}/cm^{-1}}$ 3328m.br (N-H), 2971w (C-H), 1667s (C=O), 1512m (C-N); ***m/z*** (ESI^+) 550.4 ($[M+H]^+$, 100%), HRMS (ESI^+) found $[M+Na]^+$ 572.3200 $[C_{31}H_{43}N_5O_4Na]^+$ requires 572.3213; $\mathbf{[\alpha]_D^{20}}$ = −58.4 (*c* 1.00; CH_3OH).

Cbz-L-αMv-Aib_4-OH – S37

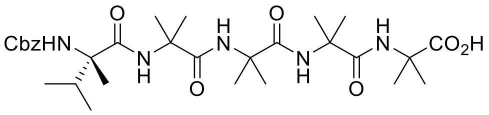

Prepared as previously reported.[4]

Cbz-L-αMv-Aib$_5$-Bip-Aib-D-αMv-NHMe – 4c

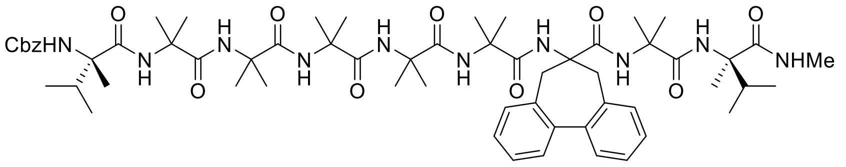

From **S37** (97 mg, 0.16 mmol), EDC.HCl (46 mg, 0.24 mmol), Et_3N (40 µL, 0.24 mmol) and **S36** (88 mg, 0.16 mmol) according to **General Procedure F**. Crude **4c** was purified by flash column chromatography (99:1 DCM:MeOH increasing to 19:1) to give the pure compound as a white solid (34 mg, 0.03 mmol, 19%). $\mathbf{R_f}$ 0.32 (19:1 DCM:MeOH); **m.p.** 190-192 °C; δ_H (500 MHz, $CDCl_3$) 7.77-6.89 (21H, m, ArH, CON*H*), 6.68 (1H, s, CON*H*), 5.86 (1H, s, CON*H*), 5.13-4.93 (2H, m, PhC*H*$_2$), 3.26-2.51 (7H, m, ArC*H*$_2$, NHC*H*$_3$), 2.34-1.88 (2H, m, C*H*(CH_3)$_2$), 1.81-1.09 (42H, m, NHC(C*H*$_3$)$_2$, NHC(C*H*$_3$)), 0.95-0.81 (12H, m, NHC(C*H*$_3$)$_2$); δ_C (125 MHz, CD_2Cl_2/CD_3OD) 176.0 (*C*ONH), 175.8 (*C*ONH), 175.8 (*C*ONH), 175.6 (*C*ONH), 175.1 (*C*ONH), 174.8 (*C*ONH), 174.3 (*C*ONH), 173.4 (*C*ONH), 156.3 (*C*O$_2$Bn), 140.7, 140.7, 136.5, 130.2, 129.8, 128.6, 128.3, 127.8, 127.3, 127.3 (Ar), 69.7 (ArCH$_2$*C*), 67.0 (Ph*C*H$_2$), 63.2 (NH*C*(CH_3)), 62.9 (NH*C*(CH_3)), 57.3-56.2 (NH*C*(CH_3)$_2$), 36.0-33.7 (Ar*C*H$_2$), 31.9 (NHC(*C*H$_3$)), 29.8-21.7 (NH*C*H$_3$, NHC(*C*H$_3$)$_2$, NHC(*C*H$_3$)), 18.3-16.6 (CH(*C*H$_3$)$_2$); ν_{max}/cm^{-1} 3294m.br (N-H), 2983m (C-H), 1652s (C=O), 1530m (C-N); ***m/z*** (ESI$^+$) 1159.7 ([M+Na]$^+$, 100%), HRMS (ESI$^+$) found [M+Na]$^+$ 1159.6530 [$C_{61}H_{88}N_{10}O_{11}Na$]$^+$ requires 1159.6526; $[\alpha]_D^{20}$ = −7.4 (*c* 0.50; CH_3OH).

H-Aib$_3$-O^{t}Bu – S38

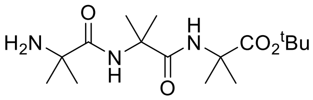

Prepared as previously reported.[5]

Cbz-Gly-Aib$_3$-O^{t}Bu – S39

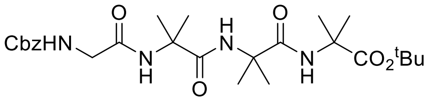

From Cbz-Gly-OH (211 mg, 1.01 mmol), NMM (0.29 mL, 1.58 mmol), ^{i}BuCOCl (0.13 mL, 1.01 mmol) and **S38** (500 mg, 1.52 mmol) according to **General Procedure E**. Crude **S39** was purified by flash column chromatography (99:1 DCM:MeOH increasing to 19:1) to give the pure product as a white powder (467 mg, 0.90 mmol, 89%). $\mathbf{R_f}$ 0.31 (19:1 DCM:MeOH); **m.p.** 139-141 °C; δ_H (500 MHz, $CDCl_3$) 7.38-7.30 (5H, m, Ph*H*) 7.05 (1H, s, CON*H*), 6.80 (1H, s, CON*H*), 6.54 (1H, s, CON*H*), 5.72 1H, s, br, N*H*CO$_2$), 5.13 (2H, s, PHC*H*$_2$), 3.77 (2H, d, *J* = 5.1, NHC*H*$_2$), 1.48 (6H, s, NHC(C*H*$_3$)$_2$), 1.46 (6H, s, NHC(C*H*$_3$)$_2$), 1.45 (6H, s, NHC(C*H*$_3$)$_2$), 1.43 (9H, s, C(C*H*$_3$)$_3$); δ_C (125 MHz, $CDCl_3$) 174.0 (*C*ONH), 173.3 (*C*ONH), 172.5 (*C*ONH), 169.2 (*C*O$_2$C(CH_3)$_3$), 157.1 (*C*O$_2$Bn), 136.0, 128.6, 128.5, 128.2 (Ar), 80.7

(C(CH_3)$_3$), 67.5 (PhCH_2), 57.2 (NHC(CH_3)$_2$), 56.8 (NHC(CH_3)$_2$), 53.5 (NHC(CH_3)$_2$), 45.5 (NHCH_2), 27.9 (C(CH_3)$_3$), 25.2 (NHC(CH_3)$_2$), 25.1 (NHC(CH_3)$_2$), 24.5 (NHC(CH_3)$_2$); **ν_{max}/cm^{-1}** 3323m.br (N-H), 2982m (C-H), 1672s (C=O), 1531s (C-N); ***m/z*** (ESI$^+$) 543.7 ([M+Na]$^+$, 100%), HRMS (ESI$^+$) found [M+Na]$^+$ 543.2800 [$C_{26}H_{40}N_4O_7Na$]$^+$ requires 543.2789.

Cbz-Gly-Aib$_3$-OH – S40

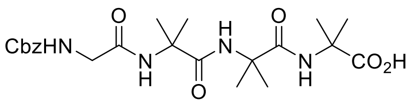

From **S39** (467 mg, 0.90 mmol) according to **General Procedure D**. Crude **S40** was purified by flash column chromatography (99:1 DCM:MeOH increasing to 19:1) to give the pure product as a white powder (361 mg, 0.78 mmol, 87%). **R_f** 0.30 (19:1 DCM:MeOH); **m.p.** 193-195 °C; **δ_H** (400 MHz, CD_3OD) 8.31 (1H, s, br, CON*H*), 7.56 (1H, s, br, CON*H*), 7.42 (1H, s, br, CON*H*), 7.35-7.24 (5H, m, Ph*H*) 5.07 (2H, s, PHCH_2), 3.68 (2H, d, *J* = 5.1, NHCH_2), 1.44 (6H, s, NHC(CH_3)$_2$), 1.40 (6H, s, NHC(CH_3)$_2$), 1.38 (6H, s, NHC(CH_3)$_2$); **δ_C** (100 MHz, CD_3OD) 178.2 (CO$_2$H), 176.4 (CONH), 175.9 (CONH), 172.3 (CONH), 159.3 (CO$_2$Bn), 138.1, 129.5, 129.1, 128.9 (Ar), 68.0 (PhCH_2), 57.8 (NHC(CH_3)$_2$), 57.7 (NHC(CH_3)$_2$), 56.9 (NHC(CH_3)$_2$), 45.2 (NHCH_2), 25.4 (C(CH_3)$_3$), 25.2 (NHC(CH_3)$_2$); **ν_{max}/cm^{-1}** 2986m (C-H), 2481m.br (O-H), 1652s (C=O), 1429s (C-N); ***m/z*** (ESI$^+$) 487.4 ([M+Na]$^+$, 100%), HRMS (ESI$^+$) found [M+Na]$^+$ 487.2160 [$C_{26}H_{40}N_4O_7Na$]$^+$ requires 487.2163.

Cbz-Gly-Aib$_4$-L/D-αMv-NHMe – S41

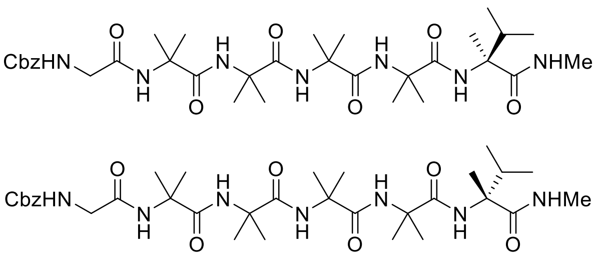

From **S40** (180 mg, 0.54 mmol), EDC.HCl (156 mg, 0.82 mmol), DIPEA (0.14 mL, 0.82 mmol) and **S20** (124 mg, 0.54 mmol) according to **General Procedure F**. Crude **S41** was purified by flash column chromatography (99:1 DCM:MeOH increasing to 19:1) to give the pure product as a white powder (137 mg, 0.25 mmol, 46%). **R_f** 0.09 (19:1 DCM:MeOH); **m.p.** 242-244 °C; **δ_H** (400 MHz, $CDCl_3$) 8.36 (1H, s, CON*H*), 7.99 (1H, s, CON*H*), 7.77 (1H, s, CON*H*), 7.72 (1H, s, CON*H*), 7.40-7.25 (6H, m, Ph*H*, CON*H*), 5.14 (1H, d, *J* = 12.5, PHCH_2), 5.08 (1H, d, *J* = 12.5, PHCH_2), 3.80 (1H, d, *J* = 16.6, NHCH_2), 3.67 (1H, d, *J* = 16.6, NHCH_2), 2.74 (3H, s, NHCH_3), 2.13 (1H, sep, *J* = 6.8, C*H*(CH_3)$_2$), 1.46 (3H, s, NHC(CH_3)$_2$), 1.43 (3H, s, NHC(CH_3)$_2$), 1.42 (3H, s, NHC(CH_3)$_2$), 1.41 (3H, s, NHC(CH_3)$_2$), 1.40 (3H, s, NHC(CH_3)$_2$), 1.39 (3H, s, NHC(CH_3)$_2$), 1.38 (3H, s, NHC(CH_3)), 1.01 (3H, d, *J* = 6.8, CH(CH_3)$_2$), 0.93 (3H, d, *J* = 6.8, CH(CH_3)$_2$); **δ_C** (125 MHz, CD_3OD) 150.5 (CONHCH$_3$), 179.9 (CONH), 179.5 (CONH), 179.4 (CONH), 179.2 (CONH), 174.7 (CH$_2$$C$ONH), 161.9z ($CO_2$Bn), 140.7, 132.1, 131.7, 131.3 (Ar), 70.3 (NHC(CH_3)), 67.0 (PhCH_2), 60.7 (NHC(CH_3)$_2$), 60.4 (NHC(CH_3)$_2$), 60.3 (NHC(CH_3)$_2$), 60.1 (NHC(CH_3)$_2$), 47.8 (NHCH_2), 39.4 (CH(CH_3)$_2$), 30.0 (NHC(CH_3)$_3$), 29.6 (NHC(CH_3)$_2$), 29.3 (NHC(CH_3)$_2$), 29.0 (NHCH_3), 28.8 (NHC(CH_3)$_2$) 26.8

(NHC(CH_3)$_2$), 26.7 (NHC(CH_3)$_2$), 26.5 (NHC(CH_3)$_2$), 26.4 (NHC(CH_3)$_2$), 26.3 (NHC(CH_3)), 20.9 (CH(CH_3)$_2$, 20.3 (CH(CH_3)$_2$); **ν_{max}/cm^{-1}** 2983m (C-H), 2472m.br (N-H), 1646s (C=O), 1413m (C-N); ***m/z*** (ESI$^+$) 699.6 ([M+Na]$^+$, 100%), HRMS (ESI$^+$) found [M+H]$^+$ 676.4019 [$C_{33}H_{54}N_7O_8$]$^+$ requires 676.4028; (**L**) **[α]$_D^{20}$** = +30.8 (*c* 1.00; CH_3OH), (**D**) **[α]$_D^{20}$** = −26.3 (*c* 0.99, CH_3OH).

H-Gly-Aib$_4$-L/D-αMv-NHMe – S42

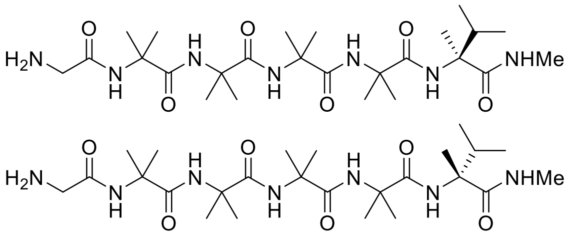

From **S41** (137 mg, 0.21 mmol) according to **General Procedure B**. **S42** was obtained as a white powder (105 mg, 0.19 mmol, 92%). **R$_f$** 0.00 (19:1 DCM:MeOH); **m.p.** 258-260 °C; **δ$_H$** (400 MHz, CD_3OD) 3.37-3.31 (2H, m, NHCH_2), 2.77 (3H, s, NHCH_3), 2.16 (1H, sep, *J* = 6.7, C*H*(CH_3)$_2$), 1.51-1.42 (24H, m, NHC(CH_3)$_2$), 1.39 (3H, s, NHC(CH_3)), 1.04 (3H, d, *J* = 6.7, CH(CH_3)$_2$), 0.96 (3H, d, *J* = 6.7, CH(CH_3)$_2$); **δ$_C$** (100 MHz, CD_3OD) 177.9 (*C*ONHCH$_3$), 177.3 (*C*ONH), 176.9 (*C*ONH), 176.7 (*C*ONH), 176.5 (*C*ONH), 174.5 (CH$_2$*C*ONH) 64.5 (NH*C*(CH_3)), 60.7 (NH*C*(CH_3)$_2$), 58.1 (NH*C*(CH_3)$_2$), 57.8 (NH*C*(CH_3)$_2$), 57.7 (NH*C*(CH_3)$_2$), 57.4 (NH*C*(CH_3)$_2$), 45.0 (NH*C*H$_2$), 36.9 (NH*C*H(CH_3)$_2$), 27.4 (NHC(CH_3)$_3$), 26.9 (NHC(CH_3)$_2$), 26.6 (NHC(CH_3)$_2$), 26.5 (NH*C*H$_3$), 26.2 (NHC(CH_3)-$_2$) 24.4 (NHC(CH_3)$_2$), 24.0 (NHC(CH_3)$_2$), 23.8 (NHC(CH_3)$_2$), 23.8 (NHC(CH_3)$_2$), 23.8 (NHC(CH_3)), 18.3 (CH(CH_3)$_2$, 17.7 (CH(CH_3)$_2$); **ν_{max}/cm^{-1}** 3378m.br (N-H), 2970m (C-H), 2476m.br (N-H), 1639s (C=O), 1424s (C-N); ***m/z*** (ESI$^+$) 564.4 ([M+Na]$^+$, 100%), HRMS (ESI$^+$) found [M+Na]$^+$ 564.3480 [$C_{25}H_{47}N_7O_6Na$]$^+$ requires 564.3480; (**L**) **[α]$_D^{20}$** = +19.6 (*c* 1.00; CH_3OH), (**D**) **[α]$_D^{20}$** = −22.4 (*c* 1.00; CH_3OH).

Cbz-L-αMv-Aib$_4$-Gly-Aib$_4$-L-αMv-NHMe – 5a

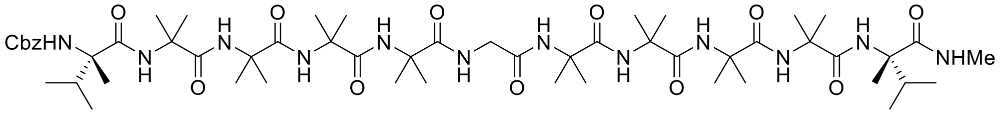

From **S37** (56 mg, 0.09 mmol), EDC.HCl (26 mg, 0.14 mmol), DIPEA (24 μL, 0.14 mmol) and **L-S42** (46 mg, 0.09 mmol) according to **General Procedure F**. Crude **5a** was purified by flash column chromatography (99:1 DCM:MeOH increasing to 19:1) to give the pure compound as a clear solid (26 mg, 0.02 mmol, 26%). **R$_f$** 0.15 (19:1 DCM:MeOH); **m.p.** 245-247 °C **δ$_H$** (400 MHz, $CDCl_3$) 8.19 (1H, t, *J* = 5.4, CON*H*), 7.91 (1H, s, CON*H*), 7.81 (1H, s, CON*H*), 7.78 (1H, s, CON*H*), 7.69 (1H, s, CON*H*), 7.61 (H, s, CON*H*), 7.51 (1H, s, CON*H*), 7.46-7.30 (7H, m, CON*H*, Ph*H*), 7.02 (1H, s, CON*H*), 6.64 (1H, s, br, CON*H*), 5.68 (1H, s, br, CON*H*), 5.09 (2H, ABq, $\Delta\delta_{AB}$ = 0.03, J_{AB} = 12.4, PhCH_2), 3.86 (1H, dd, *J* = 16.4, 5.0, NHCH_2), 3.67 (1H, dd, *J* = 16.4, 6.1, NHCH_2), 2.79 (3H, d, *J* = 4.5, NHCH_3), 2.13 (1H, sep, *J* = 6.7, C*H*(CH_3)$_2$), 1.99 (1H, m, C*H*(CH_3)$_2$), 1.48 (3H, s, NHC(CH_3)$_2$), 1.45 (12H, s, NHC(CH_3)$_2$), 1.44 (9H, s, NHC(CH_3)$_2$), 1.42 (6H, s, NHC(CH_3)$_2$), 1.41 (9H, s, NHC(CH_3)$_2$), 1.39 (6H, s, NHC(CH_3)$_2$), 1.37 (3H, s, NHC(CH_3)$_2$), 1.36 (3H, s, NHC(CH_3)), 1.35 (3H, s, NHC(CH_3)), 1.00-0.82 (12H,

m, CH(CH_3)$_2$); **δ$_C$** (100 MHz, $CDCl_3$) 177.9, 176.6, 176.3, 176.1, 175.8, 175.6, 175.2, 174.9, 174.6, 173.5, 171.0 (CONH), 156.5 (CO_2Bn), 136.3, 128.9, 128.8, 128.3 (Ar), 67.6 (PhCH_2), 63.4 (NHC(CH_3)), 63.3 (NHC(CH_3)), 57.1, 57.0, 56.9, 56.9, 56.7 (NHC(CH_3)$_2$), 45.5 (NHCH_2), 36.0, 35.6 (CH(CH_3)$_2$), 27.6 (NHCH_3), 27.5-26.9 (NHC(CH_3)$_2$), 23.0-22.8 (NHC(CH_3)$_2$, NHC(CH_3)), 17.9, 17.4, 17.3, 17.2 (CH(CH_3)$_2$); **ν_{max}/cm^{-1}** 3289m (N-H), 2984w (C-H), 1655s (C=O), 1535m (C-N); ***m/z*** (ESI$^+$) 1152.8 ([M+Na]$^+$, 100%), HRMS (ESI$^+$) found [M+H]$^+$ 1129.6974 [$C_{55}H_{93}N_{12}O_{13}$]$^+$ requires 1129.6980; **[α]$_D^{20}$** = +44.2 (*c* 0.95; CH_3OH).

Cbz-L-αMv-Aib$_4$-Gly-Aib$_4$-D-αMv-NHMe – 5b

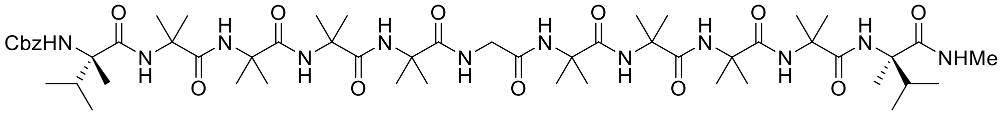

From **S37** (62 mg, 0.10 mmol), EDC.HCl (28 mg, 0.15 mmol), DIPEA (26 μL, 0.15 mmol) and **D-S42** (53 mg, 0.09 mmol) according to **General Procedure F**. Crude **5b** was purified by flash column chromatography (99:1 DCM:MeOH increasing to 19:1) to give the pure compound as a clear solid (26 mg, 0.02 mmol, 26%). **R$_f$** 0.18 (19:1 DCM:MeOH); **m.p.** 240-242 °C **δ$_H$** (400 MHz, $CDCl_3$) 8.06 (1H, s, br, CON*H*), 7.95 (1H, s, CON*H*), 7.76 (1H, s, CON*H*), 7.75 (1H, s, CON*H*), 7.62 (1H, s, CON*H*), 7.52 (2H, s, CON*H*), 7.40-7.29 (6H, m, CON*H*, Ph*H*), 7.22 (1H, s, CON*H*), 7.10 (1H, s, CON*H*), 6.94 (1H, s, br, CON*H*), 6.09 (1H, s, br, CON*H*), 5.13 (1H, d, *J* = 12.4, PhCH_2), 5.06 (1H, d, *J* = 12.4, PhCH_2), 3.95 (1H, dd, *J* = 16.5, 6.2, NHCH_2), 3.66 (1H, dd, *J* = 16.5, 4.9, NHCH_2), 2.77 (3H, d, *J* = 4.2, NHCH_3), 2.25 (1H, sep, *J* = 6.3, C*H*(CH_3)$_2$), 2.15 (1H, sep, *J* = 6.3, C*H*(CH_3)$_2$), 1.55 (3H, s, NHC(CH_3)$_2$), 1.53 (6H, s, NHC(CH_3)$_2$), 1.52 (3H, s, NHC(CH_3)$_2$), 1.49 (15H, s, NHC(CH_3)$_2$), 1.47 (6H, s, NHC(CH_3)$_2$), 1.45 (6H, s, NHC(CH_3)$_2$), 1.42 (3H, s, NHC(CH_3)$_2$), 1.41 (3H, s, NHC(CH_3)$_2$), 1.40 (3H, s, NHC(CH_3)$_2$), 1.39 (3H, s, NHC(CH_3)), 1.33 (3H, s, NHC(CH_3)), 1.02-0.90 (12H, m, CH(CH_3)$_2$); **δ$_C$** (100 MHz, $CDCl_3$) 177.3, 176.8, 176.4, 176.1, 175.9, 175.5, 175.1, 174.9, 174.8, 174.3, 171.1 (CONH), 156.5 (CO_2Bn), 136.4, 128.8, 128.6, 128.0 (Ar), 67.3 (PhCH_2), 63.1 (NHC(CH_3)), 62.9 (NHC(CH_3)), 57.2, 57.0, 57.0, 56.9, 56.8 (NHC(CH_3)$_2$), 45.1 (NHCH_2), 35.1, 34.6 (CH(CH_3)$_2$), 26.8-24.0 (NHCH_3, NHC(CH_3)$_2$, NHC(CH_3)), 18.0, 17.8, 17.5, 17.4 (CH(CH_3)$_2$); **ν_{max}/cm^{-1}** 3290m (N-H), 2984w (C-H), 1651s (C=O), 1529m (C-N); ***m/z*** (ESI$^+$) 1152.8 ([M+Na]$^+$, 100%), HRMS (ESI$^+$) found [M+H]$^+$ 1129.6980 [$C_{55}H_{93}N_{12}O_{13}$]$^+$ requires 1129.6980; **[α]$_D^{20}$** = −7.5 (*c* 0.40, CH_3OH).

Cbz-L-αMv-Aib$_2$-OH – S43

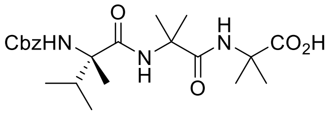

From **S11** (195 mg, 0.39 mmol) according to **General Procedure D**. Crude **S43** was purified by automatic purification system (99:1 DCM:MeOH increasing to 9:1) to give the pure product (121 mg, 0.28 mmol, 72%). **R$_f$** 0.21 (19:1 DCM:MeOH); **m.p.** 180-182 °C; **δ$_H$** (400 MHz, $CDCl_3$) 7.42 (1H, s, CON*H*) 7.38-7.32 (5H, m, Ph*H*),

6.46 (1H, s, CON*H*), 5.35 (1H, s, CON*H*), 5.16 (1H, d, *J* = 12.2, PhCH_2), 5.03 (1H, d, *J* = 12.2, PhCH_2), 1.96 (1H, sep, *J* = 6.9, C*H*(CH_3)$_2$), 1.55 (3H, s, NHC(CH_3)$_2$), 1.52 (3H, s, NHC(CH_3)$_2$), 1.48 (3H, s, NHC(CH_3)$_2$), 1.41 (3H, s, NHC(CH_3)$_2$), 1.26 (3H, s, NHC(CH_3)), 0.97 (3H, d, *J* = 6.9, CH(CH_3)$_2$), 0.92 (3H, d, *J* = 6.9, CH(CH_3)$_2$); **δ_C** (100 MHz, $CDCl_3$); 174.9 (*C*O_2H), 173.1 (*C*ONH), 169.3 (*C*ONH), 156.2 (*C*O_2Bn), 136.1, 128.9, 128.8, 128.4 (Ar), 67.6 (Ph*C*H_2), 63.3 (NH*C*(CH_3)), 57.5 (NH*C*(CH_3)$_2$), 57.0 (NH*C*(CH_3)$_2$), 35.7 (*C*H(CH_3)$_2$), 26.9 (*C*H(CH_3)$_2$), 25.4 (NH*C*H_3), 24.8 (NHC(*C*H_3)$_2$), 23.8 (NHC(*C*H_3)$_2$), 17.5 (NHC(*C*H_3)), 17.4 (CH(*C*H_3)$_2$), 17.2 (CH(*C*H_3)$_2$); **ν_{max}/cm^{-1}** 3350m.br (N-H, O-H), 2924m (C-H), 1705s (C=O), 1667s (C=O), 1510s (C-N), 1453m (C-O); ***m/z*** (ESI$^+$) 458.2 ([M+Na]$^+$, 100%), HRMS (ESI$^+$) found [M+Na]$^+$ 458.2260 [$C_{22}H_{33}N_3O_6Na$]$^+$ requires 458.2262; **[α]$_D^{20}$** = +12.0 (*c* 1.00; CH_3OH).

N_3-Aib$_3$-OH – S44

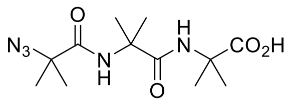

Prepared as previously reported.[8]

N_3-Aib$_4$-D-αMv-NHMe – S45

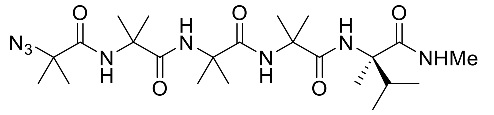

From **S44** (249 mg, 0.83 mmol), EDC.HCl (287 mg, 1.50 mmol), Et_3N (0.21 mL, 1.50 mmol) and **D-S20** (229 mg, 1.00 mmol) according to **General Procedure F**. Crude **S45** was purified by automatic purification system (99:1 DCM:MeOH increasing to 9:1) to give the pure product (260 mg, 0.51 mmol, 61%). **R_f** 0.21 (19:1 DCM:MeOH); **m.p.** 209-210 °C; **δ_H** (500 MHz, $CDCl_3$) 7.52 (1H, s, CON*H*) 7.25 (1H, d, *J* = 4.6, N*H*CH_3), 7.03 (1H, s, CON*H*), 6.89 (1H, s, CON*H*), 6.24 (1H, s, CON*H*), 2.77 (3H, d, *J* = 4.6, NHCH_3), 2.12 (1H, sep, *J* = 6.9, C*H*(CH_3)$_2$), 1.54 (3H, s, NHC(CH_3)$_2$), 1.52 (3H, s, NHC(CH_3)$_2$), 1.48 (3H, s, NHC(CH_3)$_2$), 1.48 (3H, s, NHC(CH_3)$_2$), 1.47 (3H, s, NHC(CH_3)$_2$), 1.46 (3H, s, NHC(CH_3)$_2$), 1.45 (3H, s, N_3C(CH_3)$_2$), 1.44 (3H, s, N_3C(CH_3)$_2$), 1.36 (3H, s, NHC(CH_3)), 0.97 (3H, d, *J* = 6.9, CH(CH_3)$_2$), 0.94 (3H, d, *J* = 6.9, CH(CH_3)$_2$); **δ_C** (125 MHz, $CDCl_3$); 175.1 (*C*ONH), 174.2 (*C*ONH), 174.1 (*C*ONH), 173.2 (*C*ONH), 172.8 (*C*ONH), 64.1 (NH*C*(CH_3)), 63.4 (N_3*C*(CH_3)$_2$), 57.3 (NH*C*(CH_3)$_2$), 57.1 (NH*C*(CH_3)$_2$), 56.8 (NH*C*(CH_3)$_2$), 35.8 (*C*H(CH_3)$_2$), 27.3 (NH*C*H_3), 27.2 (N_3C(*C*H_3)$_2$), 26.5 (NHC(*C*H_3)$_2$), 26.2 (NHC(*C*H_3)$_2$), 24.5 (NHC(*C*H_3)$_2$), 24.3 (N_3C(*C*H_3)$_2$), 23.7 (NHC(*C*H_3)$_2$), 23.5 (NHC(*C*H_3)$_2$), 23.4 (NHC(*C*H_3)$_2$), 17.9 (NHC(*C*H_3)), 17.7 (CH(*C*H_3)$_2$), 17.2 (CH(*C*H_3)$_2$); **ν_{max}/cm^{-1}** 3325m.br (N-H), 2982w (C-H), 2113m (N_3), 1661s (C=O), 1526m (C-N); ***m/z*** (ESI$^+$) 533.3 ([M+Na]$^+$, 100%), HRMS (ESI$^+$) found [M+H]$^+$ 511.3345 [$C_{23}H_{43}N_8O_5$]$^+$ requires 511.3351; **[α]$_D^{20}$** = −24.0 (*c* 1.00; CH_3OH).

H-Aib$_4$-D-αMv-NHMe – S46

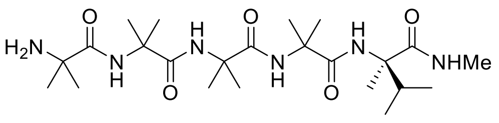

From **S45** (260 mg, 0.51 mmol) according to **General Procedure B**. **S46** was obtained as a white solid (247 mg, 0.51 mmol, *quant.*). **R_f** 0.05 (19:1 DCM:MeOH); **m.p.** 185-186 °C; **δ_H** (400 MHz, CD_3OD) 2.76 (3H, d, *J* = 4.1, NHC*H*$_3$), 2.16 (1H, sep, *J* = 6.8, C*H*(CH_3)$_2$), 1.49 (3H, s, H_2NC(C*H*$_3$)$_2$), 1.46 (3H, s, H_2NC(C*H*$_3$)$_2$), 1.45 (3H, s, NHC(C*H*$_3$)$_2$), 1.45 (3H, s, NHC(C*H*$_3$)$_2$), 1.44 (3H, s, NHC(C*H*$_3$)$_2$), 1.42 (3H, s, NHC(C*H*$_3$)$_2$), 1.38 (3H, s, NHC(C*H*$_3$)$_2$), 1.37 (6H, s, NHC(C*H*$_3$)$_2$), NHC(C*H*$_3$)), 1.04 (3H, d, *J* = 6.8, CH(C*H*$_3$)$_2$), 0.96 (3H, d, *J* = 6.8, CH(C*H*$_3$)$_2$); **δ_C** (100 MHz, CD_3OD); 179.3 (*C*ONH), 177.3 (*C*ONH), 177.0 (*C*ONH), 177.0 (*C*ONH), 176.1 (*C*ONH), 64.5 (NH*C*(CH_3)), 58.3 (NH*C*(CH_3)$_2$), 58.1 (NH*C*(CH_3)$_2$), 57.5 (NH*C*(CH_3)$_2$), 55.9 (H_2N*C*(CH_3)$_2$), 36.9 (*C*H(CH_3)$_2$), 28.3 (H_2C(*C*H$_3$)$_2$), 27.8 (H_2C(*C*H$_3$)$_2$), 27.4 (NH*C*H$_3$), 27.0 (NHC(*C*H$_3$)$_2$), 26.5 (NHC(*C*H$_3$)$_2$), 26.0 (NHC(*C*H$_3$)$_2$), 24.0 (NHC(*C*H$_3$)$_2$), 23.8 (NHC(*C*H$_3$)$_2$), 23.7 (NHC(*C*H$_3$)$_2$), 18.4 (NHC(*C*H$_3$)), 17.7 (CH(*C*H$_3$)$_2$), 17.4 (CH(*C*H$_3$)$_2$); **ν_{max}/cm^{-1}** 3315m.br (N-H), 2982w (C-H), 1655s (C=O), 1526m (C-N); ***m/z*** (ESI$^+$) 485.3 ([M+H]$^+$, 100%), 507.3 ([M+Na]$^+$, 60%), HRMS (ESI$^+$) found [M+Na]$^+$ 507.3269 [$C_{23}H_{44}N_6O_5Na$]$^+$ requires 507.3269; **$[\alpha]_D^{20}$** = −19.0 (*c* 1.00; CH_3OH).

(*S*)-2-amino-2-methylpropanoic-3-^{13}C acid hydrobromide – S47

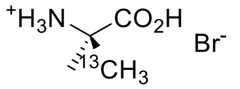

Prepared as previously reported.[9]

(*S*)-2-azido-2-methylpropanoic-3-^{13}C acid – S48

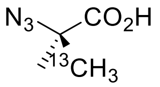

According to the procedure of Lee:[10] To a stirred solution of NaN_3 (2.05 g, 31.5 mmol) in water (5.25 mL) and DCM (8.75 mL) at 0 °C was added trifluoromethanesulfonic anhydride over 5 min. The reaction was stirred for a further 2 h. The reaction mixture was separated, and the aqueous further extracted with DCM (2 × 4.7 mL). The combined organic layers were washed with sat. aq. $NaHCO_3$ (15 mL). The resultant solution of trifluoromethanesulfonyl azide in DCM was never allowed to evaporate to dryness, but added as soon as possible to a stirred solution of **S47** (580 mg, 3.13 mmol), K_2CO_3 (1.09 g, 7.89 mmol) and $CuSO_4$ (80 mg, 0.32 mmol) in water (10.5 mL) and MeOH (20.5 mL). The reaction was stirred overnight then diluted with water (60 mL), acidified to pH 6 with conc. HCl and further diluted with phosphate buffer (250 mM, pH 6.2, 120 mL). This was then washed with EtOAc (4 × 50 mL). The aqueous was acidified to pH 1 with further conc. HCl and extracted with EtOAc (3 × 50 mL). The organic solvent was removed *in vacuo* to give **S48** as a yellow oil (241 mg, 1.85 mmol, 59%). **R_f** 0.29 (19:1 DCM:MeOH); **δ_H** (500 MHz, $CDCl_3$) 11.09 (1H, s, br, CO_2*H*), 1.53 (3H, d, *J* = 129.9, NHC(CH_3)(^{13}C*H*$_3$)), 1.53 (3H, d, *J* = 4.4, NHC(C*H*$_3$)(^{13}CH$_3$); **δ_C** (125 MHz, $CDCl_3$) 179.2 (*C*O$_2$H), 63.0 (d, *J* = 37.6, NH*C*(CH_3)(^{13}CH$_3$)), 24.4 (NHC(*C*H$_3$)(^{13}CH$_3$)); **ν_{max}/cm^{-1}** 2986m.br (O-H), 2093s (N_3), 1714m

(C=O), 1470m (C-O); ***m/z*** (ESI$^-$) 129.0 ([M-H]$^-$, 100%), HRMS (ESI$^-$) found [M-H]$^-$ 129.0498 [$C_3{}^{13}CH_6N_3O_2$]$^+$ requires 129.0499.

N_3-Aib*-Aib_4-D-αMv-NHMe – S49

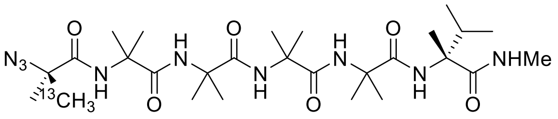

From **S48** (30 mg, 0.23 mmol), oxalyl chloride (30 μL, 0.35 mmol), **S46** (186 mg, 0.38 mmol) and Et_3N (45 μL, 0.38 mmol) according to **General Procedure A**. Crude **S49** was purified by flash column chromatography (99:1 DCM:MeOH increasing to 19:1) to give the pure compound as a white solid (75 mg, 0.13 mmol, 57%). **R_f** 0.19 (19:1 DCM:MeOH); **m.p.** 250-251 °C; **δ_H** (400 MHz, $CDCl_3$) 7.62 (1H, s, CON*H*), 7.40 (1H, s, CON*H*), 7.32 (1H, d, *J* = 5.0, N*H*CH_3), 7.03 (1H, s, CON*H*), 6.93 (1H, s, CON*H*), 6.31 (1H, s, CON*H*), 2.79 (3H, d, *J* = 4.7, NHC*H*$_3$), 2.15 (1H, sep, *J* = 6.6, C*H*(CH_3)$_2$), 1.72-1.36 (6H, m, N_3C(C*H*$_3$)(^{13}C*H*$_3$), 1.56-1.52 (3H, m, NHC(C*H*$_3$)$_2$), 1.50 (3H, s, NHC(C*H*$_3$)$_2$), 1.49 (3H, s, NHC(C*H*$_3$)$_2$), 1.48 (3H, s, NHC(C*H*$_3$)$_2$), 1.47 (3H, s, NHC(C*H*$_3$)$_2$), 1.47 (3H, s, NHC(C*H*$_3$)$_2$), 1.45 (3H, s, NHC(C*H*$_3$)$_2$), 1.44 (6H, s, NHC(C*H*$_3$)$_2$, NHC(C*H*$_3$)), 0.99 (3H, d, *J* = 6.6, CH(C*H*$_3$)$_2$), 0.95 (3H, d, *J* = 6.6, CH(C*H*$_3$)$_2$); **δ_C** (100 MHz, $CDCl_3$) 175.3 (*C*ONH), 175.3 (*C*ONH), 174.3 (*C*ONH), 173.5 (*C*ONH), 173.3 (*C*ONH), 173.2 (*C*ONH), 64.1 (d, *J* = 36.8, N_3*C*(CH_3)($^{13}CH_3$)), 63.4 (NH*C*(CH_3)), 57.2 (NH*C*(CH_3)$_2$), 57.0 (NH*C*(CH_3)$_2$), 57.0 (NH*C*(CH_3)$_2$), 56.9 (NH*C*(CH_3)$_2$), 35.8 (*C*H(CH_3)$_2$), 27.4 (NHC(*C*H$_3$)$_2$), 27.2 (NH*C*H$_3$), 26.8 (NHC(*C*H$_3$)$_2$), 26.5 (NHC(*C*H$_3$)$_2$), 26.1 (NHC(*C*H$_3$)$_2$), 24.5 (N_3C(^{13}*C*H$_3$)(CH_3)$_{minor}$), 24.4 (N_3C(CH_3)(^{13}*C*H$_3$)$_{major}$), 23.8 (NHC(*C*H$_3$)$_2$), 23.6 (NHC(*C*H$_3$)$_2$), 23.5 (NHC(*C*H$_3$)$_2$), 17.8 (NHC(*C*H$_3$)), 17.8 (CH(*C*H$_3$)$_2$), 17.3 (CH(*C*H$_3$)$_2$); **ν_{max}/cm^{-1}** 3317m.br (N-H), 2984w (C-H), 2113m (N_3), 1656s (C=O), 1521m (C-N); ***m/z*** (ESI$^+$) 619.4 ([M+Na]$^+$, 100%), HRMS (ESI$^+$) found [M+Na]$^+$ 619.3732 [$C_{26}{}^{1}H_{49}N_9O_6Na$]$^+$ requires 619.3732; **[α]$_D^{20}$** = −4.0 (*c* 1.00; CH_3OH).

H-Aib*-Aib_4-D-αMv-NHMe – S50

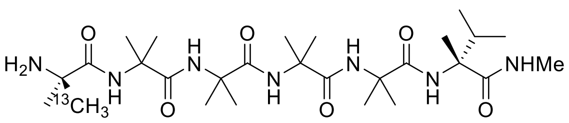

From **S49** (75 mg, 0.13 mmol) according to **General Procedure B**. **S50** was obtained as a white solid (58 mg, 0.10 mg, 77%). **R_f** 0.12 (19:1 DCM:MeOH); **m.p.** 229-230 °C; **δ_H** (400 MHz, CD_3OD) 2.74 (3H, s, NHC*H*$_3$), 2.13 (1H, sep, *J* = 6.7, C*H*(CH_3)$_2$), 1.66-1.30 (3H, m, H_2NC(CH_3)(^{13}C*H*$_3$), 1.48 (3H, s, H_2NC(C*H*$_3$)($^{13}CH_3$), 1.46 (12H, s, NHC(C*H*$_3$)$_2$), 1.44 (3H, s, NHC(C*H*$_3$)$_2$), 1.42 (3H, s, NHC(C*H*$_3$)$_2$), 1.42 (3H, s, NHC(C*H*$_3$)$_2$), 1.40 (3H, s, NHC(C*H*$_3$)$_2$), 1.40 (3H, s, NHC(C*H*$_3$)), 1.01 (3H, d, *J* = 6.7, CH(C*H*$_3$)$_2$), 0.94 (3H, d, *J* = 6.7, CH(C*H*$_3$)$_2$); **δ_C** (100 MHz, CD_3OD) 177.8 (*C*ONH), 177.3 (*C*ONH), 177.3 (*C*ONH), 177.0 (*C*ONH), 176.5 (*C*ONH), 176.3 (*C*ONH), 64.6 (NH*C*(CH_3)), 58.1 (NH*C*(CH_3)$_2$), 58.0 (H_2N*C*($^{13}CH_3$)(CH_3)), 57.8 (NH*C*(CH_3)$_2$), 57.8 (NH*C*(CH_3)$_2$), 57.8 (NH*C*(CH_3)$_2$), 36.9 (*C*H(CH_3)$_2$), 27.4 (NHC(*C*H$_3$)$_2$), 27.2 (NH*C*H$_3$), 27.0 (NHC(*C*H$_3$)$_2$), 26.6 (H_2NC(CH_3)(^{13}*C*H$_3$)$_{major}$), 26.4 (NHC(^{13}*C*H$_3$)(CH_3)$_{minor}$), 25.8 (NHC(*C*H$_3$)$_2$), 25.6 (NHC(*C*H$_3$)$_2$), 24.5 (NHC(*C*H$_3$)$_2$), 23.9 (NHC(*C*H$_3$)$_2$),

23.8 (NHC(*C*H_3)$_2$), 23.3 (NHC(*C*H_3)$_2$), 18.3 (NHC(*C*H_3)), 17.7 (CH(*C*H_3)$_2$), 17.5 (CH(*C*H_3)$_2$); **ν_{max}/cm^{-1}** 3314m.br (N-H), 2982w (C-H), 1653s (C=O), 1526m (C-N); ***m/z*** (ESI$^+$) 593.4 ([M+Na]$^+$, 100%), HRMS (ESI$^+$) found [M+Na]$^+$ 593.3824 [$C_{26}{}^{13}CH_{51}N_7O_6Na$]$^+$ requires 593.3827; **[α]$_D$^{20}** = −32.0 (*c* 1.00; CH_3OH).

Cbz-L-αMv-Aib$_2$-Aib*-Aib$_4$-D-αMv-NHMe – 6a

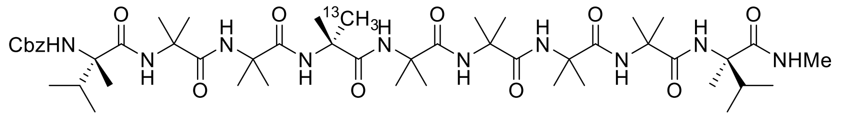

From **S43** (53 mg, 0.12 mmol), EDC.HCl (35 mg, 0.18 mmol), Et_3N (26 μL, 0.18 mmol) and **S50** (58 mg, 0.10 mmol) according to **General Procedure F**. Crude **6a** was purified by automatic purification system (99:1 DCM:MeOH increasing to 9:1) to give the pure product as a white solid (54 mg, 0.05 mmol, 50%). **R$_f$** 0.18 (19:1 DCM:MeOH); **m.p.** >250 °C; **δ$_H$** (500 MHz, $CDCl_3$) 7.75 (1H, s, CON*H*) 7.72 (1H, s, CON*H*), 7.69 (1H, s, CON*H*), 7.66 (1H, s, CON*H*), 7.62 (1H, s, CON*H*), 7.49 (1H, s, CON*H*), 7.39-7.30 (6H, m, Ph*H*, CON*H*), 7.08 (1H, s, CON*H*), 6.71 (1H, s, CON*H*), 5.86 (1H, s, CON*H*), 5.09 (2H, ABq, $\Delta\delta_{AB}$ = 0.04, J_{AB} = 11.7, PhC*H*$_2$), 2.78 (3H, d, *J* = 4.6, NHC*H*$_3$), 2.33 (1H, m, C*H*(CH_3)$_2$), 2.17 (1H, m. C*H*(CH_3)$_2$), 1.51 (9H, s, NHC(C*H*$_3$)$_2$), 1.48 (9H, s, NHC(C*H*$_3$)$_2$), 1.48 (6H, s, NHC(C*H*$_3$)$_2$), 1.46 (7H, s, NHC(C*H*$_3$)$_2$, NHC($^{13}CH_3$) (C*H*$_3$)$_{minor}$), 1.46 (2H, d, *J* = 125.4, NHC(C*H*$_3$)(^{13}C*H*$_3$)$_{major}$), 1.43 (3H, s, NHC(C*H*$_3$)$_2$), 1.42 (3H, s, NHC(C*H*$_3$)$_2$), 1.41 (2H, s, NHC(C*H*$_3$)($^{13}CH_3$)$_{major}$), 1.41 (1H, d, *J* = 125.4, NHC(^{13}C*H*$_3$)(CH_3)$_{minor}$), 1.41 (3H, s, NHC(C*H*$_3$), 1.34 (3H, s, NH(C*H*$_3$)), 1.00-0.91 (12H, m, CH(C*H*$_3$)$_2$); **δ$_C$** (125 MHz, $CDCl_3$); 176.3 (*C*ONH), 175.9 (*C*ONH), 175.8 (*C*ONH), 175.8 (*C*ONH), 175.6 (*C*ONH), 175.5 (*C*ONH), 174.7 (*C*ONH), 174.5 (*C*ONH), 173.8 (*C*ONH), 156.3 (*C*O$_2$Bn), 136.1, 128.9, 128.7, 128.2 (Ar), 67.5 (Ph*C*H$_2$), 63.1 (NH*C*(CH_3)), 62.8 (NH*C*(CH_3)), 57.3 (NH*C*(CH_3)$_2$), 56.9 (NH*C*(CH_3)$_2$), 56.9 (NH*C*(CH_3)$_2$), 56.9 (NH*C*(CH_3)$_2$), 56.8 (NH*C*(CH_3)$_2$), 56.7 (NH*C*(CH_3)$_2$), 56.6 (NH*C*(CH_3)$_2$), 34.8-34.6 (*C*H(CH_3)$_2$), 26.9 (NHC(*C*H$_3$)$_2$), 26.5 (NH*C*H$_3$), 26.2 (NHC(*C*H$_3$)$_2$), 25.8-24.1 (NHC(*C*H$_3$)$_2$), NHC($^{13}CH_3$)), 18.4 (NHC(*C*H$_3$)), 18.0 (NHC(*C*H$_3$)), 17.9 (CH(*C*H$_3$)$_2$), 17.8 (CH(*C*H$_3$)$_2$), 17.7 (CH(*C*H$_3$)$_2$), 17.5 (CH(*C*H$_3$)$_2$); **ν_{max}/cm^{-1}** 3293m.br (N-H), 2981w (C-H), 1656s (C=O). 1534m (C-N); ***m/z*** (ESI$^+$) 1010.6 ([M+Na]$^+$, 100%), HRMS (ESI$^+$) found [M+Na]$^+$ 1010.6070 [$C_{48}{}^{13}CH_{82}N_{10}O_{11}Na$]$^+$ requires 1010.6090; **[α]$_D$$^{21.5}$** = −0.2 (*c* 0.25; CH_3OH).

N_3-Aib*(50%)-Aib-D-αMv-NHMe – S51

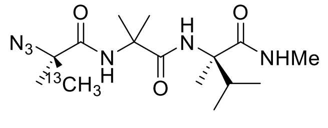

From **S48** (73 mg, 0.56 mg) and **S6** (72 mg, 0.56 mmol), oxalyl chloride (0.10 mL, 1.22 mmol), **D-S20** (254 mg, 1.11 mmol) and Et_3N (0.16 mL, 1.11 mmol) according to **General Procedure A**. Crude **S51** was purified by flash column chromatography (99:1 DCM:MeOH increasing to 19:1) to give the pure compound as a white solid (217 mg, 0.64 mmol, 57%). **R$_f$** 0.53 (19:1 DCM:MeOH); **m.p.** 125-127 °C; **δ$_H$** (400 MHz, $CDCl_3$) 7.13 (1H, d, *J* = 4.5, N*H*CH$_3$), 7.06 (1H, s, CON*H*), 6.22 (1H, s, CON*H*), 2.71 (3H, d, *J* = 4.5, NHC*H*$_3$), 2.10 (1H,

sep, J = 6.9, CH(CH$_3$)$_2$), 1.51 (3H, s, NHC(CH_3)$_2$), 1.47 (7.5H, s, NHC(CH_3)$_2$, NHC(CH_3), N$_3$C(CH_3)$_2$), 1.47 (1.5H, d, J = 129.7, N$_3$C(^{13}CH_3)(CH$_3$)), 1.43 (3H, s, N$_3$(^{13}CH$_3$)(CH_3), N$_3$C(CH_3)$_2$), 0.86 (3H, d, J = 6.9, CH(CH_3)$_2$), 0.81 (3H, d, J = 6.9, CH(CH_3)$_2$); **δ_C** (100 MHz, CDCl$_3$) 173.5 (CONH), 173.0 (CONH), 172.5 (CONH), 64.0 (N$_3$$C$(CH$_3$)$_2$), 64.0 (d, J = 36.7, N$_3$$C$(^{13}CH$_3$)(CH$_3$)), 63.0 (NH$C$(CH$_3$)), 57.4 (NH$C$(CH$_3$)$_2$), 35.6 ($C$H(CH$_3$)$_2$), 26.3 (NH$CH_3$), 24.2 (N$_3$C(^{13}CH$_3$)($CH_3$)), 24.0 (NHC($CH_3$)$_2$), 19.7 (NHC($CH_3$)) 17.4 (CH($CH_3$)$_2$), 16.7 (CH($CH_3$)$_2$); **$\nu_{max}$/cm^{-1}** 3358m.br (N-H), 2973m (C-H), 2110s (N$_3$), 1651s (C=O), 1500s (C-N); ***m/z*** (ESI$^+$) 341.2 and 342.2 ([M+Na]$^+$, 100%), HRMS (ESI$^+$) found [M+Na]$^+$ 363.2133 and 364.2147 [$C_{15}H_{28}N_6O_3Na$]$^+$ and [$C_{14}{}^{13}CH_{28}N_6O_3Na$]$^+$ require 363.2121 and 364.2154; **[α]$_D$^{20}** = −16.8 (c 1.10; CH$_3$OH).

H-Aib*(50%)-Aib-D-αMv-NHMe – S52

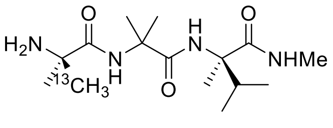

From **S51** (80 mg, 0.24 mmol) according to **General Procedure B**. **S52** was obtained as a clear film (77 mg, 0.24 mmol, *quant.*). **R$_f$** 0.05 (EtOAc); **m.p.** 203-205 °C; **δ_H** (400 MHz, CD$_3$OD) 2.83-2.80 (3H, m, NHCH_3), 2.11 (1H, sep, J = 6.7, CH(CH$_3$)$_2$), 1.57 (3H, s, NHC(CH_3)$_2$), 1.55 (3H, s, NHC(CH_3)$_2$), 1.51 (3H, s, NHC(CH_3)), 1.41 (4.5H, s, H$_2$NC(CH_3)$_2$, H$_2$NC(^{13}CH$_3$)(CH_3)), 1.41 (3H, d, J = 129.4, H$_2$NC(^{13}CH_3)(CH$_3$)), 1.03-1.01 (3H, m, CH(CH_3)$_2$), 1.01-0.99 (3H, m, CH(CH_3)$_2$); **δ_C** (100 MHz, CD$_3$OD) 180.5 (CONH), 175.7 (CONH), 175.7 (CONH), 64.1 (NHC(CH$_3$)), 57.9 (NHC(CH$_3$)$_2$), 55.7 (H$_2$NC(CH$_3$)$_2$), 55.7 (d, J = 36.7, H$_2$NC(^{13}CH$_3$)(CH$_3$)), 36.9 (CH(CH$_3$)$_2$), 28.6 (H$_2$NC(^{13}CH$_3$)(CH$_3$), H$_2$NC(CH$_3$)$_2$), 28.4 (H$_2$NC(CH$_3$)(^{13}CH$_3$), H$_2$NC(CH$_3$)$_2$), 26.5 (NHCH$_3$), 26.0 (NHC(CH$_3$)$_2$), 24.3 (NHC(CH$_3$)$_2$), 19.6 (NHC(CH$_3$)) 17.7 (CH(CH$_3$)$_2$), 17.8 (CH(CH$_3$)$_2$); **ν_{max}/cm^{-1}** 3351m.br (N-H), 2930m (C-H), 1645s (C=O), 1503m (C-N); ***m/z*** (ESI$^+$) 315.2 and 316.3 ([M+H]$^+$, 100%), HRMS (ESI$^+$) found [M+Na]$^+$ 337.2206 and 338.2235 [$C_{15}H_{30}N_4O_3Na$]$^+$ and [$C_{14}{}^{13}CH_{30}N_4O_3Na$]$^+$ require 337.2216 and 338.2249; **[α]$_D$^{20}** = −19.2 (c 1.00; CH$_3$OH).

N$_3$-Aib-Aib*(50%)-Aib-D-αMv-NHMe – S53

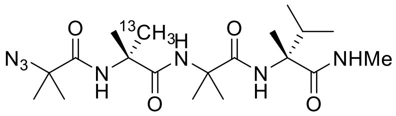

From **S6** (48 mg, 0.37 mmol), oxalyl chloride (0.03 mL, 0.38 mmol), **S52** (77 mg, 0.24 mmol) and Et$_3$N (0.05 mL, 0.37 mmol) according to **General Procedure A**. Crude **S53** was purified by flash column chromatography (99:1 DCM:MeOH increasing to 19:1) to give the pure compound as a white solid (47 mg, 0.11 mmol, 45%). **R$_f$** 0.38 (19:1 DCM:MeOH); **m.p.** 131-133 °C; **δ_H** (500 MHz, CDCl$_3$) 7.11 (1H, d, J = 4.4, NHCH$_3$), 6.88 (1H, s, CONH), 6.76 (1H, s, CONH), 6.39 (1H, s, CONH), 2.77 (3H, d, J = 4.4, NHCH_3), 2.14 (1H, sep, J = 6.2, CH(CH$_3$)$_2$), 1.53 (3H, m, NHC(CH_3)$_2$), 1.52 (3H, s, NHC(CH_3)$_2$,), 1.51-1.48 (4.5H, m, NHC(CH_3)$_2$, NHC(^{13}CH$_3$)(CH_3)), 1.49 (1.5H, d, J = 129.1, NHC(^{13}CH_3)(CH$_3$)), 1.47 (3H, m, NHC(CH_3)), 1.45 (N$_3$C(CH_3)$_2$), 1.39 (N$_3$C(CH_3)$_2$), 0.94 (3H, d, J = 6.2, CH(CH_3)$_2$), 0.92 (3H, d, J = 6.2, CH(CH_3)$_2$); **δ_C** (125 MHz, CDCl$_3$) 174.7 (CONH), 173.6 (CONH), 172.9 (CONH), 172.8 (CONH), 64.2 (N$_3$$C$(CH$_3$)$_2$), 63.5

(NHC(CH3)), 57.3 (NHC(CH3)2), 57.2 (NHC(CH3)2) 57.2 (d, *J* = 36.2, N3C(13CH3)(CH3)), 35.5 (CH(CH3)2), 27.3 (NHC(CH3)2), 26.5 (NHCH3), 26.1 (N3C(13CH3)major), 24.6 (N3C(CH3)2), 24.3 (N3C(CH3)2), 23.9 (NHC(13CH3)minor), 23.8 (NHC(CH3)2), 17.9 (NHC(CH3)) 17.6 (CH(CH3)2), 17.2 (CH(CH3)2); **ν_{max}/cm^{-1}** 3315m (N-H), 2977m (C-H), 2126m (N3), 1652s (C=O), 1517m (C-N); ***m/z*** (ESI$^+$) 448.3 and 449.3 ([M+Na]$^+$, 100%), HRMS (ESI$^+$) found [M+Na]$^+$ 448.2647 and 449.2686 [$C_{20}H_{35}N_7O_4Na$]$^+$ and [$C_{19}{}^{13}CH_{35}N_7O_4Na$]$^+$ require 448.2648 and 449.2682; **$[\alpha]_D^{20}$** = −23.6 (*c* 1.00; CH3OH).

H-Aib-Aib*(50%)-Aib-D-αMv-NHMe – S54

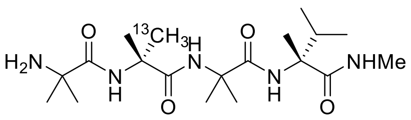

From **S53** (98 mg, 0.23 mmol) according to **General Procedure B**. **S54** was obtained as a clear film (81 mg, 0.20 mmol, 87%). **R*f*** 0.01 (19:1 DCM:MeOH); **m.p.** 203-205 °C; **δ_H** (500 MHz, CDCl3) 8.16 (1H, s, CONH), 7.17 (1H, d, *J* = 4.5, NHCH3), 6.96 (1H, s, CONH), 6.68 (1H, s, CONH), 2.77 (3H, d, *J* = 4.5, NHCH3), 2.15 (1H, sep, *J* = 6.8, CH(CH3)2), 1.70 (2H, s, br, NH2), 1.48 (3H, s, NHC(CH3)2, NHC(13CH3)(CH3)), 1.47 (3H, s, NHC(CH3)2), 1.46 (1.5H, s, NHC(CH3)2), 1.46 (1.5H, d, *J* = 128.9, NHC(13CH3)(CH3)), 1.43 (3H, s, NHC(CH3)2), 1.37 (3H, s, NHC(CH3)), 1.36 (3H, s, H2NC(CH3)2), 1.34 (3H, s, H2NC(CH3)2), 0.94 (3H, d, *J* = 6.8, CH(CH3)2), 0.92 (3H, d, *J* = 6.8, CH(CH3)2); **δ_C** (100 MHz, CDCl3) 178.5 (CONH), 174.9 (CONH), 174.3 (CONH), 173.2 (CONH), 63.5 (NHC(CH3)), 57.1 (NHC(CH3)2), 56.7 (NHC(CH3)2), 56.7 (d, *J* = 36.3, NHC(13CH3)(CH3)), 55.0 (H2NC(CH3)2), 35.6 (CH(CH3)2), 29.2 (H2NC(CH3)2), 28.9 (NHC(CH3)2, 27.4 (H2NC(CH3)2), 26.5 (NHC(CH3)2), 26.4 (NHCH3), 26.3 (NHC(13CH3)major), 24.0 (NHC(13CH3)major), 17.9 (NHC(CH3)) 17.7 (CH(CH3)2), 17.3 (CH(CH3)2); **ν_{max}/cm^{-1}** 3317m (N-H), 2967w (C-H), 1646s (C=O), 1525m (C-N); ***m/z*** (ESI$^+$) 400.4 and 401.4 ([M+Na]$^+$, 100%), HRMS (ESI$^+$) found [M+Na]$^+$ 422.2748 and 423.2775 [$C_{19}H_{37}N_5O_4Na$]$^+$ and [$C_{18}{}^{13}CH_{37}N_5O_4Na$]$^+$ require 422.2743 and 423.2777; **$[\alpha]_D^{20}$** = −17.2 (*c* 1.00; CH3OH).

N3-Aib*-Aib-Aib*(50%)-Aib-D-αMv-NHMe – S55

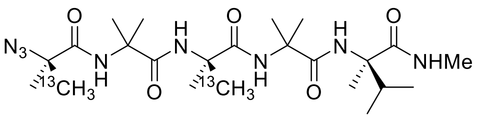

From **S48** (22 mg, 0.17 mmol), oxalyl chloride (15 µL, 0.18 mmol), **S54** (44 mg, 0.11 mmol) and Et3N (24 µL, 0.17 mmol) according to **General Procedure A**. Crude **S55** was purified by flash column chromatography (99:1 DCM:MeOH increasing to 19:1) to give the pure compound as a white solid (17 mg, 0.03 mmol, 30%). **R*f*** 0.14 (19:1 DCM:MeOH); **m.p.** 163-165 °C; **δ_H** (500 MHz, CDCl3) 7.50 (1H, s, CONH), 7.26 (1H, s, br, CONH), 6.95 (1H, s, CONH), 6.86 (1H, s, CONH), 6.15 (1H, s, CONH), 2.79 (3H, d, *J* = 4.7, NHCH3), 2.13 (1H, sep, *J* = 6.8, CH(CH3)2), 1.68-1.52 (5.5H, m, NHC(13CH3)(CH3)major, NHC(13CH3)(CH3)minor, NHC(CH3)2), 1.51-1.44 (17.5H, NHC(CH3)2, N3C(13CH3)(CH3)major, N3(13CH3)(CH3)minor, NHC(13CH3)(CH3)major, NHC(13CH3)(CH3)minor, NHC(13CH3)(CH3), N3(13CH3)(CH3)), 1.43-1.34 (4H, m, NHC(CH3), N3C(13CH3)(CH3)major,

$N_3(^{13}CH_3)(CH_3)_{minor}$), 0.97 (3H, d, *J* = 6.8, $CH(CH_3)_2$), 0.95 (3H, d, *J* = 6.8, $CH(CH_3)_2$); **δ_C** (125 MHz, $CDCl_3$) 175.1 (*C*ONH), 174.1 (*C*ONH), 174.1 (*C*ONH), 173.3 (*C*ONH), 172.7 (*C*ONH), 64.1 (d, *J* = 36.7, $N_3C(CH_3)_2$), 63.5 ($NHC(CH_3)$), 57.4 ($NHC(CH_3)_2$), 57.2 ($NHC(CH_3)_2$), 56.8 (0.5, d, *J* = 35.3, $NHC(^{13}CH_3)(CH_3)$), 56.8 (0.5, $NHC(CH_3)(CH_3)$), 35.8 ($CH(CH_3)_2$), 27.3 ($NHC(^{13}CH_3)(CH_3)_{minor}$), 26.5 ($NHCH_3$), 26.3 ($NHC(CH_3)_2$), 25.3 ($NHC(CH_3)_2$), 24.6 ($N_3C(^{13}CH_3)(CH_3)_{minor}$), 24.3 ($N_3C(^{13}CH_3)(CH_3)_{major}$), 23.8 ($NHC(CH_3)_2$), 23.6 ($NHC(CH_3)_2$), 23.5 ($NHC(^{13}CH_3)(CH_3)_{minor}$), 18.1 ($NHC(CH_3)$) 17.7 ($CH(CH_3)_2$), 17.2 ($CH(CH_3)_2$); **$\nu_{max}$/cm^{-1}** 3331m.br (N-H), 29761 (C-H), 2112s (N_3), 1656s (C=O), 1526m (C-N); ***m/z*** (ESI^+) 512.5 and 513.5 ($[M+H]^+$, 100%), HRMS (ESI^+) found $[M+Na]^+$ 534.3212 and 535.3246 $[C_{22}{}^{13}CH_{42}N_8O_5Na]^+$ and $[C_{21}{}^{13}C_2H_{41}N_8O_5Na]^+$ require 534.3209 and 535.3243; **$[\alpha]_D^{20}$** = −19.2 (*c* 0.50; CH_3OH).

H-Aib*-Aib-Aib*(50%)-Aib-D-αMv-NHMe – S56

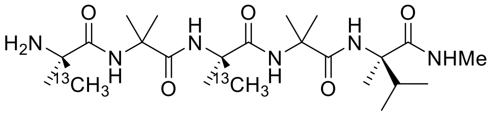

From **S55** (17 mg, 0.03 mmol) according to **General Procedure B**. **S56** was obtained as a clear film (16 mg, 0.03 mmol, *quant.*). **R_f** 0.12 (19:1 DCM:MeOH); **m.p.** 125-127 °C; **δ_H** (400 MHz, CD_3OD) 2.75 (3H, m, $NHCH_3$), 2.15 (1H, sep, *J* = 6.5, $CH(CH_3)_2$), 1.55-1.34 (24H, m, $NHC(CH_3)_2$, $NHC(^{13}CH_3)(CH_3)$ $NHC(^{13}CH_3)(CH_3)$), 1.48 (3H, s, $NHC(CH_3)_2$), 1.48 (1.22-1.17 (3H, m, $NHC(CH_3)$), 1.04 (3H, d, *J* = 6.6, $CH(CH_3)_2$), 0.96 (3H, d, *J* = 6.6, $CH(CH_3)_2$); **δ_C** (100 MHz, $CDCl_3$) 179.7 (*C*ONH), 177.3 (*C*ON*H*), 177.1 (*C*ONH), 177.0 (*C*ONH), 176.2 (*C*ONH), 64.6 ($NHC(CH_3)$), 58.1 ($NHC(CH_3)_2$), 57.5 (0.5, d, J = 35.2, $NHC(^{13}CH_3)(CH_3)$), 57.5 (0.5, $NHC(CH_3)(CH_3)$, 57.5 ($NHC(CH_3)_2$), 55.7 (d, *J* = 36.4 ($NHC(^{13}CH_3)(CH_3)$), 36.9 ($CH(CH_3)_2$), 28.5 ($H_2NC(^{13}CH_3)(CH_3)_{minor}$), 28.3 ($NHC(^{13}CH_3)(CH_3)_{major}$), 27.2 ($NHC(CH_3)_2$), 26.5 ($NHCH_3$), 25.8 ($NHC(CH_3)_2$), 25.3 ($NHC(CH_3)_2$), 26.9 ($H_2NC(^{13}CH_3)(CH_3)_{minor}$), 24.1 ($NHC(CH_3)_2$), 23.7 ($NHC(^{13}CH_3)(CH_3)_{minor}$), 18.3 ($NHC(CH_3)$) 17.7 ($CH(CH_3)_2$), 17.4 ($CH(CH_3)_2$); **$\nu_{max}$/cm^{-1}** 3326w.br (N-H), 2978w (C-H), 1652m (C=O), 1623s (C=O), 1417m (C-N); ***m/z*** (ES^-) 485.4 and 486.4 ($[M]^-$, 100%), HRMS (ESI^+) found $[M+Na]^+$ 508.3305 and 509.3330 $[C_{22}{}^{13}CH_{44}N_6O_5Na]^+$ and $[C_{21}{}^{13}C_2H_{44}N_6O_5Na]^+$ require 508.3304 and 509.3338; **$[\alpha]_D^{20}$** = −9.3 (*c* 1.00; CH_3OH).

Cbz-L-αMv-Aib_3-Aib*-Aib-Aib*(50%)-Aib-D-αMv-NHMe – 6b

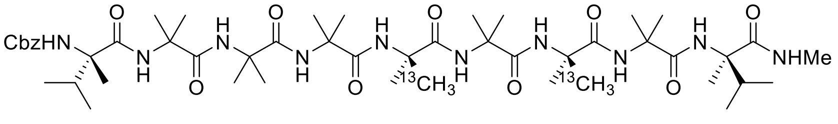

From **S30** (105 mg, 0.20 mmol), EDC.HCl (57 mg, 0.30 mmol), Et_3N (42 μL, 0.30 mmol) and **S56** NHMe (98 mg, 0.20 mmol) according to **General Procedure F**. Crude **6b** was purified by automatic purification system (99:1 DCM:MeOH increasing to 9:1) to give the pure compound as a white solid (153 mg, 0.15 mmol, 75%). **R_f** 0.22 (19:1 DCM:MeOH); **m.p.** >250 °C; **δ_H** (500 MHz, $CDCl_3$) 7.79 (1H, br,

s, CON*H*), 7.73 (1H, br, s, CON*H*), 7.71 (1H, br, s, CON*H*), 7.68 (1H, br, s, CON*H*), 7.63 (1H, br, s, CON*H*), 7.51 (1H, br, s, CON*H*), 7.38 – 7.30 (6H, m, CON*H*, Ph*H*), 7.12 (1H, br, s, CON*H*), 6.86 (1H, br, s, CON*H*), 6.11 (1H, br, s, CON*H*), 5.11 (1H, d, *J* = 12.5, PhCH_2), 5.08 (1H, d, *J* = 12.5, PhCH_2), 2.77 (3H, d, *J* = 4.5, NHCH_3), 2.38-2.28 (1H, m, C*H*(CH_3)$_2$), 2.20 (1H, sep, *J* = 7.0, C*H*(CH_3)$_2$), 1.51 (7.5H, m, NHC(CH_3)$_2$, NHC(CH_3)$_{2\text{(unlabelled)}}$ (50%)), 1.51 (1.5H, d, J =129.5, NHC(CH_3)(^{13}CH_3) NHC(CH_3)($^{13}CH_3$) (50%)), 1.49 (9H, m, NHC(CH_3)$_2$), 1.48 (3H, d, J =129.5, NHC(CH_3)(^{13}CH_3), NHC(^{13}CH_3)(CH_3) (100%)), 1.48 (3H, m, NHC(CH_3)($^{13}CH_3$), NHC($^{13}CH_3$)(CH_3) (100%)), 1.47 (6H, m, NHC(CH_3)$_2$), 1.46 (3H, s, NHC(CH_3)$_2$), 1.43 (3H, s, NHC(CH_3)$_2$), 1.42 (3H, s, NHC(CH_3)$_2$), 1.42 (3H, s, NHC(CH_3)$_2$), 1.41 (3H, s, NHC(CH_3)), 1.36 (3H, s, NHC(CH_3)), 1.00 – 0.94 (12H, m, CH(CH_3)$_2$); **δ_C** (125 MHz, $CDCl_3$) 176.2 (*C*ONH), 175.9 (*C*ONH), 175.8 (*C*ONH), 175.7 (*C*ONH), 175.6 (*C*ONH), 175.5 (*C*ONH), 174.7 (*C*ONH), 174.6 (*C*ONH), 174.2 (*C*ONH), 156.3 (*C*O_2Bn), 136.3, 128.6, 128.4, 127.9 (Ar), 67.0 (Ph*C*H_2), 62.9 (NH*C*(CH_3)), 62.6 (NH*C*(CH_3)), 57.1 (NH*C*(CH_3)$_2$), 56.7 (NH*C*(CH_3)$_2$), 56.7 (NH*C*(CH_3)$_2$), 56.7 (NH*C*(CH_3)$_2$), 56.6 (0.5, d, *J* = 37.0, NH*C*(CH_3)($^{13}CH_3$)), 56.6 (0.5, NH*C*(CH_3)(CH_3)), 56.6 (NH*C*(CH_3)$_2$), 56.5 (d, *J*=37.5, NH*C*(CH_3)($^{13}CH_3$)), 34.6 (*C*H(CH_3)$_2$), 34.2 (*C*H(CH_3)$_2$), 27.1 (NHC(*C*H_3)$_2$), 26.9 (NHC(*C*H_3)$_2$), 26.3 (NH*C*H_3), 26.1 (NHC(*C*H_3)$_2$), 25.3 (NHC(CH_3)(^{13}*C*H_3), NHC(CH_3)(*C*H_3)), 24.9 (NHC(CH_3)(^{13}*C*H_3)), 22.7 (NHC(*C*H_3)$_2$), 17.8 (CH(*C*H_3)$_2$), 17.6 (CH(*C*H_3)$_2$), 17.6 (CH(*C*H_3)$_2$), 17.4 (CH(*C*H_3)$_2$); **ν_{max}/cm^{-1}** 3294m.br (N-H), 2984m (C-H), 1655s (C=O), 1533m (C-N); ***m/z*** (ESI$^+$) 1010.6 and 1011.6 ([M+Na]$^+$, 100%), HRMS (ESI$^+$) found [M+Na]$^+$ 1010.6085 and 1011.6114 [$C_{48}{}^{13}CH_{82}N_{10}O_{11}Na$] and [$C_{47}{}^{13}C_2H_{82}N_{10}O_{11}Na$] require 1010.6090 and 1011.6124; **$[\alpha]_D^{20}$** = −0.7 (*c* 1.00; CH_3OH).

N_3-Aib$_2$-D-αMv-NHMe – S57

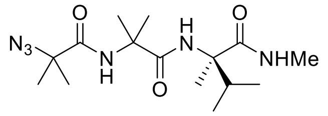

From **S6** (262 mg, 2.03 mmol), oxalyl chloride (0.55 mL, 2.03 mmol), **D-S20** (310 mg, 1.35 mmol) and Et_3N (0.29 mL, 2.03 mmol) according to **General Procedure A**. Crude **S57** was purified by flash column chromatography (99:1 DCM:MeOH increasing to 19:1) to give the pure compound as a white solid (168 mg, 0.49 mmol, 36%). **R_f** 0.53 (19:1 DCM:MeOH); **m.p.** 125-127 °C; **δ_H** (500 MHz, $CDCl_3$) 7.07 (1H, d, *J* = 4.5, N*H*CH_3), 6.95 (1H, s, CON*H*), 6.21 (1H, s, CON*H*), 2.72 (3H, d, *J* = 4.5, NHCH_3), 2.12 (1H, sep, *J* = 6.9, C*H*(CH_3)$_2$), 1.50 (3H, s, NHC(CH_3)$_2$), 1.47 (6H, s, NHC(CH_3)$_2$, NHC(CH_3)), 1.47 (3H, s, N_3C(CH_3)$_2$), 1.43 (3H, s, N_3C(CH_3)$_2$), 0.86 (3H, d, *J* = 6.9, CH(CH_3)$_2$), 0.82 (3H, d, *J* = 6.9, CH(CH_3)$_2$); **δ_C** (125 MHz, $CDCl_3$) 173.7 (*C*ONH), 173.1 (*C*ONH), 172.6 (*C*ONH), 64.1 (N_3*C*(CH_3)$_2$), 63.3 (NH*C*(CH_3)), 57.5 (NH*C*(CH_3)$_2$), 35.6 (*C*H(CH_3)$_2$), 26.4 (NH*C*H_3), 25.8 (N_3C(*C*H_3)$_2$), 24.3 (NHC(*C*H_3)$_2$), 19.7 (NHC(*C*H_3)) 17.4 (CH(*C*H_3)$_2$), 16.8 (CH(*C*H_3)$_2$); **ν_{max}/cm^{-1}** 3353m.br (N-H), 2978m (C-H), 2111s (N_3), 1652s (C=O), 1502s (C-N); ***m/z*** (ESI$^+$) 341.2 ([M+H]$^+$, 100%), HRMS (ESI$^+$) found [M+H]$^+$ 341.2317 [$C_{15}H_{29}N_6O_3$]$^+$ requires 341.2301; **$[\alpha]_D^{20}$** = −16.8 (*c* 1.10; CH_3OH).

H-Aib$_2$-D-αMv-NHMe – S58

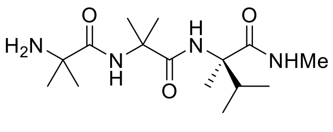

From **S57** (168 mg, 0.49 mmol) according to **General Procedure B**. **S58** was obtained as a clear film (116 mg, 0.37 mmol, 76%). **R$_f$** 0.05 (EtOAc); **m.p.** 203-205 °C; **δ$_H$** (400 MHz, $CDCl_3$) 8.22 (1H, s, CON*H*), 7.36 (1H, d, *J* = 4.6, CON*H*), 6.29 (1H, s, CON*H*), 2.76 (3H, d, *J* = 4.6, NHC*H*$_3$), 2.13 (1H, sep, *J* = 6.9, C*H*(CH_3)$_2$), 1.78 (2H, s, br, N*H*$_2$), 1.54 (3H, s, NHC(C*H*$_3$)$_2$), 1.49 (3H, s, NHC(C*H*$_3$)$_2$), 1.46 (3H, s, NHC(C*H*$_3$)), 1.34 (6H, s, H_2NC(C*H*$_3$)$_2$), 0.90 (3H, d, *J* = 6.9, CH(C*H*$_3$)$_2$), 0.85 (3H, d, *J* = 6.9, CH(C*H*$_3$)$_2$); **δ$_C$** (100 MHz, $CDCl_3$) 178.8 (*C*ONH), 173.9 (*C*ONH), 173.3 (*C*ONH), 63.2 (NH*C*(CH_3)), 57.0 (NH*C*(CH_3)$_2$), 55.0 (H_2N*C*(CH_3)$_2$), 35.8 (*C*H(CH_3)$_2$), 29.1 (H_2NC(*C*H$_3$)$_2$), 28.9 (H_2NC(*C*H$_3$)$_2$), 26.5 (NH*C*H$_3$), 26.2 (NH*C*(CH_3)$_2$), 24.3 (NH*C*(CH_3)$_2$), 20.0 (NHC(*C*H$_3$)) 17.6 (CH(*C*H$_3$)$_2$), 16.8 (CH(*C*H$_3$)$_2$); **ν$_{max}$/cm^{-1}** 3329m.br (N-H), 2967m (C-H), 1649s (C=O), 1503m (C-N); ***m/z*** (ESI$^+$) 315.2 ([M+H]$^+$, 100%), HRMS (ESI$^+$) found [M+Na]$^+$ 337.2221 [$C_{15}H_{30}N_4O_3Na$]$^+$ requires 337.2216; **[α]$_D^{20}$** = −16.4 (*c* 1.00; CH_3OH).

N_3-Aib*-Aib$_2$-D-αMv-NHMe – S59

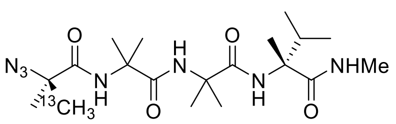

From **S48** (48 mg, 0.37 mmol), oxalyl chloride (34 μL, 0.41 mmol), **S58** (92 mg, 0.29 mmol) and Et_3N (57 μL, 0.41 mmol) according to **General Procedure A**. Crude **S59** was purified by flash column chromatography (99:1 DCM:MeOH increasing to 19:1) to give the pure compound as a white solid (30 mg, 0.07 mmol, 24%). **R$_f$** 0.38 (19:1 DCM:MeOH); **m.p.** 130-132 °C; **δ$_H$** (500 MHz, $CDCl_3$) 7.14 (1H, d, *J* = 4.6, N*H*CH$_3$), 6.91 (1H, s, CON*H*), 6.78 (1H, s, CON*H*), 6.41 (1H, s, CON*H*), 2.77 (3H, d, *J* = 4.6, NHC*H*$_3$), 2.13 (1H, sep, *J* = 6.8, C*H*(CH_3)$_2$), 1.54-1.47 (12H, m, NHC(C*H*$_3$)$_2$), 1.46 (3H, s, NHC(C*H*$_3$)$_2$,), 1.44 (3H, s, NHC(C*H*$_3$)), 1.41-1.33 (6H, m, N_3C(C*H*$_3$)), 0.93 (3H, d, *J* = 6.8, CH(C*H*$_3$)$_2$), 0.92 (3H, d, *J* = 6.8, CH(C*H*$_3$)$_2$); **δ$_C$** (125 MHz, $CDCl_3$) 174.7 (*C*ONH), 173.6 (*C*ONH), 172.9 (*C*ONH), 172.8 (*C*ONH), 64.2 (d, *J* = 37.1, N_3*C*(CH_3)$_2$), 63.5 (NH*C*(CH_3)), 57.3 (NH*C*(CH_3)$_2$), 57.2 (NH*C*(CH_3)$_2$), 35.5 (*C*H(CH_3)$_2$), 27.3 (NH*C*(CH_3)$_2$), 26.5 (NH*C*H$_3$), 26.1 (NHC(*C*H$_3$)$_2$), 24.6 (N_3C(^{13}*C*H$_3$)$_{minor}$), 24.3 (N_3C(^{13}*C*H$_3$)$_{major}$), 23.9 (NHC(*C*H$_3$)$_2$), 23.7 (NHC(*C*H$_3$)$_2$), 17.9 (NHC(*C*H$_3$)) 17.6 (CH(*C*H$_3$)$_2$), 17.2 (CH(*C*H$_3$)$_2$); **ν$_{max}$/cm^{-1}** 3336m.br (N-H), 2977m (C-H), 2113m (N_3), 1659s (C=O), 1514s (C-N); ***m/z*** (ESI$^+$) 427.3 ([M+H]$^+$, 100%), HRMS (ESI$^+$) found [M+Na]$^+$ 449.2676 [$C_{18}{}^{13}CH_{35}N_7O_4Na$]$^+$ requires 449.2682; **[α]$_D^{20}$** = −12.4 (*c* 1.10; CH_3OH).

H-Aib*-Aib$_2$-D-αMv-NHMe – S60

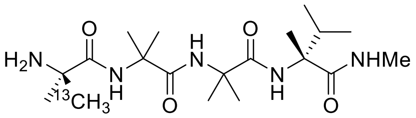

From **S59** (25 mg, 0.06 mmol) according to **General Procedure B**. **S60** was obtained as a clear film (23 mg, 0.06 mmol, *quant.*). $\mathbf{R_f}$ 0.00 (19:1 DCM:MeOH); **m.p.** 203-205 °C; $\boldsymbol{\delta_H}$ (400 MHz, CD_3OD) 2.65 (3H, s, $NHCH_3$), 2.09 (1H, sep, *J* = 6.8, $CH(CH_3)_2$), 1.36 (3H, s, $NHC(CH_3)_2$), 1.34 (3H, s, $NHC(CH_3)_2$), 1.31 (3H, s, $NHC(CH_3)_2$), 1.30 (3H, s, $NHC(CH_3)_2$), 1.26 (3H, s, $NHC(CH_3)$), 1.26-1.21 (3H, m, $H_2NC(CH_3)_2$), 1.11-1.06 (3H, m, $H_2NC(CH_3)_2$), 0.92 (3H, d, *J* = 6.8, $CH(CH_3)_2$), 0.83 (3H, d, *J* = 6.8, $CH(CH_3)_2$); $\boldsymbol{\delta_C}$ (100 MHz, CD_3OD) 179.6 (*C*ONH), 177.4 (*C*ONH), 177.0 (*C*ONH), 176.6 (*C*ONH), 64.5 (NH*C*(CH_3)), 57.9 (NH*C*($CH_3)_2$), 57.3 (NH*C*($CH_3)_2$), 55.8 (d, *J* = 36.5, $H_2NC(CH_3)_2$), 36.6 ($CH(CH_3)_2$), 28.7 $(H_2NC(^{13}CH_3))_{major}$, 28.4 ($NHC(CH_3)_2$, 28.1 $(H_2NC(^{13}CH_3))_{minor}$, 27.2 ($NHC(CH_3)_2$), 26.4 ($NHCH_3$), 25.5 ($NHC(CH_3)_2$), 25.0 ($NHC(CH_3)_2$), 18.4 ($NHC(CH_3)$) 17.8 ($CH(CH_3)_2$), 16.8 ($CH(CH_3)_2$); $\mathbf{v_{max}/cm^{-1}}$ 3339w.br (N-H), 2973w (C-H), 1649s (C=O), 1413m (C-N); ***m/z*** (ESI^+) 423.8 ($[M+Na]^+$, 100%), 401.3 ($[M+H]^+$, 60%), HRMS (ESI^+) found $[M+Na]^+$ 423.2756 $[C_{18}{}^{13}CH_{37}N_5O_4Na]^+$ requires 423.2777; $\mathbf{[\alpha]_D^{20}}$ = −2.4 (*c* 0.50; CH_3OH).

Cbz-L-αMv-Aib$_4$-Aib*-Aib$_2$-D-αMv-NHMe – 6c

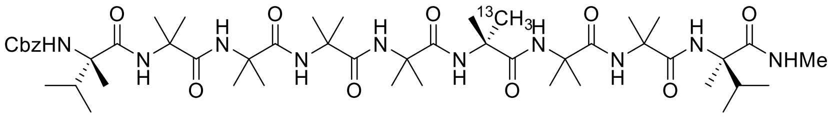

From **S37** (38 mg, 0.06 mmol), EDC.HCl (18 mg, 0.09 mmol), DIPEA (16 µL, 0.09 mmol) and **S60** (23 mg, 0.06 mmol) according to **General Procedure F**. Crude **6c** was purified by flash column chromatography (99:1 DCM:MeOH increasing to 19:1) to give the pure compound as a clear solid (10 mg, 0.01 mmol, 17%). $\mathbf{R_f}$ 0.17 (19:1 DCM:MeOH); **m.p.** >250 °C; $\boldsymbol{\delta_H}$ (500 MHz, $CDCl_3$) 7.72-7.70 (2H, m, CON*H*) 7.68 (1H, s, CON*H*), 7.63 (1H, s, CON*H*), 7.60 (1H, s, CON*H*), 7.48 (1H, s, CON*H*), 7.39-7.32 (6H, m, Ph*H*, CON*H*), 7.06 (1H, s, CON*H*), 6.54 (1H, s, CON*H*), 5.59 (1H, s, CON*H*), 5.03 (2H, ABq, $\Delta\delta_{AB}$ = 0.03, J_{AB} = 12.3, $PhCH_2$), 2.79 (3H, d, *J* = 4.6, $NHCH_3$), 2.34 (1H, m, $CH(CH_3)_2$), 2.13 (1H, m. $CH(CH_3)_2$), 1.52-1.50 (9H, m, $NHC(CH_3)_2$), 1.49 (6H, s, $NHC(CH_3)_2$), 1.47 (12H, s, $NHC(CH_3)_2$), 1.46 (6H, s, $NHC(CH_3)_2$), 1.44 (3H, s, $NHC(CH_3)_2$), 1.43 (3H, s, $NHC(CH_3)_2$), 1.41 (6H, s, $NHC(CH_3)_2$, $NHC(CH_3)$), 1.33 (3H, s, $NH(CH_3)$), 0.99 (3H, d, *J* = 7.2, $CH(CH_3)_2$), 0.97 (3H, d, *J* = 7.2, $CH(CH_3)_2$), 0.96 (3H, d, *J* = 6.4, $CH(CH_3)_2$), 0.95 (3H, d, *J* = 6.4, $CH(CH_3)_2$); $\boldsymbol{\delta_C}$ (125 MHz, $CDCl_3$); 176.2 (*C*ONH), 175.8 (*C*ONH), 175.7 (*C*ONH), 175.5 (*C*ONH), 175.4 (*C*ONH), 174.6 (*C*ONH), 174.4 (*C*ONH), 173.6 (*C*ONH), 156.3 (CO_2Bn), 136.0, 128.9, 128.8, 128.2 (Ar), 67.6 ($PhCH_2$), 63.0 (NH*C*(CH_3)), 62.8 (NH*C*(CH_3)), 57.2 (NH*C*($CH_3)_2$), 56.9 (NH*C*($CH_3)_2$), 56.9 (NH*C*($CH_3)_2$), 56.7 (NH*C*($CH_3)_2$), 56.7 (NH*C*($CH_3)_2$), 56.6 (NH*C*($CH_3)_2$), 56.6 (NH*C*($CH_3)_2$), 30.5 ($CH(CH_3)_2$), 29.9 ($CH(CH_3)_2$), 26.9 ($NHC(CH_3)_2$), 26.5 ($NHCH_3$), 26.4 ($NHC(CH_3)_2$), 25.6-24.6 ($NHC(CH_3)_2$), $NHC(^{13}CH_3)$), 18.5 ($NHC(CH_3)$), 18.0 ($NHC(CH_3)$), 18.0 ($CH(CH_3)_2$), 17.8 ($CH(CH_3)_2$), 17.6 ($CH(CH_3)_2$), 17.4 ($CH(CH_3)_2$); $\mathbf{v_{max}/cm^{-1}}$ 3292m.br (N-H), 3984w

(C-H), 1655s (C=O), 1535m (C-N); ***m/z*** (ESI$^+$) 1010.7 ([M+Na]$^+$, 100%), HRMS (ESI$^-$) found [M-H]$^-$ 986.6080 [$C_{48}{}^{13}CH_{81}N_{10}O_{11}$]$^-$ requires 986.6120; **$[\alpha]_D^{20}$** = −0.4 (*c* 1.00; CH_3OH).

2. NMR Spectra

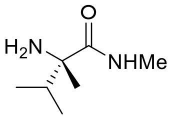

S3

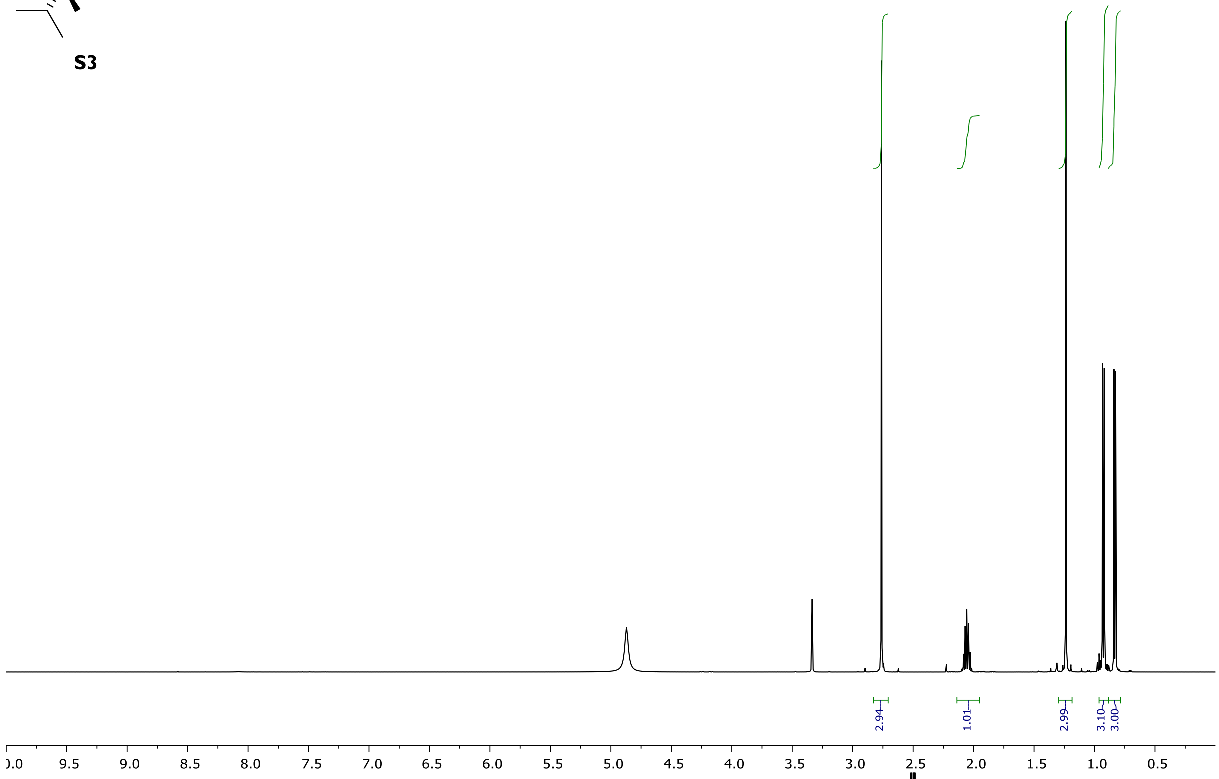

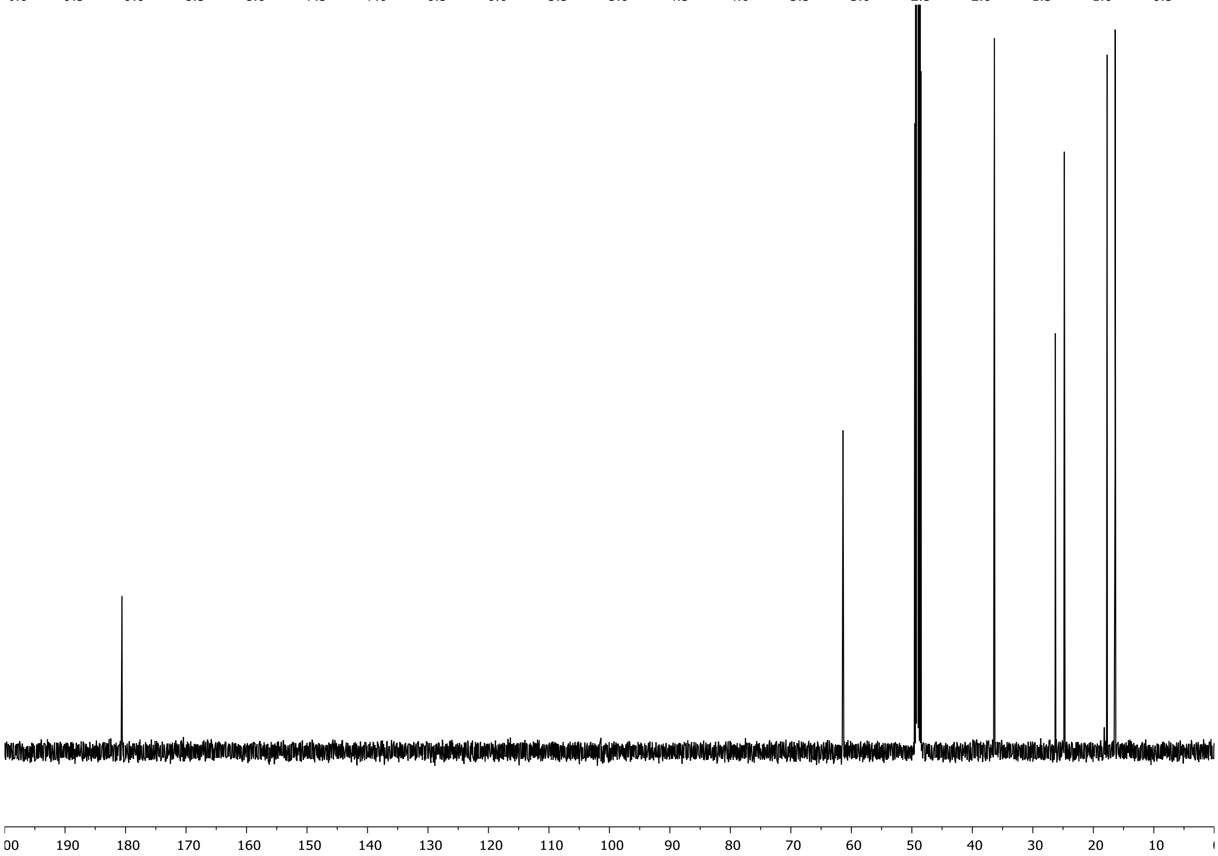

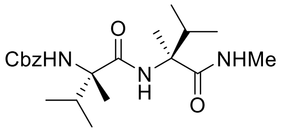
CbzHN
NHMe
O
N
H
O

S4

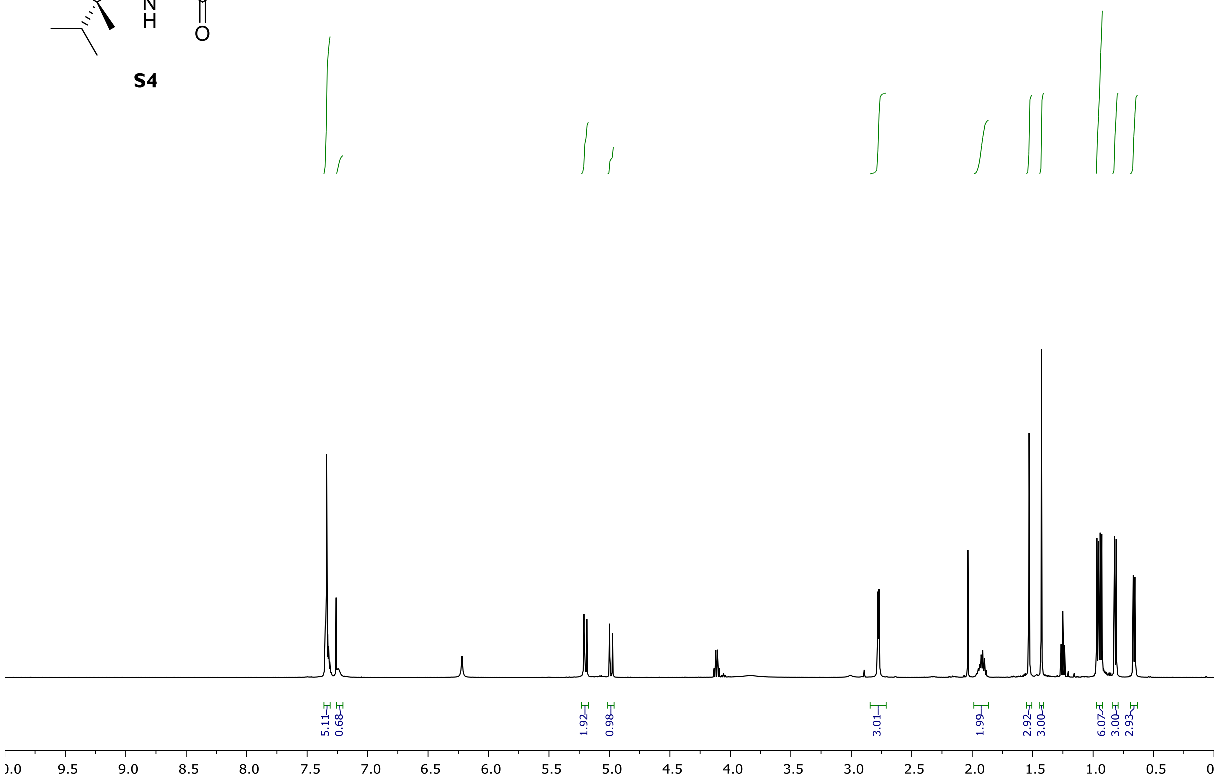
5.11
0.68
1.92
0.98
3.01
1.99
2.92
3.00
6.07
3.00
2.93
9.5
9.0
8.5
8.0
7.5
7.0
6.5
6.0
5.5
5.0
4.5
4.0
3.5
3.0
2.5
2.0
1.5
1.0
0.5
0

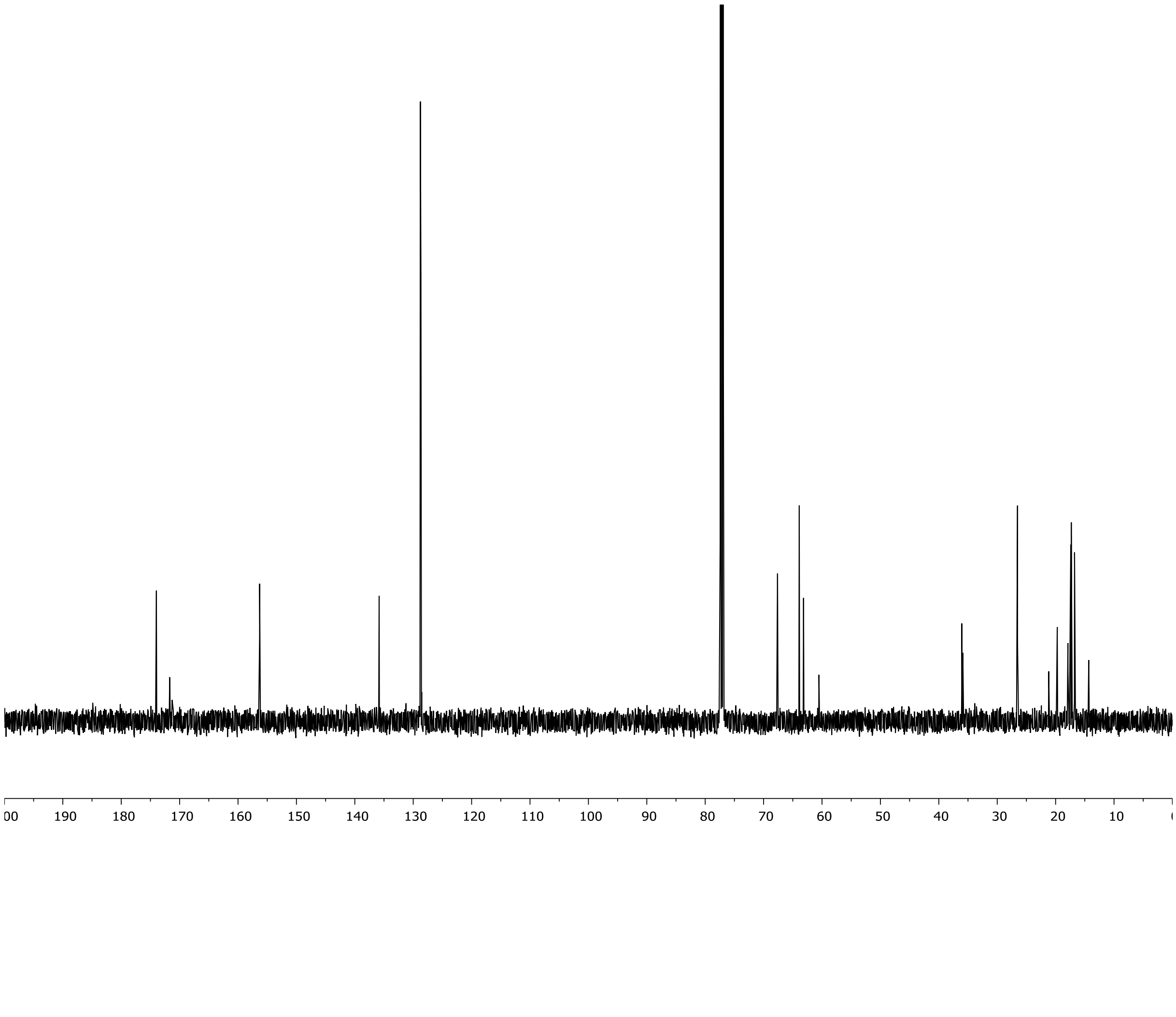
190
180
170
160
150
140
130
120
110
100
90
80
70
60
50
40
30
20
10

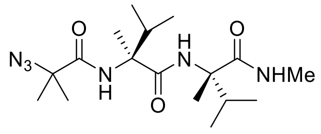
N3
O
N
H
O
H
N
O
NHMe

S7

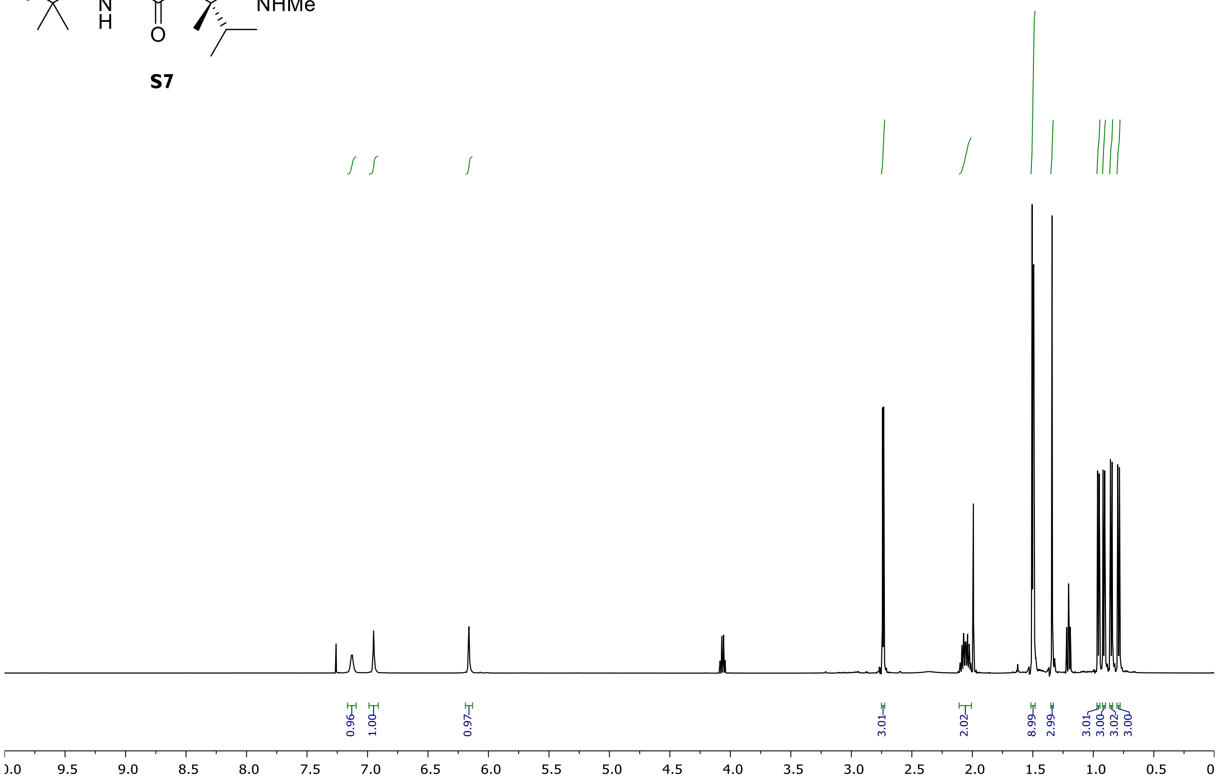
0.96
1.00
0.97
3.01
2.02
8.99
2.99
3.01
3.00
3.02
3.00
0.0
9.5
9.0
8.5
8.0
7.5
7.0
6.5
6.0
5.5
5.0
4.5
4.0
3.5
3.0
2.5
2.0
1.5
1.0
0.5
0

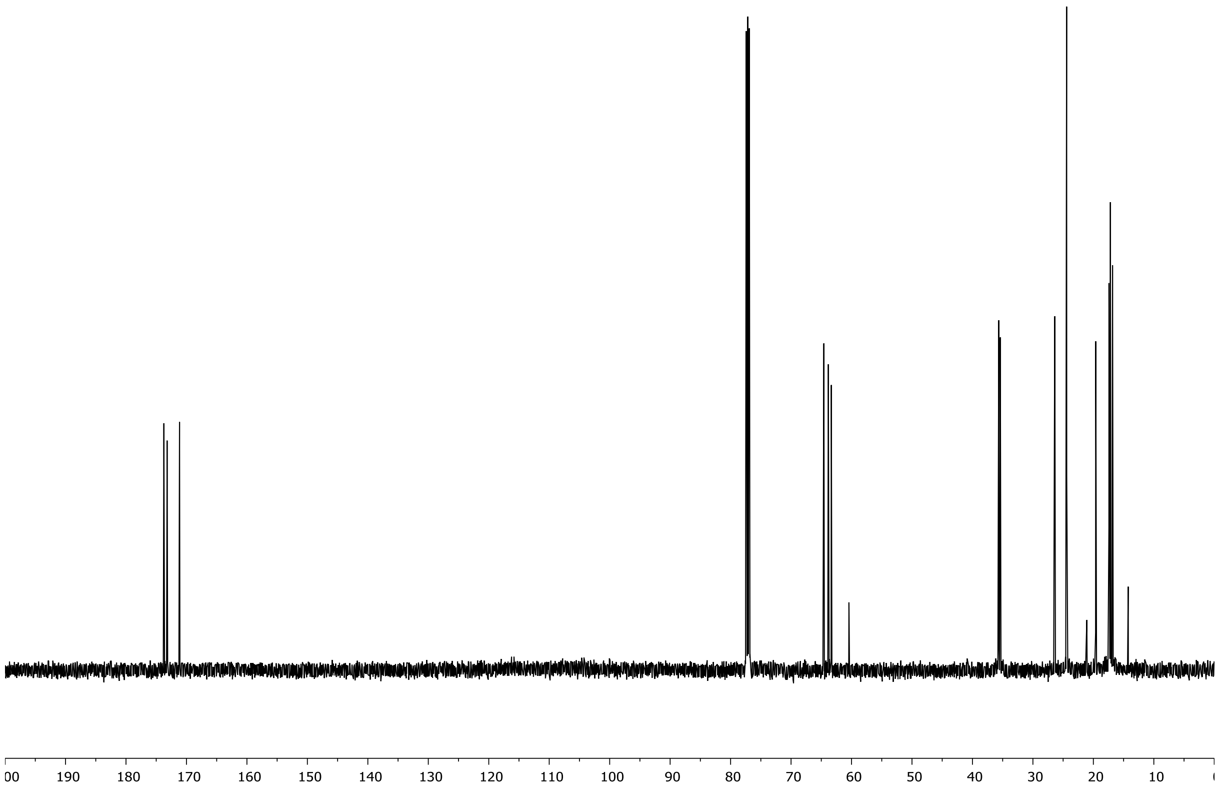
00
190
180
170
160
150
140
130
120
110
100
90
80
70
60
50
40
30
20
10

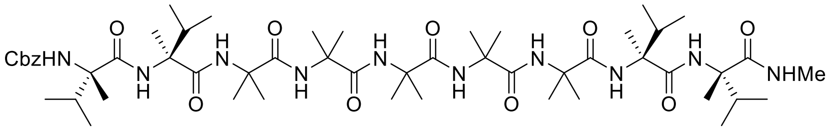
CbzHN
NHMe

1a

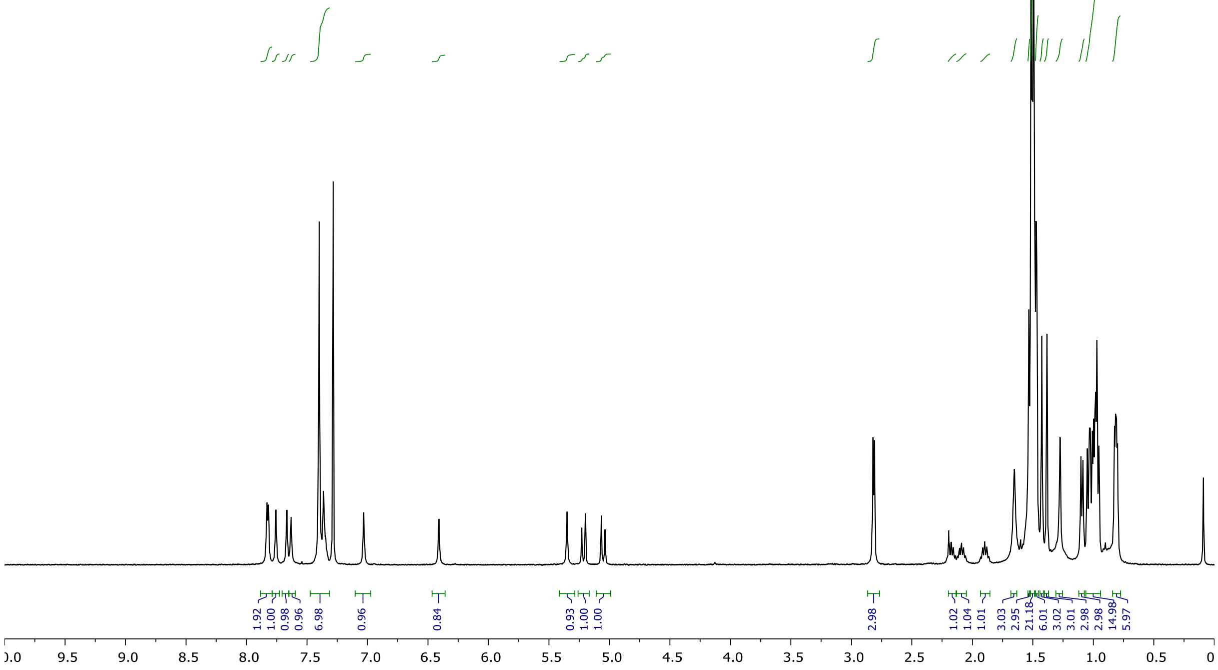
1.92
1.00
0.98
0.96
6.98
0.96
0.84
0.93
1.00
1.00
2.98
1.02
1.04
1.01
3.03
2.95
21.18
6.01
3.02
3.01
2.98
2.98
14.98
5.97

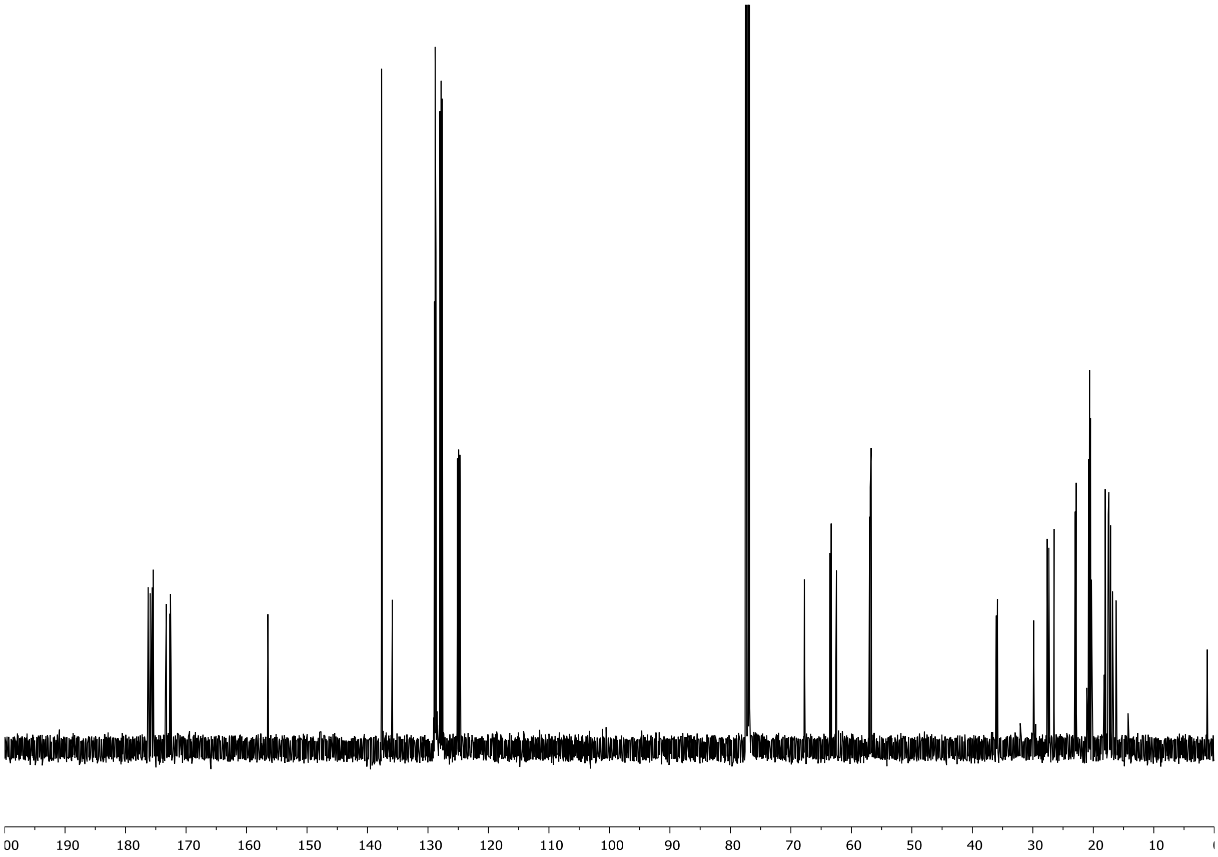

^{15}N HSQC

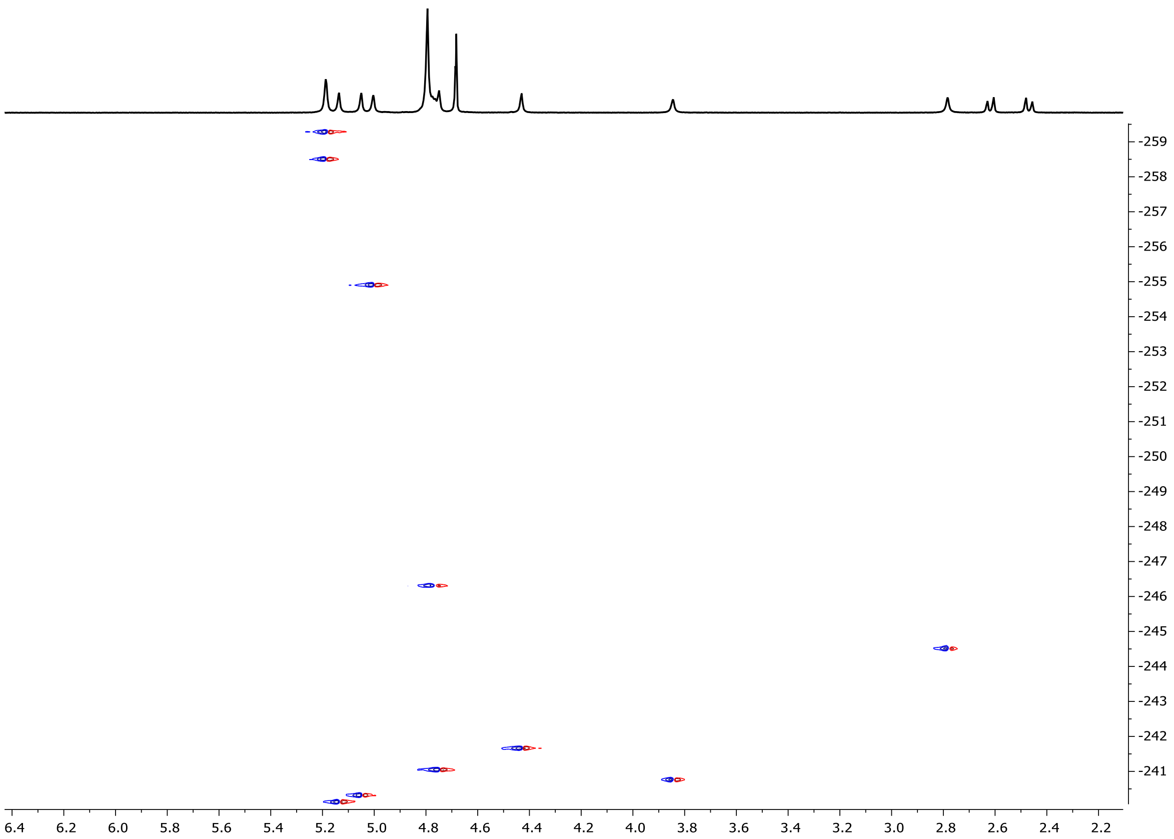

HSQC

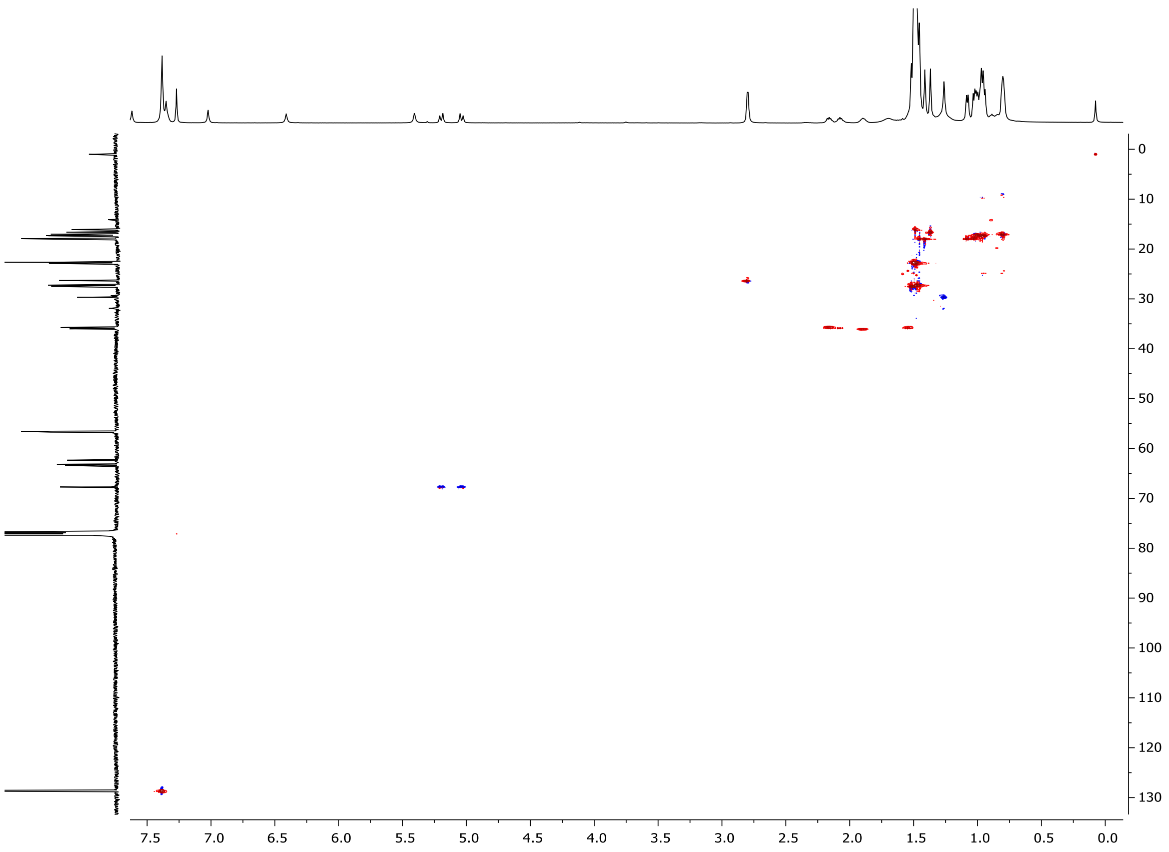

HMBC

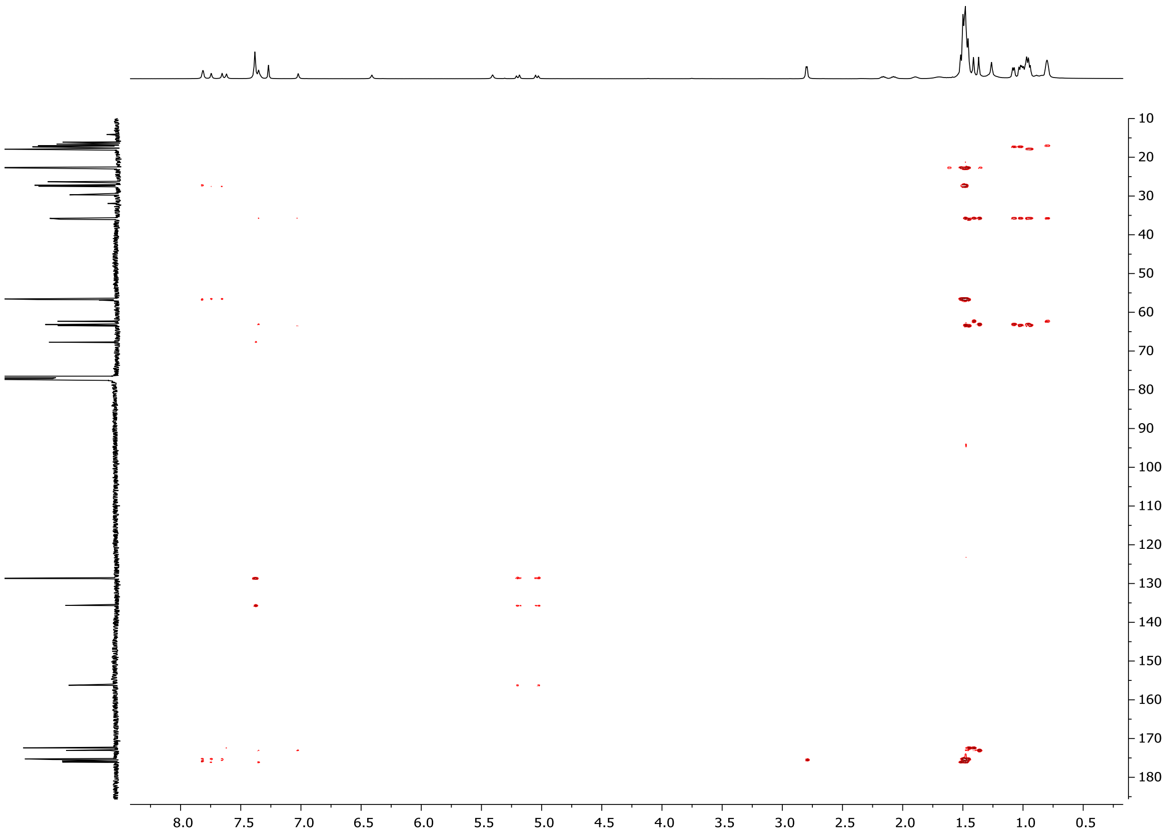

8.0
7.5
7.0
6.5
6.0
5.5
5.0
4.5
4.0
3.5
3.0
2.5
2.0
1.5
1.0
0.5
10
20
30
40
50
60
70
80
90
100
110
120
130
140
150
160
170
180

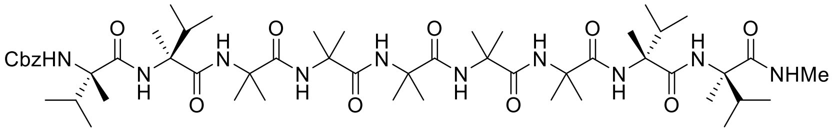

1b

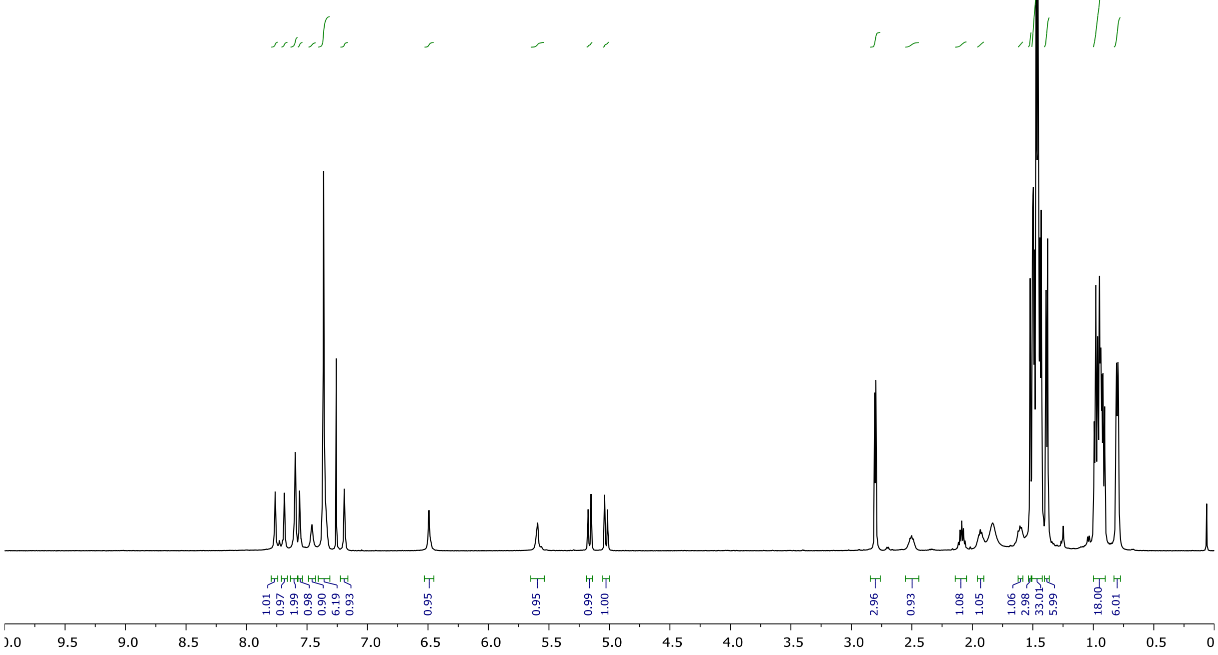

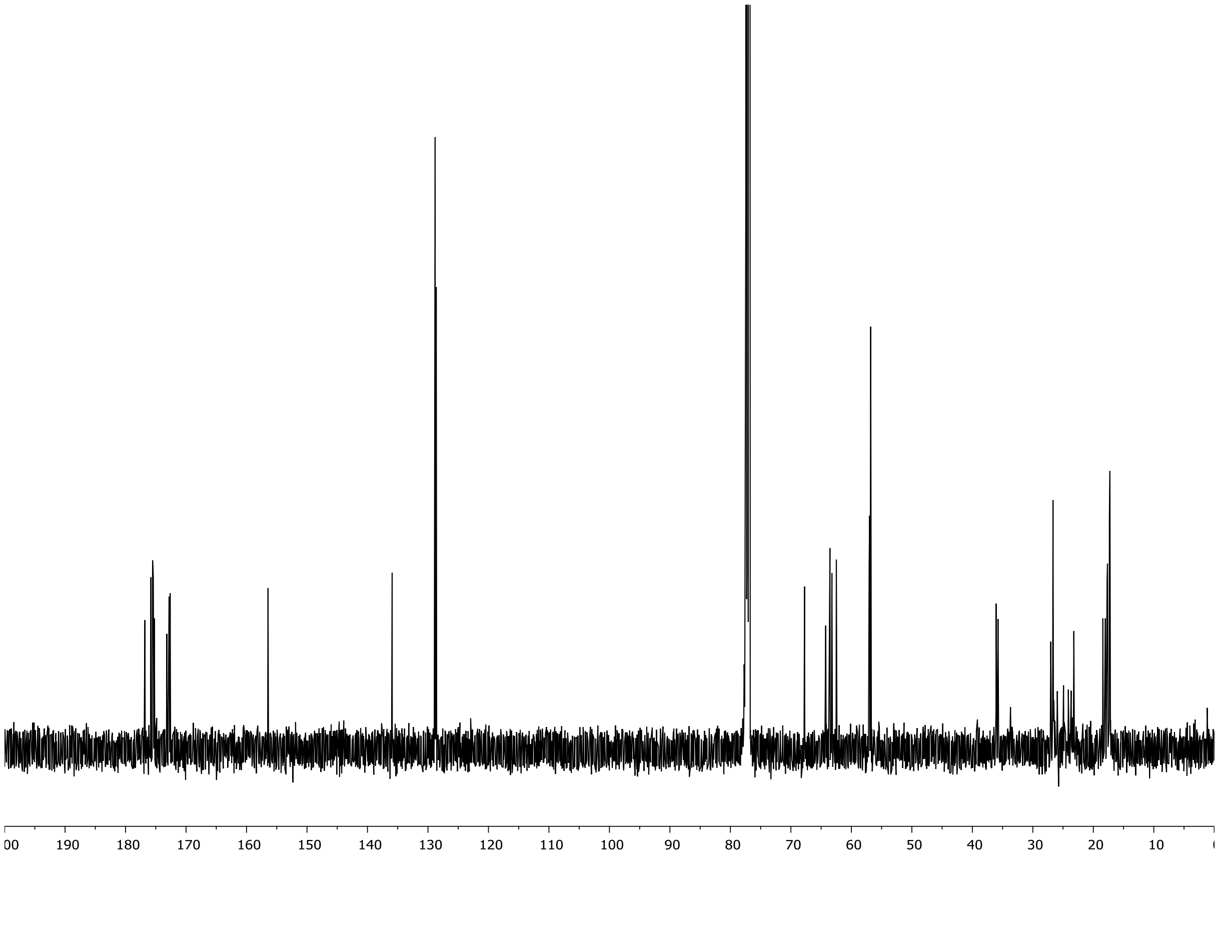

¹⁵N HSQC

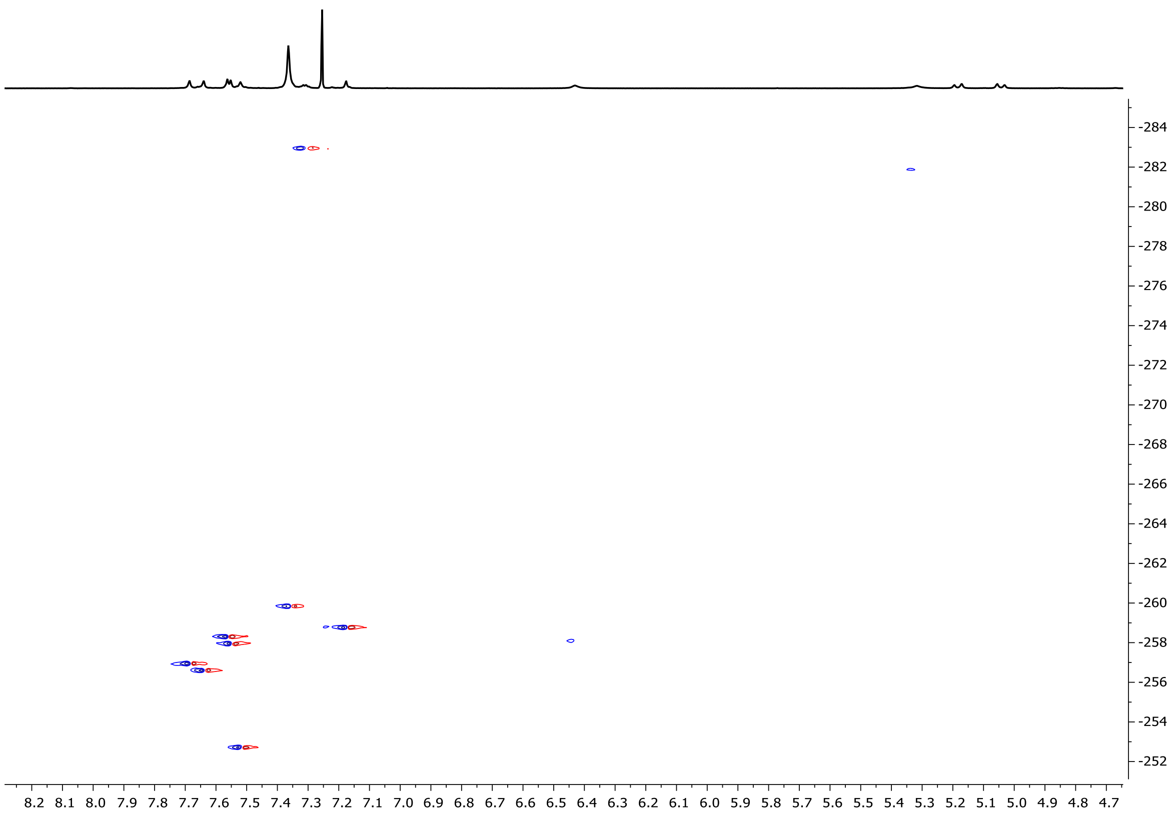

HSQC

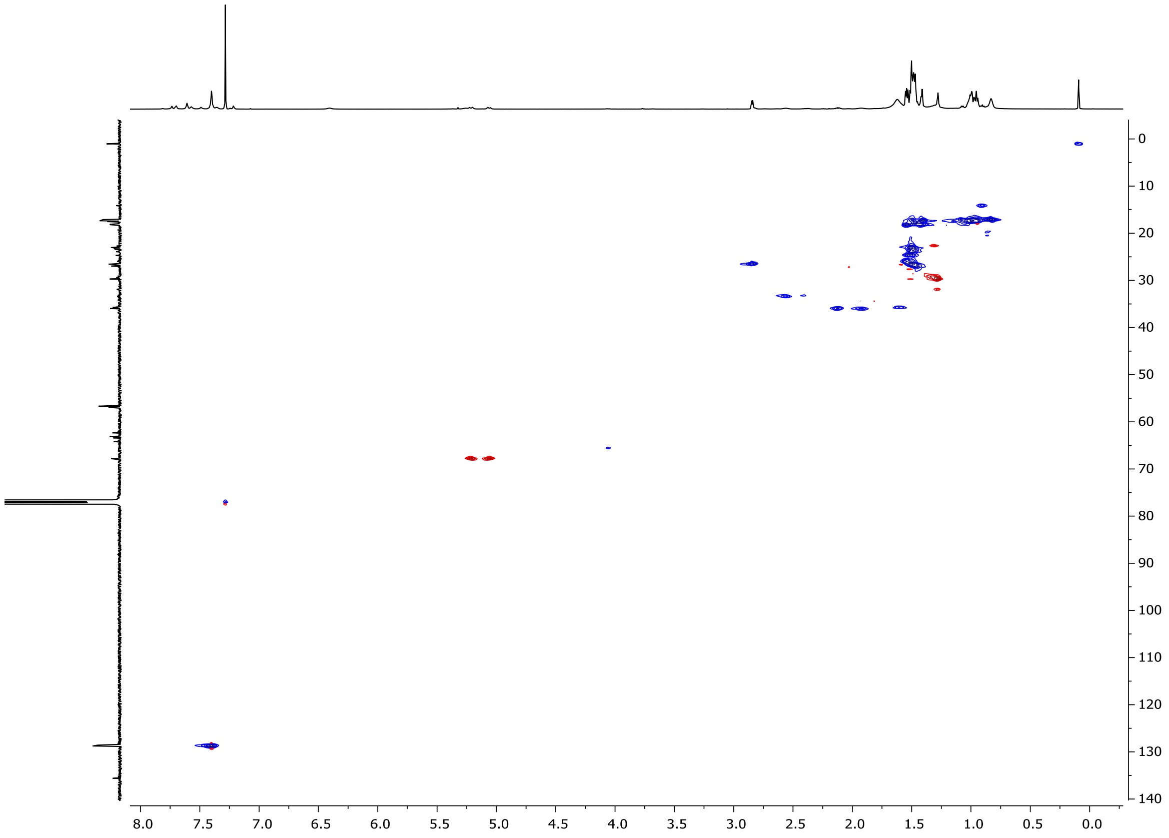

HMBC

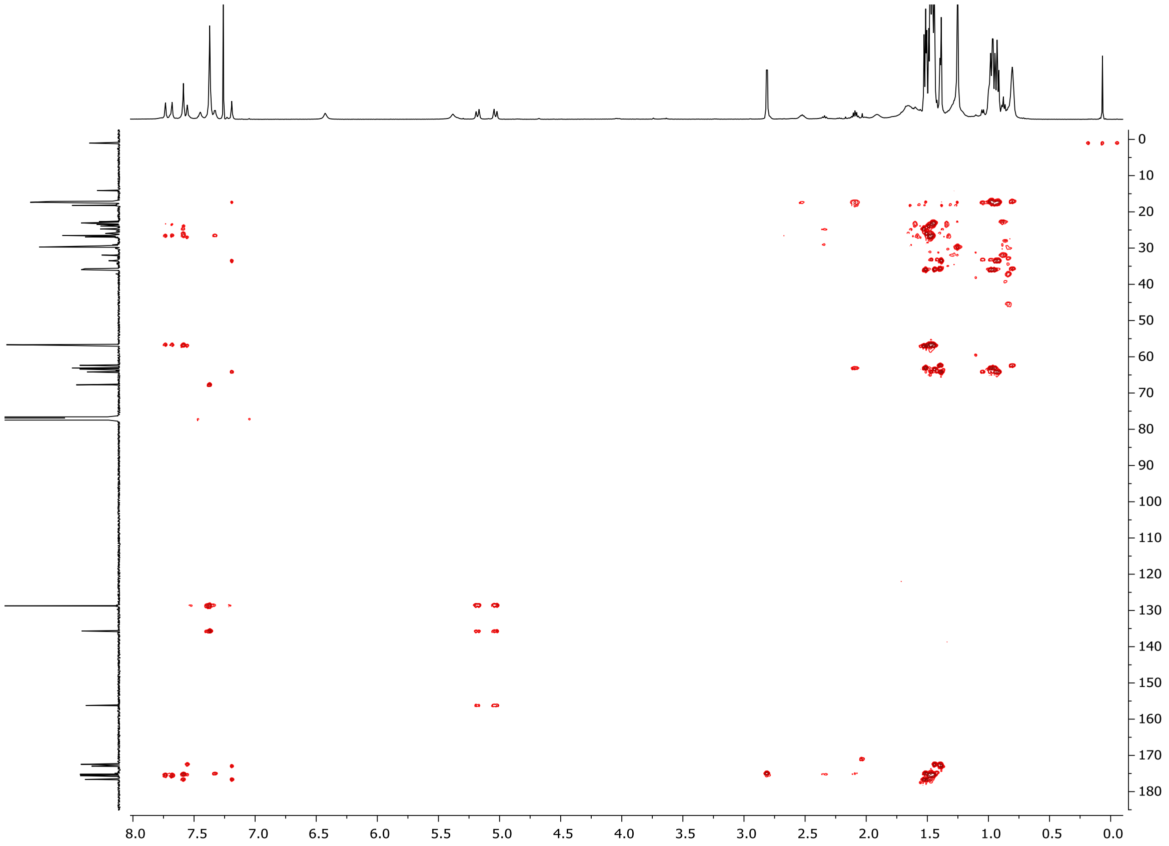

0
10
20
30
40
50
60
70
80
90
100
110
120
130
140
150
160
170
180
8.0 7.5 7.0 6.5 6.0 5.5 5.0 4.5 4.0 3.5 3.0 2.5 2.0 1.5 1.0 0.5 0.0

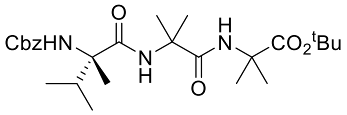

S11

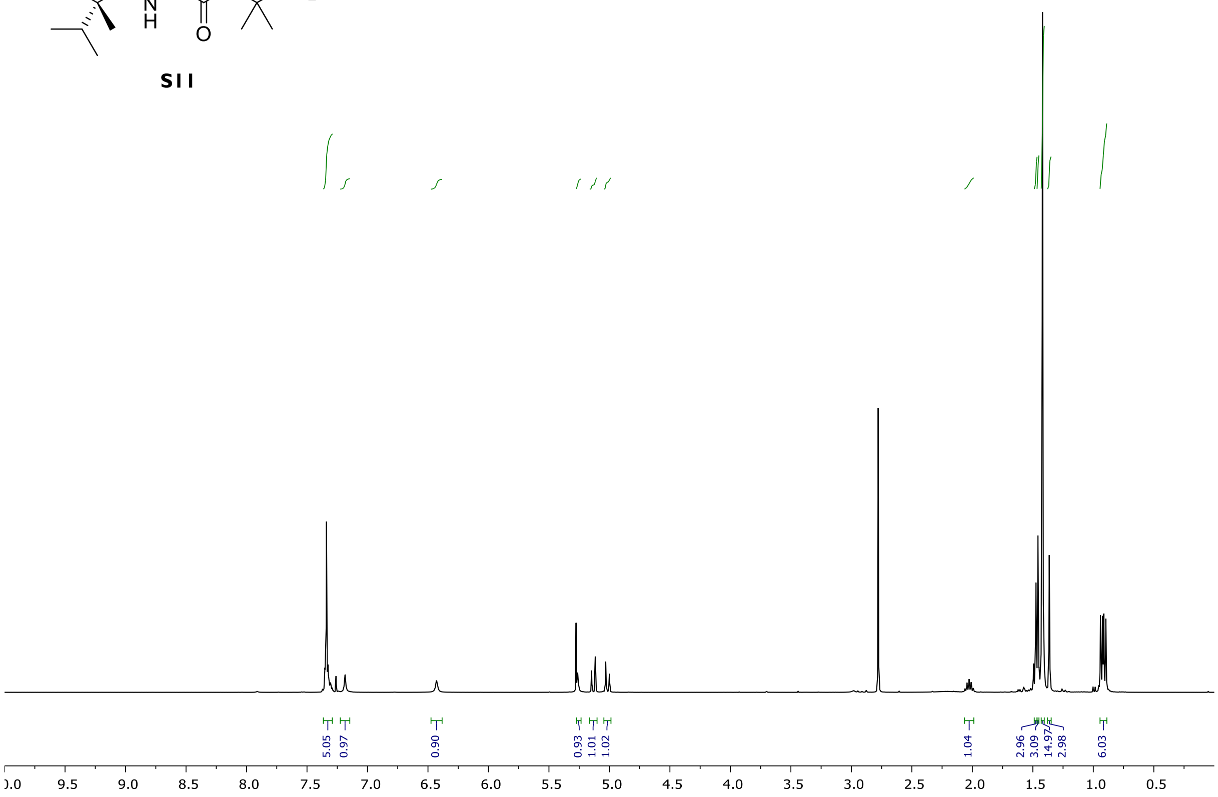

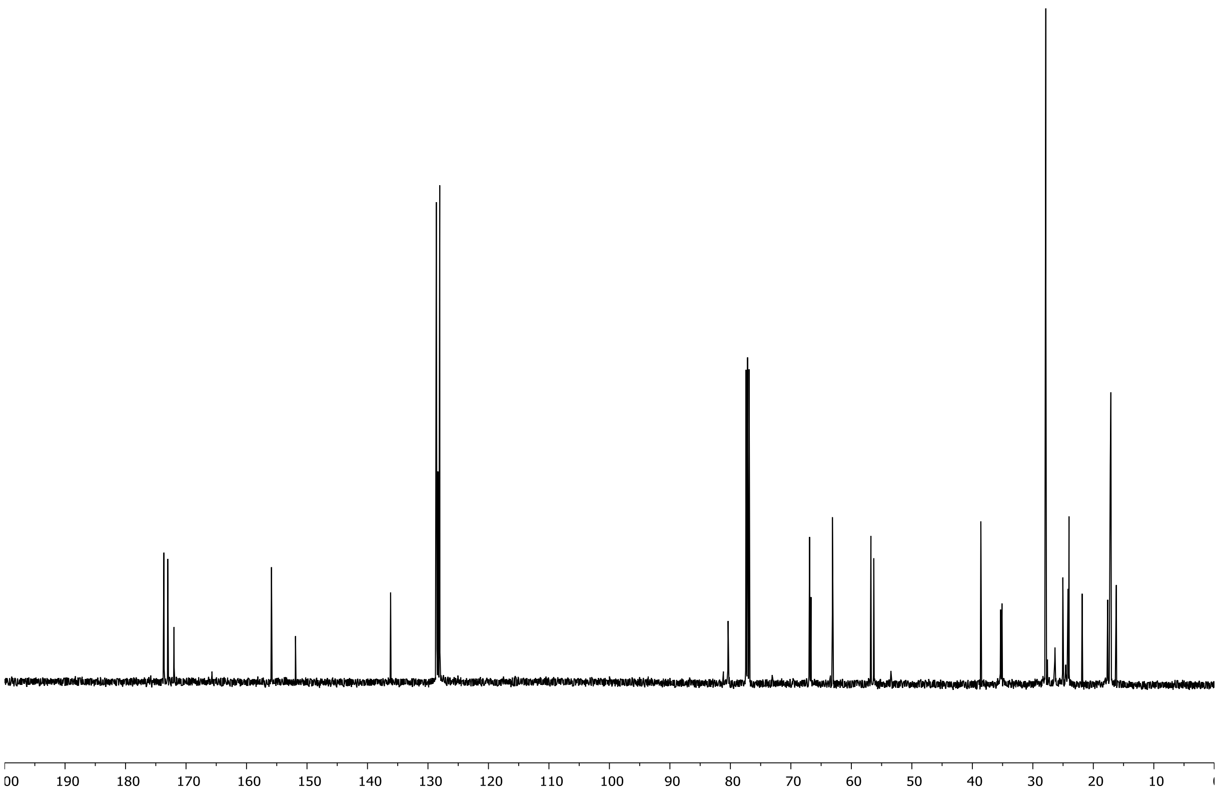

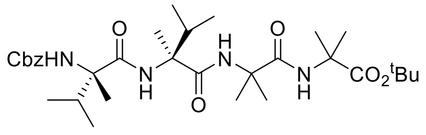

S13

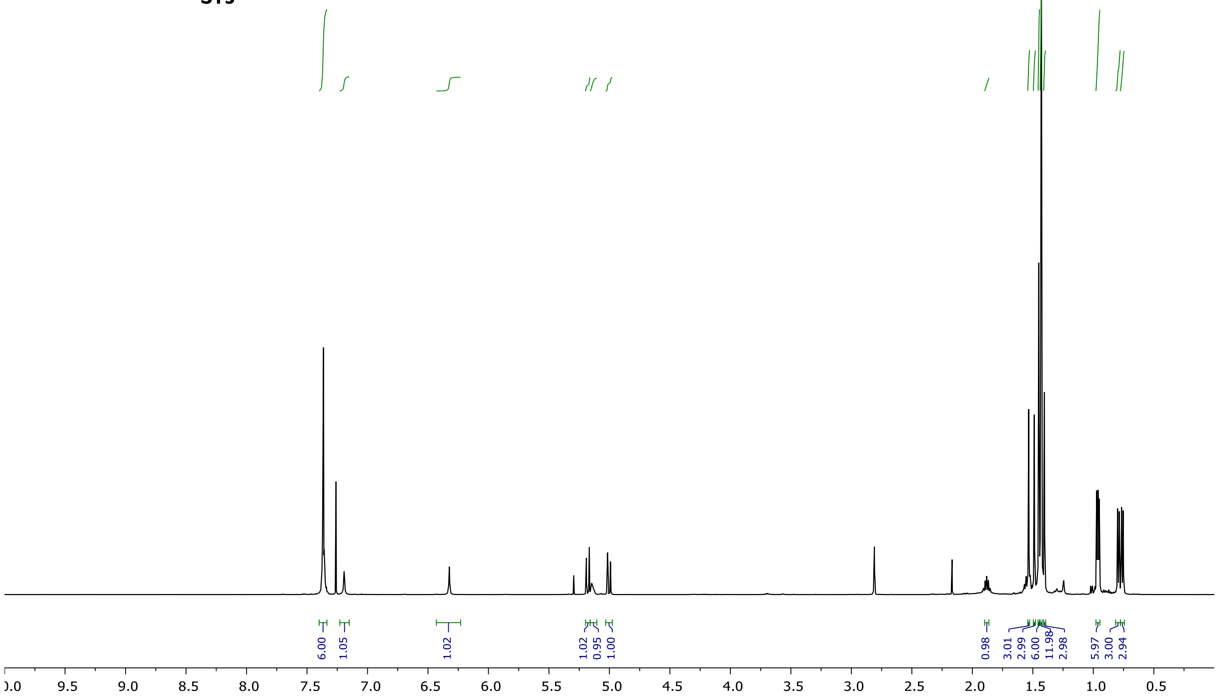

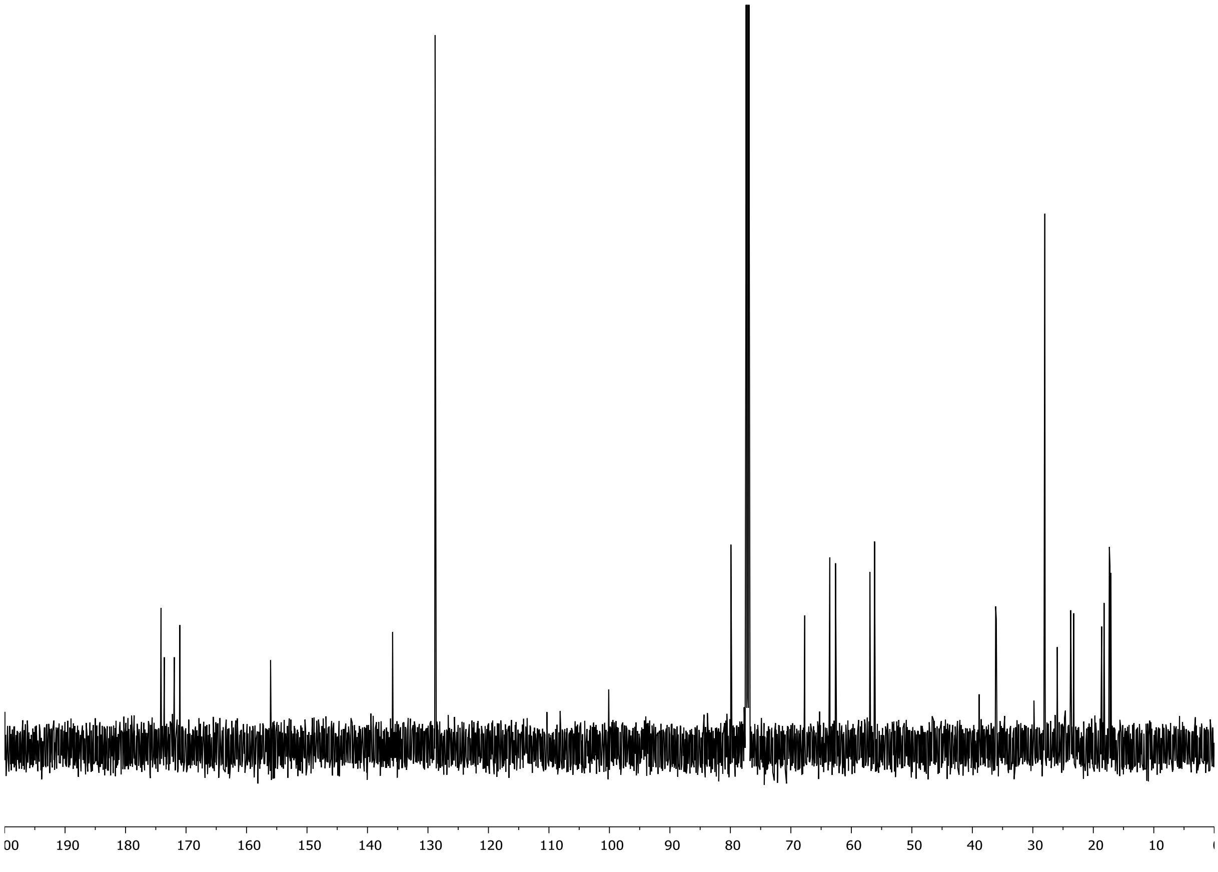

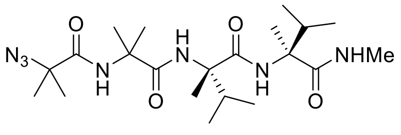
N3
O
N
H
H
N
O
O
N
H
NHMe
O

S15

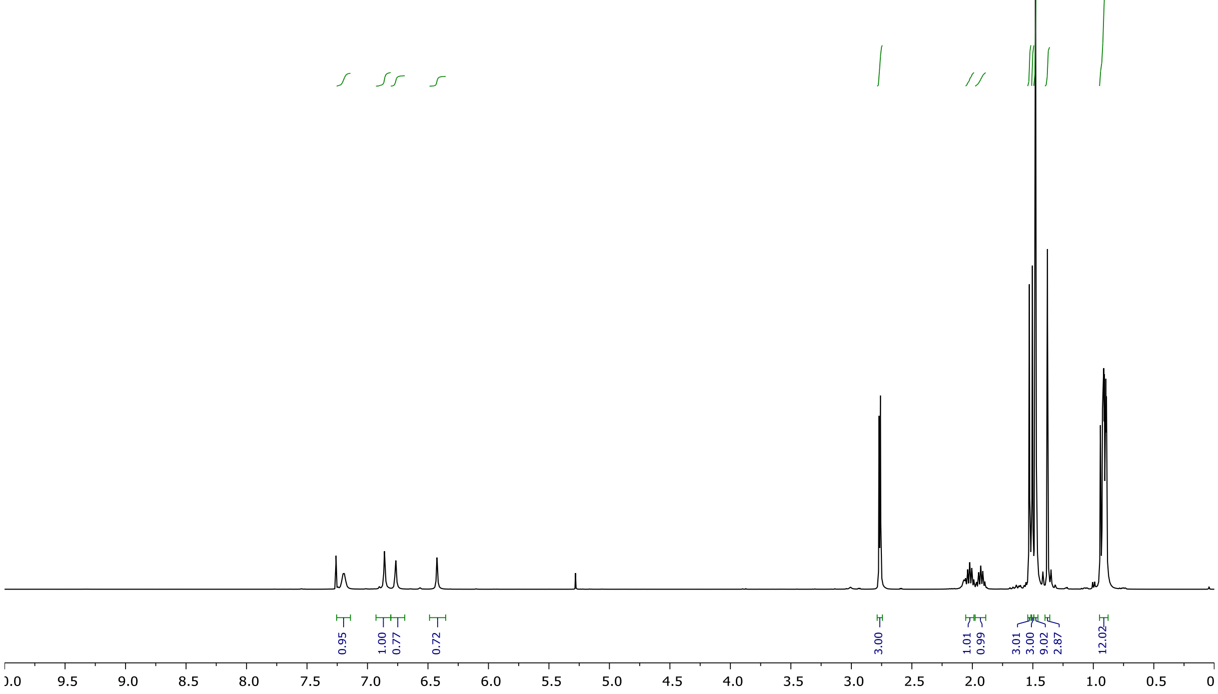
0.95
1.00
0.77
0.72
3.00
1.01
0.99
3.01
3.00
9.02
2.87
12.02
9.5
9.0
8.5
8.0
7.5
7.0
6.5
6.0
5.5
5.0
4.5
4.0
3.5
3.0
2.5
2.0
1.5
1.0
0.5

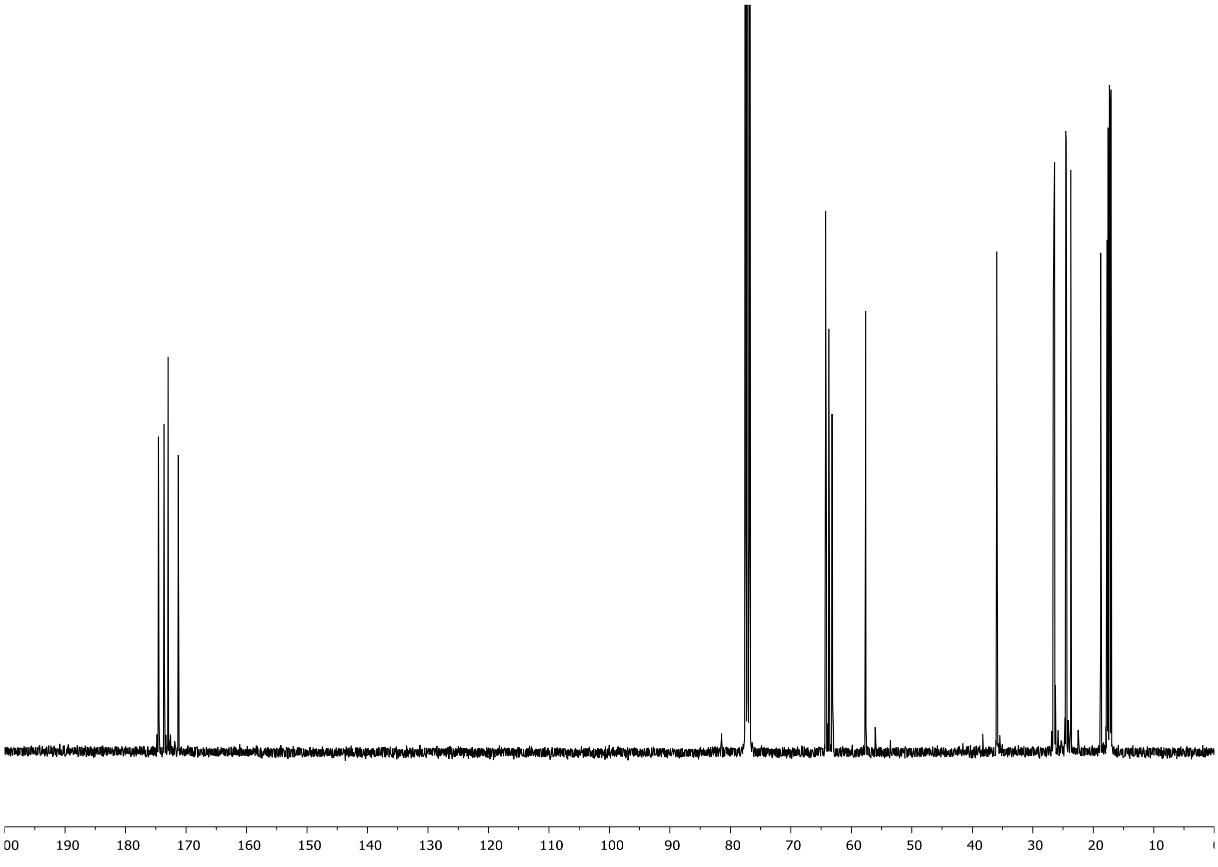
190
180
170
160
150
140
130
120
110
100
90
80
70
60
50
40
30
20
10

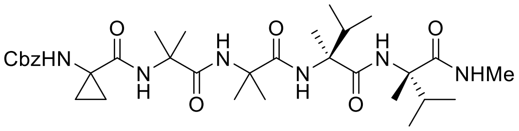
CbzHN
NHMe

S17

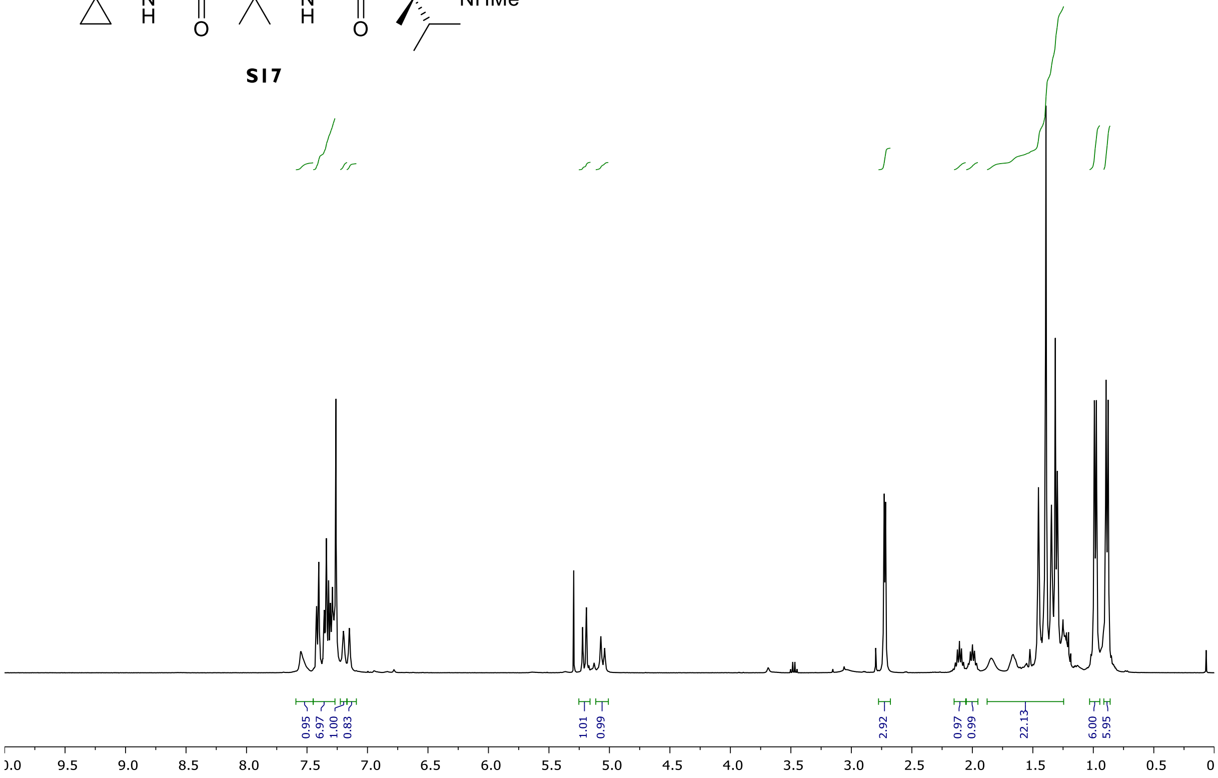
0.95
6.97
1.00
0.83
1.01
0.99
2.92
0.97
0.99
22.13
6.00
5.95
0.0 9.5 9.0 8.5 8.0 7.5 7.0 6.5 6.0 5.5 5.0 4.5 4.0 3.5 3.0 2.5 2.0 1.5 1.0 0.5 0

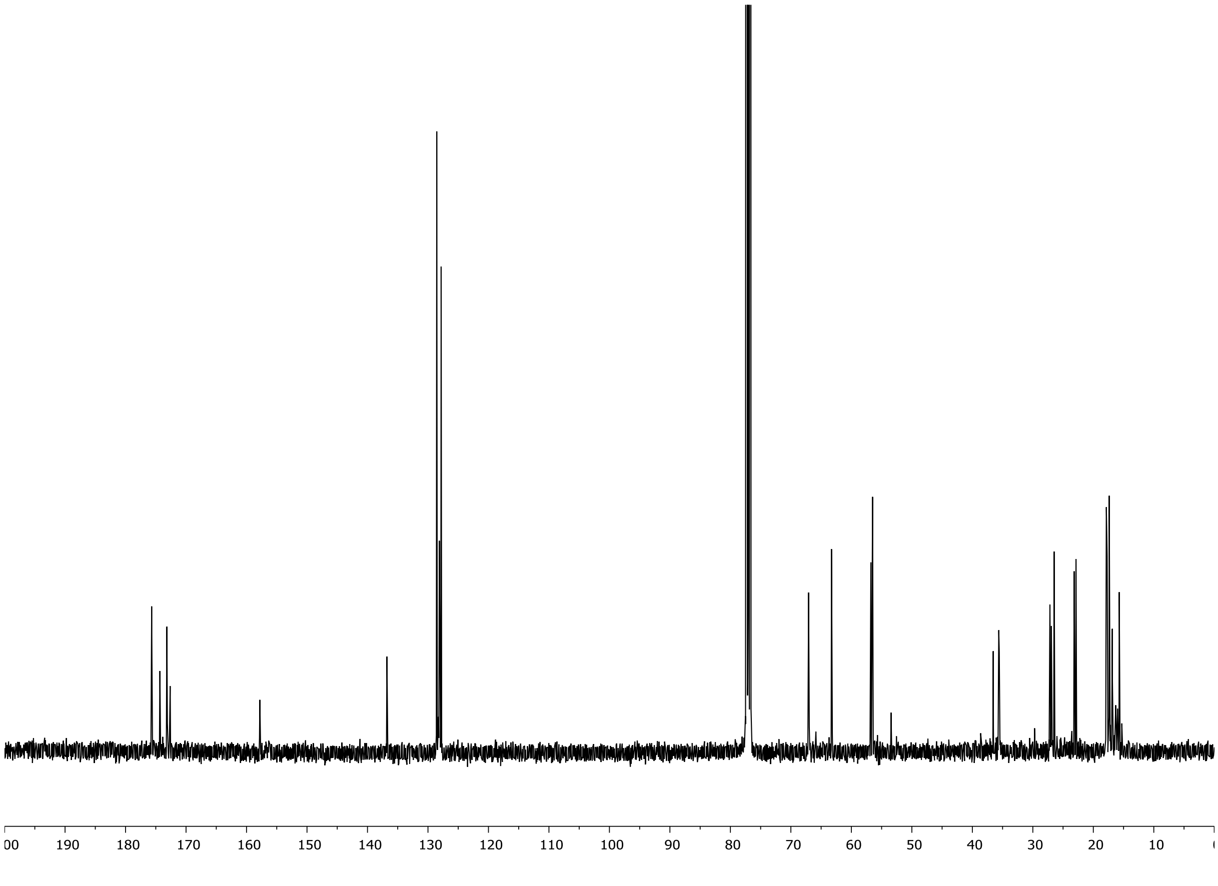
00 190 180 170 160 150 140 130 120 110 100 90 80 70 60 50 40 30 20 10

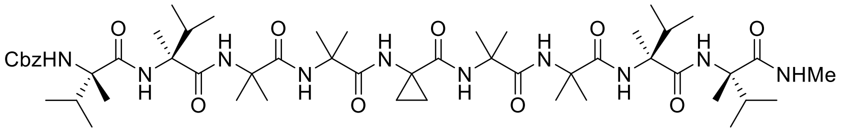

2a

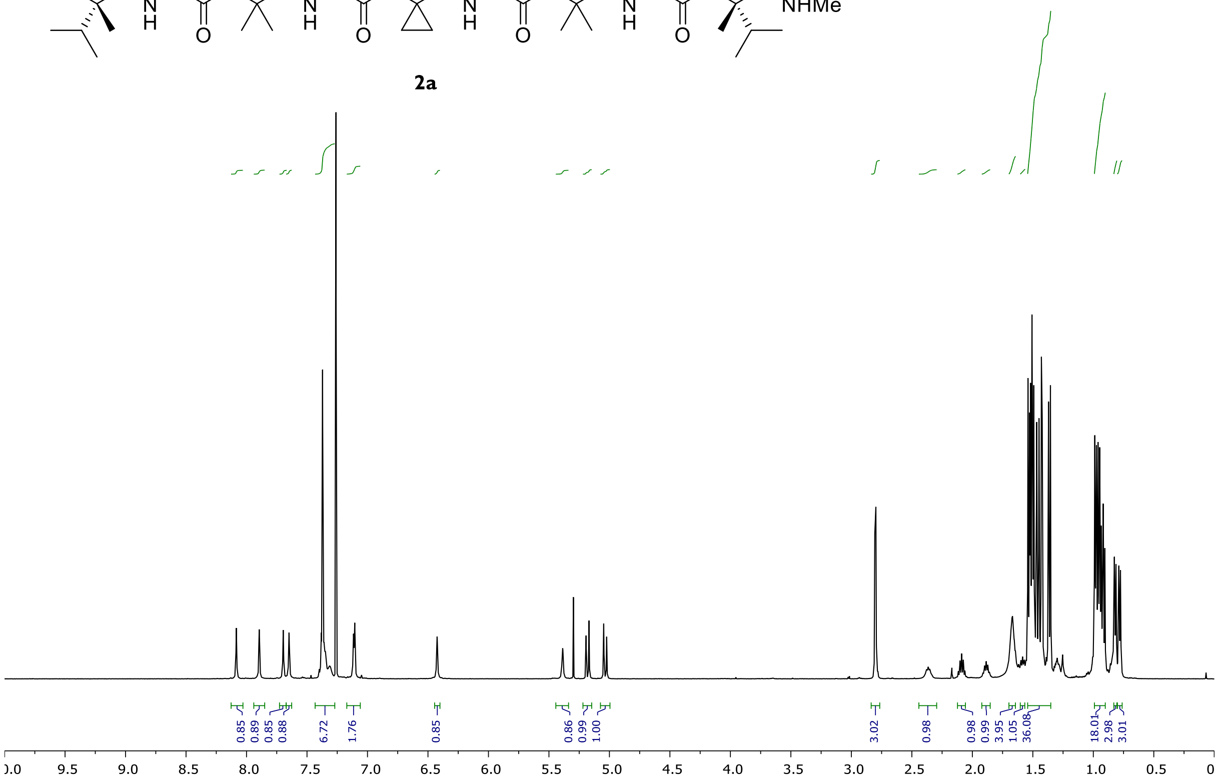

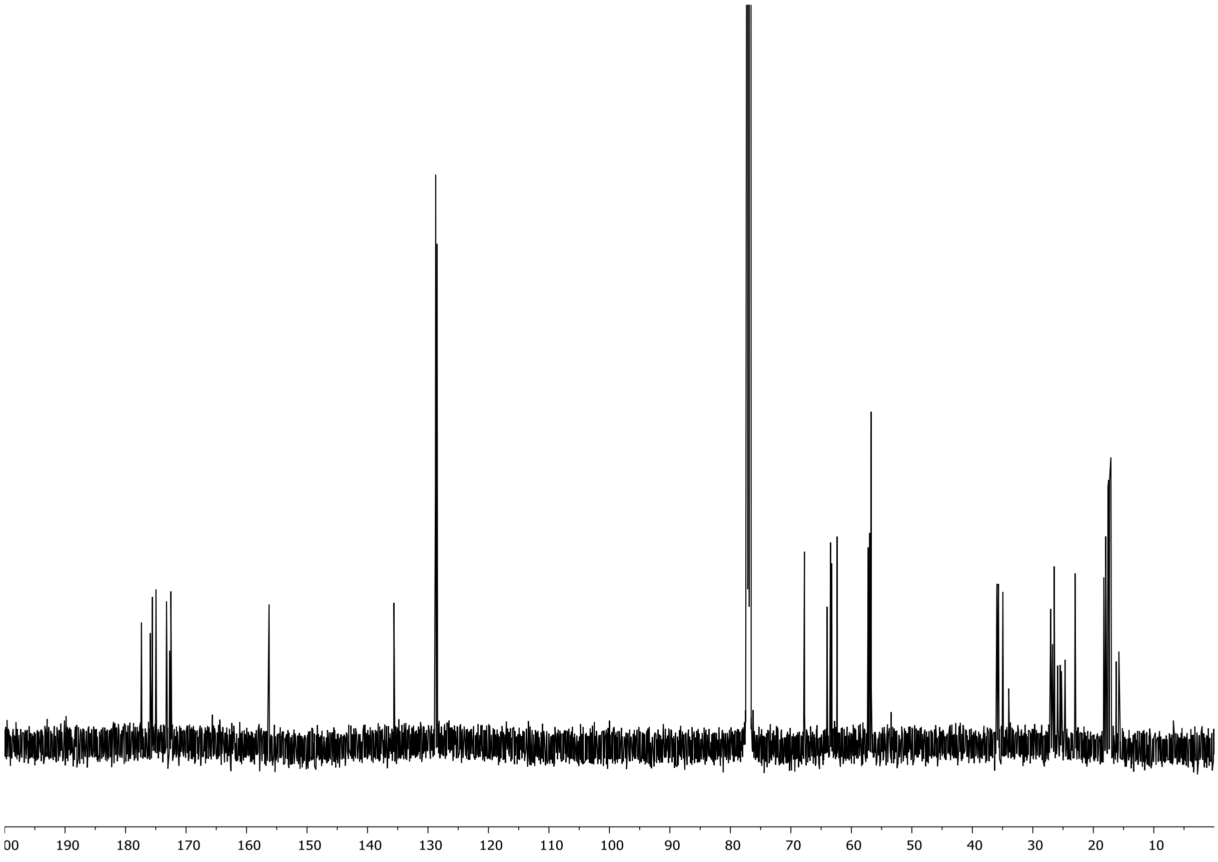

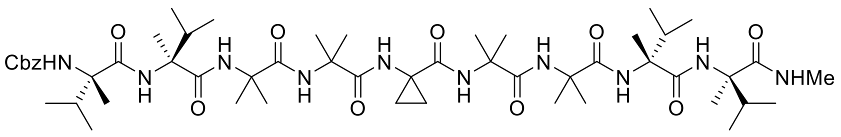

2b

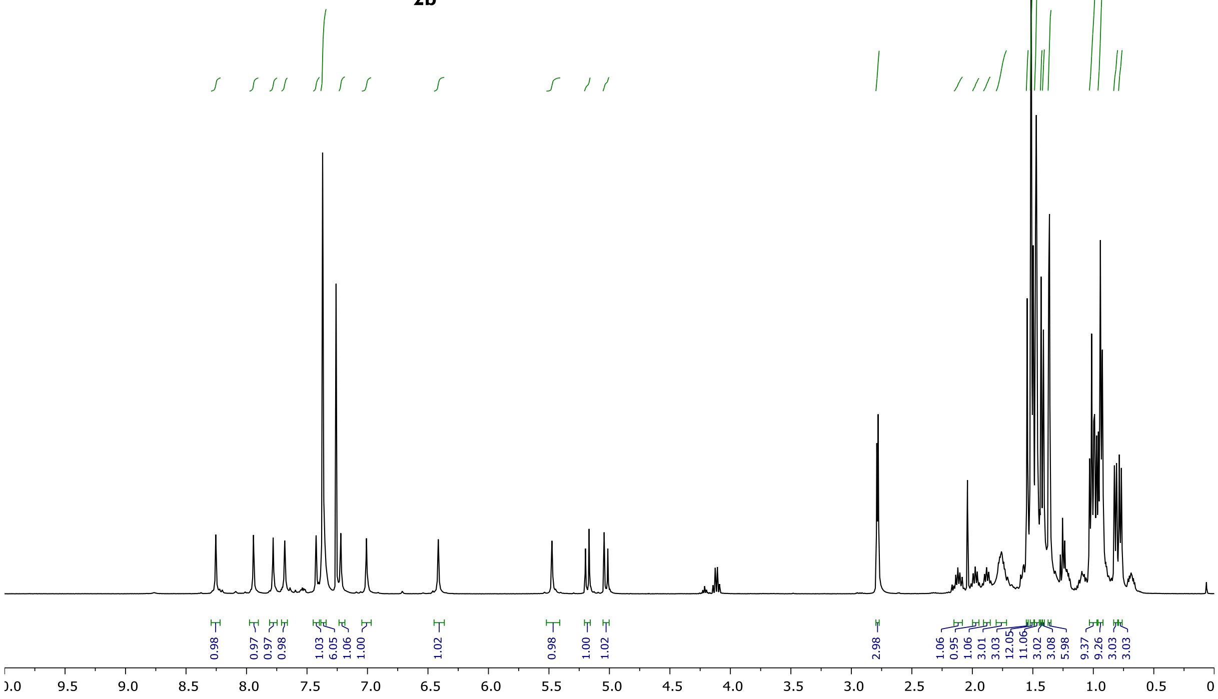

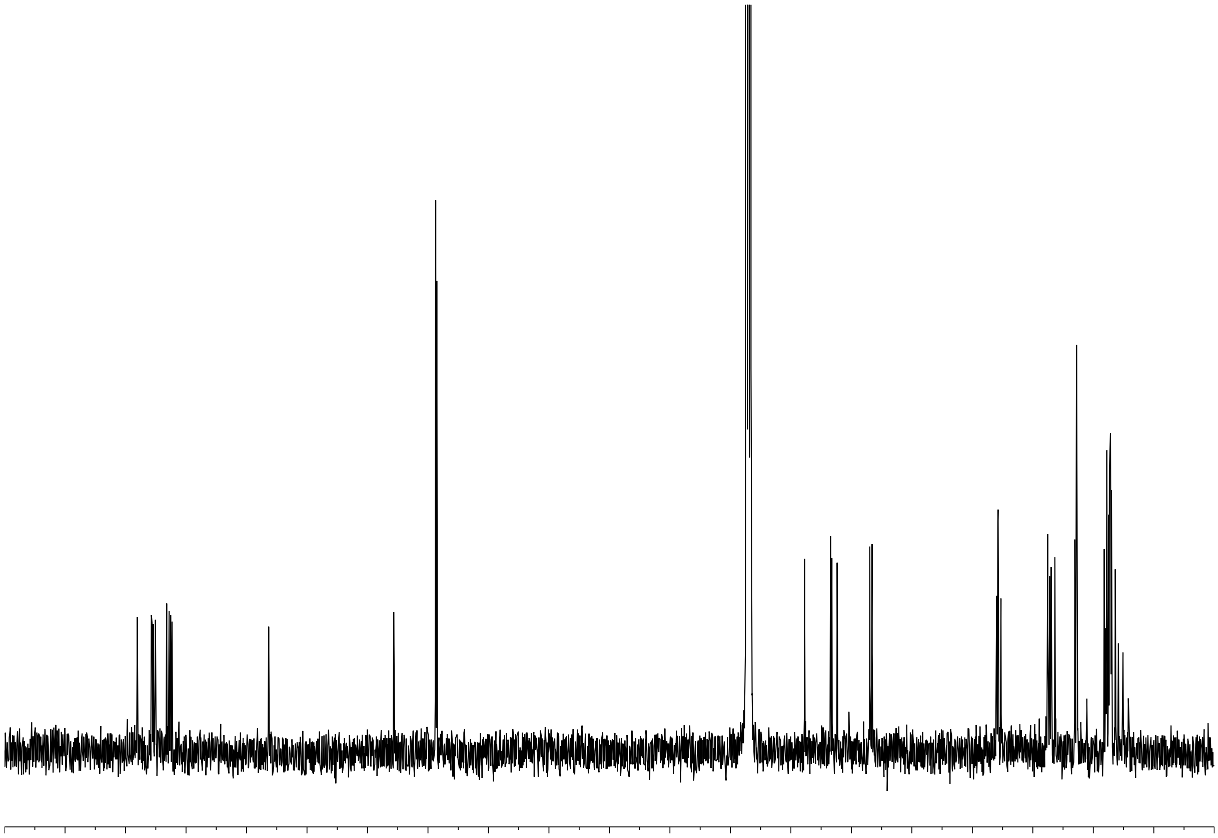

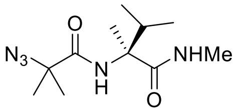
N3
O
N
H
NHMe
O

S19

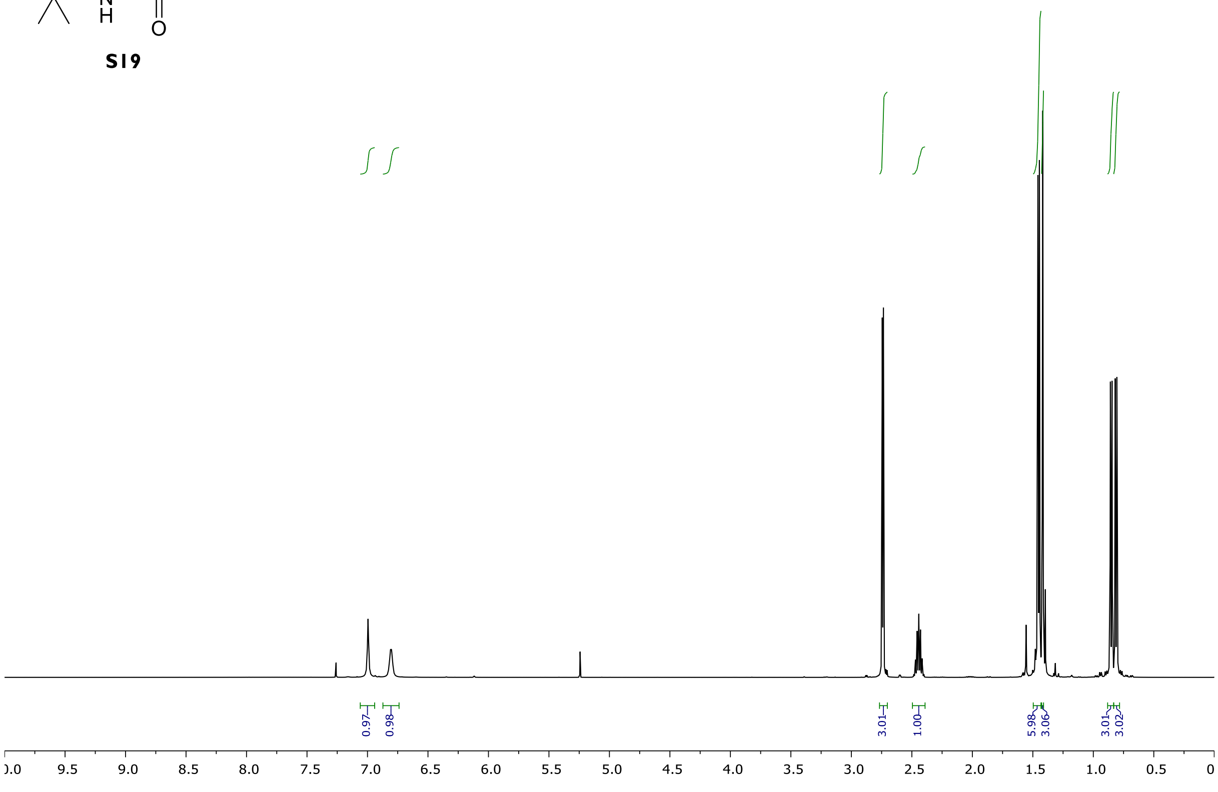
0.97
0.98
3.01
1.00
5.98
3.06
3.01
3.02
10.0 9.5 9.0 8.5 8.0 7.5 7.0 6.5 6.0 5.5 5.0 4.5 4.0 3.5 3.0 2.5 2.0 1.5 1.0 0.5 0

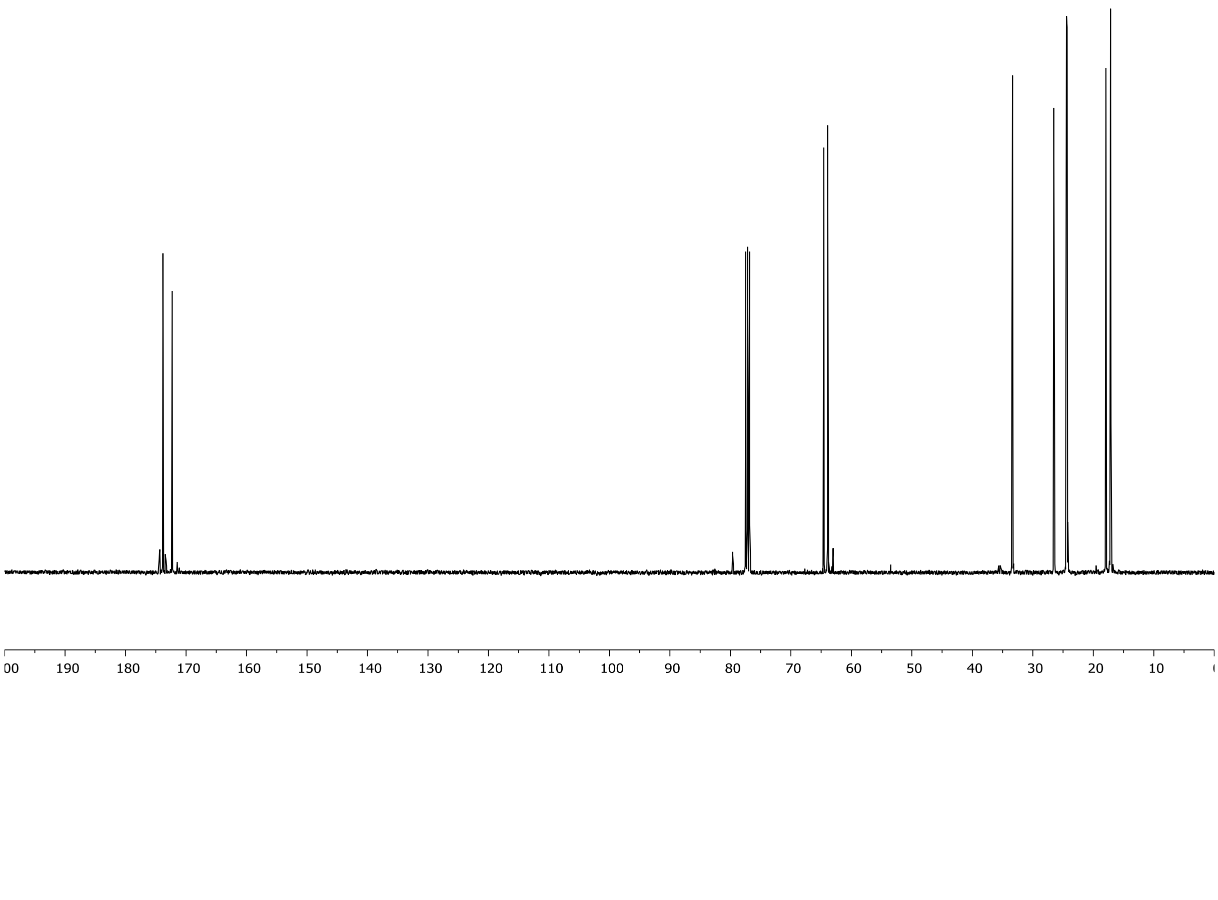
200 190 180 170 160 150 140 130 120 110 100 90 80 70 60 50 40 30 20 10 0

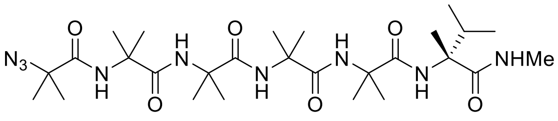

S22

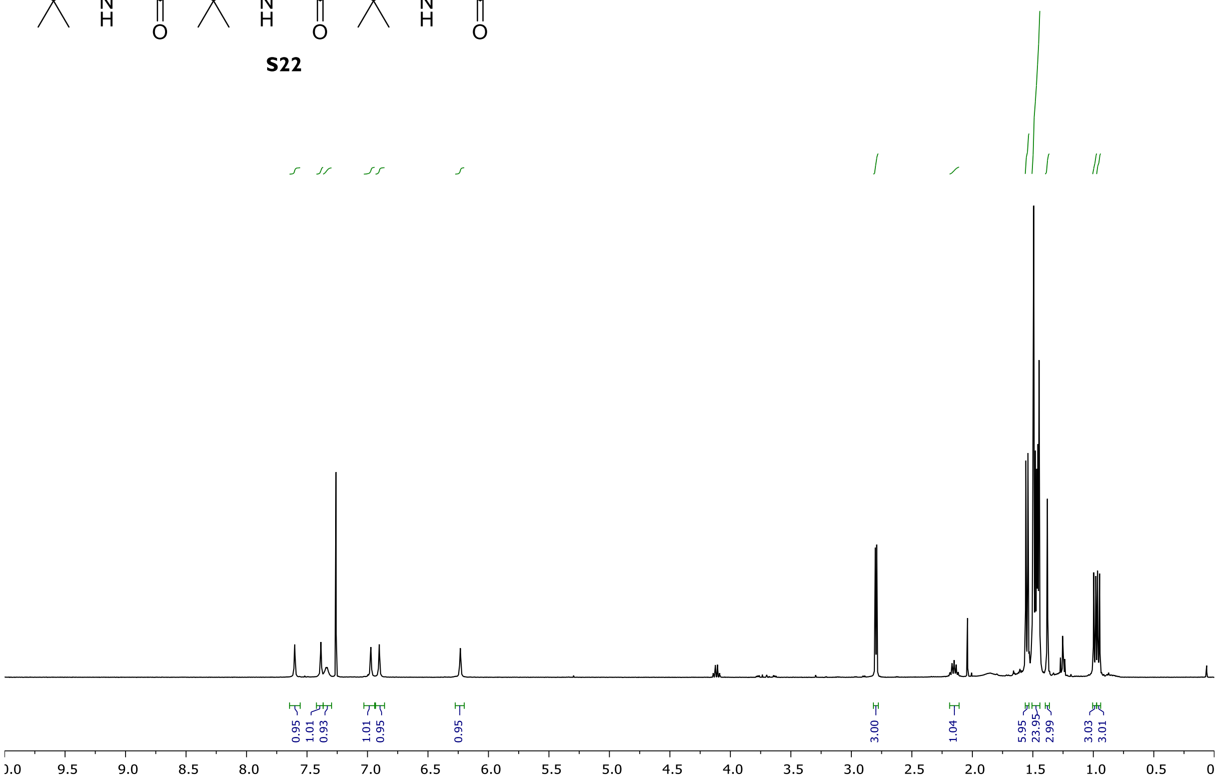

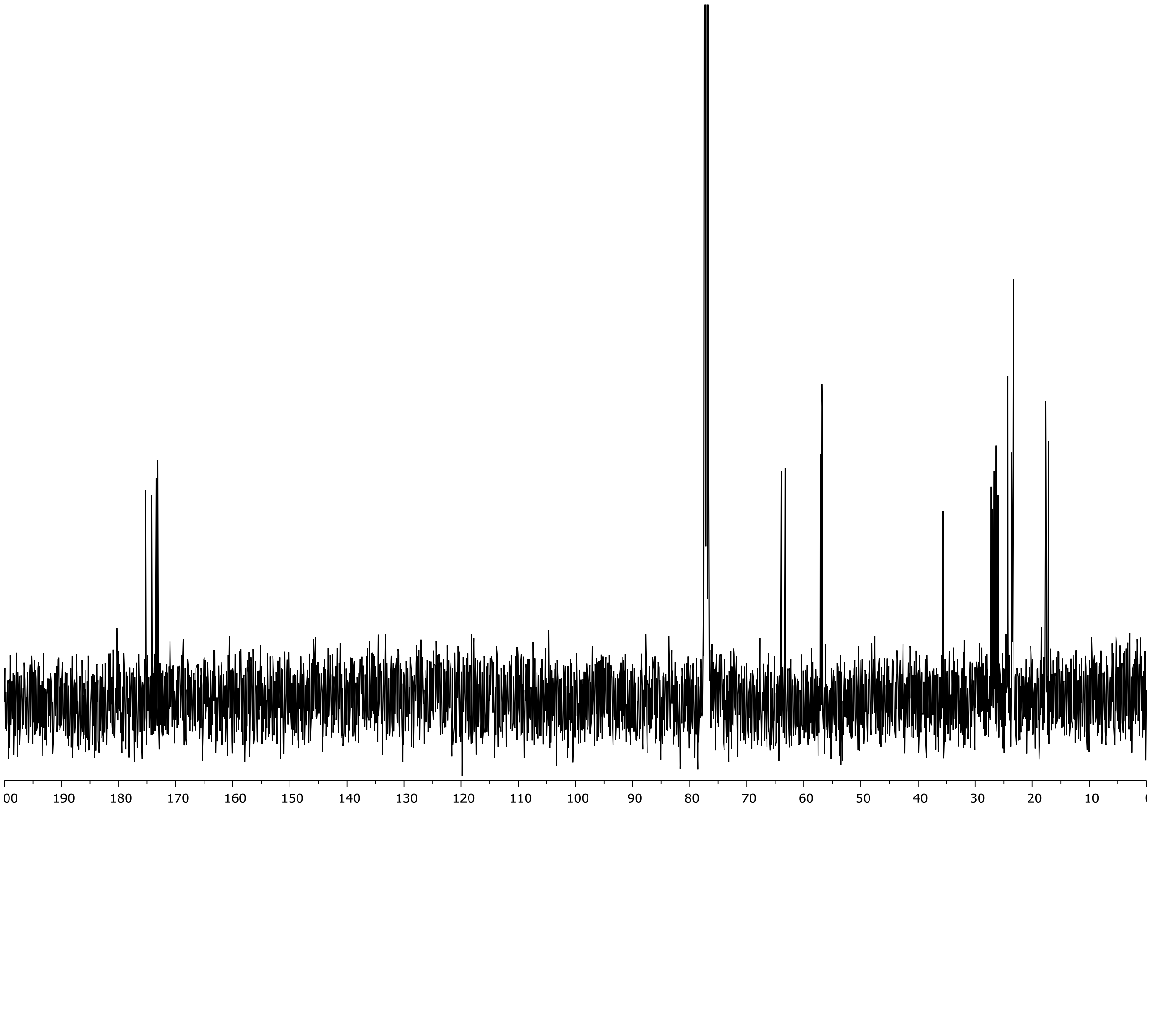

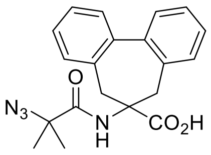

S26

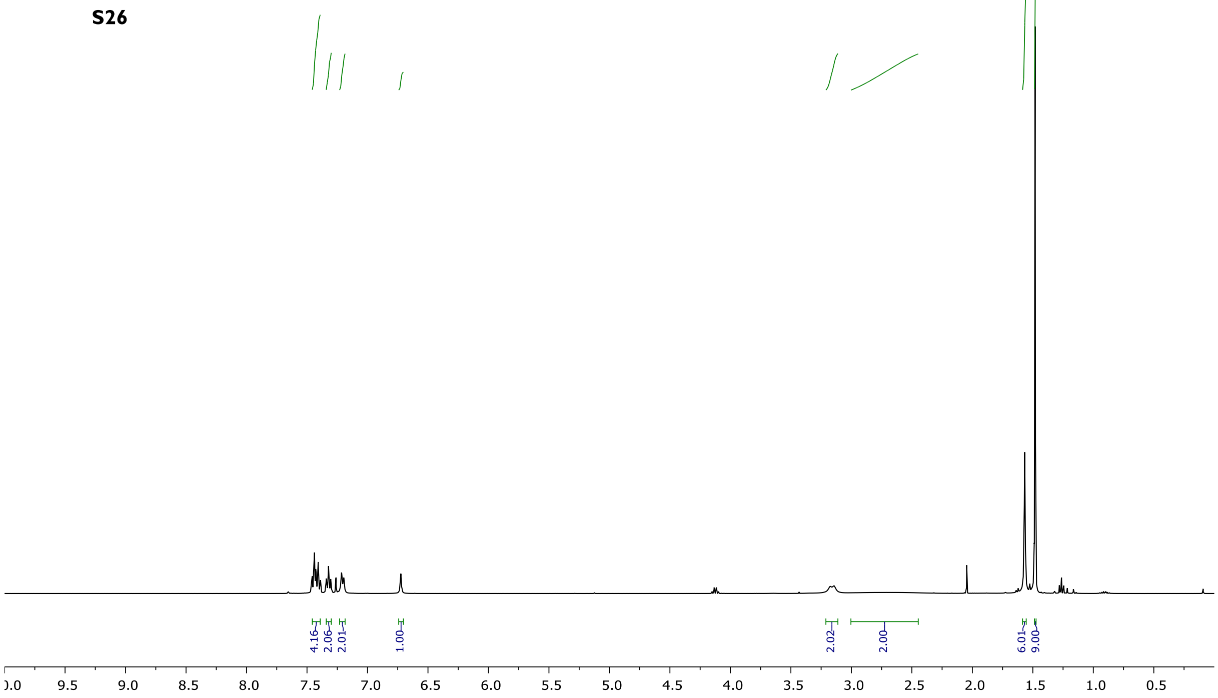

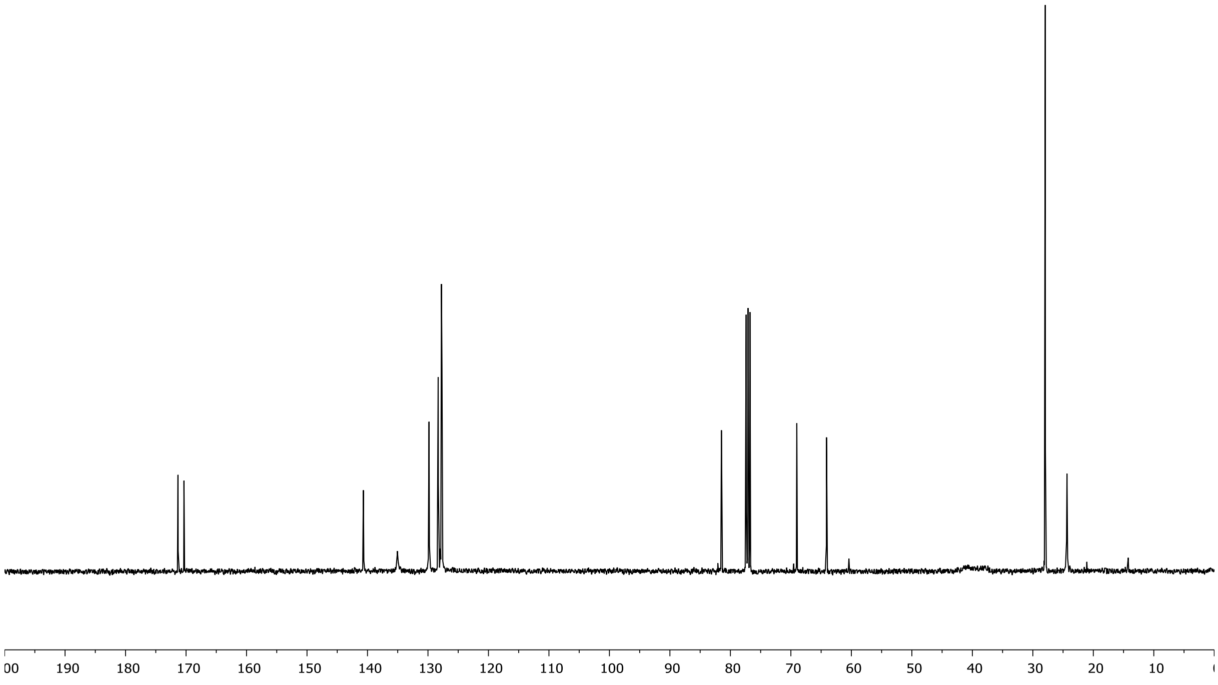

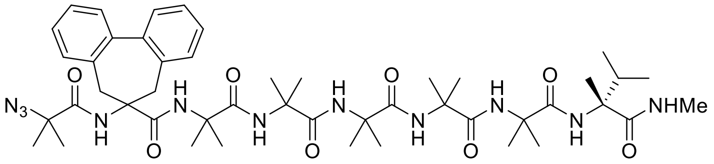

S27

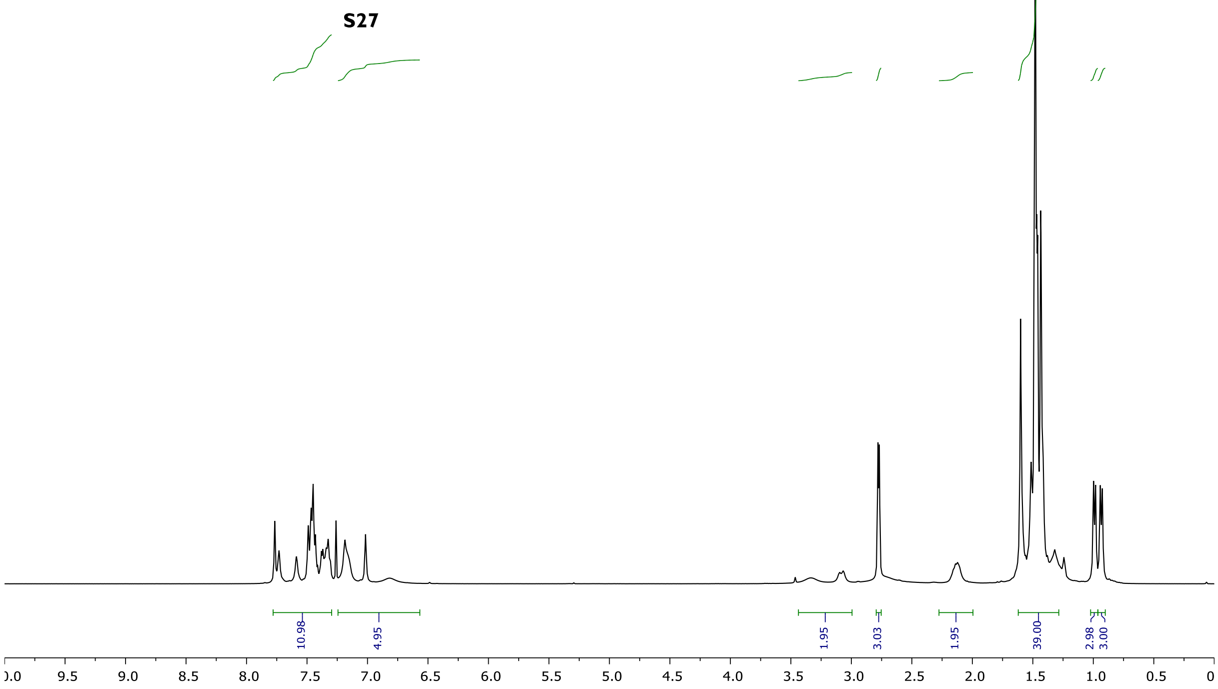

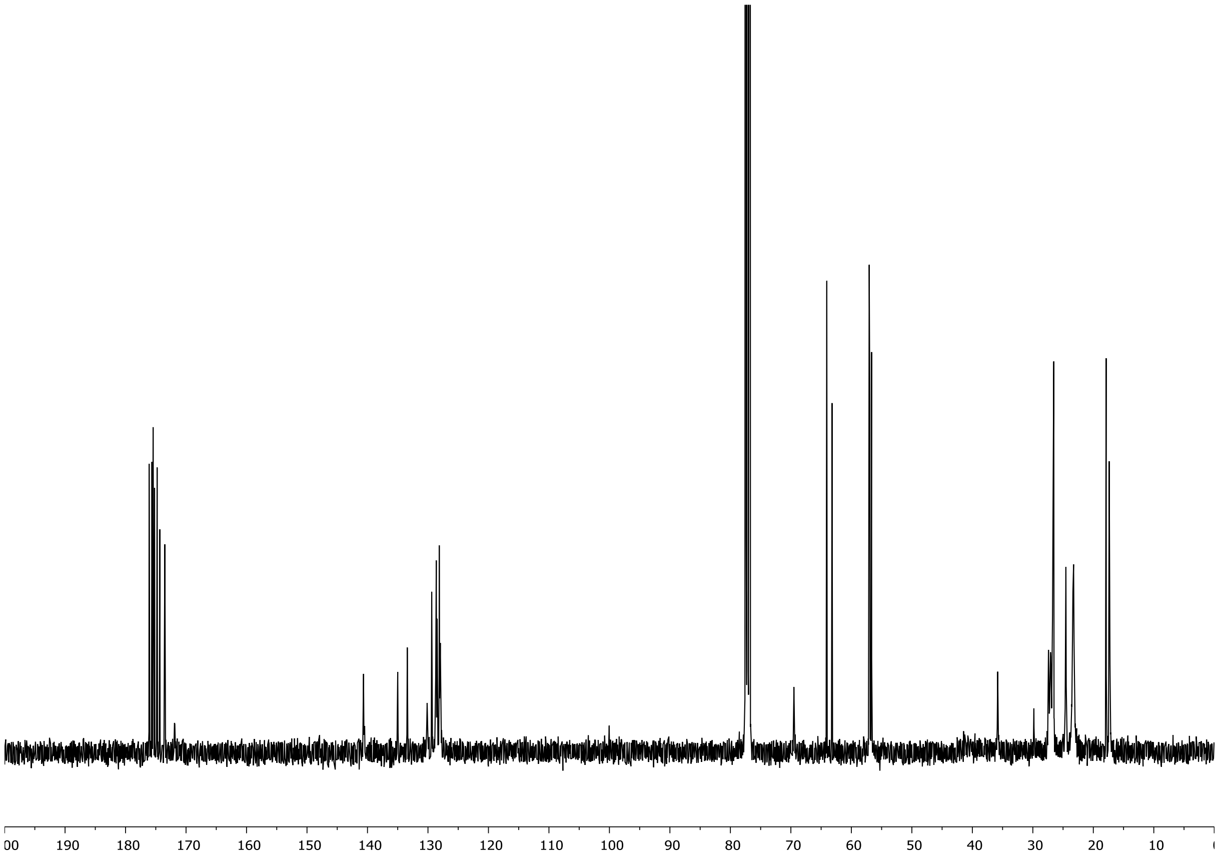

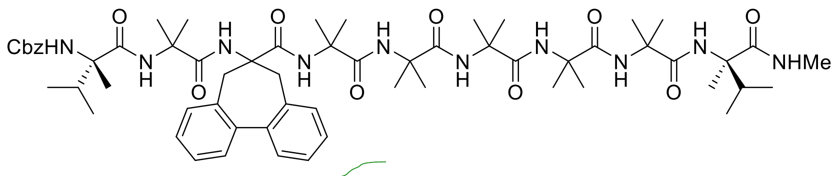
CbzHN
NHMe

4a

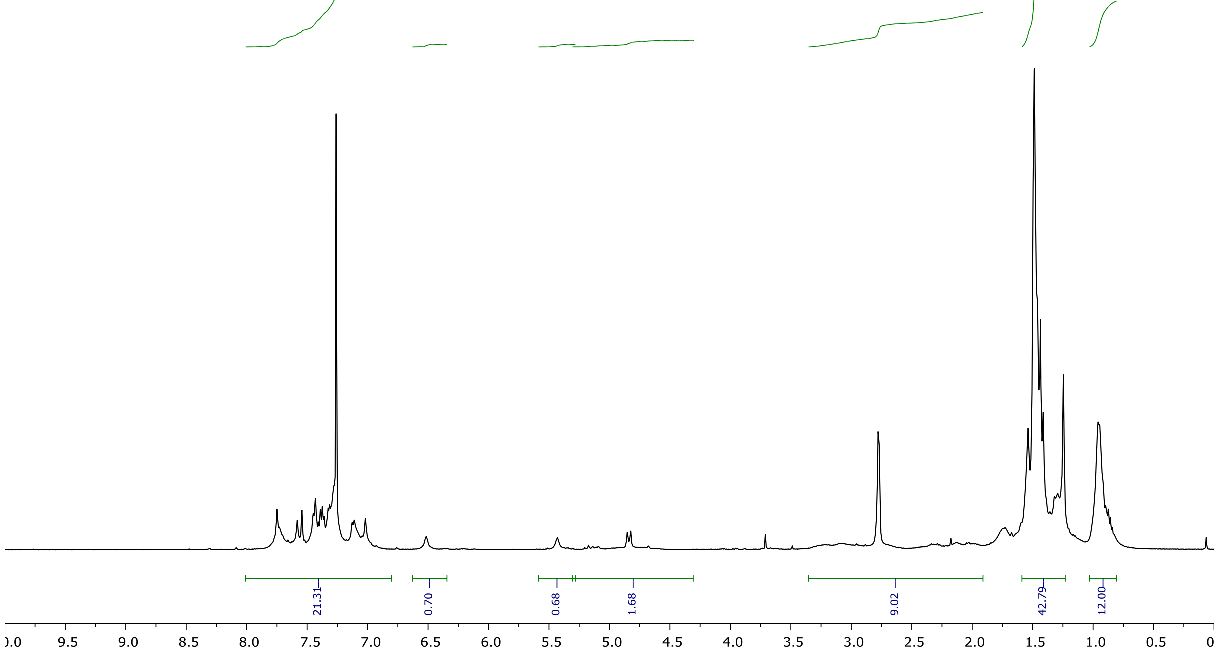
21.31
0.70
0.68
1.68
9.02
42.79
12.00
0.0 9.5 9.0 8.5 8.0 7.5 7.0 6.5 6.0 5.5 5.0 4.5 4.0 3.5 3.0 2.5 2.0 1.5 1.0 0.5 0

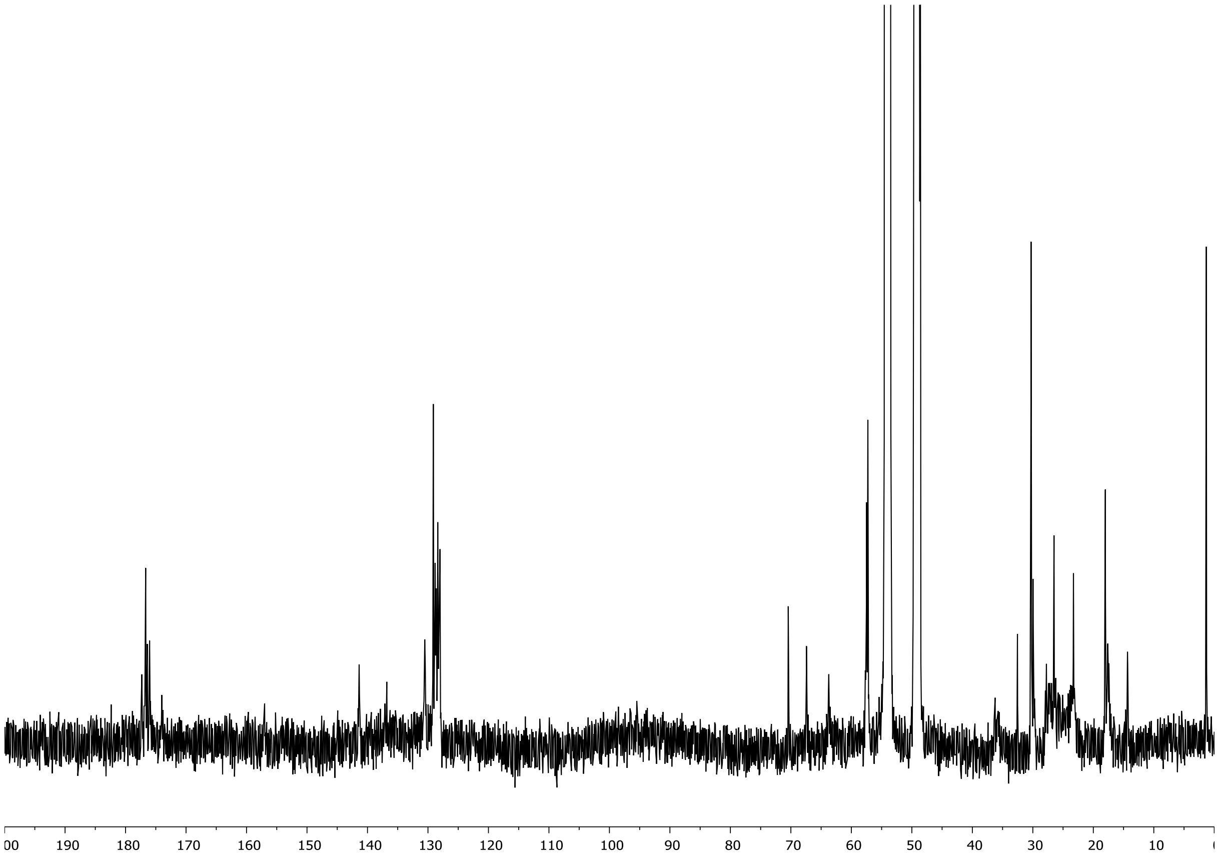
00 190 180 170 160 150 140 130 120 110 100 90 80 70 60 50 40 30 20 10

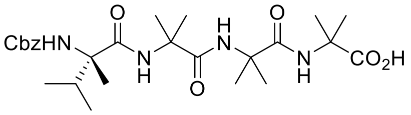
CbzHN
O
H
N
O
N
H
O
N
H
CO_2H

S30

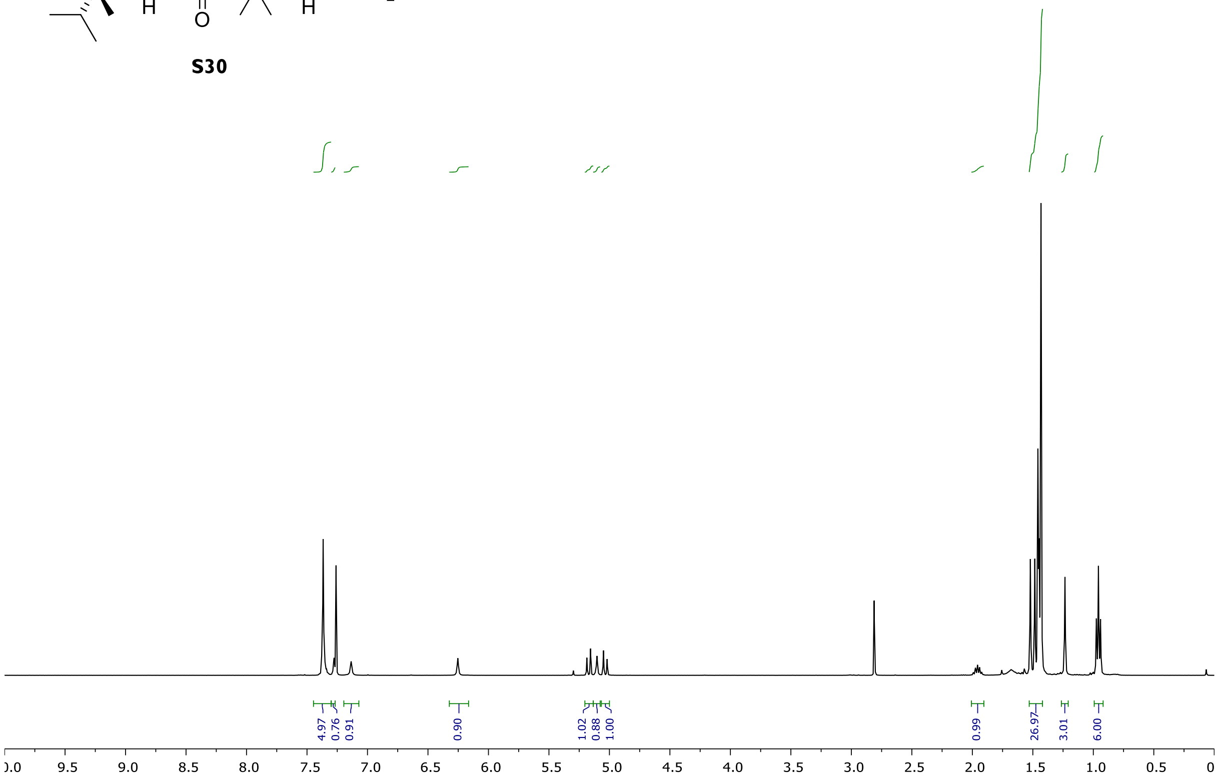
4.97
0.76
0.91
0.90
1.02
0.88
1.00
0.99
26.97
3.01
6.00
9.5 9.0 8.5 8.0 7.5 7.0 6.5 6.0 5.5 5.0 4.5 4.0 3.5 3.0 2.5 2.0 1.5 1.0 0.5

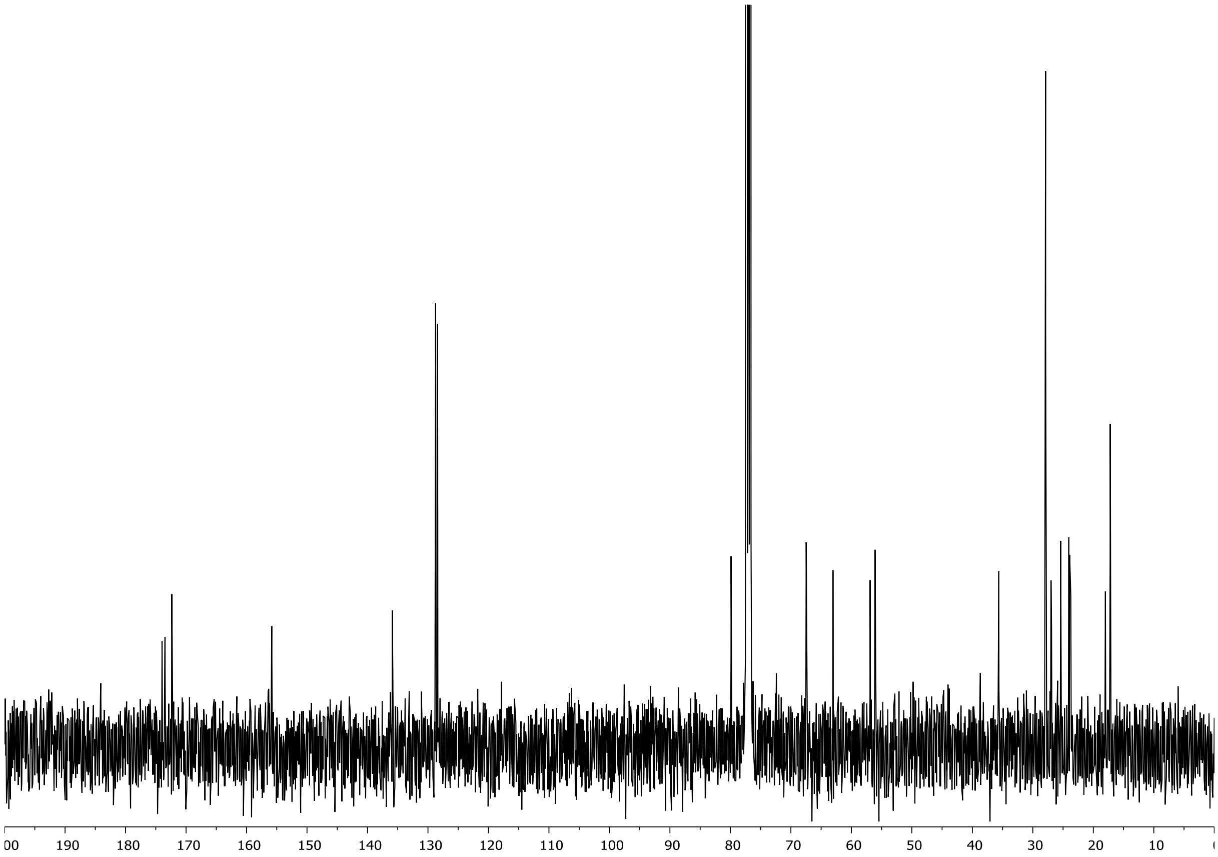
190 180 170 160 150 140 130 120 110 100 90 80 70 60 50 40 30 20 10

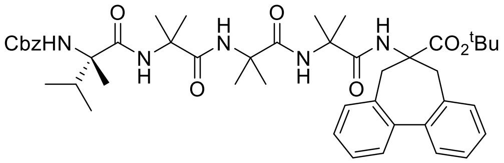

S31

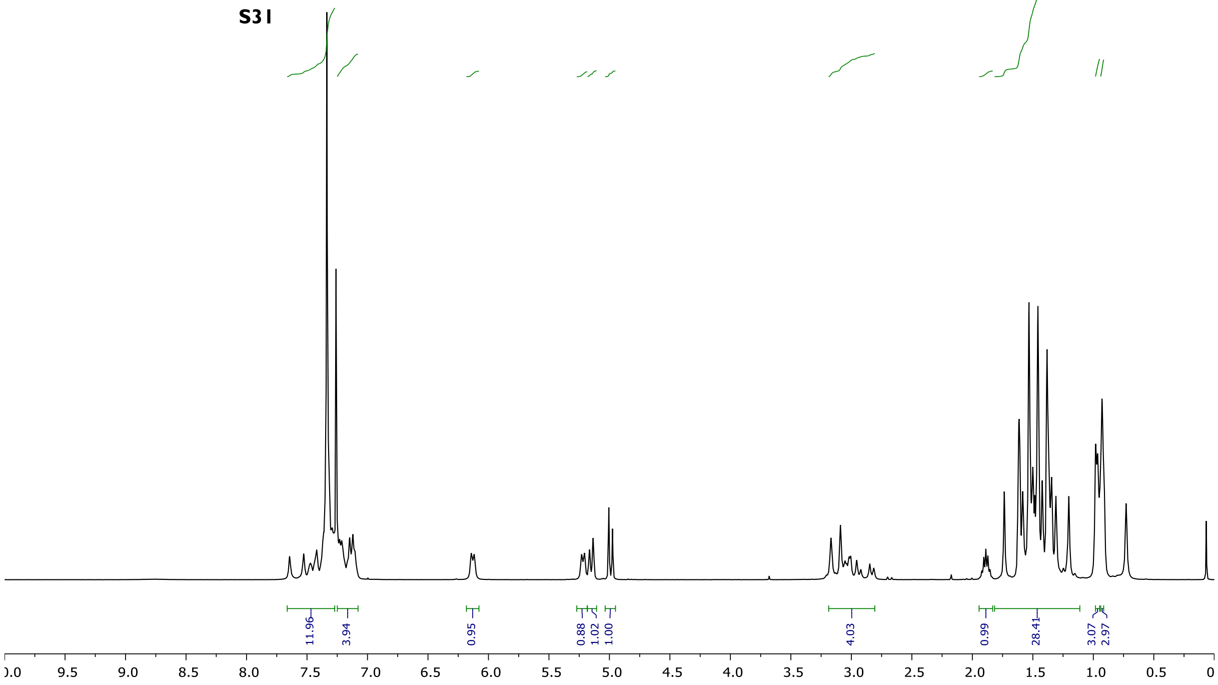

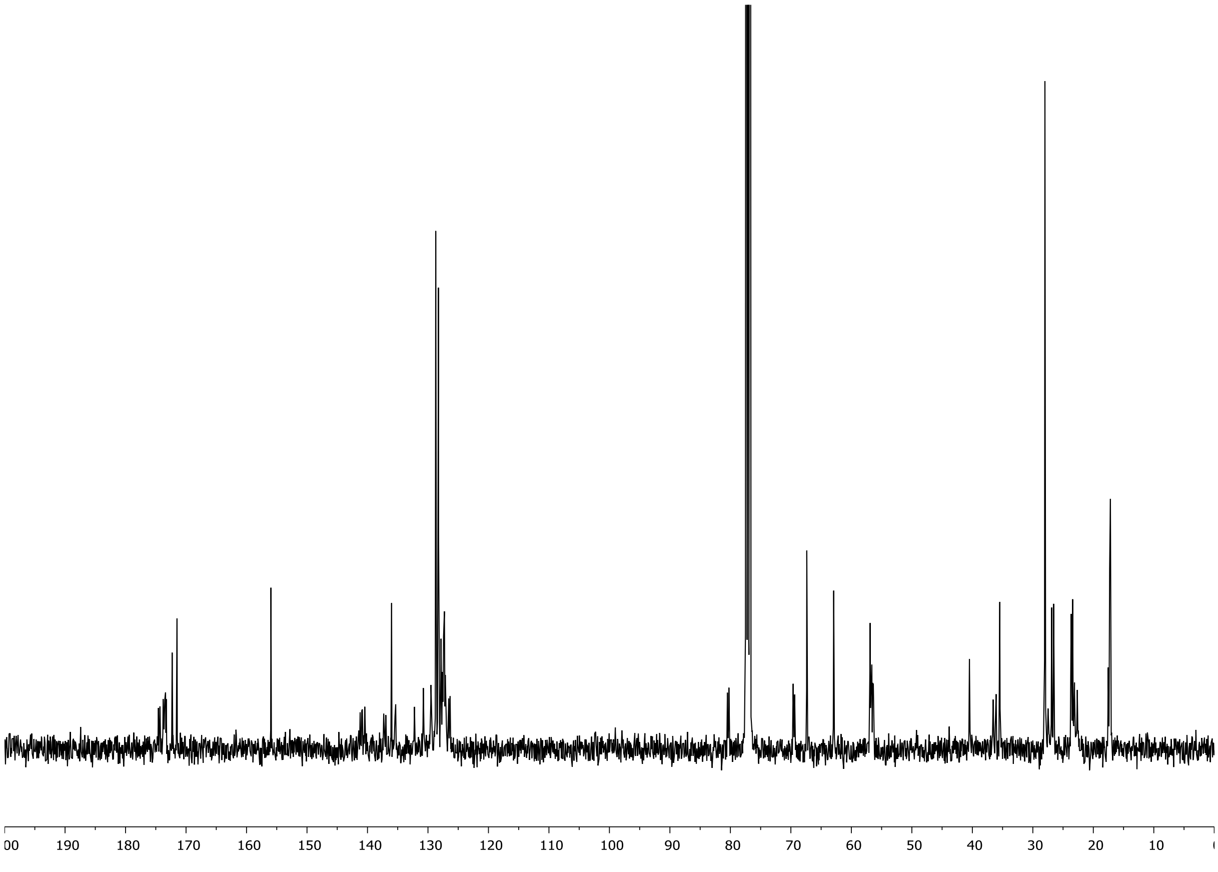

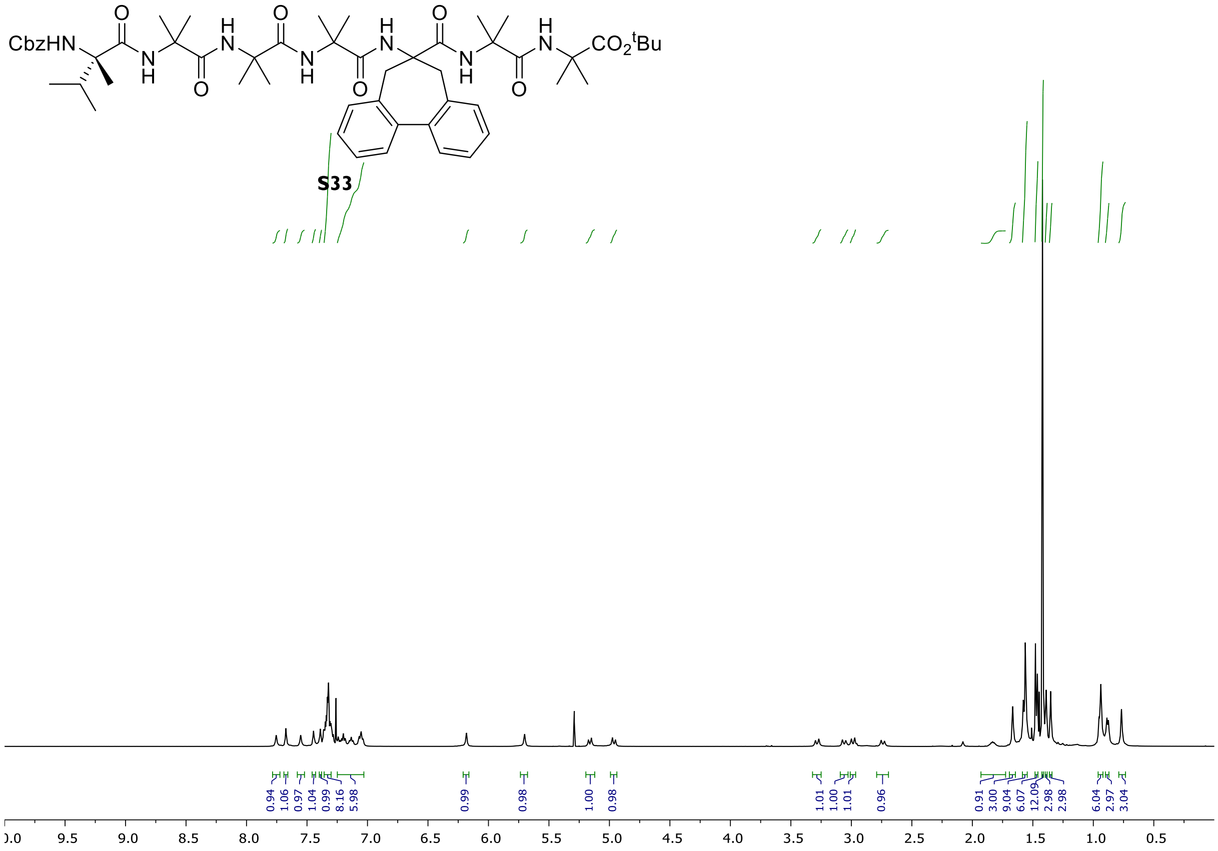
CbzHN
CO2tBu
S33
9.5 9.0 8.5 8.0 7.5 7.0 6.5 6.0 5.5 5.0 4.5 4.0 3.5 3.0 2.5 2.0 1.5 1.0 0.5

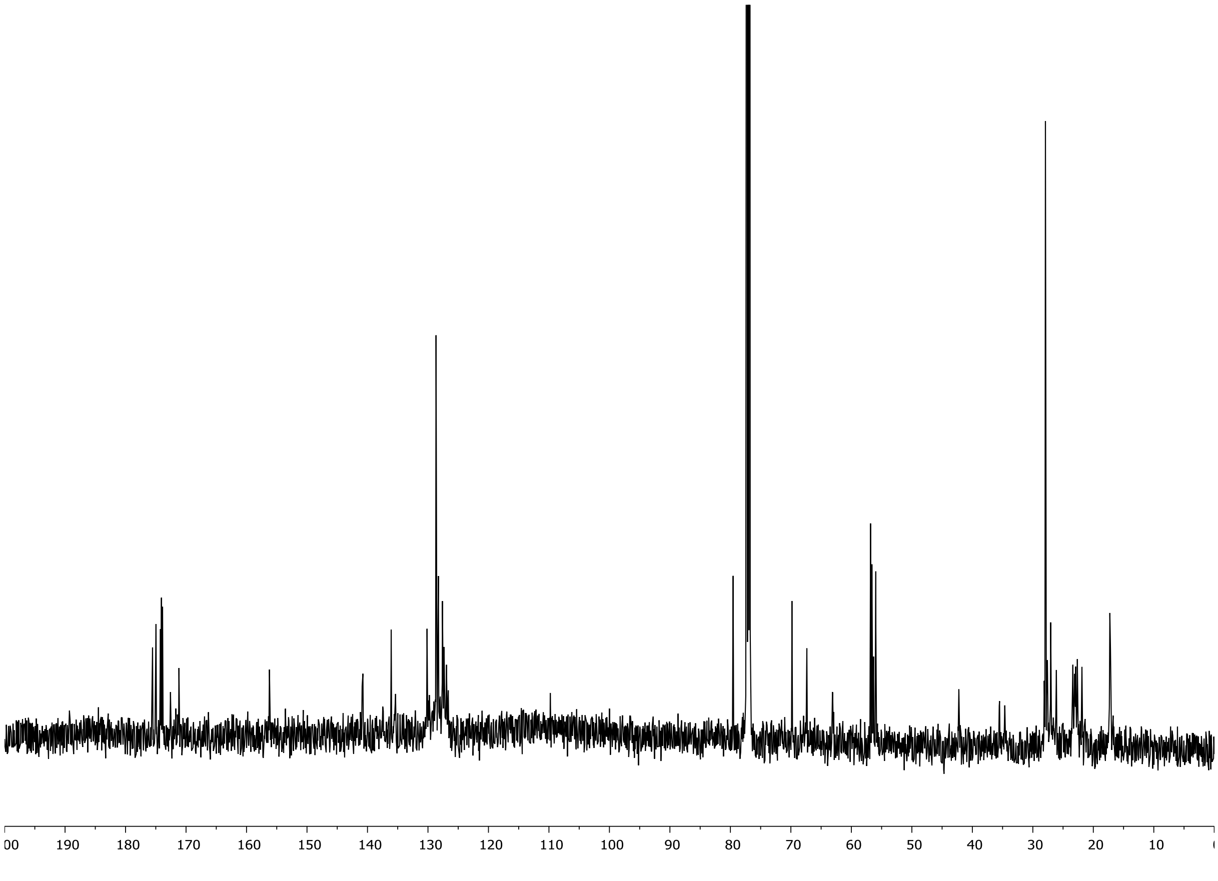
190 180 170 160 150 140 130 120 110 100 90 80 70 60 50 40 30 20 10

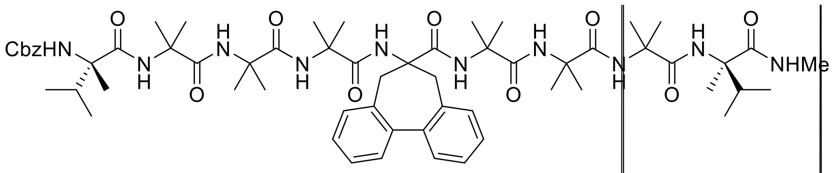
CbzHN
NHMe

4b

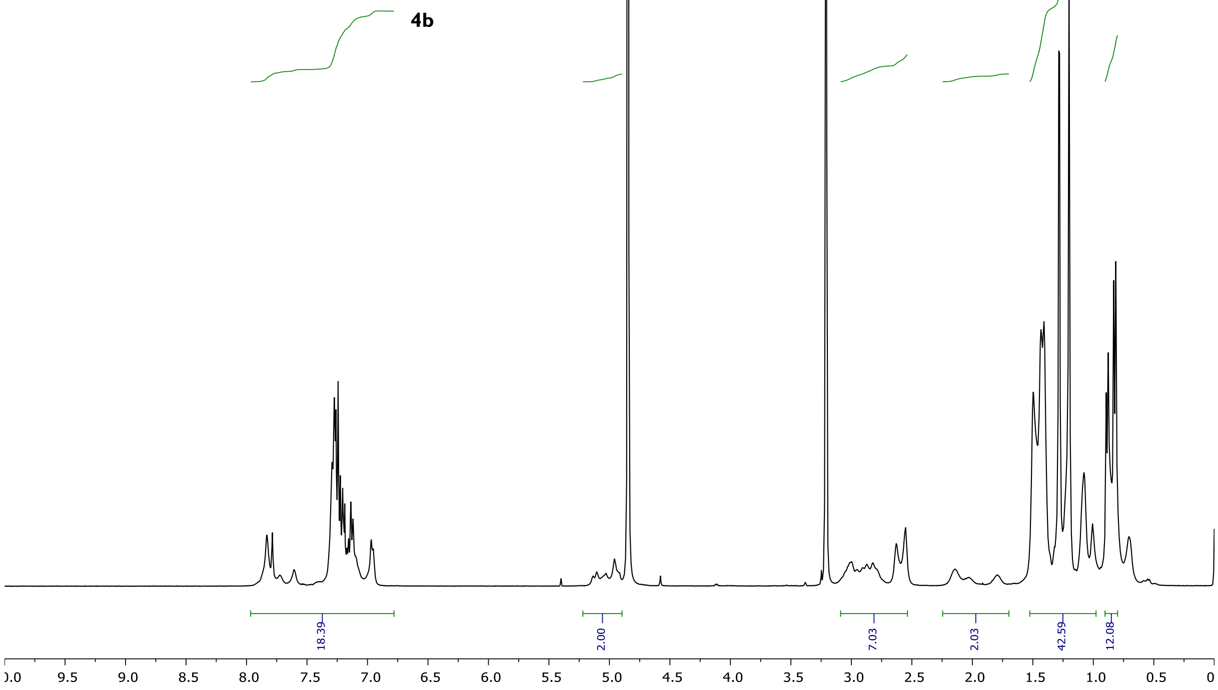
18.39
2.00
7.03
2.03
42.59
12.08
0.0 9.5 9.0 8.5 8.0 7.5 7.0 6.5 6.0 5.5 5.0 4.5 4.0 3.5 3.0 2.5 2.0 1.5 1.0 0.5 0

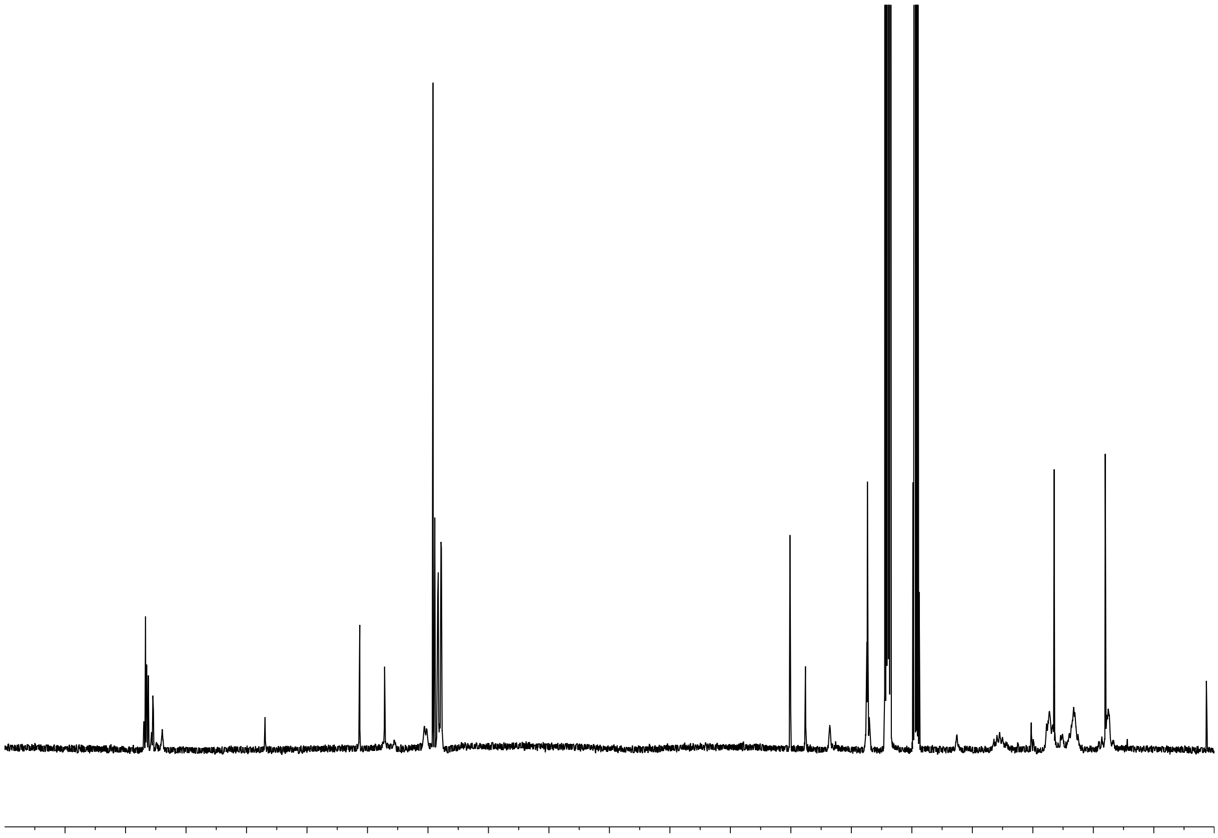
190 180 170 160 150 140 130 120 110 100 90 80 70 60 50 40 30 20 10

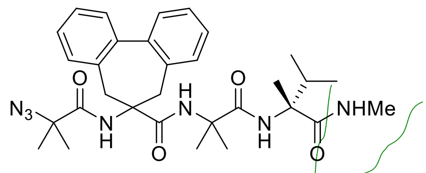

S35

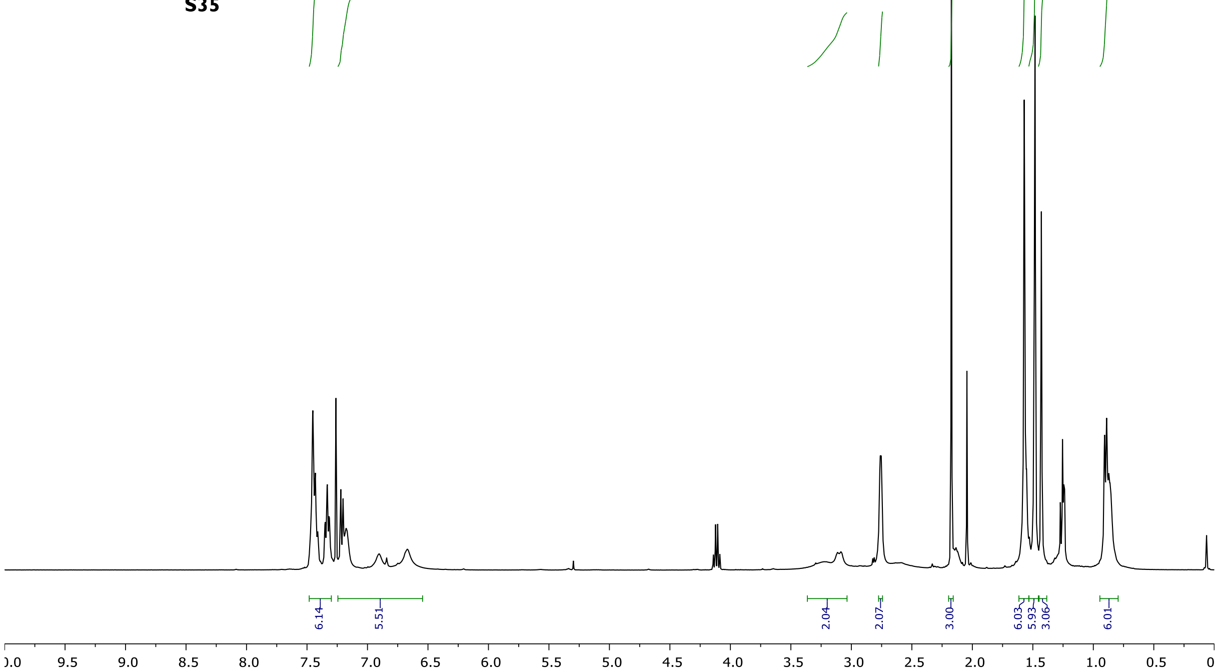

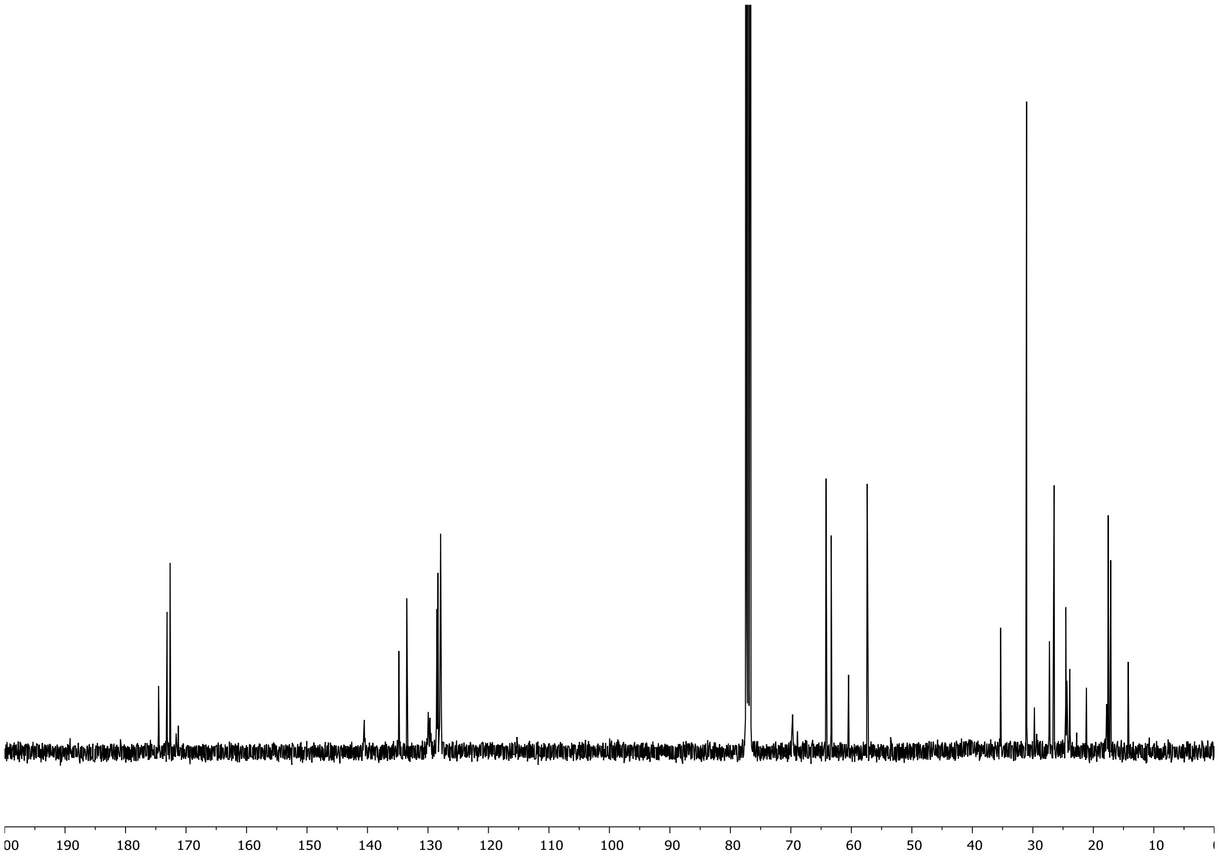

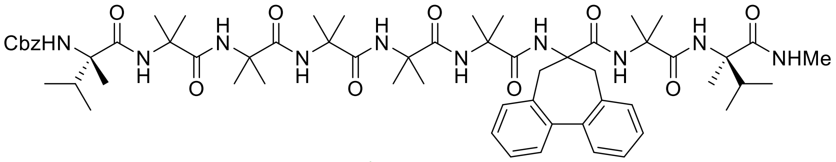

4c

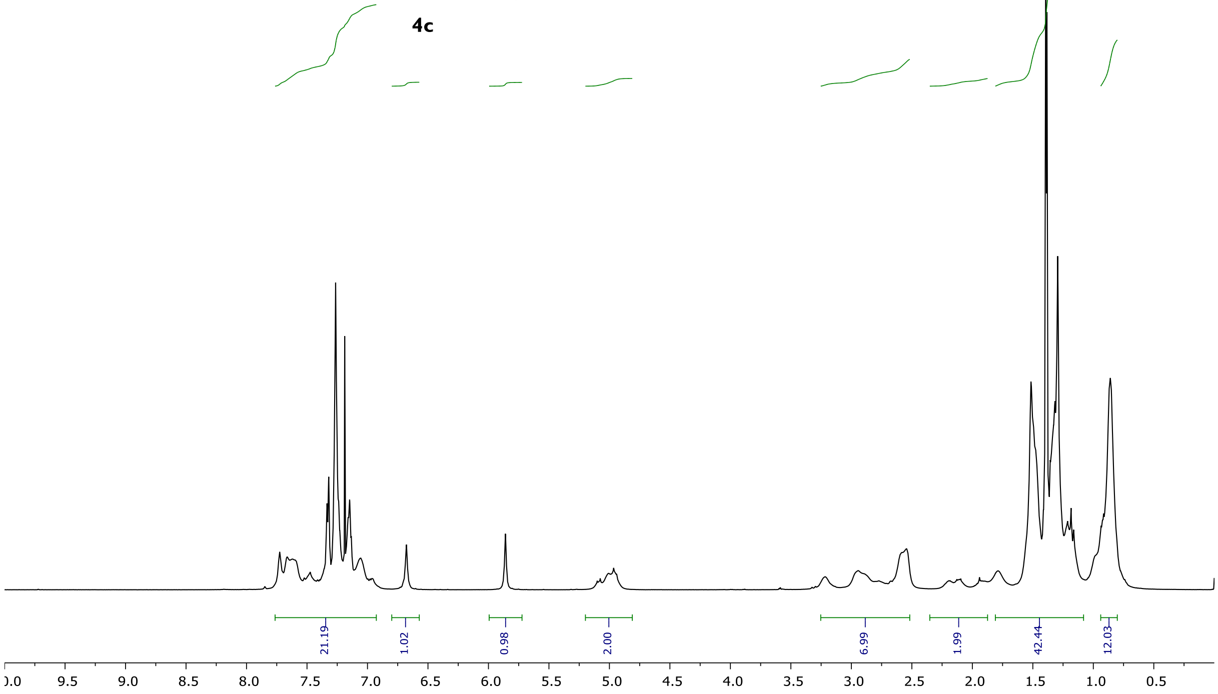

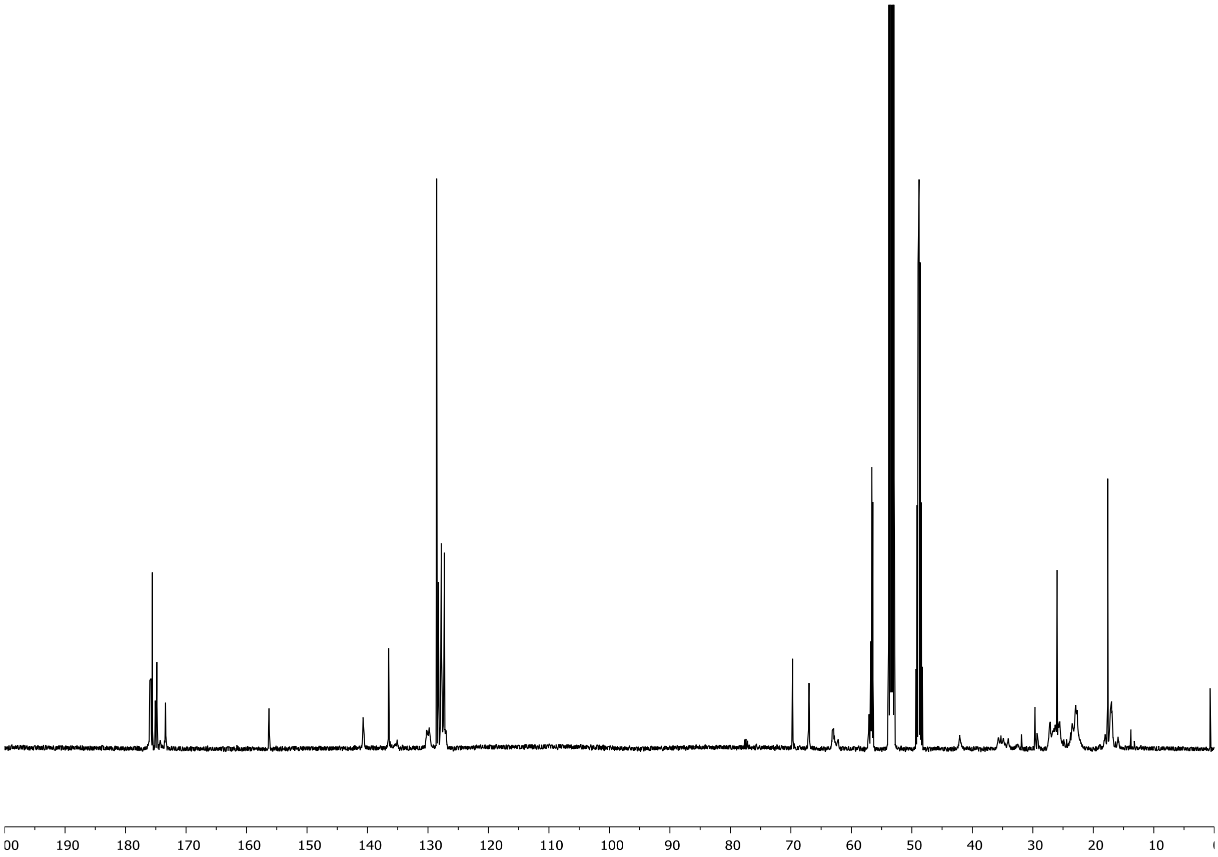

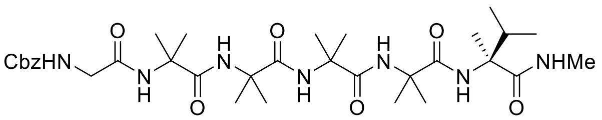
CbzHN
NHMe

S41

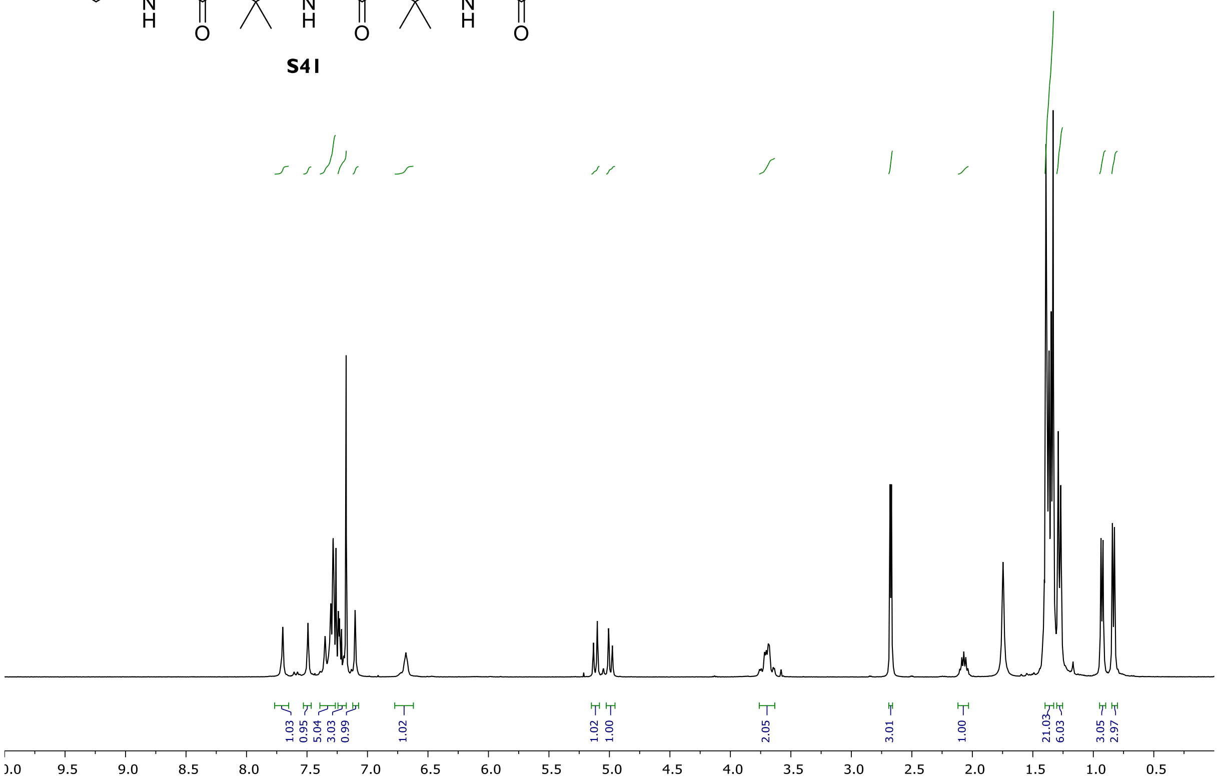
1.03
0.95
5.04
3.03
0.99
1.02
1.02
1.00
2.05
3.01
1.00
21.03
6.03
3.05
2.97
9.5 9.0 8.5 8.0 7.5 7.0 6.5 6.0 5.5 5.0 4.5 4.0 3.5 3.0 2.5 2.0 1.5 1.0 0.5

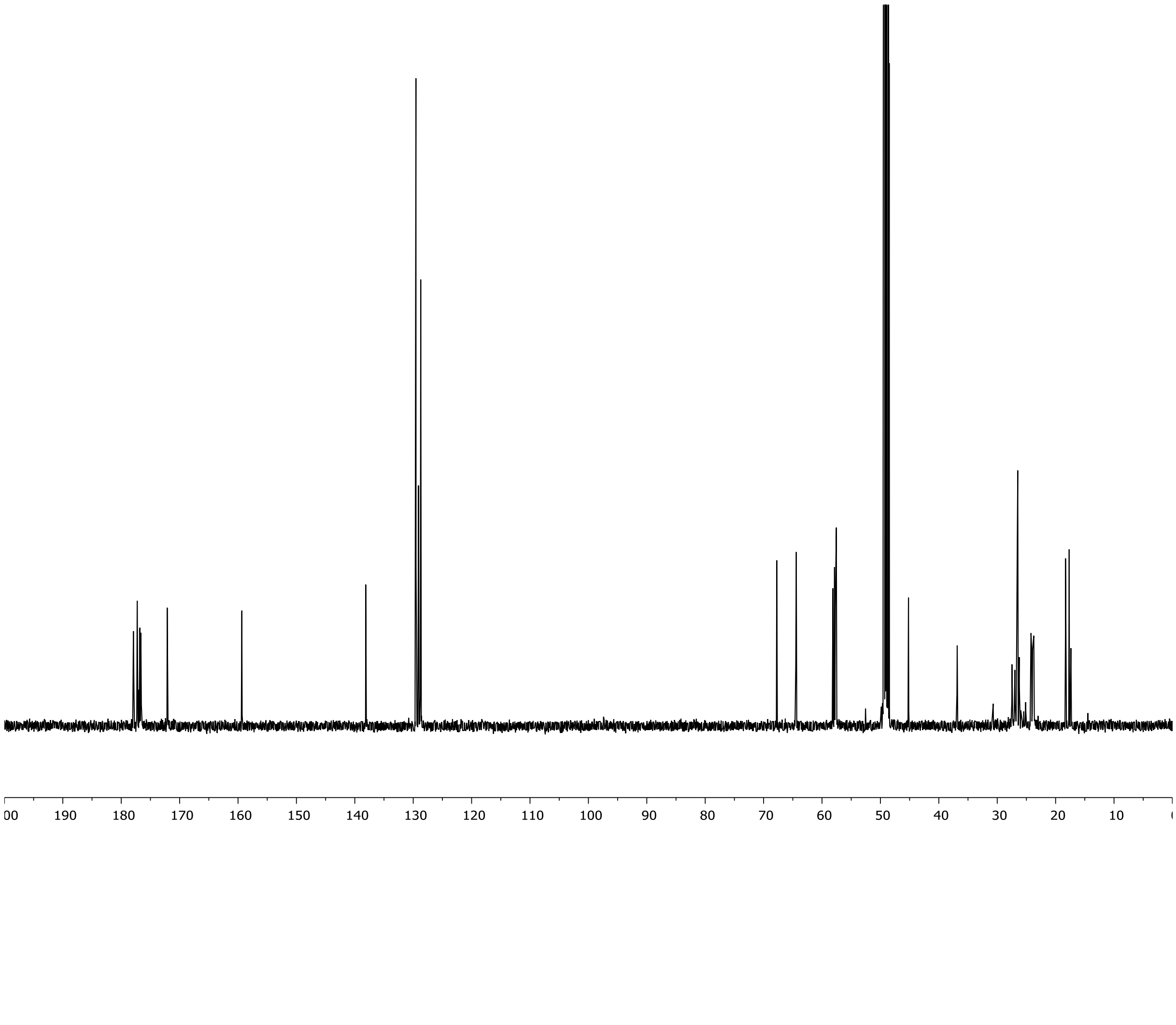
190 180 170 160 150 140 130 120 110 100 90 80 70 60 50 40 30 20 10

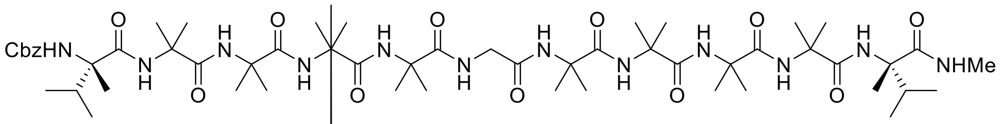

5a

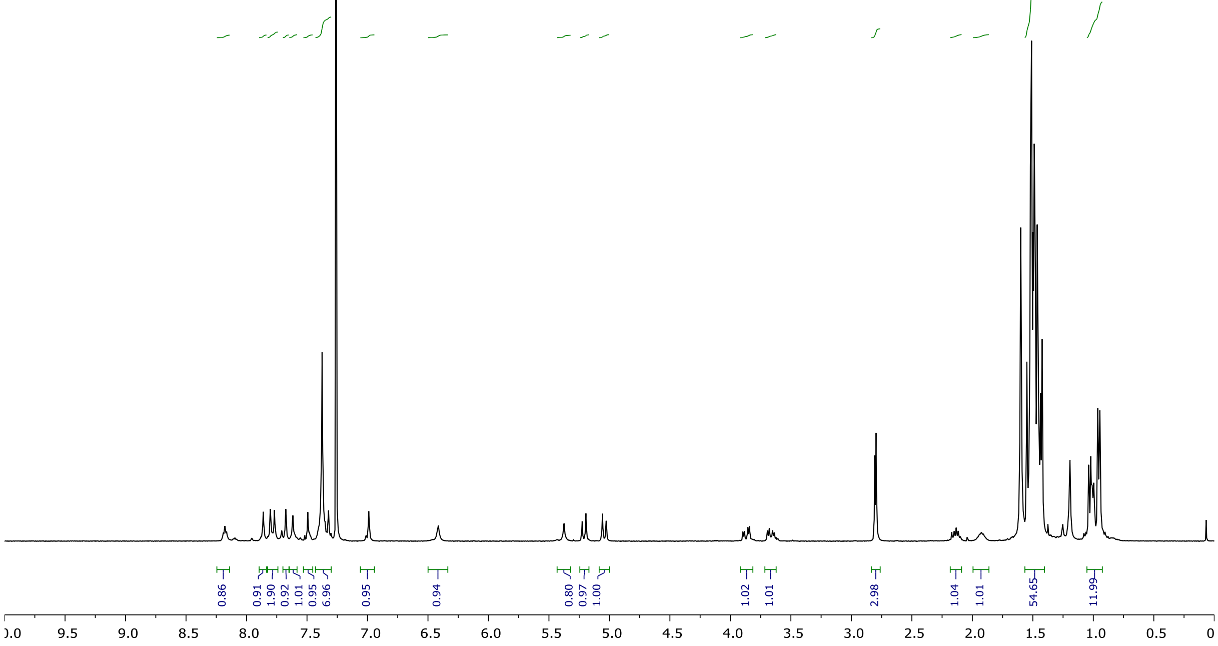

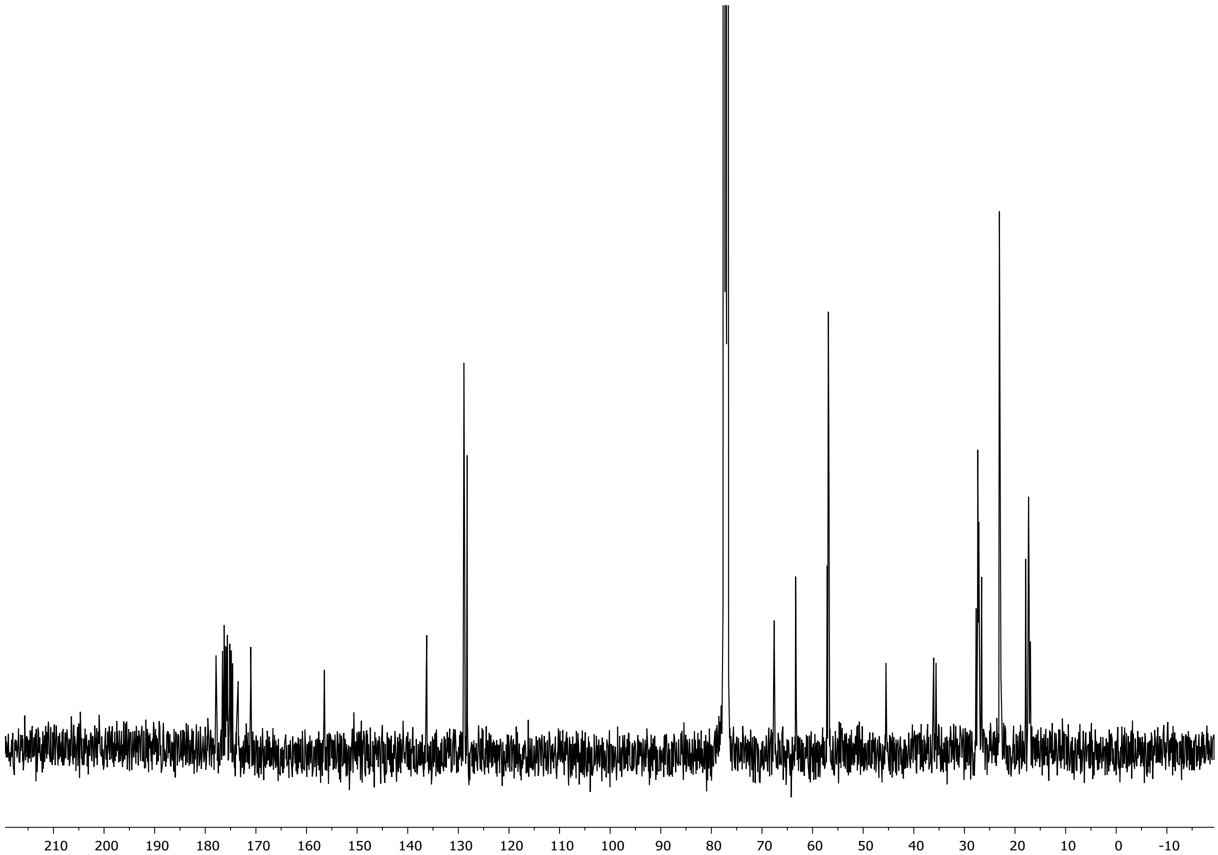

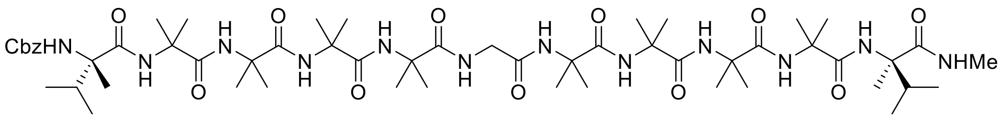
CbzHN
NHMe

5b

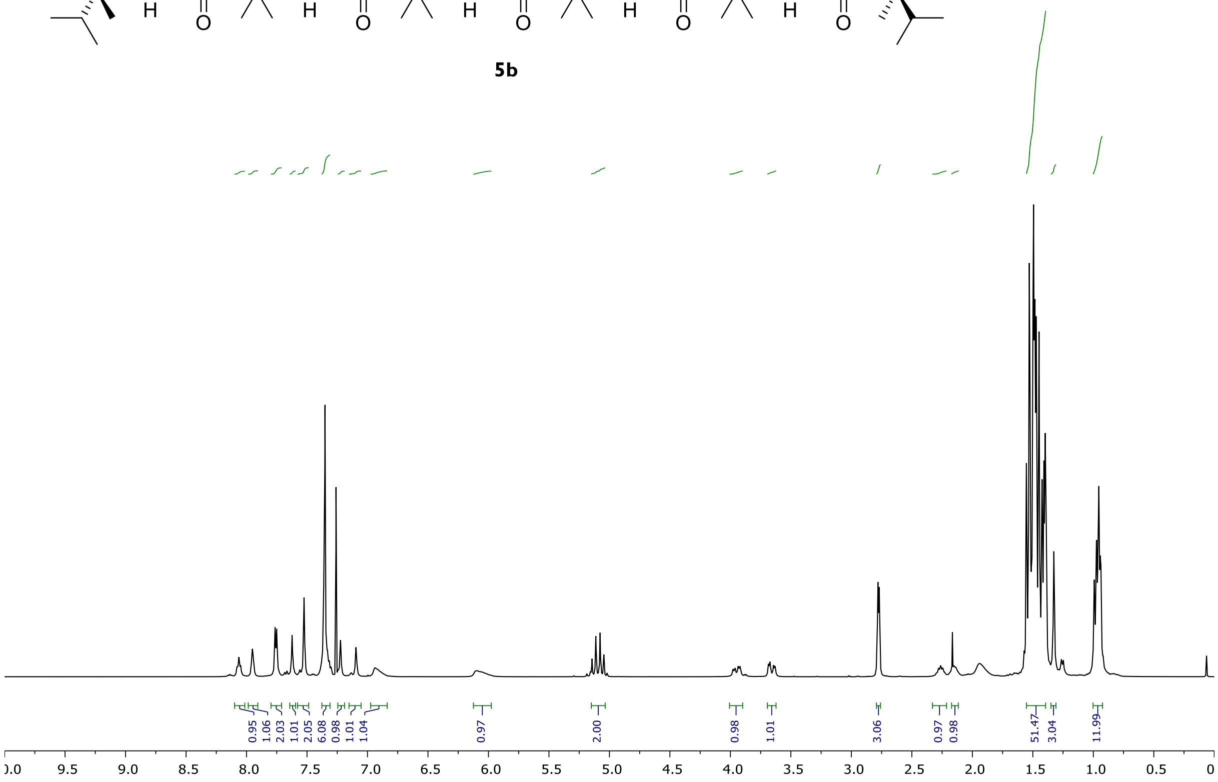
0.95
1.06
2.03
1.01
2.05
6.08
0.98
1.01
1.04
0.97
2.00
0.98
1.01
3.06
0.97
0.98
51.47
3.04
11.99

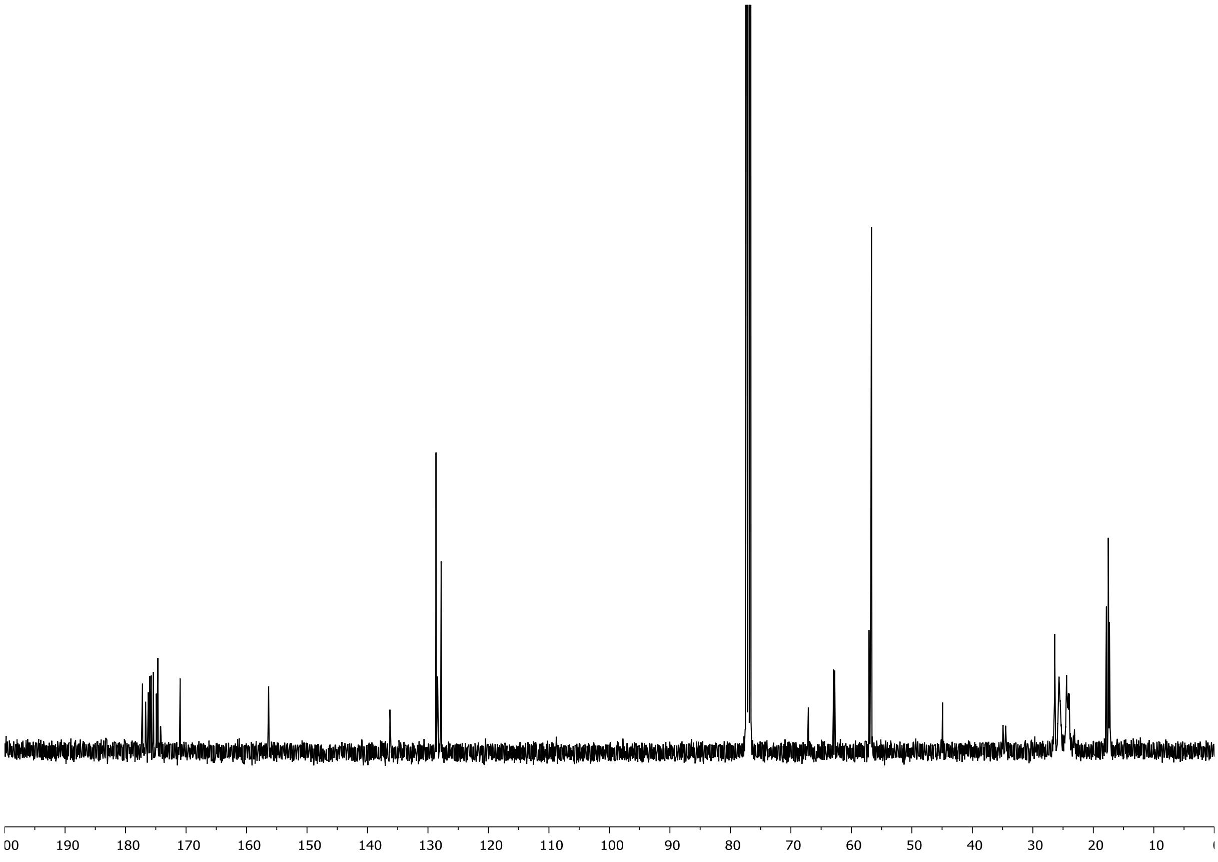

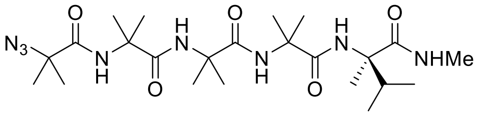

S45

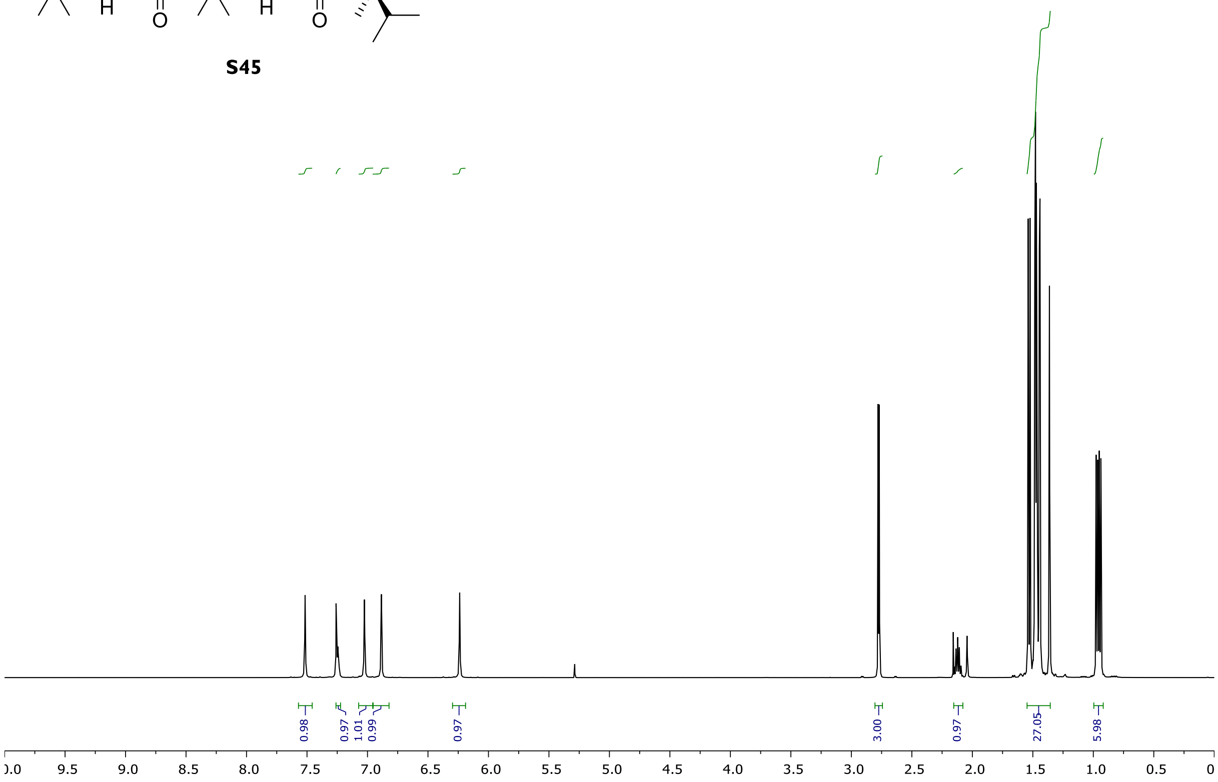

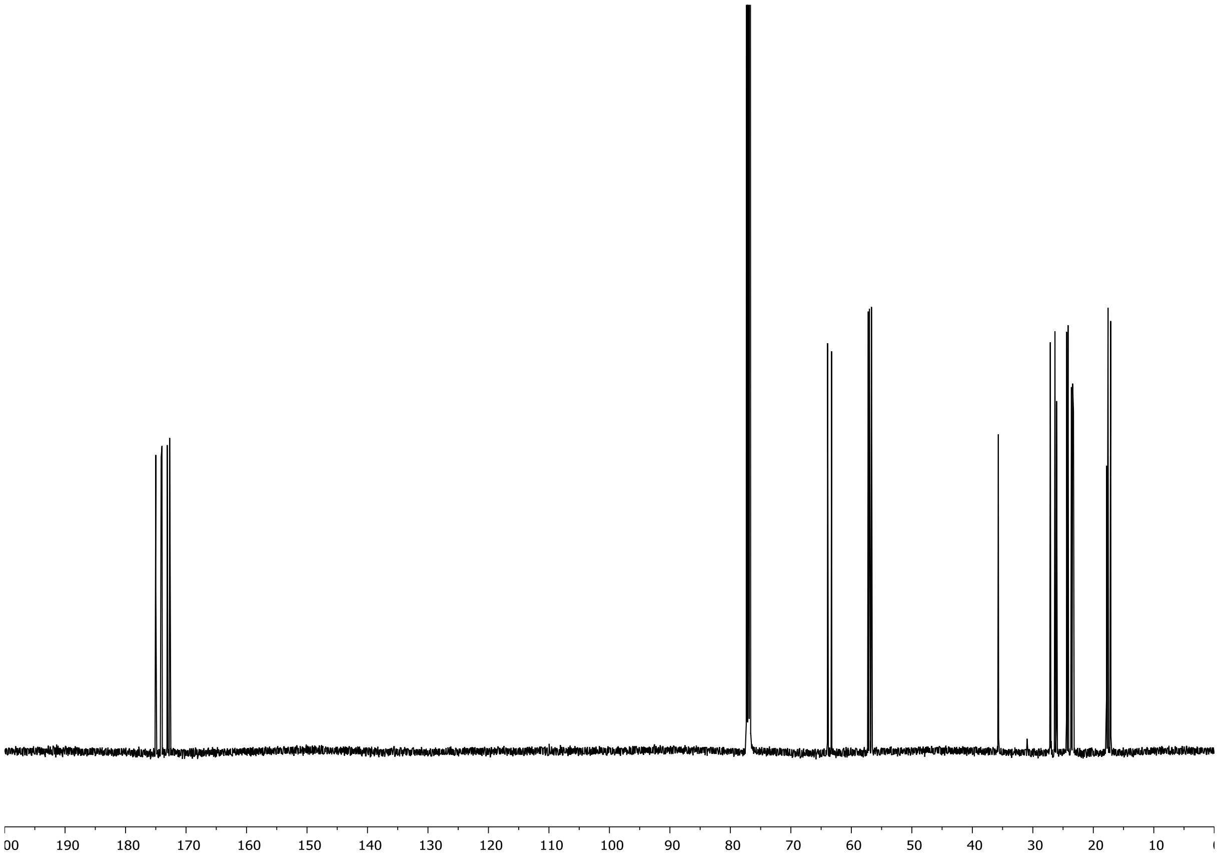

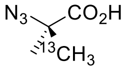

S48

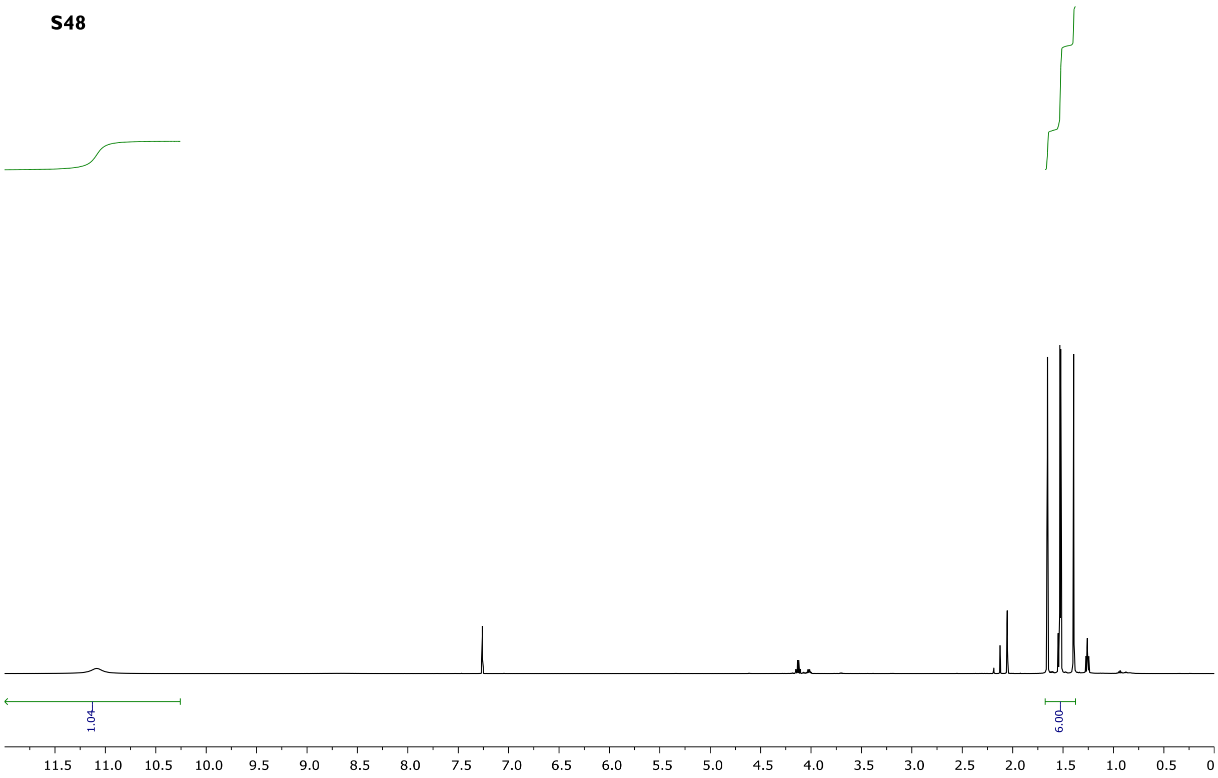

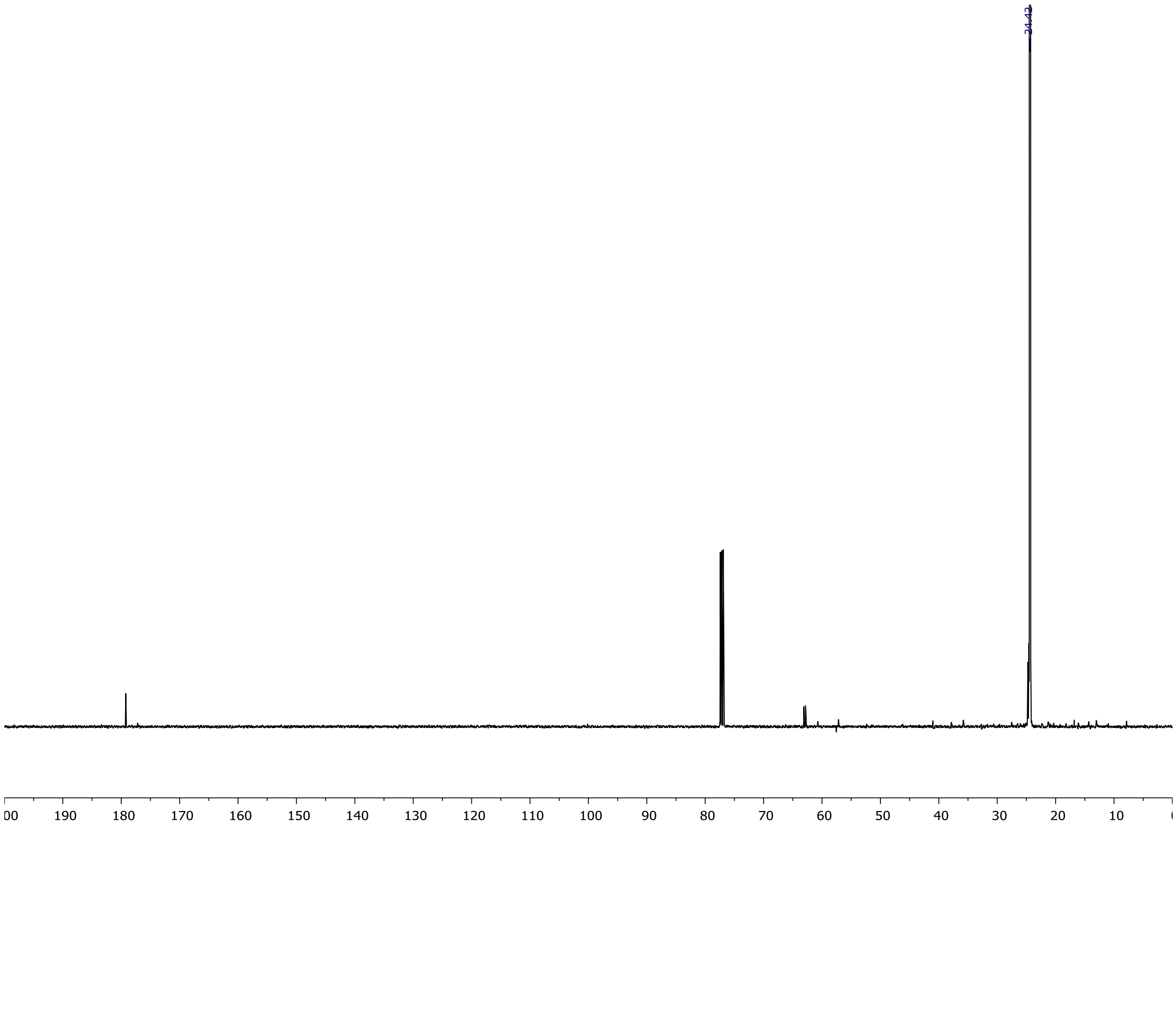

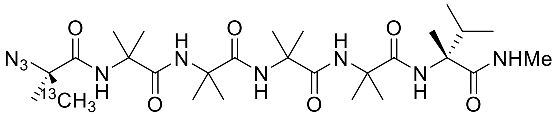

S49

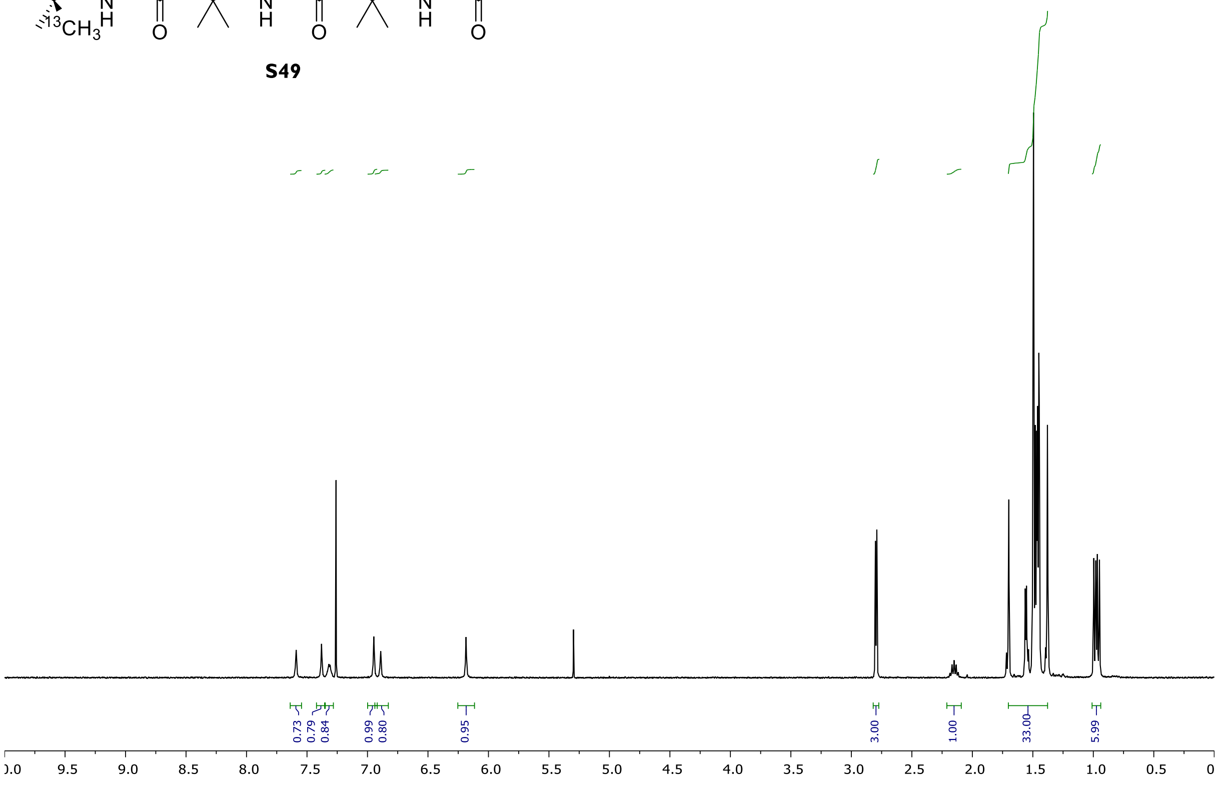

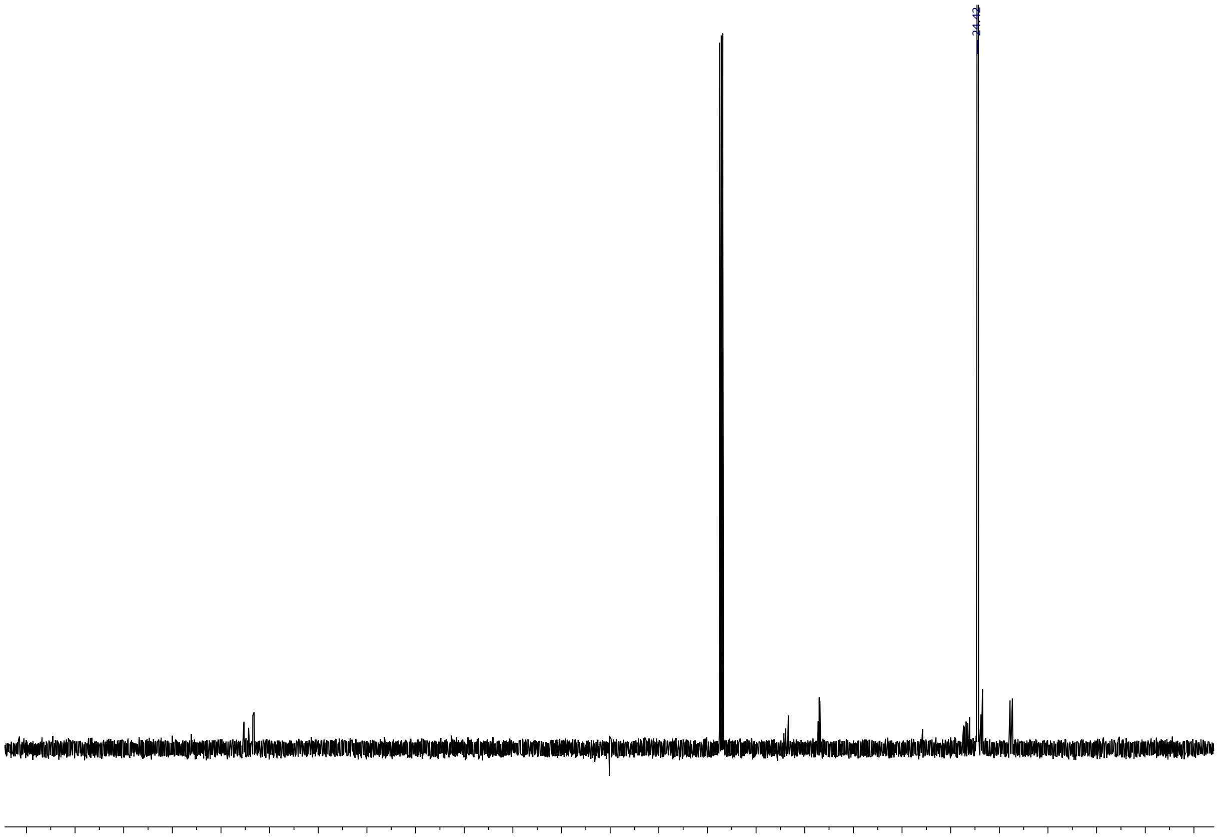

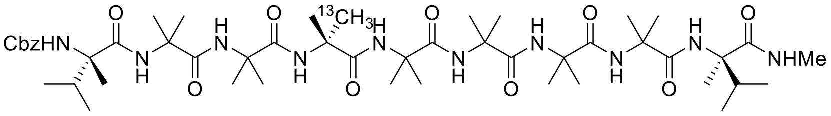

6a

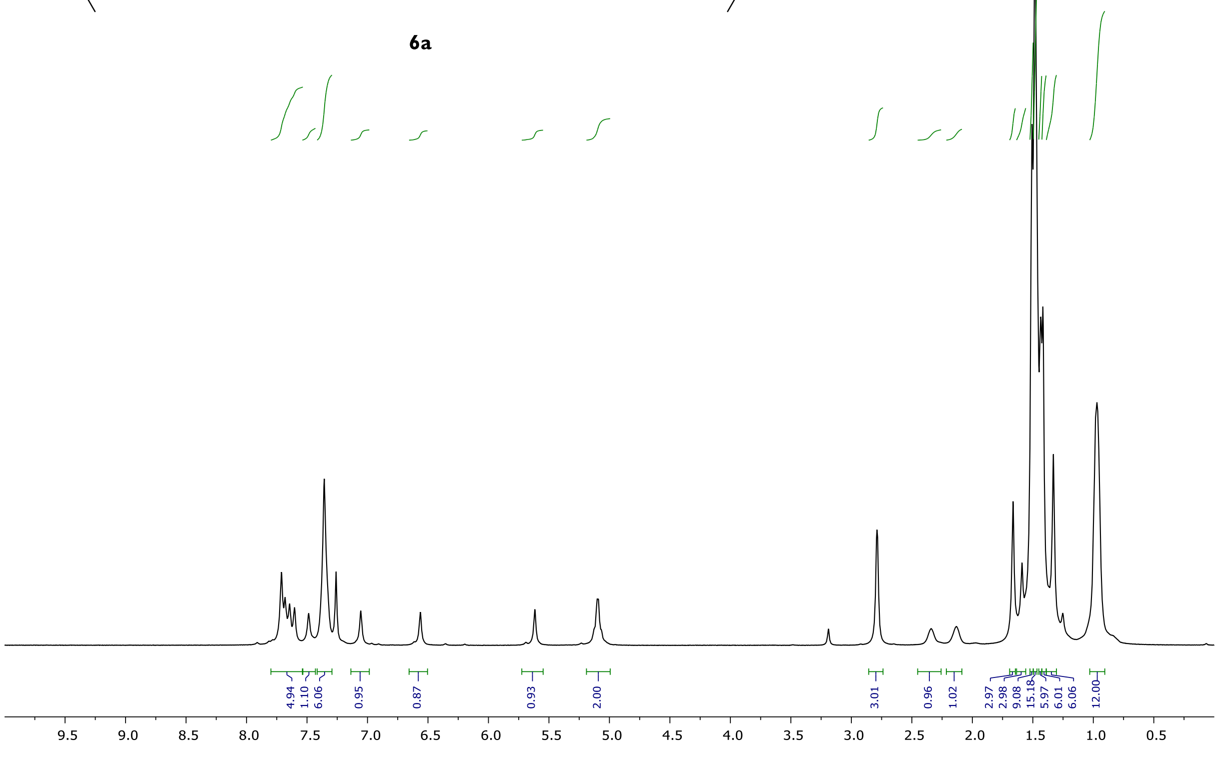

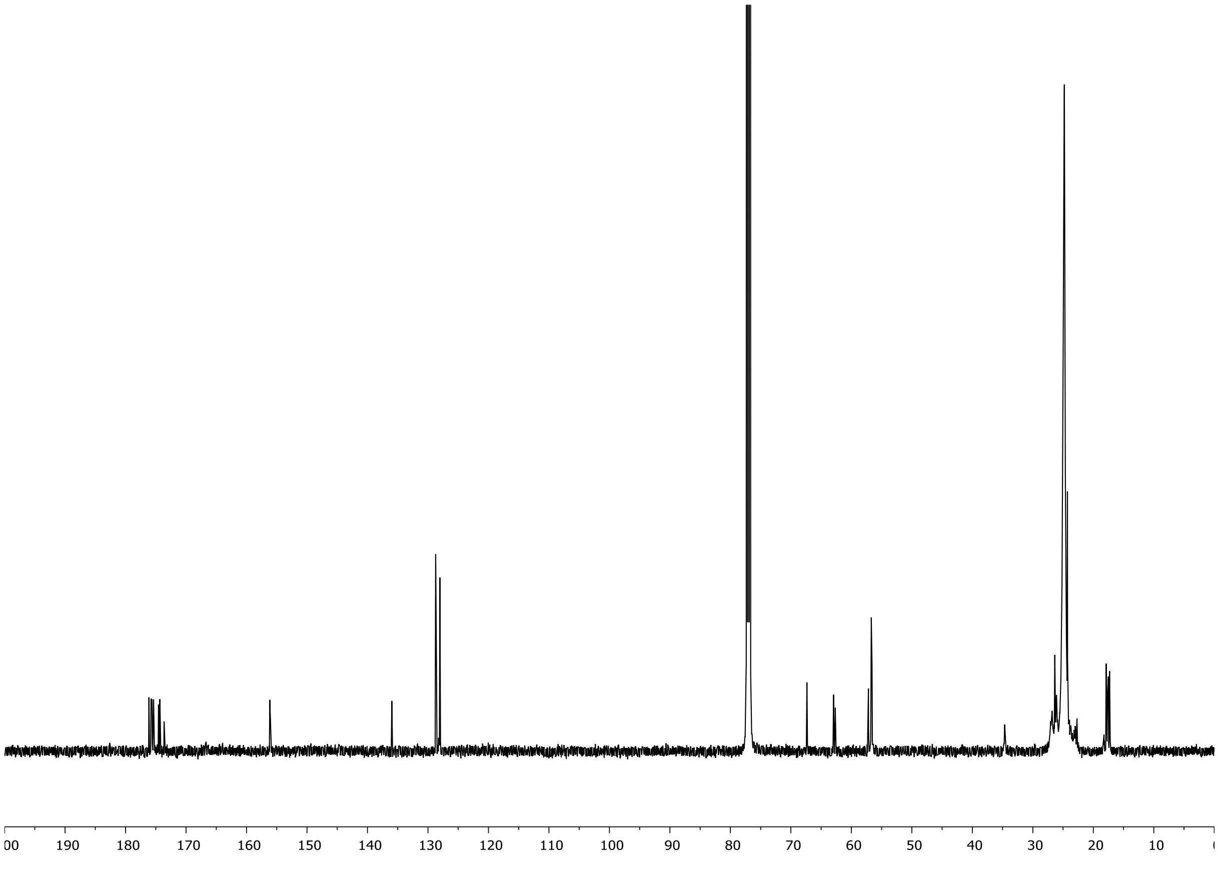

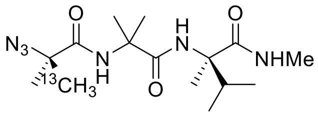

S51

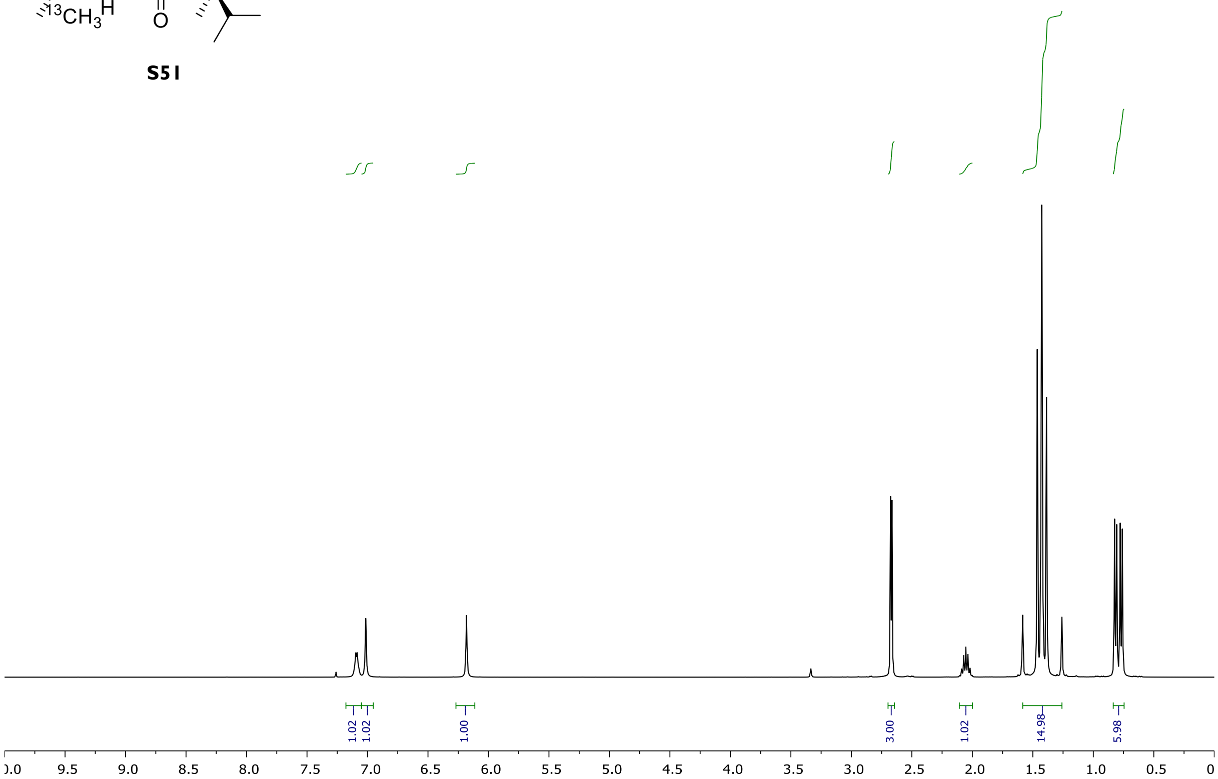

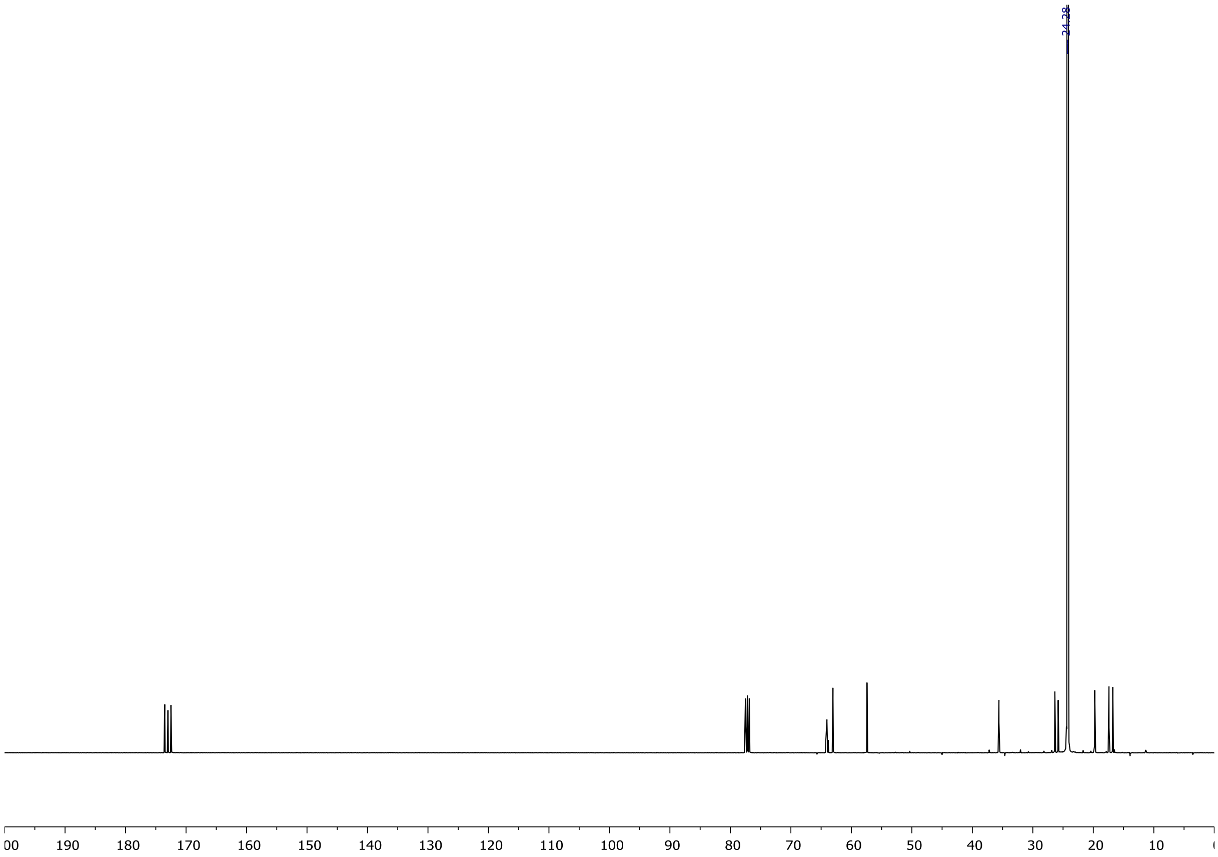

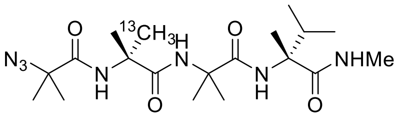
N3
O
13CH3
H
N
NHMe
O

S53

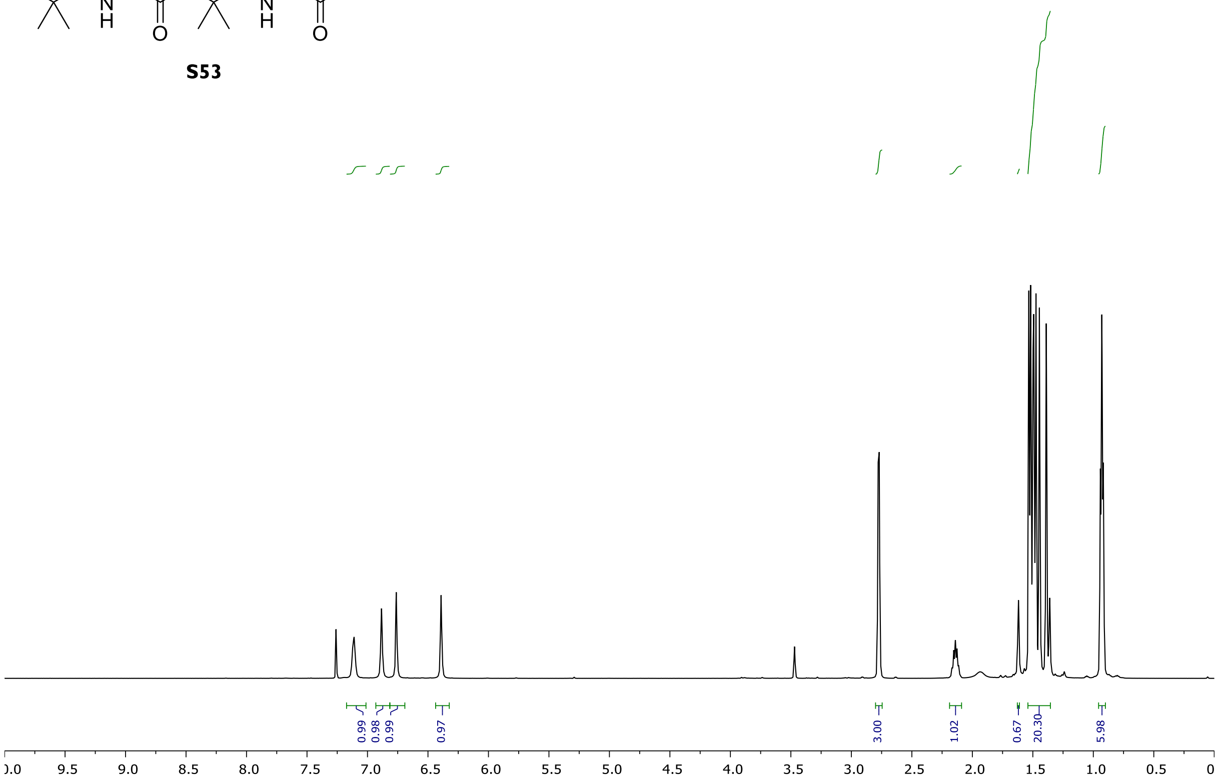
0.99
0.98
0.99
0.97
3.00
1.02
0.67
20.30
5.98
0.0 9.5 9.0 8.5 8.0 7.5 7.0 6.5 6.0 5.5 5.0 4.5 4.0 3.5 3.0 2.5 2.0 1.5 1.0 0.5 0

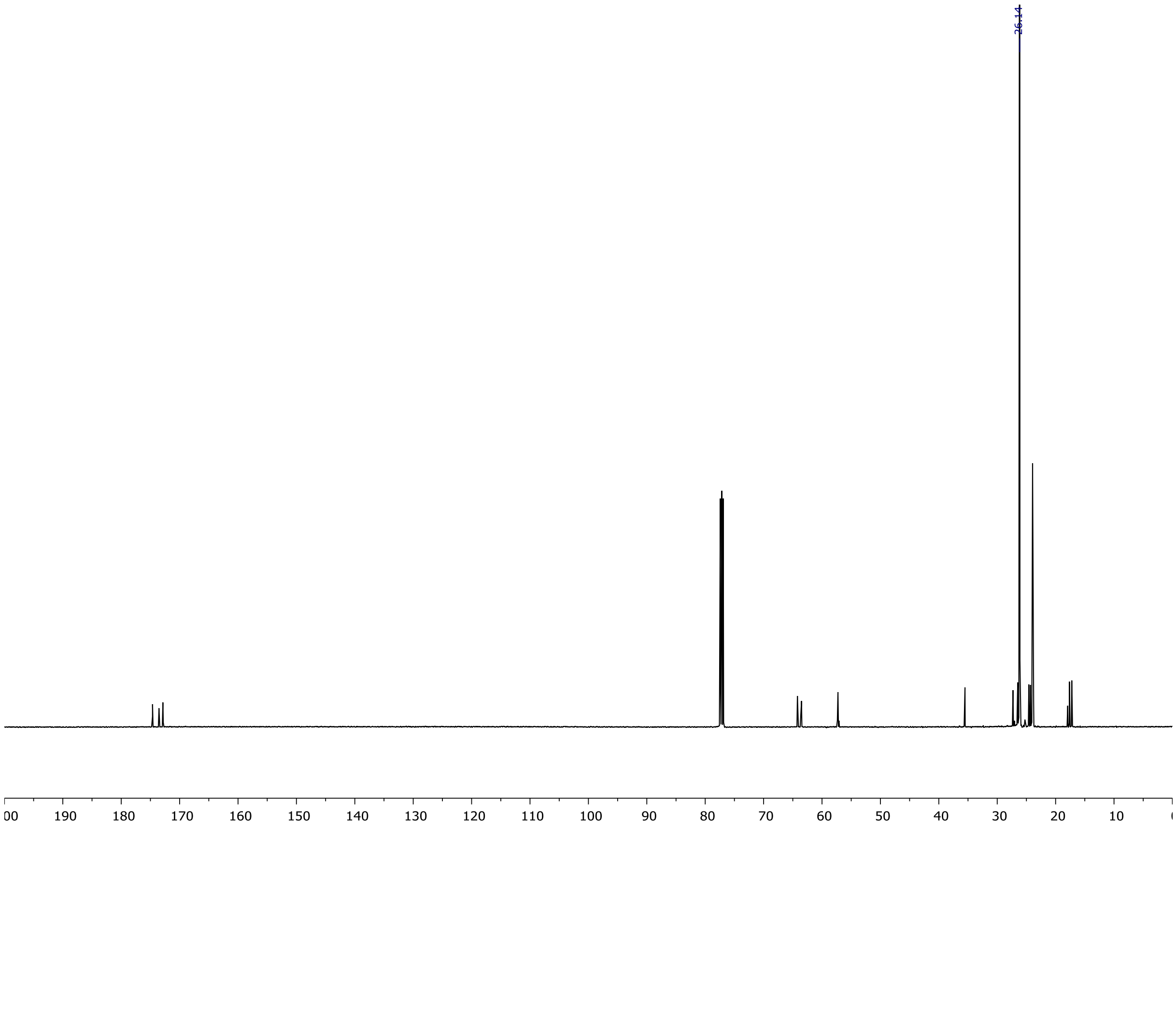
26.14
00 190 180 170 160 150 140 130 120 110 100 90 80 70 60 50 40 30 20 10

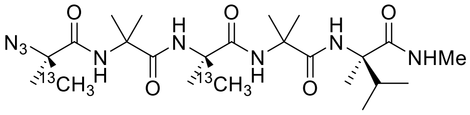

S55

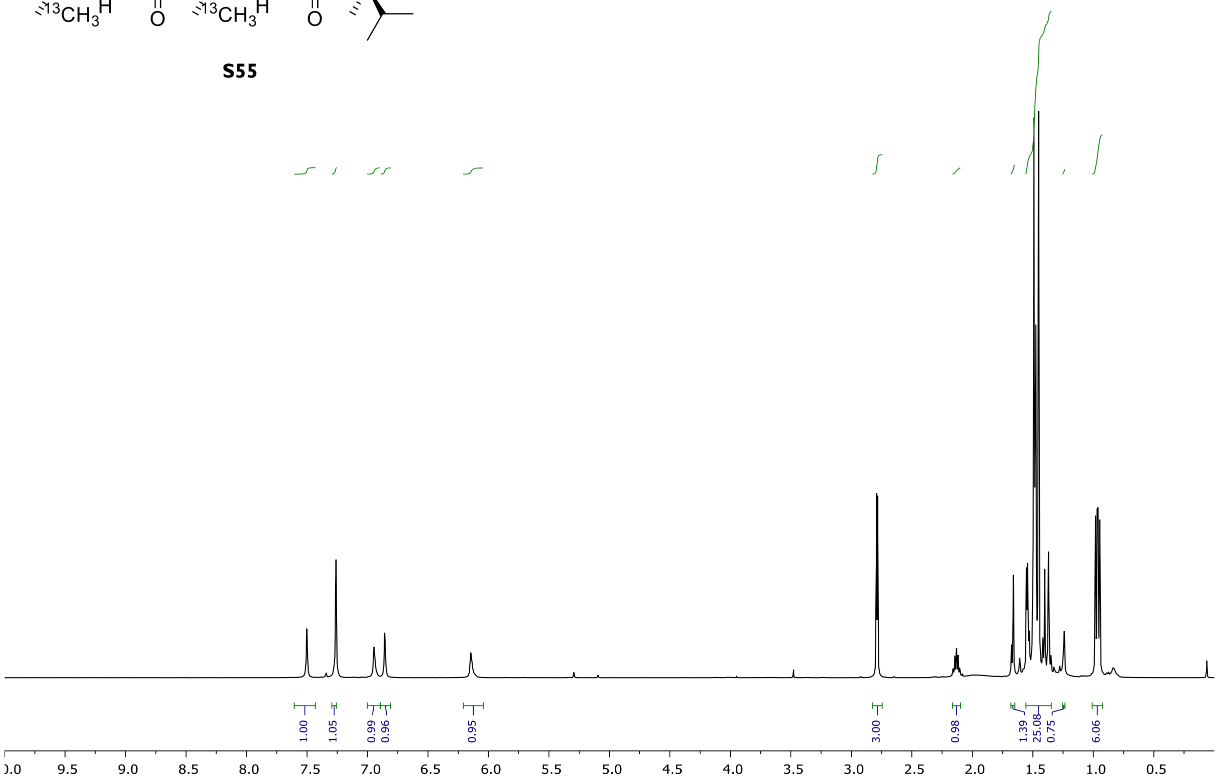

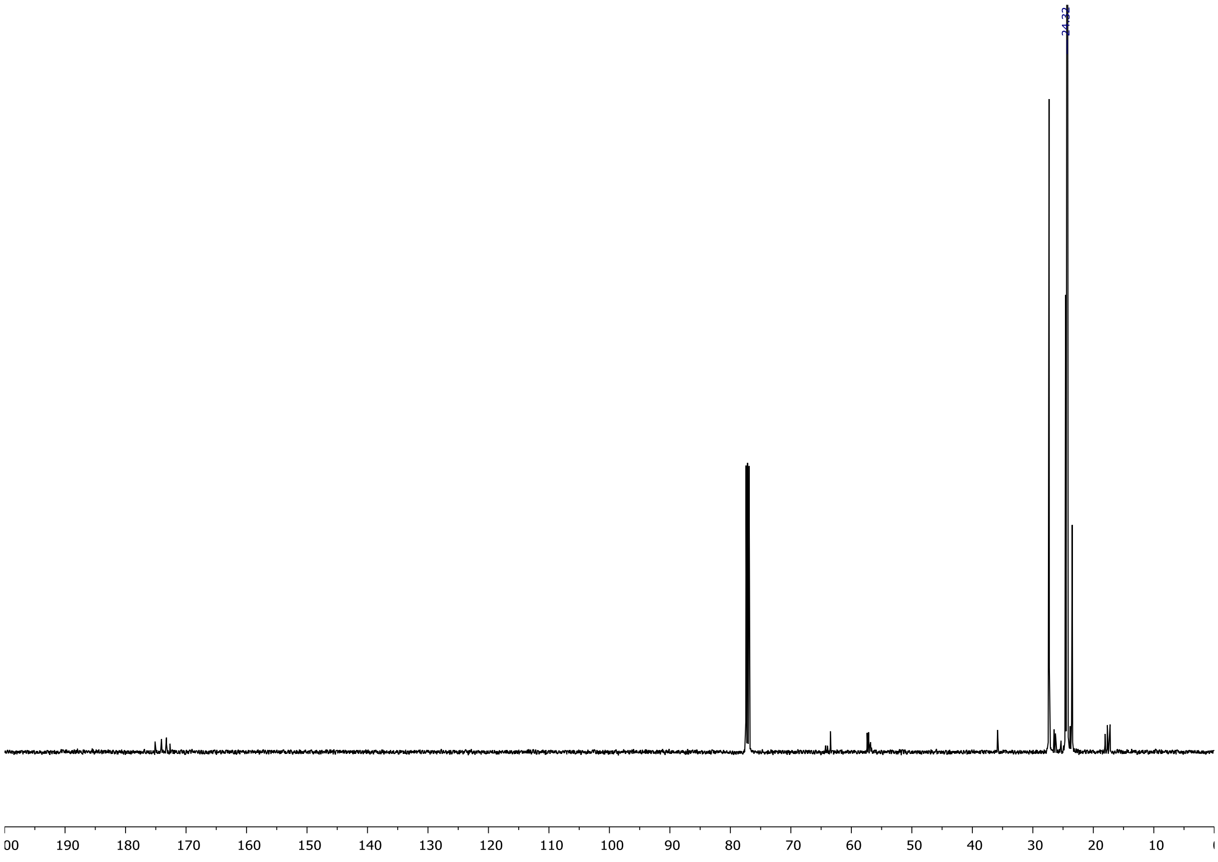

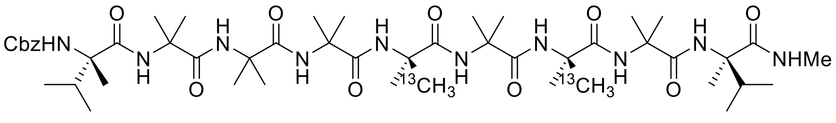
CbzHN
NHMe
O
H
N
$^{13}CH_3$

6b

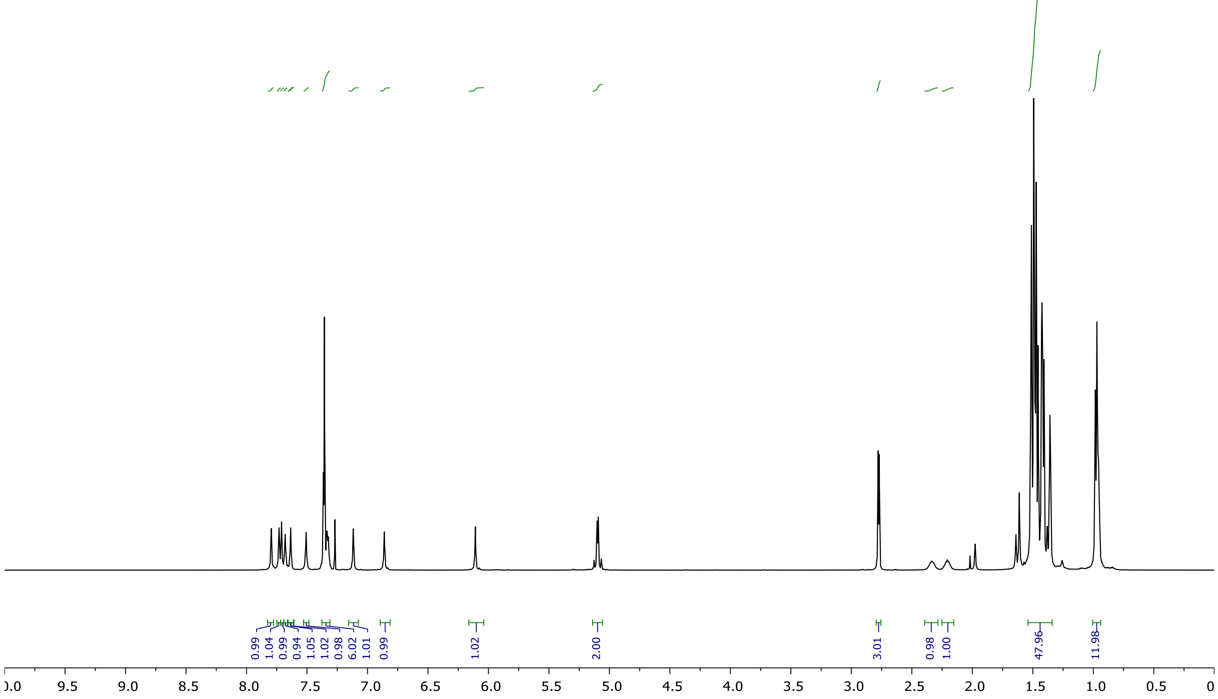
0.99
1.04
0.99
0.94
1.05
1.02
0.98
6.02
1.01
0.99
1.02
2.00
3.01
0.98
1.00
47.96
11.98
10.0
9.5
9.0
8.5
8.0
7.5
7.0
6.5
6.0
5.5
5.0
4.5
4.0
3.5
3.0
2.5
2.0
1.5
1.0
0.5
0

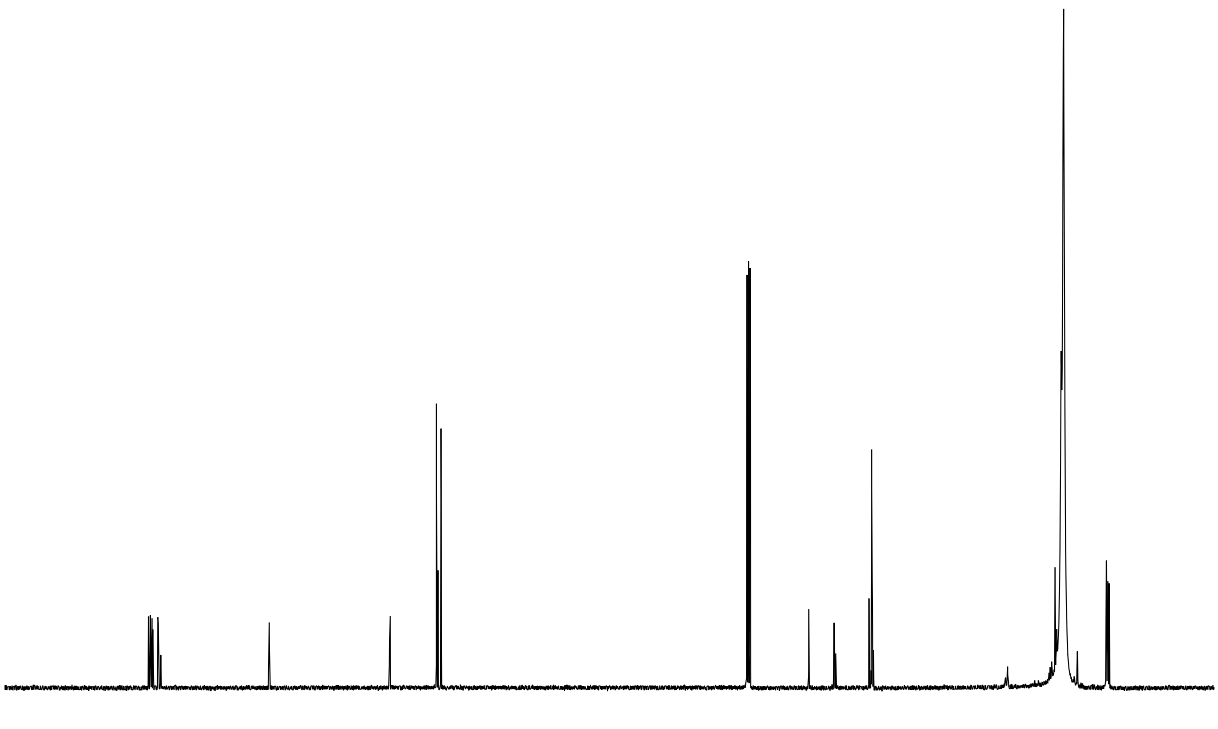
190
180
170
160
150
140
130
120
110
100
90
80
70
60
50
40
30
20
10

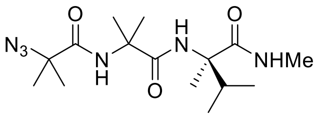
N3
O
N
H
O
H
N
O
NHMe

S57

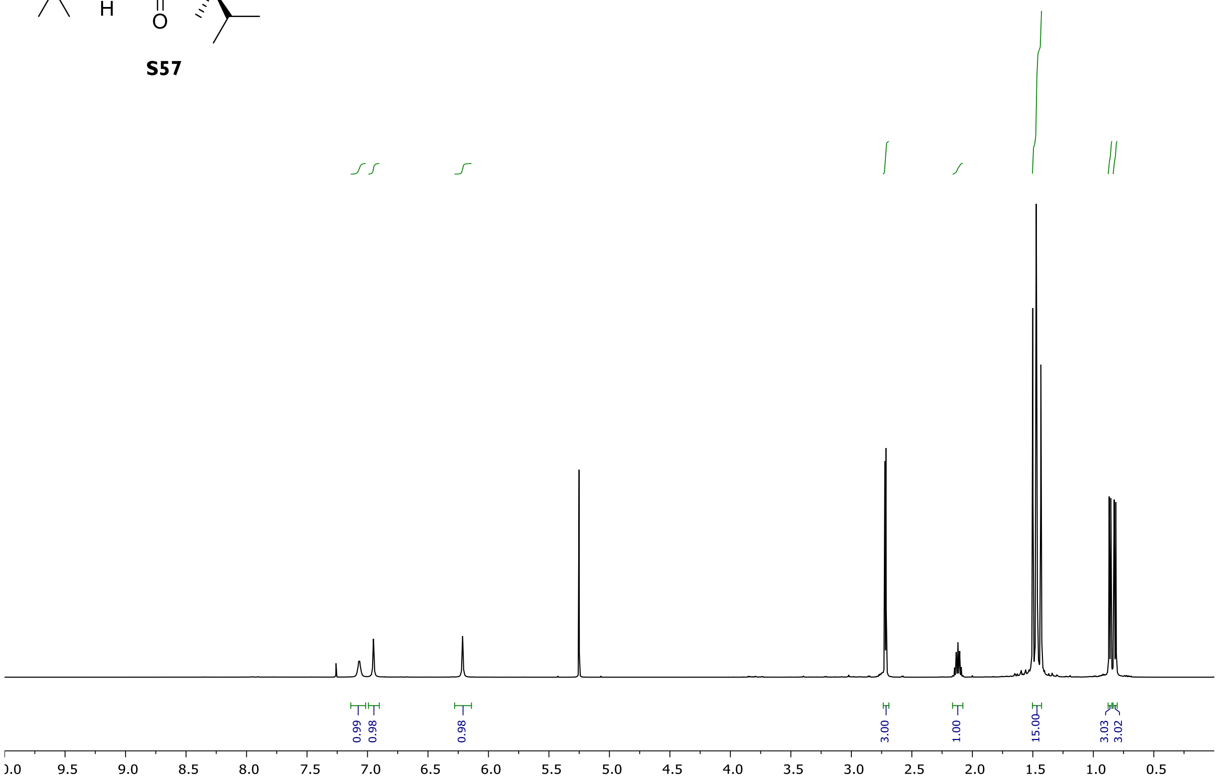
0.99
0.98
0.98
3.00
1.00
15.00
3.03
3.02
9.5 9.0 8.5 8.0 7.5 7.0 6.5 6.0 5.5 5.0 4.5 4.0 3.5 3.0 2.5 2.0 1.5 1.0 0.5

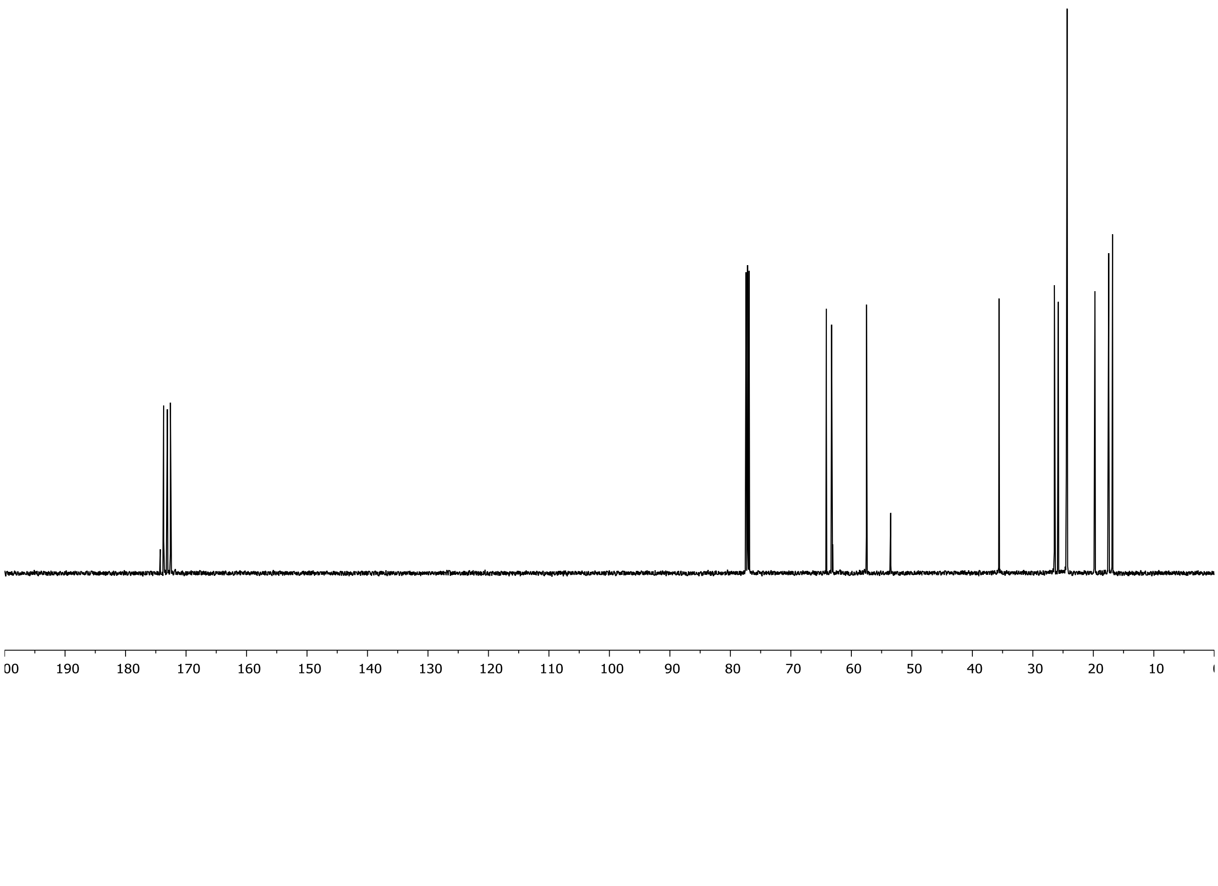
190 180 170 160 150 140 130 120 110 100 90 80 70 60 50 40 30 20 10

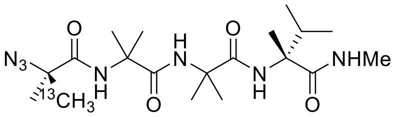
N3
13CH3
NHMe

S59

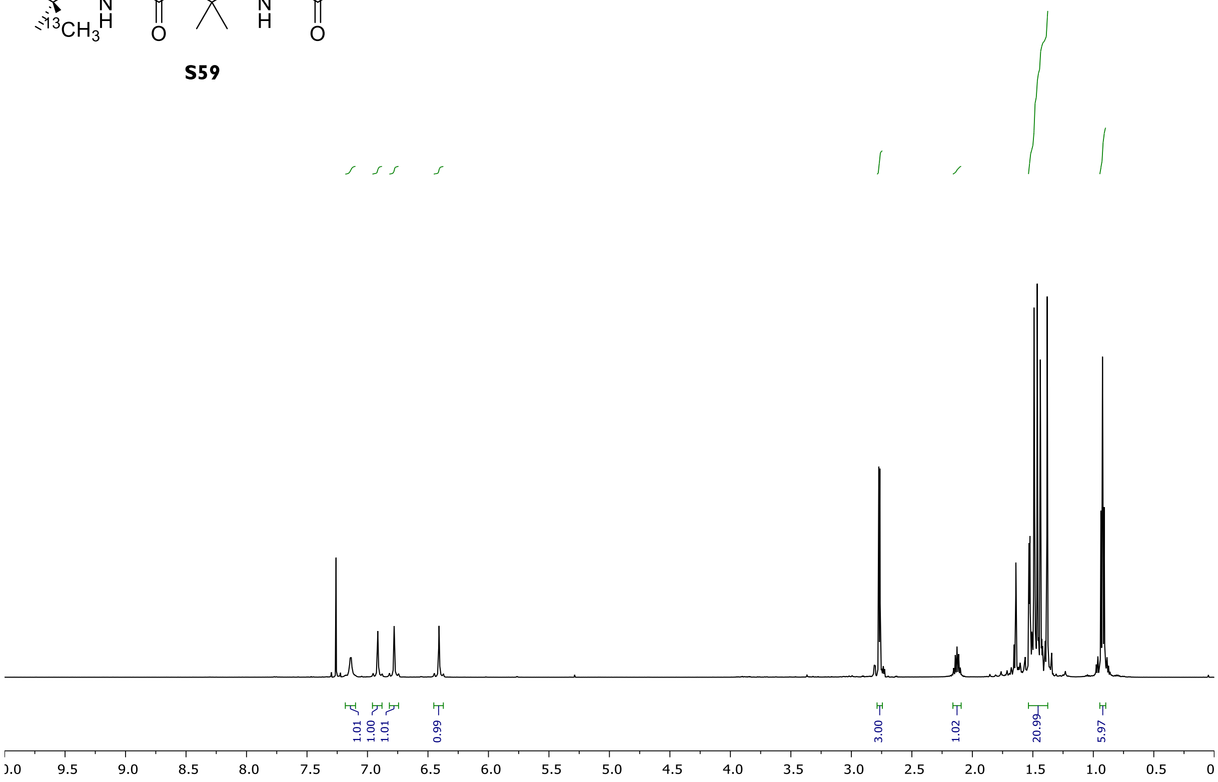
1.01
1.00
1.01
0.99
3.00
1.02
20.99
5.97
9.5 9.0 8.5 8.0 7.5 7.0 6.5 6.0 5.5 5.0 4.5 4.0 3.5 3.0 2.5 2.0 1.5 1.0 0.5 0

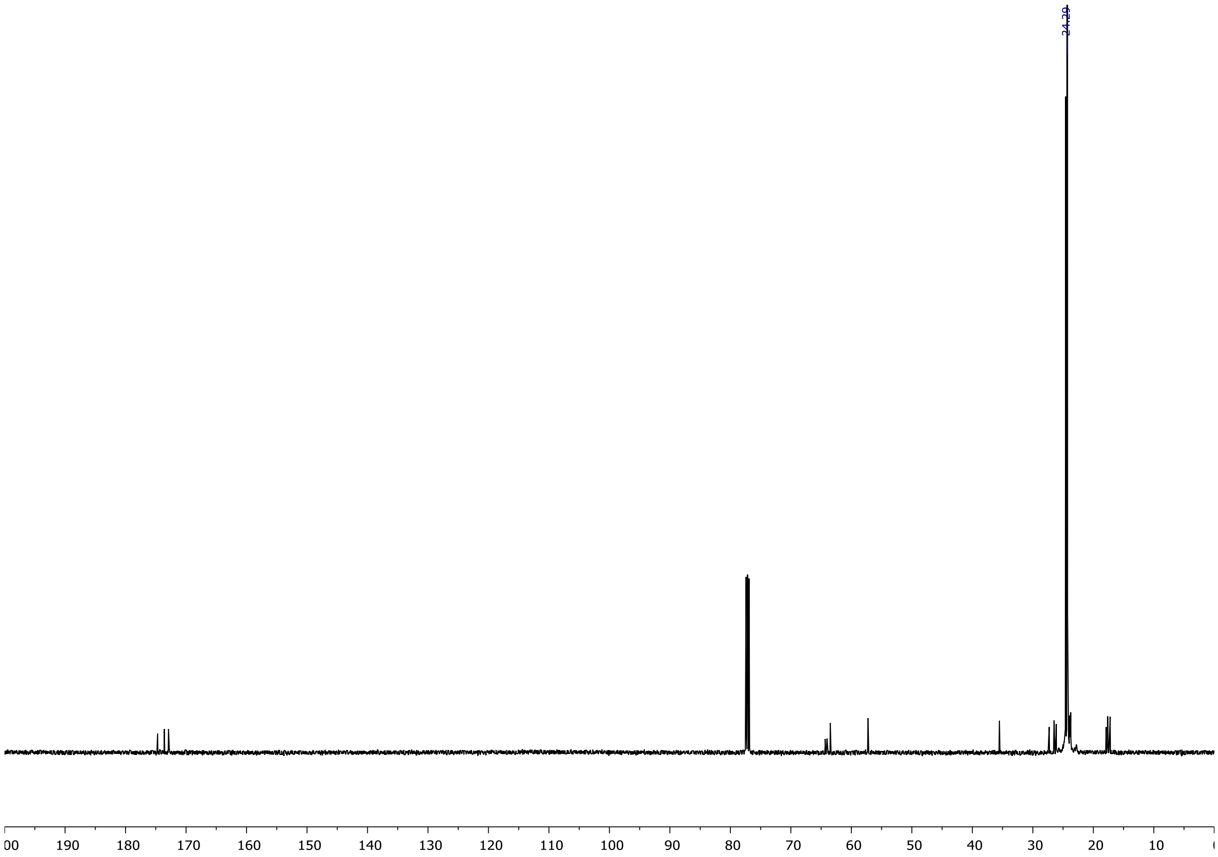
24.29
190 180 170 160 150 140 130 120 110 100 90 80 70 60 50 40 30 20 10

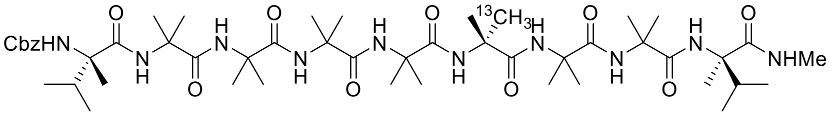

6c

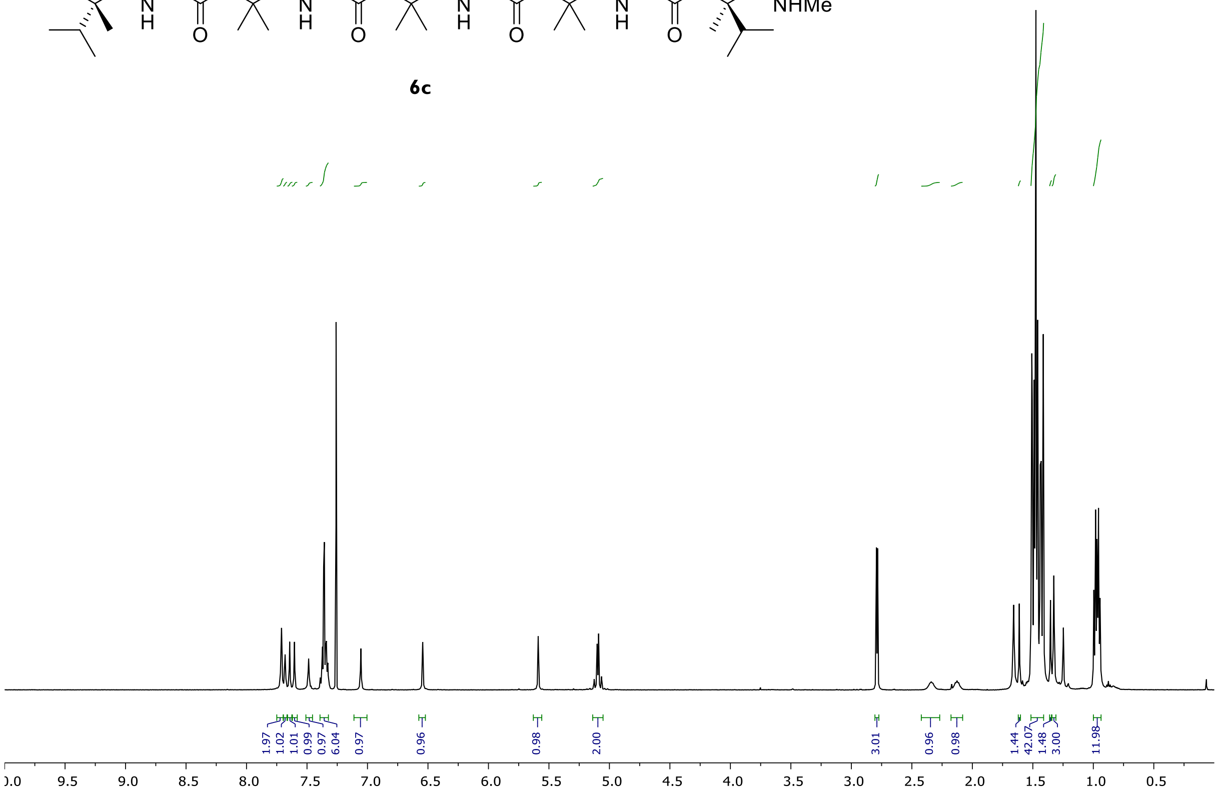

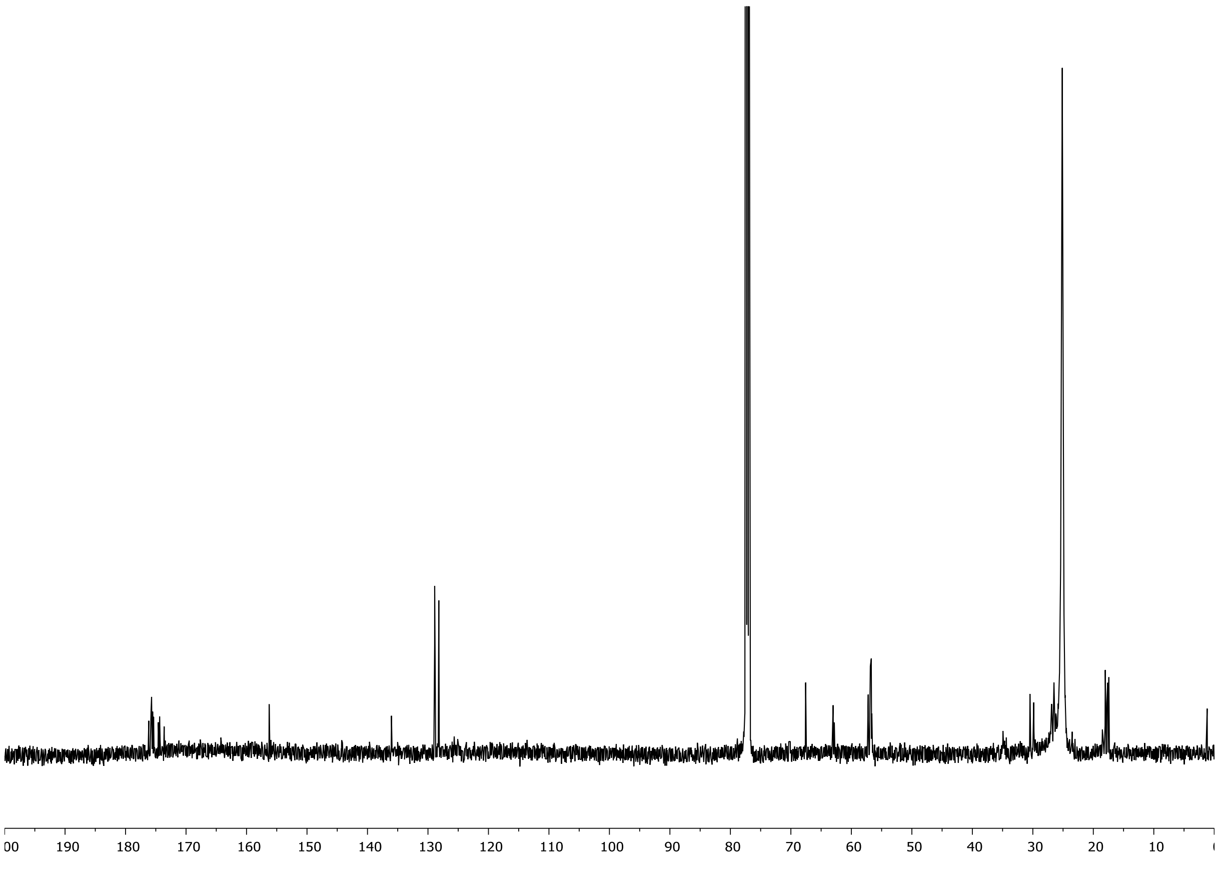

3. Computational methods

REMD Simulations. Aib, (R)- and (S)-αMeVal amino acids and the Cbz protecting group were designed using MOE[11], the formers capped with an acetyl (Ac) and a NHMe group at the N- and C-termini, respectively, while the latter capped with the only NHMe group. They were then submitted to a "Low Mode" conformational search by setting MMFF94x as force field, Born solvation model, iteration limit = 40000, MM iteration limit = 2500, and rejection limit = 500. For each molecule, the two conformations showing the lowest energy and, in the case of the three amino acids, with the ϕ and ψ dihedrals corresponding to the right- and left-handed helical ones (ϕ = ±60° and ψ = ±45°) were chosen for partial charges derivation performed by the R.E.D.IV software[12]. For this step, the selected geometries were optimized at the HF/6-31G(d) theory level and the RESP-A1 charges were derived using two different spatial orientations, in order to have an orientation- and conformation-independent charge derivation. Moreover, the amino acids backbone nitrogen, hydrogen, carbonyl carbon and oxygen charges were fixed at the values reported in the AMBER *ff99SBildn-φ* force field[13] for standard amino acids (e.g. -0.4157, 0.2719, 0.5973 and -0.5679, respectively).

Cbz-(S)-αMeVal_2-Aib_5-(S)-αMeVal_2-NHMe (**1a**) and Cbz-(S)-αMeVal_2-Aib_5-(R)-αMeVal_2-NHMe (**1b**) peptides were built by imposing an extended conformation (ϕ = ψ = ω = 180°). REMD simulations in implicit solvent of the two peptides were performed using the AMBER *ff99SBildn-φ* force field coupled with the implicit solvent model GB-Neck2 (igb = 8),[14] combination that proved to give the best results in predicting peptides secondary structures.[15] 16 replica, spanning temperatures between 260.00 K and 690.08 K with a 0.5 probability exchange, were run for 100 ns each, for a total of 1.6 µs of simulation for each peptide, using the *pmemd* module of the Amber14 package.[16] The trajectories at 297.31 K were extracted, unless stated otherwise, and analyzed on the 50-100 ns time interval.

For the REMD simulations in explicit methanol, the Cbz-(S)-αMeVal_2-Aib_5-(R)-αMeVal_2-NHMe peptide in the extended conformation was solvated with an octahedral box of 1290 MeOH molecules (closeness = 8.0 Å) and preliminary submitted to minimization and equilibrations cycles. Initially 5000 cycles of hydrogens minimization (1000 cycles of steepest descent and 4000 cycles of conjugated gradient), followed by 5000 cycles of solvent minimization (2000 cycles of steepest descent and 3000 cycles of conjugated gradient) were carried out. Then, the solvent box was equilibrated at 300 K by 1ns of NVT equilibration and 1ns of NPT equilibration using the Langevin thermostat with a frequency collision on 2.0. This step was followed by 5000 cycles (2500 of steepest descent and 2500 of conjugated gradient) of solvent and sidechains minimization and by 5000 cycles (2500 of steepest descent and 2500 of conjugated gradient) of total minimization. The last step consisted in 100 ps of NVT and 100 ps of NPT equilibration of the whole system. The REMD simulation of the equilibrated system was carried out with the AMBER *ff99SBildn-φ*

force field and by performing 40 replica of 120 ns each (4.8 μs totally) between 290.00 K and 511.61 with an exchange probability of 0.20. The trajectory at 303.60 K was extracted, the solvent was stripped out and the simulation convergence was checked every 10 ns by assuring that the conformations obtained during the 10 ns time intervals were similar on the base of the Root Mean Square Deviation (RMSD).

Cluster analyses were performed with Amber14[16] *cpptraj* using the average-linkage algorithm and the pairwise mass-weighted RMSD on the Cα of residues 7-11, in order to clearly identify where the inversion of the screw helical sense occurs. For the simulations conducted in implicit solvent the 50-100 ns time interval was analyzed by sampling one every four frames and by requesting 5 clusters on the basis of pseudo-F statistics and SSR/SST ratio.[17] As regards the REMD in explicit solvent, since convergence was reached after 50 ns, the last 60 ns were submitted to cluster analysis, one every four frames was sampled and 15 clusters were requested.

H-bond occupancies during the simulations were computed with VMD 1.9.1[18] over the whole trajectories for the simulations in implicit solvent and on the last 60 ns for that in explicit methanol, with a donor-acceptor distance limit of 4.0 Å and an angle cutoff of 60°. This very low angle acceptance threshold was chosen in order to be able to identify also the presence of γ-turns, since it has been showed that the hydrogen bond in γ-turns is highly bent[19] and the N-H-O angle can reach values of 110-130°. Only H-bonds with an occupancy greater than 5% were considered. The H-bond analysis between peptide **2** and methanol molecules was performed with Amber14 *cpptraj*, using successively the backbone carbonyl oxygen atoms as acceptor atoms and setting methanol molecules as solvent donor, then the methanol residues were considered as solvent acceptor and the backbone amidic hydrogens were considered as donor atoms. In this case the distance cutoff was set to 4.0 Å and the minimum angle accepted was fixed at 150°, as for standard H-bonds.

Potential of Mean Force (PMF) as a function of ϕ and ψ dihedrals were computed with Amber software coupled with the Weighted Histogram Analysis Method (WHAM)[20] over the whole implicit solvent trajectories and over the last 60 ns for the explicit methanol simulation by setting a histogram limit of ±180°, 100 bins and a tolerance of 0.01. Temperatures between 260.00 K and 317.73 K were considered. A threshold of 6 kcal/mol has been fixed for the non-accessible conformations.

Table S1. H-bond analyses of REMD trajectories of peptides **1a** and **1b** (Donor, N-H; Acceptor, C=O).

peptide **1a**			peptide **1b**		
donor	acceptor	occupancy	Donor	acceptor	occupancy
Aib4	(S)-αMeVal1	90.24%	Aib4	(S)-αMeVal1	88.43%
Aib5	(S)-αMeVal2	93.05%	Aib5	(S)-αMeVal2	86.74%
Aib6	Aib3	92.92%	Aib6	Aib3	86.11%
Aib7	Aib4	92.74%	Aib7	Aib4	86.95%
Aib7	Aib5	6.52%	Aib7	Aib5	8.42%
(S)-αMeVal8	Aib5	73.44%	(R)-αMeVal8	Aib5	56.07%
(S)-αMeVal8	Aib6	7.46%	(R)-αMeVal8	Aib6	10.50%
(S)-αMeVal9	Aib6	77.69%	(R)-αMeVal9	Aib6	59.00%
(S)-αMeVal9	Aib7	6.91%	(R)-αMeVal9	Aib7	8.40%

Table S2. H-bond analysis of explicit solvent REMD trajectory of peptide **1b**.

donor	acceptor	occ%
Aib4	(S)-αMeVal1	82.83
Aib3	(S)-αMeVal1	7.38
Aib5	(S)-αMeVal2	81.43
Aib4	(S)-αMeVal2	5.9
Aib6	Aib3	79.53
Aib5	Aib3	6.06
Aib7	Aib4	81.81
Aib6	Aib4	5.55
(R)-αMeVal8	Aib5	73.43
Aib7	Aib5	8.93
(R)-αMeVal9	Aib6	75.51
(R)-αMeVal8	Aib6	14.83
(R)-αMeVal9	Aib7	11.41

Donor	Acceptor	Frac%§	Donor	Acceptor	Frac%§
(S)-αMeVal1	MeOH	70.4	MeOH	(R)-αMeVal9	71.55
(S)-αMeVal2	MeOH	41.5	MeOH	(R)-αMeVal8	61.03
Aib6	MeOH	11.9	MeOH	Aib7	41.68
Aib5	MeOH	11.4	MeOH	Aib3	34.52
Aib7	MeOH	9.85	MeOH	Aib4	31.52
Aib4	MeOH	7.09	MeOH	Aib5	29.84
(R)-αMeVal8	MeOH	3.79	MeOH	(S)-αMeVal2	26.68
Aib3	MeOH	3.6	MeOH	(S)-αMeVal1	24.73
(R)-αMeVal9	MeOH	0.83	MeOH	Aib6	22.45

§ The frac% doesn't represent a real occupancy, since for any given frame more than one solvent molecule can bind to the same place.

Table S3. Variation helical excess with position in the chain for the achiral Aib5 domain of **1a** calculated from the Boltzmann distributions resulting from PMF profiles in implicit solvent.

Aib res	Erel (kcal/mol)		B-factor[a]		h.e.%
	M	P	M	P	
1	1.66	0.00	0.06	1.00	88.38
2	1.32	0.00	0.11	1.00	80.18
3	1.30	0.00	0.11	1.00	80.18
4	1.08	0.00	0.16	1.00	72.41
5	1.51	0.00	0.08	1.00	85.19

[a]Calculated as $exp^{\left(\frac{-E_{rel}}{RT}\right)}$ with R being the gas constant and T the temperature (300K).

Table S4. Variation helical excess with position in the chain for the achiral Aib5 domain of **1b** calculated from the Boltzmann distributions resulting from PMF profiles in implicit solvent.

Aib res	Erel (kcal/mol)[a]		B-factor[b]		h.e.%
	M	P	M	P	
1	1.53	0.00	0.08	1.00	85.19
2	0.98	0.00	0.19	1.00	68.07
3	0.62	0.00	0.35	1.00	48.15
4	0.39	0.00	0.52	1.00	31.58
5	-0.57	0.00	2.60	1.00	-44.44

[a]Calculated as $exp^{\left(\frac{-E_{rel}}{RT}\right)}$ with R being the gas constant and T the temperature (300K).

Table S5. Variation of helical excess with position in the chain for the achiral Aib5 domain of **1b** calculated from the Boltzmann distributions resulting from PMF profiles in explicit methanol.

Aib res	Erel (kcal/mol)[a]		B-factor[b]		h.e.%
	M	P	M	P	
1	0.75	0.00	0.28	1.00	56.25
2	0.32	0.00	0.58	1.00	26.58
3	-0.10	0.00	1.18	1.00	-8.23
4	-0.43	0.00	2.06	1.00	-34.64
5	-0.77	0.00	3.64	1.00	-56.90

Calculated as $exp^{\left(\frac{-E_{rel}}{RT}\right)}$ with R being the gas constant and T the temperature (300K).

4. Raman spectroscopy

Experimental procedure

Raman spectra were recorded on a ChiralRaman spectrometer (BioTools Inc., USA). Solutions of compounds **1a**, **1b**, **5a**, **5b** and $N_3Aib_2O^tBu$,[5] were prepared in $CHCl_3$ (100 μL, 60 mg/mL). All measurements were performed at 25 °C. Spectral deconvolution was performed using Origin Pro9.

Raman spectra of 1a and 1b

Foldamers **1a** and **1b** both present one peak, centred at about 1664 cm^{-1}, in the amide I region after deconvolution. The position of this peak is consistent with foldamer in a 3_{10} helical conformation.

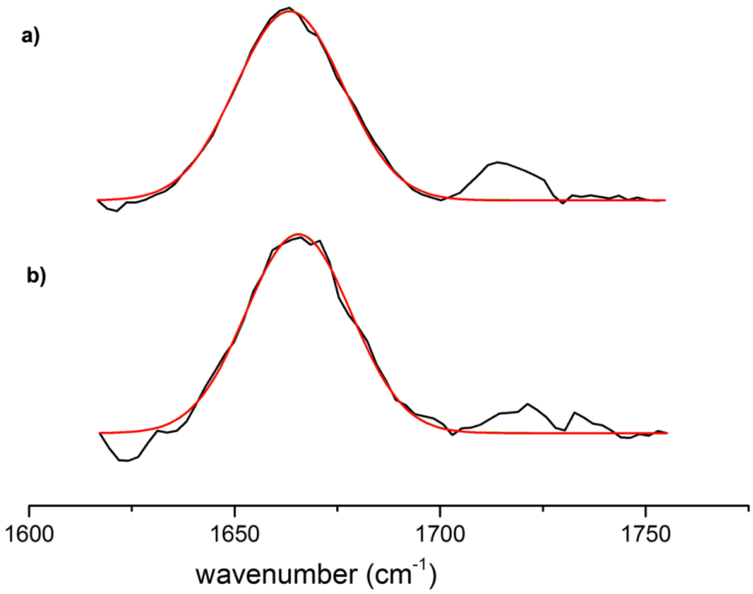

Figure S1: Amide I region of foldamers a) **1a** and b) **1b** in $CHCl_3$. The black trace shows the experimental data, the green trace (obscured) shows the peak deconvolution and the red trace shows the calculated cumulative peak.

Raman spectra of 5a, 5b and $N_3Aib_2O^tBu$

The deconvolution of the amide I region for the peptides **5a** and **5b** showed the peptides are principally 3_{10}-helical. Deconvolution shows that 95% of the population is given by the peak at 1661 cm^{-1} and 5% by the peak at 1682 cm^{-1} for **5a** and 91% at 1661 cm^{-1} and 9% at 1682 cm^{-1} for **5b**.

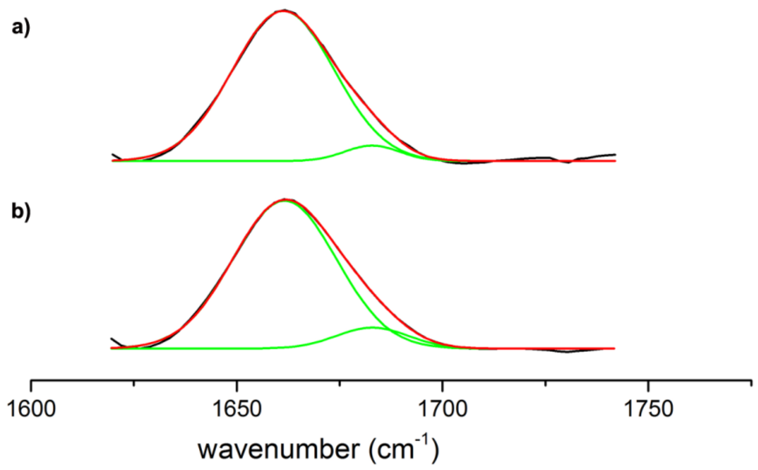

Figure S2: Amide I region of foldamers a) **5a** and b) **5b** in $CHCl_3$. The black trace shows the experimental data, the green trace shows the peak deconvolution and the red trace shows the calculated cumulative peak.

The Raman spectrum of the dimer $N_3Aib_2O^tBu$, which is too short to fold into a helix, shows only a single amide I band at 1681 cm^{-1} after deconvolution, suggesting that the additional peak at 1682 cm-1 in the Raman spectra of 5a and 5b arises from unfolded conformations.

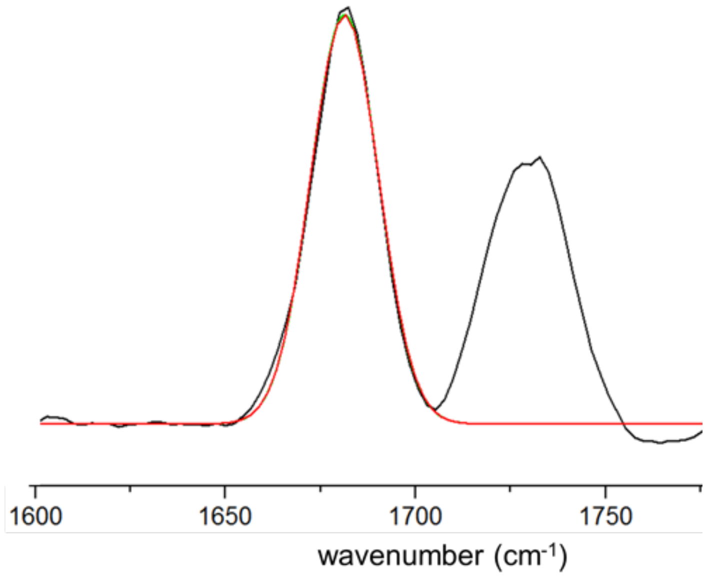

Figure S3: Amide I region of dimer $N_3Aib_2O^tBu$ in $CHCl_3$. The black trace shows the experimental data, the green trace (obscured) shows the peak deconvolution and the red trace shows the calculated cumulative peak.

5. X-ray crystallography

X-ray crystal structure data for **1a** and **1b** have been deposited at the CCDC with the deposition numbers CCDC 1518807 and CCDC 1518806 respectively.

6. References

1 T. S. Cooper, P. Laurent, C. J. Moody and A. K. Takle, *Org. Biomol. Chem.*, 2004, **2**, 265–76.

2 F. Formaggio, M. Bonchio, M. Crisma, C. Peggion, S. Mezzato, A. Polese, A. Barazza, S. Antonello, F. Maran, Q. B. Broxterman, B. Kaptein, J. Kamphuis, R. M. Vitale, M. Saviano, E. Benedetti and C. Toniolo, *Chem. - A Eur. J.*, 2002, **8**, 84–93.

3 M. Meldal*, M. A. Juliano and A. M. Jansson, *Tetrahedron Lett.*, 1997, **38**, 2531–2534.

4 L. Byrne, J. Solà, T. Boddaert, T. Marcelli, R. W. Adams, G. A. Morris and J. Clayden, *Angew. Chemie Int. Ed.*, 2014, **53**, 151–155.

5 J. Clayden, A. Castellanos, J. Solà and G. A. Morris, *Angew. Chemie Int. Ed.*, 2009, **48**, 5962–5965.

6 J.-P. Mazaleyrat, A. Gaucher, J. Šavrda and M. Wakselman, *Tetrahedron: Asymmetry*, 1997, **8**, 619–631.

7 J. Solà, G. A. Morris and J. Clayden, *J. Am. Chem. Soc.*, 2011, **133**, 3712–5.

8 S. J. Pike, J. Raftery, S. J. Webb and J. Clayden, *Org. Biomol. Chem.*, 2014, **12**, 4124–31.

9 S. P. Fletcher, J. Solà, D. Holt, R. A. Brown and J. Clayden, *Beilstein J. Org. Chem.*, 2011, **7**, 1304–9.

10 H. Kim, J. K. Cho, S. Aimoto and Y.-S. Lee, *Org. Lett.*, 2006, **8**, 1149–51.

11 Chemical Computing Group Inc., Montreal, 2013.

12 F.-Y. Dupradeau, A. Pigache, T. Zaffran, C. Savineau, R. Lelong, N. Grivel, D. Lelong, W. Rosanski and P. Cieplak, *Phys. Chem. Chem. Phys.*, 2010, **12**, 7821–7839.

13 P. S. Nerenberg and T. Head-gordon, *J. Chem. Theory Comput.*, 2011, **7**, 1220–1230.

14 H. Nguyen, D. R. Roe and C. Simmerling, *J. Chem. Theory Comput.*, 2013, **9**, 2020–2034.

15 I. Maffucci and A. Contini, *J. Chem. Theory Comput.*, 2016, **12**, 714–727.

16 D. A. Case, V. Babin, J. T. Berryman, R. M. Betz, Q. Cai, D. S. Cerutti, T. E. Cheatham, III, T. A. Darden, R. E. Duke, H. Gohlke, A. W. Goetz, S. Gusarov, N. Homeyer, P. Janowski, J. Kaus, I. Kolossváry, A. Kovalenko, T. S. Lee, S. LeGrand, T. Luchko, R. Luo, B. X. Wu, P. A. Kollman, *Univ. California, San Fr.*, 2014.

17 T. Calinski and J. Harabasz, *Commun. Stat.*, 1974, **22**, 2577–2637.

18 W. Humphrey, A. Dalke and K. Schulten, *J. Mol. Graph.*, 1996, **14**, 33–38.

19 M. Crisma, M. De Zotti, A. Moretto, C. Peggion, B. Drouillat, K. Wright, F. Couty, C. Toniolo and F. Formaggio, *New J. Chem.*, 2015, **39**, 3208–3216.

20 A. Grossfield, *http //membrane.urmc.rochester.edu/content/wham*, 2015.
